# Temporal patterns of cancer burden in Asia, 1990–2019: a systematic examination for the Global Burden of Disease 2019 study

**DOI:** 10.1016/j.lansea.2023.100333

**Published:** 2024-01-02

**Authors:** GBD 2019 Asia and All Cancers Collaborators, Rajesh Sharma, Hedayat Abbastabar, Deldar Morad Abdulah, Hassan Abidi, Hassan Abolhassani, Zahra Abrehdari-Tafreshi, Abdorrahim Absalan, Hiwa Abubaker Ali, Eman Abu-Gharbieh, Juan Manuel Acuna, Nasrin Adib, Qorinah Estiningtyas Sakilah Adnani, Abbas Aghaei, Aqeel Ahmad, Sajjad Ahmad, Ali Ahmadi, Sepideh Ahmadi, Luai A. Ahmed, Marjan Ajami, Hanadi Al Hamad, Syed Mahfuz Al Hasan, Fahad Mashhour Alanezi, Adel Ali Saeed Al-Gheethi, Mohammed Khaled Al-Hanawi, Abid Ali, Beriwan Abdulqadir Ali, Yousef Alimohamadi, Syed Mohamed Aljunid, Sadeq Ali Ali Al-Maweri, Saleh A. Alqahatni, Mohammad AlQudah, Rajaa M. Al-Raddadi, Ala'a B. Al-Tammemi, Alireza Ansari-Moghaddam, Sumadi Lukman Anwar, Razique Anwer, Muhammad Aqeel, Jalal Arabloo, Morteza Arab-Zozani, Hany Ariffin, Al Artaman, Judie Arulappan, Tahira Ashraf, Elaheh Askari, Mohammad Athar, Maha Moh'd Wahbi Atout, Sina Azadnajafabad, Muhammad Badar, Ashish D. Badiye, Nayereh Baghcheghi, Sara Bagherieh, Ruhai Bai, Khuloud Bajbouj, Shrikala Baliga, Mainak Bardhan, Azadeh Bashiri, Pritish Baskaran, Saurav Basu, Uzma Iqbal Belgaumi, Amiel Nazer C Bermudez, Bharti Bhandari, Nikha Bhardwaj, Ajay Nagesh Bhat, Saeid Bitaraf, Archith Boloor, Milad Bonakdar Hashemi, Zahid A. Butt, Joshua Chadwick, Jeffrey Shi Kai Chan, Vijay Kumar Chattu, Pankaj Chaturvedi, William C.S. Cho, Aso Mohammad Darwesh, Nihar Ranjan Dash, Amin Dehghan, Arkadeep Dhali, Mostafa Dianatinasab, Mahmoud Dibas, Abhinav Dixit, Shilpi Gupta Dixit, Fariba Dorostkar, Haneil Larson Dsouza, Iffat Elbarazi, Noha Mousaad Elemam, Waseem El-Huneidi, Eyad Elkord, Omar Abdelsadek Abdou Elmeligy, Mohammad Hassan Emamian, Ryenchindorj Erkhembayar, Rana Ezzeddini, Zehra Fadoo, Razana Faiz, Ildar Ravisovich Fakhradiyev, Aida Fallahzadeh, MoezAlIslam Ezzat Mahmoud Faris, Hossein Farrokhpour, Ali Fatehizadeh, Hamed Fattahi, Ginenus Fekadu, Takeshi Fukumoto, Abhay Motiramji Gaidhane, Nasrin Galehdar, Priyanka Garg, Fataneh Ghadirian, Mansour Ghafourifard, MohammadReza Ghasemi, Mohammad Ghasemi Nour, Fariba Ghassemi, Maryam Gholamalizadeh, Asadollah Gholamian, Elena Ghotbi, Mahaveer Golechha, Pouya Goleij, Sahil Goyal, Mohammed Ibrahim Mohialdeen Gubari, D Sanjeeva Gunasekera, Damitha Asanga Gunawardane, Sapna Gupta, Parham Habibzadeh, Helia Sadat Haeri Boroojeni, Esam S. Halboub, Randah R. Hamadeh, Rifat Hamoudi, Mehdi Harorani, Mohammad Hasanian, Treska S. Hassan, Simon I. Hay, Mohammad Heidari, Mahsa Heidari-Foroozan, Kamran Hessami, Kamal Hezam, Yuta Hiraike, Ramesh Holla, Mohammad Hoseini, Md Mahbub Hossain, Sahadat Hossain, Vivian Chia-rong Hsieh, Junjie Huang, Nawfal R. Hussein, Bing-Fang Hwang, Farideh Iravanpour, Nahlah Elkudssiah Ismail, Masao Iwagami, Linda Merin J, Farhad Jadidi-Niaragh, Morteza Jafarinia, Mohammad Ali Jahani, Haitham Jahrami, Abhishek Jaiswal, Mihajlo Jakovljevic, Mahsa Jalili, Elham Jamshidi, Umesh Jayarajah, Shubha Jayaram, Sweety Suman Jha, Mohammad Jokar, Nitin Joseph, Ali Kabir, Md. Awal Kabir, Dler Hussein Kadir, Pradnya Vishal Kakodkar, Laleh R. Kalankesh, Leila R. Kalankesh, Rohollah Kalhor, Feroze Kaliyadan, Vineet Kumar Kamal, Zul Kamal, Ashwin Kamath, Sitanshu Sekhar Kar, Hanie Karimi, Navjot Kaur, Leila Keikavoosi-Arani, Mohammad Keykhaei, Yousef Saleh Khader, Himanshu Khajuria, Ejaz Ahmad Khan, M Nuruzzaman Khan, Maseer Khan, Moien A.B. Khan, Yusra H. Khan, Shaghayegh Khanmohammadi, Moawiah Mohammad Khatatbeh, Sorour Khateri, Maryam Khayamzadeh, Hamid Reza Khayat Kashani, Min Seo Kim, Farzad Kompani, Hamid Reza Koohestani, Sindhura Lakshmi Koulmane Laxminarayana, Kewal Krishan, Narinder Kumar, Naveen Kumar, Tezer Kutluk, Ambily Kuttikkattu, Daphne Teck Ching Lai, Dharmesh Kumar Lal, Faris Hasan Lami, Savita Lasrado, Sang-Woong Lee, Seung Won Lee, Yeong Yeh Lee, Yo Han Lee, Elvynna Leong, Ming-Chieh Li, Jue Liu, Farzan Madadizadeh, Ahmad R. Mafi, Soleiman Mahjoub, Reza Malekzadeh, Ahmad Azam Malik, Iram Malik, Tauqeer Hussain Mallhi, Mohammad Ali Mansournia, Santi Martini, Elezebeth Mathews, Manu Raj Mathur, Jitendra Kumar Meena, Ritesh G. Menezes, Reza Mirfakhraie, Seyed Kazem Mirinezhad, Mohammad Mirza-Aghazadeh-Attari, Prasanna Mithra, Ashraf Mohamadkhani, Soheil Mohammadi, Maryam Mohammadzadeh, Syam Mohan, Ali H. Mokdad, Ahmed Al Montasir, Fateme Montazeri, Maryam Moradi, Mostafa Moradi Sarabi, Farhad Moradpour, Maliheh Moradzadeh, Paula Moraga, Abbas Mosapour, Majid Motaghinejad, Sumaira Mubarik, Jibran Sualeh Muhammad, Christopher J.L. Murray, Ahamarshan Jayaraman Nagarajan, Mohsen Naghavi, Shumaila Nargus, Zuhair S. Natto, Biswa Prakash Nayak, Seyed Aria Nejadghaderi, Phuong The Nguyen, Robina Khan Niazi, Nafise Noroozi, Hassan Okati-Aliabad, Akinkunmi Paul Okekunle, Sokking Ong, Anu Mary Oommen, Jagadish Rao Padubidri, Ashok Pandey, Eun-Kee Park, Seoyeon Park, Siddhartha Pati, Shankargouda Patil, Rajan Paudel, Uttam Paudel, Majid Pirestani, Indrashis Podder, Ghazaleh Pourali, Mona Pourjafar, Akram Pourshams, Zahiruddin Quazi Syed, Raghu Anekal Radhakrishnan, Venkatraman Radhakrishnan, Mosiur Rahman, Shayan Rahmani, Vahid Rahmanian, Pushkal Sinduvadi Ramesh, Juwel Rana, Indu Ramachandra Rao, Sowmya J. Rao, Sina Rashedi, Mohammad-Mahdi Rashidi, Nazila Rezaei, Negar Rezaei, Nima Rezaei, Saeid Rezaei, Mohsen Rezaeian, Gholamreza Roshandel, S.N. Chandan, Maha Mohamed Saber-Ayad, Siamak Sabour, Leila Sabzmakan, Basema Saddik, Umar Saeed, Sher Zaman Safi, Fatemeh Saheb Sharif-Askari, Amirhossein Sahebkar, Harihar Sahoo, Seyed Aidin Sajedi, Mirza Rizwan Sajid, Mohammad Amin Salehi, Amir Salek Farrokhi, Made Ary Sarasmita, Saman Sargazi, Gargi Sachin Sarode, Sachin C. Sarode, Brijesh Sathian, Maheswar Satpathy, Prabhakar Semwal, Subramanian Senthilkumaran, Sadaf G. Sepanlou, Melika Shafeghat, Saeed Shahabi, Ataollah Shahbandi, Fariba Shahraki-Sanavi, Masood Ali Shaikh, Mohammed Shannawaz, Rahim Ali Sheikhi, Parnian Shobeiri, Seyed Afshin Shorofi, Sunil Shrestha, Soraya Siabani, Garima Singh, Paramdeep Singh, Surjit Singh, Dhirendra Narain Sinha, Samarjeet Singh Siwal, Saraswathy Sreeram, Muhammad Suleman, Rizwan Suliankatchi Abdulkader, Iyad Sultan, Abida Sultana, Mohammad Tabish, Takahiro Tabuchi, Majid Taheri, Iman M. Talaat, Arash Tehrani-Banihashemi, Mohamad-Hani Temsah, Pugazhenthan Thangaraju, Nihal Thomas, Nikhil Kenny Thomas, Amir Tiyuri, Ruoyan Tobe-Gai, Razie Toghroli, Marcos Roberto Tovani-Palone, Sana Ullah, Bhaskaran Unnikrishnan, Era Upadhyay, Sahel Valadan Tahbaz, Rohollah Valizadeh, Shoban Babu Varthya, Yasir Waheed, Song Wang, Dakshitha Praneeth Wickramasinghe, Nuwan Darshana Wickramasinghe, Hong Xiao, Naohiro Yonemoto, Mustafa Z. Younis, Chuanhua Yu, Mazyar Zahir, Nazar Zaki, Maryam Zamanian, Zhi-Jiang Zhang, Hanqing Zhao, Osama A. Zitoun, Mohammad Zoladl

**Keywords:** Cancer burden, Asia, Incidence, Mortality, Disability adjusted life years, Global burden of disease

## Abstract

**Background:**

Cancers represent a challenging public health threat in Asia. This study examines the temporal patterns of incidence, mortality, disability and risk factors of 29 cancers in Asia in the last three decades.

**Methods:**

The age, sex and year-wise estimates of incidence, mortality, and disability-adjusted life years (DALYs) of 29 cancers for 49 Asian countries from 1990 through 2019 were generated as a part of the Global Burden of Disease, Injuries and Risk Factors 2019 study. Besides incidence, mortality and DALYs, we also examined the cancer burden measured in terms of DALYs and deaths attributable to risk factors, which had evidence of causation with different cancers. The development status of countries was measured using the socio-demographic index. Decomposition analysis was performed to gauge the change in cancer incidence between 1990 and 2019 due to population growth, aging and age-specific incidence rates.

**Findings:**

All cancers combined claimed an estimated 5.6 million [95% uncertainty interval, 5.1–6.0 million] lives in Asia with 9.4 million [8.6–10.2 million] incident cases and 144.7 million [132.7–156.5 million] DALYs in 2019. The age-standardized incidence rate (ASIR) of all cancers combined in Asia was 197.6/100,000 [181.0–214.4] in 2019, varying from 99.2/100,000 [76.1–126.0] in Bangladesh to 330.5/100,000 [298.5–365.8] in Cyprus. The age-standardized mortality rate (ASMR) was 120.6/100,000 [110.1–130.7] in 2019, varying 4-folds across countries from 71.0/100,000 [59.9–83.5] in Kuwait to 284.2/100,000 [229.2–352.3] in Mongolia. The age-standardized DALYs rate was 2970.5/100,000 [2722.6–3206.5] in 2019, varying from 1578.0/100,000 [1341.2–1847.0] in Kuwait to 6574.4/100,000 [5141.7–8333.0] in Mongolia. Between 1990 and 2019, deaths due to 17 of the 29 cancers either doubled or more, and 20 of the 29 cancers underwent an increase of 150% or more in terms of new cases. Tracheal, bronchus, and lung cancer (both sexes), breast cancer (among females), colon and rectum cancer (both sexes), stomach cancer (both sexes) and prostate cancer (among males) were among top-5 cancers in most Asian countries in terms of ASIR and ASMR in 2019 and cancers of liver, stomach, hodgkin lymphoma and esophageal cancer posted the most significant decreases in age-standardized rates between 1990 and 2019. Among the modifiable risk factors, smoking, alcohol use, ambient particulate matter (PM) pollution and unsafe sex remained the dominant risk factors between 1990 and 2019. Cancer DALYs due to ambient PM pollution, high body mass index and fasting plasma glucose has increased most notably between 1990 and 2019.

**Interpretation:**

With growing incidence, cancer has become more significant public health threat in Asia, demanding urgent policy attention and guidance. Its heightened risk calls for increased cancer awareness, preventive measures, affordable early-stage detection, and cost-effective therapeutics in Asia. The current study can serve as a useful resource for policymakers and researchers in Asia for devising interventions for cancer management and control.

**Funding:**

The GBD study is funded by the 10.13039/100000865Bill and Melinda Gates Foundation.

**Research in context**

**Evidence before this study**

Cancer is one of the leading non-communicable diseases in Asia. Previously, Global Burden of Disease, Injuries and Risk Factors 2017 Study (GBD 2017) provided estimates for cancer incidence, deaths and disability-adjusted life years (DALYs) for the period 1990 to 2017. Apart from GBD 2017, the International Agency for Research on Cancer (IARC) provided estimates for cancer for the year 2020 under the GLOBOCAN project. The present study is a part of the Global Burden of Disease, Injuries and Risk Factors 2019 (GBD 2019), which produced estimates for 302 causes of death, 369 diseases and injuries and 87 risk factors for 204 countries and territories for 1990–2019.

**Added value of this study**

In this study, we examined age, sex and location-wise estimates of incidence, deaths and DALYs for 49 countries and territories in Asia between 1990 and 2019. While GLOBOCAN 2020 provides cancer estimates for 2020, the GBD project provides entire time series estimates from 1990 through 2019 for all countries and territories. Apart from estimates of incident cases, deaths and age-standardized rates, as is done in GLOBOCAN 2020, we also estimated the burden of deaths and disability quantified using DALYs—defined as the sum of years of life lost due to premature death and years lived with disability. The cancer burden was also examined in the light of country-level development measured by socio-demographic index. The contribution of main risk factors in cancer DALYs was examined by sex in 49 countries. Decomposition analysis was performed to examine how much of the increased incidence between 1990 and 2019 was due to population growth, population aging and changes in age-specific incidence rates.

**Implications of all the available evidence**

Between 1990 and 2019, all-age death counts of 17 of the 29 cancers either doubled or more, and 20 of the 29 cancers underwent an increase of 150% or higher in terms of all-age incidence counts. Country-wise, cancer incidence doubled or more in 38 of 49 countries. Risk factor analysis indicated that smoking, alcohol use and ambient particulate matter pollution resulted in maximum DALYs and deaths and cancer burden due to high body-mass index and high fasting plasma glucose increased substantially between 1990 and 2019. Public health interventions are required to stem the rising cancer burden in Asia focussing on cancer awareness, screening and prevention through the containment of modifiable risk factors, timely referral and availability of cost-effective cancer infrastructure including cancer registries.

## Introduction

Asia is witnessing an epidemiological transition characterized by declining fertility rates, increasing life spans, and decreasing mortality rates along with a shifting burden of infectious diseases towards non-communicable diseases (NCDs) such as cardiovascular diseases, cancer, and diabetes mellitus.^1^ Among NCDs, cancer incidence has substantially increased ubiquitously worldwide and it has become a significant public health threat in both developed and developing countries.^2^ The fast-rising cancer burden in Asia has been attributed to rising incomes leading to changes in lifestyles (e.g., sedentary behavior, a diet rich in fats and low in fruits/vegetables), obesity, tobacco use, alcohol consumption, urbanization, and increasing longevity.^3^^,^^4^ Rapid economic growth in several Asian countries have also allowed them to invest on health infrastructure and cancer care. Therefore, cancer incidence might have also increased due to better access, population-based screening or opportunistic screening in conjunction with universal health coverage or coverage of treatment expenses, and improved cancer registration in several Asian countries.

Cancer poses significant policy challenges in low and middle-income countries (LMICs) in Asia due to increasing incidence, higher mortality and lower survival rates than in high-income countries (HICs).^2^^,^^5^^,^^6^ Moreover, many cancers require intensive and multi-model treatment resulting in substantial productivity losses, and treatment and ancillary costs, which are mostly incurred from the patient's pocket in LMICs.^4^^,^^7^ A recent study predicted that the total economic cost of cancers worldwide would be $25.1 trillion between 2020 and 2050, of which, China and India are predicted to account for $5.3 trillion and $1.4 trillion, respectively.^8^ To combat the unique, yet diverse, and formidable public health threat of cancer, it is pertinent to understand the cancer epidemiology in Asia and its temporal patterns to devise strategies and policy interventions in a country-specific manner.

Previous studies examined the cancer burden at the global level^2^^,^^9^ or in individual countries of Asia or have focused on individual cancers in Asia such as breast cancer,^10^ prostate cancer,^11^ lung cancer,^12^ colorectal cancer^13^ and oral cancer.^14^ Departing from individual country-specific or cancer-specific studies, we examined the temporal patterns of 29 cancers in 49 Asian countries between 1990 and 2019 using estimates from the Global Burden of Disease, Injuries and Risk Factors 2019 Study (GBD 2019).^1^^,^^15^^,^^16^ Specifically, we examined the cancer burden in Asia, focussing on six primary dimensions. First, we investigated the temporal patterns of aggregate cancer burden in Asia in the last three decades. Second, we examined the burden of leading cancers in 2019 while paying attention to the cancers exerting the most significant burden in terms of incidence and deaths. Third, we examined the cancer burden across 49 countries in 2019, while also examining the leading cancers in each country. We also conducted decomposition analysis to understand how much of the incidence change between 1990 and 2019 occurred due to three factors: population growth, population aging and change in age-specific incidence rates. Changes due to each of these three factors would imply policy implications in a country-specific manner. Fourth, we investigated the age-wise distribution of all cancers combined as well as different cancers in Asia, which helps us understand the age groups most affected by different cancers. Fifth, we examined the association between age-standardized rates of 29 cancers and country's development status measured using socio-demographic index (SDI). Lastly, we examined the cancer disability-adjusted life years (DALYs) and deaths attributable to 34 risk factors between 1990 and 2019.

This comprehensive and comparable report on the cancer burden across Asian countries, providing past trends and progress is vital to gauge current policy focus and attention to deal with the massive public health challenge as severe as cancer in Asia.

## Methods

### Overview of data

We employed cancer estimates from the GBD 2019 study for 49 countries on the Asian continent for 29 cancers^17^ from the GBD results tool (https://vizhub.healthdata.org/gbd-results). The 49 countries in Asia are considered as classified by the United Nations statistical division (Appendix pp 5–6). To arrive at mortality estimates, GBD utilizes data from all sources including vital registration, national and subnational cancer registries and verbal autopsy. For GBD 2019, a total of 929193 cancer, location and year-specific sources of data were used (vital registration: 767514; cancer registries: 155542; verbal autopsy: 6137).

Disease and injuries in the GBD 2019 study are organized into a comprehensive hierarchy of four nested levels. Level 1 captures the burden at a broader level into three categories: a) Communicable, maternal, neonatal and nutritional diseases, b) Non-communicable Diseases and c) Injuries. Level 1 categories are further sub-divided into 22 subgroups termed as Level 2 and neoplasms are one of the 22 level 2 groups. Level 3 includes further disaggregation of disease burden. For instance, cancers are classified into 30 cancer groups under Level 3 (e.g., breast). Level 4 includes further disaggregation, for instance, leukemia is further classified into 4 groups (e.g., chronic myeloid leukemia). The list of 30 cancers is provided in Appendix (p 7); however, to focus on malignant cancers, we didn't report and examine the cancer group classified as “other neoplasms” which are further subdivided into: a) Myelodysplastic, myeloproliferative and other hematopoietic neoplasms b) Benign and insitu intestinal neoplasms c) Benign and insitu cervical and uterine neoplasms d) other benign and insitu neoplasms. Therefore, for the aggregate burden in Asia as well as in individual countries, we examined estimates of “Total cancers” from the GBD results tool. Additionally, we focus on overall estimates for liver cancer, leukemia and non-melanoma skin cancer; however, GBD results tool also provides age, sex and country-wise data on non-melanoma skin cancer segregated into squamous cell and basal cell; liver cancer segregated into primary liver cancer due to hepatitis B, hepatitis C, alcohol use, non-alcoholic steatohepatitis (NASH) and due to other causes; and leukemia segregated into acute lymphoid, chronic lymphoid, acute myeloid, chronic myeloid and other leukemia. Country-wise estimates of etiologies of liver cancer, leukemia, other neoplasms and non-melanoma skin cancer can be accessed via GBD results tool (https://vizhub.healthdata.org/gbd-results).

### GBD estimation framework

The GBD estimation framework and calculation of various metrics are detailed elsewhere^1^^,^^2^^,^^15^^,^^16^; here, we briefly discuss how GBD estimates the cancer burden measured in terms of incidence, mortality and DALYs due to cancers. GBD estimation framework begins with the estimation of mortality using data from cancer registries. The country-wise cancer registry data from Asia employed in GBD 2019 study is listed in Supplementary Table S3 (Appendix pp 8–16). First, we created the mortality-to-incidence ratio (MIR) by matching the incidence and mortality data from cancer registries by cancer, age, sex, year, and location.^2^ Final MIR estimates were obtained by fitting a linear step mixed-effects model using the healthcare access and quality (HAQ) index^18^ as a covariate. The HAQ index is developed by the GBD and is measured on a scale from 0 (worst) to 100 (best) and the index values are based on mortality data from 32 causes of death, which could be avoided by timely and effective medical care (termed as ‘amenable mortality’).^18^ These MIR estimates were smoothed using spatio-temporal Gaussian Process Regression (ST-GPR)^19^; the resulting MIR estimates were multiplied with incidence data from cancer registries to generate mortality estimates. These mortality estimates were used as inputs for the cause of death ensemble model (CODEm).^20^ The CODEm is an ensemble modelling framework that provides mortality estimates based on large number of models, where model construction and performance is evaluated through out-of-sample predictive validity.^2^ The CODEm produces mortality estimates using mortality input from the previous step, along with mortality data from vital registration systems and verbal autopsy reports along with location-level covariates, which have been found to have a plausible relation with death due to particular cancer (see eTable 7 of Kocarnik et al^2^ for the list of covariates for each cancer). The mortality estimates from the CODEm were divided by the MIR generated by ST-GPR to generate the estimates of incidence. The final mortality estimates from the CODEm were combined with reference life tables to generate the estimates of years of life lost (YLLs).

Ten-year prevalence was calculated using incidence, mortality and estimated relative survival curves and their respective correlation with modelled MIRs from ST-GPR.^2^ Ten-year prevalence is divided into four sequelae: diagnosis and treatment, remission, metastatic and disseminated phase and terminal phase.^2^ Years of life lived with disability (YLDs) were obtained by multiplying disability weights with sequelae prevalence from previous step, where disability weights reflect the severity of health loss on a scale of 0 (full health) to 1 (death).^1^^,^^2^ The YLD estimates were added to YLL estimates to produce the DALY estimates—one DALY is equivalent to one year of healthy life lost.^1^

In addition to all-age and age-wise counts of incidence, mortality and DALYs, we also reported and compared the age-standardized rates of these metrics and the age-standardization was done using GBD reference population.^15^ GBD aims to capture data uncertainty due to data quality and availability in each modelling step; therefore, all the estimates reported in this paper are provided with 95 percent uncertainty intervals (UIs), which were calculated based on 2.5th and 97.5th percentile from 1000 draws in each modelling step; we report these UIs inside square brackets along with mean estimates. Those percent changes are interpreted as statistically significant which doesn't include zero in the UI.

### Socio-demographic index (SDI)

We used SDI to measure a country's development status.^2^^,^^15^ The SDI, proposed and developed by GBD, is a composite indicator made up of three indicators: lag distributed income per capita, mean education for those aged 15 and above, and the total fertility rate (aged below 25). Each of these three indicators is rescaled on 0 (lowest) to 1 (highest) scale, and the geometric mean of the three indices produces the final SDI.

### Risk factors

The GBD risk factors methodology is detailed elsewhere,^16^^,^^21^ here, we briefly outline the steps involved in GBD risk factors estimation. The GBD risk estimation framework is based on comparative risk assessment framework, which begins with the identification of risk-outcome pairs, which meet the criteria of plausible or convincing evidence as per World Cancer Research Fund^22^ (Appendix pp 17–18). Second step involves the calculation of relative risk (RR) as a function of exposure for each risk-outcome combination. The exposure levels of risk factors are modelled for each age, sex, location, and year using data from published literature, household surveys, censuses, remote sensing data, ground monitor data, and administrative records. Fourth, for each risk factor, theoretical minimum risk exposure level (TMREL) is calculated, which is the plausible level of risk exposure posing minimum risk for the population. For risk factors having monotonically increasing risk functions, the TMREL was set to 0. For risk factors having risk function in the shape of J or U (e.g., sodium and ischemic heart disease; body-mass index and ischemic heart disease), the lowest point of the risk function provided the TMREL (Appendix pp 19–24^16^). The fifth step involves estimation of population attributable fraction (PAF) as a function of RR, TMREL and exposure levels. The PAFs are multiplied by the YLLs, YLDs, DALYs and deaths to generate the burden of cancer attributable to the particular risk factor. Sixth step involves calculation of PAF and attributable burden for combination of risk factors. GBD 2019 framework involves 87 risk factors, out of which, 34 risk factors had non-zero cancer DALYs in Asia; in this study, we examine the burden of cancers linked with these 34 risk factors in terms of DALYs and deaths. The cancer burden of different risk factors measured in terms of YLLs and YLDs are also available online from the GBD results tool (https://vizhub.healthdata.org/gbd-results).

### Decomposition analysis

We conducted decomposition analysis to understand how much of the increased incidence between 1990 and 2019 occurred due to three different factors: population growth, population aging and age-specific incidence rates. For this purpose, we adopted an approach in which two out of three factors are kept constant and effect of the third is observed.^23^ Specifically, incident cases were calculated as per two different scenarios: 1) age-specific incidence rates and age structure (composition) of 1990 were applied to the total population in 2019; 2) age-specific incidence rates of 1990 were adapted to the population age structure (composition) and total population of 2019. Incidence cases in 1990 were subtracted from incident cases from scenario 1 to generate changes in incident cases due to population growth. Difference between incident cases from scenario 2 and scenario 1 defined the change in incident cases due to population aging and the rest of the change was attributed to changes in age-specific incidence rates (incident cases in 2019 − incident cases from scenario 2).

The data analysis and visualization in this paper were done using statistical software R (version 4.1.1), Stata (version 13.0), and Python (version 3.8.8).

### Role of funding source

The GBD study is funded by the Bill & Melinda Gates Foundation. The funders of the study had no role in study design; collection, analysis, and interpretation of data; or writing of the report. The corresponding author had full access to the data and had responsibility for final submission of the manuscript.

## Results

### Aggregate burden

**Fig. 1:**
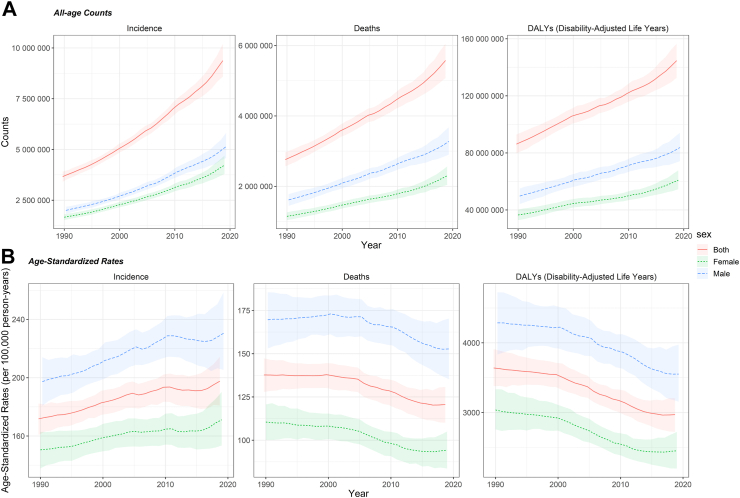
**Temporal patterns of cancer burden in Asia, 1990–2019 A) all-age counts B) age-standardized rates (per 100,000)**. Data source: Global burden of disease, injuries and risk factors 2019 study.

In Asia, there were 9.4 million [95% UI, 8.6–10.2 million] new cancer cases in 2019, having more than doubled in comparison to 1990 (3.7 million [3.4–3.9 million]) (Fig. 1A). In 2019, all cancers resulted in an estimated 5.6 million [5.1–6.0 million] deaths in Asia up from 2.8 million [2.6–3.0 million] in 1990. The burden of cancers measured in terms of DALYs increased from 86.2 million [79.8–92.9 million] in 1990 to 144.7 million [132.7–156.5 million] in 2019 (Fig. 1A). Among 22 GBD level 2 causes, the rank of cancer, in terms of DALYs, has increased in Asia from seventh to second between 1990 and 2019, behind only cardiovascular diseases (243.0 million [224.6–260.7 million]) (Appendix pp 25–26).

The age-standardized incidence rate (ASIR) rose from 171.7/100,000 [160.8–182.3] in 1990 to 197.6/100,000 [181.0–214.4] in 2019; in contrast, the age-standardized mortality rate (ASMR) of all cancers combined, declined from 137.7/100,000 [128.0–147.3] in 1990 to 120.6/100,000 [110.1–130.7] in 2019 (Fig. 1B). The age-standardized DALYs rate (ASDALR) also showed a slight reduction between 1990 and 2019 (1990: 3639.5/100,000 [3378.2–3909.8]; 2019: 2970.5/100,000 [2722.6–3206.5]).

### Leading cancers

Supplementary Table S7 illustrates the burden of 29 cancers in Asia in 2019 for both sexes combined, males and females (Appendix pp 27–28). In 2019, for both sexes combined, 23 of 29 cancers recorded more than 100,000 new cases, 15/29 cancers registered more than 100,000 deaths and 22/29 cancers registered more than 1 million DALYs in 2019.

For both sexes combined, tracheal, bronchus, and lung (TBL) was the leading cancer in Asia, resulting in an estimated 1.3 million [1.2–1.5 million] cases, 1.2 million [1.1–1.3 million] deaths and 27.1 million [24.0–30.4 million] DALYs in 2019 (Supplementary Table S7, Appendix pp 27–28). It was also the most frequent cancer in males (906,600: [771,000–1.05 million]) and third most frequent cancer among females with 398,900 [339,700–464,500] cases. TBL is estimated to have caused the highest number of cancer deaths among both males (825,700 [706,200–949,800]) and females (364,800 [312,100–421,100]) in 2019.

Colon and rectum cancer (CRC) was the second leading cancer in terms of incident cases with 1.1 million [1.0–1.2 million] cases and third leading in terms of DALYs (13.4 million [12.1–14.6 million]) and all-age cancer deaths (560,400 [504,600–609,800]). CRC was the second most frequent cancer among males (677,500 [591,900–779,100] cases) and females (446,100 [388,200–505,600] cases) in 2019. CRC caused 321,200 [283,400–359,500] and 239,200 [207,800–268,000] deaths among males and females, respectively, in 2019.

Stomach cancer was third-ranked cancer in terms of incidence (930,100 [821,900–1.05 million]) and second leading in terms of deaths (672,800 [597,000–746,900]) and DALYs (15.9 million [14.1–17.7 million]) in 2019. It caused 443,800 [379,900–509,600] and 229,000 [198,500–261,500] deaths among males and females, respectively, in 2019.

Breast cancer was the leading cancer among females in terms of new cases (914,900 [815,800–1.03 million]) and second leading in terms of cancer deaths (337,800 [301,500–375,300]) in 2019. Breast cancer was responsible for the highest DALYs among females (10.9 million [9.8–12.1 million]). Non-melanoma skin cancer was ranked fifth in terms of absolute count of incident cases in both sexes combined (525,700 [461,000–589,000]). Other leading cancers in both sexes combined were esophageal cancer with 391,200 [332,600–449,300] new cases and 361,100 [307,600–411,800] deaths and liver cancer with 381,900 [342,400–429,600] new cases and 340,600 [305,200–376,900] deaths in 2019.

In terms of age-standardized rates, TBL cancer was again the leading cancer with ASIR of 27.8/100,000 [24.6–31.0], followed by CRC (23.9/100,000 [21.5–26.4]), stomach cancer (19.8/100,000 [17.5–22.3]), breast cancer (18.5/100,000 [16.5–20.7]) and non-melanoma skin cancer (11.3/100,000 [10.0–12.7]) (Appendix pp 29–30). The ASMR was the highest in the case of TBL (25.8/100,000 [22.8–28.7]), followed by stomach (14.7/100,000 [13.0–16.2]), CRC (12.5/100,000 [11.2–13.6]), esophageal (7.7/100,000 [6.6–8.8]) and liver cancer (7.2/100,000 [6.5–7.9]) (Appendix pp 29–30). In terms of ASDALR too, TBL (553.7/100,000 [488.6–619.8]) was again the leading cancer, followed by stomach cancer (324.7/100,000 [288.6–360.6]) and CRC (275.5/100,000 [249.6–299.4]) in 2019.

**Fig. 2:**
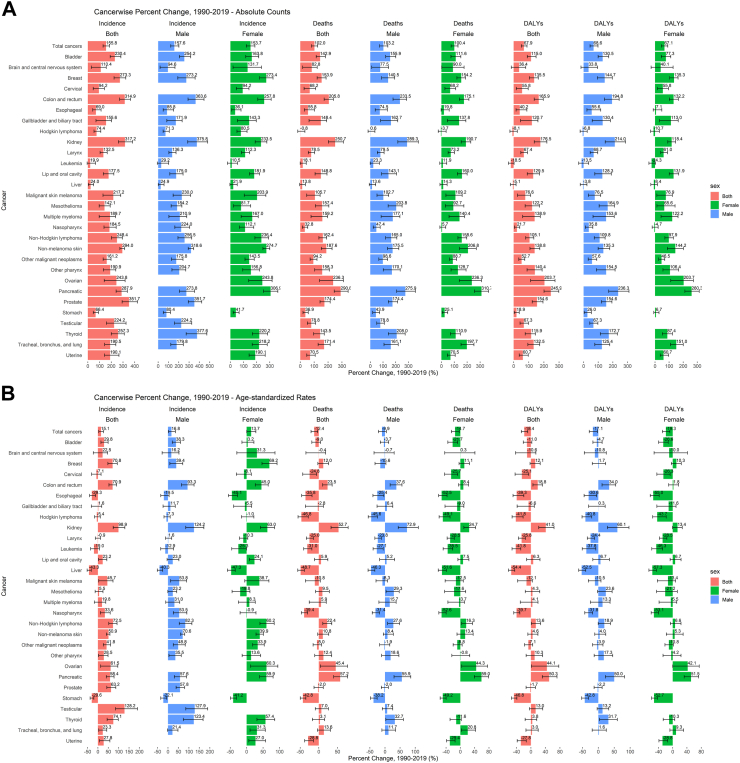
**Percent Change of 29 cancers between 1990 and 2019 A) Counts B) Age-standardized Rates (per 100,000)**. DALYs: Disability-adjusted-life-years. Data Source: Global Burden of Disease, Injuries and Risk Factors 2019 Study.

Between 1990 and 2019, all-age death counts either doubled or more in 17 of the 29 cancers led by pancreatic cancer (290.8% [244.3%–344.2%]) (Fig. 2A). Twenty cancers underwent an increase of 150% or higher in terms of incidence count led by prostate cancer, which posted an incidence growth of 351.7% [292.6%–423.0%], followed by kidney cancer (317.2% [261.5%–381.5%]) (Fig. 2A). No cancer underwent a reduction in incidence with the least growth posted by leukemia at 19.9% [−6.6% to 65.9%]. In terms of DALYs, 16 cancers underwent an increase of 100% or more between 1990 and 2019, led by pancreatic cancer (245.9% [200.9%–298.1%]) and ovarian cancer (203.7% [112.0%–273.1%]). Between 1990 and 2019, the ASIR increased by more than 50% in 10/29 cancers led by testicular cancer (128.2% [84.9%–190.4%]), followed by kidney cancer (98.9% [73.4%–129.9%]) (Fig. 2B). Thyroid, non-hodgkin lymphoma, CRC, breast, prostate and pancreatic cancers also registered large increases in ASIR between 1990 and 2019. From 1990 through 2019, the ASIR decreased the most in the case of liver cancer (−43.3% [−32.2% to −52.3%]), followed by stomach cancer (−29.6% [−19.6% to −37.8%]). In terms of ASMR, the increases were modest (<50%) in most cancers, except kidney cancer and pancreatic cancer (growth >50%). The ASMR decreased in 9/29 cancers (statistically significant) between 1990 and 2019 (Fig. 2B). Out of 29 cancers, the ASDALR decreased (statistically significant) in 8 cancers between 1990 and 2019. The gender-specific cancers such as testicular and prostate cancer witnessed significant increases between 1990 and 2019, whereas breast cancer among females increased substantially along with reduction in age-standardized rates of cervical cancer (Fig. 2).

In 1990, stomach cancer was the leading cancer in Asia in terms of ASIR, but due to a substantial reduction between 1990 and 2019, it was ranked third in 2019, behind TBL and CRC. Notably, CRC witnessed one of the most significant increases (1990: 14.0/100,000 [13.2–14.8]; 2019: 23.9/100,000 [21.5–26.4]) such that its rank rose from 4th to 2nd between 1990 and 2019 (Supplementary Table S9, Appendix pp 31–32).

For majority of cancers, much of the increase came from population aging and changes in age-specific incidence rates (Supplementary Table S10, Appendix pp 33–34). For stomach and esophageal cancer, some increase due to population aging was negated by reduction in age-specific incidence rates. Similarly, liver cancer incidence increased by only 24.0% between 1990 and 2019, as the increase due to population growth and population aging was negated by reduction in age-specific incidence rates. For majority of cancers, three forces (population growth, aging and age-specific incidence rates) worked in unison, whereas changes in age-specific rates governed major changes in cancers such as breast cancer (among females), kidney cancer (both sexes), CRC (among males), prostate cancer (among males) and thyroid cancer (both sexes) (Supplementary Table S10, Appendix pp 33–34). For instance, in case of kidney cancer (both sexes combined), major increment in incident cases occurred due to age-specific rates (208.6%). In case of leukemia (both sexes combined), the increase in incident cases due to population growth was offset to some extent by reduction in age-specific rates.

### Country-wise burden

In terms of all-age counts, China was the top-ranked country with 4.8 million [4.1–5.5 million] new cases and 2.7 million [2.3–3.1 million] deaths, followed by India with 1.2 million [1.1–1.4 million] new cases and 929,600 [810,200–1.07 million] deaths in 2019 (Table 1). Japan was the third leading country with 887,300 [737,600–1.0 million] new cases and 437,700 [370,800–473,100] deaths behind China and India. Out of 49 countries, 15 had DALYs count more than 1 million led by China (67.3 million [57.3–78.1 million]) and India (27.1 million [23.7–31.3 million]). In 2019, the ASIR varied 3-folds across Asian countries, spanning from 99.2/100,000 [76.1–126.0] in Bangladesh to 330.5/100,000 [298.5–365.8] in Cyprus. The ASMR varied 4-folds across countries, spanning from 71.0/100,000 [59.9–83.5] in Kuwait to 284.2/100,000 [229.2–352.3] in Mongolia. The ASDALR varied from 1578.0/100,000 [1341.2–1847.0] in Kuwait to 6574.4/100,000 [5141.7–8333.0] in Mongolia (Table 1; See Supplementary Tables S11 and S12 for sex-wise burden of cancers in all countries in 2019, Appendix pp 35–44).

**Table 1:** Cancer burden by country, Both Sexes.

Location	Incidence				Deaths				Disability-adjusted life years			
	Counts, 2019 (in thousands)	Percent change, 1990–2019	Age-standardized rate (per 100,000) 2019	Percent change, 1990–2019	Counts, 2019 (in thousands)	Percent change, 1990–2019	Age-standardized rate (per 100,000) 2019	Percent change, 1990–2019	Counts, 2019 (in thousands)	Percent change, 1990–2019	Age-standardized rate (per 100,000) 2019	Percent change, 1990–2019
**Central Asia**												
Kazakhstan	36.5 [32.3–41.0]	13.3 [0.2–28.1]	204.6 [181.6–229.3]	−15.6 [−25.0 to −5.1]	21.0 [18.5–23.8]	−8.1 [−19.6 to 5.0]	123.0 [108.3–138.9]	−30.9 [−39.4 to −21.6]	599.2 [522.8–681.8]	−12.9 [−24.1 to 0.0]	3245.2 [2835.1–3682.2]	−34.6 [−43.0 to −25.1]
Kyrgyzstan	7.0 [6.2–7.8]	16.6 [3.6–30.4]	143.4 [128.4–160.0]	−23.3 [−31.6 to −14.5]	4.1 [3.7–4.7]	0.9 [−12.1 to 14.5]	90.4 [79.9–101.6]	−31.4 [−39.9 to −22.4]	125.0 [110.2–141.2]	−2.8 [−15.6 to 10.8]	2391.3 [2112.0–2696.6]	−37.9 [−45.9 to −29.3]
Tajikistan	8.3 [7.0–9.9]	59.2 [34.7–91.8]	152.3 [129.7–182.0]	−6.8 [−20.5 to 10.9]	5.4 [4.4–6.6]	47.8 [21.2–81.7]	111.4 [92.9–135.7]	−7.4 [−23.2 to 13.2]	179.9 [148.8–221.9]	46.8 [20.5–81.0]	2836.0 [2335.7–3480.4]	−20.1 [−34.2 to −2.4]
Turkmenistan	6.4 [5.4–7.7]	68.6 [41.5–104.4]	149.4 [125.7–178.4]	−13.1 [−26.1 to 3.8]	3.6 [3.0–4.5]	41.3 [13.6–75.4]	89.4 [73.3–110.7]	−28.8 [−42.0 to −12.4]	121.3 [97.5–151.1]	40.1 [11.8–75.3]	2647.5 [2149.2–3291.2]	−26.5 [−40.7 to −8.7]
Uzbekistan	39.6 [33.9–45.5]	100.1 [71.4–131.1]	169.1 [147.2–191.8]	10.2 [−4.0 to 25.1]	22.3 [18.8–25.9]	72.7 [45.0–101.6]	108.3 [93.1–123.5]	1.9 [−12.2 to 16.7]	772.3 [648.2–902.6]	75.5 [47.5–106.1]	2913.6 [2474.6–3371.0]	−7.2 [−21.3 to 7.7]
**Eastern Asia**												
China	4758.2 [4111.6–5489.4]	170.1 [124.5–227.3]	244.7 [212.1–281.1]	24.2 [4.2–49.5]	2711.9 [2311.2–3132.4]	86.1 [53.9–125.6]	140.7 [120.4–161.6]	−19.1 [−32.5 to −2.7]	67340.3 [57261.6–78067.8]	49.6 [23.8–82.3]	3411.1 [2923.2–3934.2]	−27.3 [−39.7 to −11.8]
Japan	887.3 [737.6–1028.4]	96.6 [67.0–126.6]	278.1 [235.5–322.1]	1.8 [-12.1 to 17.1]	437.7 [370.8–473.1]	81.2 [60.9–92.2]	112.5 [99.9–119.2]	−23.1 [−28.1 to −20.2]	7328.6 [6589.4–7750.7]	28.6 [18.4–34.1]	2475.8 [2298.4–2586.1]	−27.9 [−31.0 to −25.8]
Mongolia	7.2 [5.7–9.1]	115.7 [68.7–176.6]	313.5 [253.8–386.3]	1.1 [−19.6 to 28.6]	6.0 [4.7–7.6]	104.1 [56.2–165.6]	284.2 [229.2–352.3]	0.6 [−20.8 to 28.8]	170.8 [132.0–222.6]	106.8 [55.4–173.6]	6574.4 [5141.7–8333]	−7.4 [−29.0 to 20.5]
North Korea	52.4 [42.8–63.2]	62.3 [22.8–106.3]	163.7 [134.3–197.6]	−9.1 [−30.4 to 14.4]	40.8 [33.7–48.4]	72.0 [32.9–115.4]	128.0 [106.3–151.6]	−10.9 [−30.5 to 10.5]	1144.5 [916.2–1402.4]	46.1 [8.3–89.7]	3540.4 [2845.1–4337.9]	−14.6 [−36.1 to 9.6]
South Korea	221.1 [188.1–257.9]	228.0 [176.9–282.9]	259.2 [221.1–301.9]	23.7 [4.3–44.6]	103.4 [94.3–110.5]	114.4 [94.4–130.6]	118.8 [107.9–126.9]	−26.4 [−33.3 to −21.1]	2248.5 [2091.1–2398.4]	51.0 [39.7–62.5]	2624.1 [2441.6–2796.0]	−38.3 [−43.0 to −33.8]
Taiwan	112.6 [89.0–146.4]	248.0 [173.6–348.1]	301.3 [238.5–390.9]	57.8 [24.5–103.4]	56.7 [45.1–72.2]	175.8 [119.9–251.8]	145.3 [115.8–185.1]	11.6 [−11.2 to 42.0]	1328.4 [1048.8–1724.0]	118.0 [71.2–182.1]	3568.7 [2819.8–4606.4]	3.1 [−19.3 to 33.0]
**South-eastern Asia**												
Brunei Darussalam	1.0 [0.9–1.1]	193.8 [150.9–247.1]	308.3 [281.5–338.7]	7.1 [−7.2 to 23.9]	0.5 [0.5–0.6]	147.5 [112.0–192.1]	200.1 [181.9–220.2]	−9.8 [−22.0 to 4.0]	15.8 [14.2–17.6]	125.9 [91.8–168.9]	4575.5 [4145.1–5057.5]	−13.9 [−26.3 to 1.2]
Cambodia	18.8 [15.2–22.3]	144.9 [84.3–223.1]	150.5 [122.1–174.8]	9.1 [−14.6 to 34.4]	14.5 [11.7–16.9]	133.9 [81.5–195.8]	124.9 [102.5–143.4]	−0.2 [−20.8 to 21.1]	432.8 [347.1–517.6]	86.5 [39.9–154.8]	3230.5 [2607.0–3823.5]	−9.4 [−30.3 to 15.3]
Indonesia	310.2 [257.3–365.0]	146.8 [101.1–193.8]	138.6 [114.8–161.7]	28.5 [6.8–48.8]	228.9 [188.6–267.9]	139.8 [96.8–178.9]	111.9 [92.1–130.2]	21.6 [0.8–40.3]	6744.8 [5622.9–7962.2]	98.2 [62.0–139.6]	2824.8 [2350.0–3308.2]	8.2 [−10.8 to 26.8]
Lao PDR	6.4 [4.9–8.0]	71.6 [26.5–134.4]	133.4 [104.5–163.1]	−11.0 [−32.1 to 14.9]	5.0 [3.8–6.1]	62.6 [22.3–114.1]	114.1 [90.5–137.4]	−16.1 [−35.0 to 6.5]	155.8 [117.6–197.0]	43.7 [4.4–103.7]	2980.0 [2294.4–3703.0]	−24.4 [−43.7 to 1.2]
Malaysia	50.8 [40.8–62.1]	240.6 [171.1–321.2]	185.1 [150.0–225.4]	27.5 [1.3–56.6]	31.1 [25.0–37.8]	179.7 [121.8–244.3]	121.9 [98.6–147.4]	3.1 [−18.3 to 26.5]	847.9 [676.9–1044.2]	143.8 [92.1–200.7]	2976.3 [2385.1–3647.4]	−3.6 [−23.7 to 18.4]
Myanmar	66.6 [54.0–82.7]	60.3 [17.4–118.1]	140.0 [115.8–172.1]	−9.2 [−30.8 to 19.4]	52.5 [43.3–64.5]	56.6 [20.3–107.8]	117.1 [98.7–142.1]	−14.1 [−32.6 to 11.2]	1529.6 [1211.5–1906.9]	29.9 [−5.0 to 79.7]	3053.0 [2446.0–3772.4]	−23.7 [−42.7 to 3.8]
Philippines	118.8 [97.6–143.7]	128.8 [86.4–183.0]	139.4 [115.2–167.6]	−5.2 [−22.6 to 19.2]	81.1 [66.9–96.6]	115.8 [76.4–164.6]	103.7 [86.5–123.3]	−14.4 [−29.9 to 7.2]	2555.6 [2124.8–3046.0]	97.4 [61.6–139.1]	2816.3 [2339.9–3343.1]	−13.8 [−29.2 to 5.7]
Singapore	17.5 [14.4–21.4]	196.9 [143.5–264.3]	229.1 [189.2–280.0]	−9.6 [−25.7 to 10.6]	6.9 [6.4–7.3]	105.7 [91.6–117.1]	92.6 [84.0–98.1]	−41.5 [−45.2 to −38.2]	160.3 [150.2–169.9]	70.6 [61.0–80.9]	2081.8 [1943.0–2213.1]	−46.0 [−49.1 to −42.6]
Thailand	166.4 [126.1–216.8]	157.0 [93.9–242.5]	169.5 [129.0–219.5]	2.1 [−22.5 to 35.4]	115.4 [87.6–149.8]	136.6 [78.7–215.9]	115.5 [88.2–149.9]	−14.9 [−35.6 to 12.9]	2912.0 [2181.7–3832.9]	91.0 [42.2–157.4]	2958.6 [2239.8–3874.2]	−18.0 [−38.4 to 9.9]
Timor-Leste	1.0 [0.8–1.3]	167.1 [89.8–279.4]	120.6 [91.4–146.5]	17.6 [−11.4 to 48.8]	0.8 [0.6–1.0]	180.0 [106.1–270.0]	103.1 [78.5–124.3]	11.1 [−15.3 to 38.0]	23.9 [16.8–29.7]	108.7 [40.8–206.5]	2592.7 [1896.4–3205.7]	1.7 [−25.8 to 34.0]
Viet Nam	175.2 [139.3–213.2]	224.1 [142.8–320.3]	180.3 [144.9–216.5]	42.1 [8.2–81.8]	112.3 [90.5–134.9]	150.9 [90.2–223.3]	122.4 [99.9–144.6]	11.7 [−13.8 to 42.4]	3175.0 [2513.6–3904.9]	141.6 [77.7–217.8]	3161.7 [2519.4–3842.2]	7.7 [−19.9 to 39.9]
**Southern Asia**												
Afghanistan	26.9 [21.1–34.3]	102.6 [61.1–161.1]	168.4 [134.2–207.7]	−1.8 [−23.8 to 25.2]	21.2 [16.5–27.0]	83.5 [43.5–135.9]	153.1 [121.8–187.5]	−4.1 [−25.6 to 21.8]	795.4 [619.2–1039.0]	108.1 [64.0–178.3]	4107.0 [3186.3–5209.4]	−8.9 [−30.4 to 21.3]
Bangladesh	134.5 [102.8–171.1]	110.8 [58.8–184.5]	99.2 [76.1–126.0]	−11.4 [−31.8 to 15.6]	105.8 [79.7–135.3]	98.0 [51.2–161.5]	82.0 [61.9–104.2]	−20.7 [−38.2 to 1.5]	3092.2 [2343.4–3954.3]	59.6 [17.0–125.6]	2185.7 [1662.2–2789.9]	−25.3 [−43.1 to −1.0]
Bhutan	0.6 [0.5–0.8]	134.2 [62.7–260.3]	103.8 [80.0–128.7]	20.3 [−12.1 to 67.5]	0.5 [0.4–0.6]	122.9 [61.5–228.4]	86.2 [67.1–104.7]	9.3 [−18.0 to 48.3]	13.2 [9.9–16.9]	69.2 [12.9–179.6]	2143.7 [1612.2–2714.1]	−3.2 [−31.7 to 43.2]
India	1212.6 [1053.3–1388.7]	160.9 [119.3–205.2]	102.7 [89.2–117.4]	12.3 [−4.8 to 31.3]	929.6 [810.2–1074.6]	147.3 [109.8–190.4]	83.0 [72.3–95.8]	1.1 [−14.4 to 18.9]	27148.4 [23679.2–31253.7]	107.8 [76.5–145.5]	2193.3 [1915.3–2522.8]	−1.9 [−16.6 to 15.3]
Iran	124.4 [113.3–133.5]	203.7 [176.7–250.8]	165.1 [150.3–177.1]	23.7 [14.8–43.8]	66.7 [61.5–71.8]	150.7 [129.1–196.2]	95.6 [88.1–102.9]	−6.4 [−13.7 to 11.6]	1798.4 [1641.2–1937.6]	93.7 [72.4–125.1]	2315.9 [2112.1–2492.4]	−11.4 [−18.9 to 3.9]
Maldives	0.4 [0.3–0.4]	189.0 [124.1–319.0]	115.1 [97.8–133.6]	−5.5 [−24.6 to 28.6]	0.2 [0.2–0.2]	124.7 [76.2–212.6]	73.6 [62.3–85.5]	−29.3 [−43.0 to −6.0]	5.9 [5.0–7.0]	73.5 [31.3–153.7]	1670.4 [1419.2–1960.9]	−38.1 [−51.3 to −14.0]
Nepal	26.6 [21.2–31.5]	135.8 [74.8–203.6]	114.8 [93.0–134.4]	14.5 [−13.2 to 44.6]	21.6 [17.3–25.3]	127.5 [72.0–188.5]	99.1 [80.2–115.3]	6.9 [−18.2 to 34.2]	627.5 [497.0–749.0]	80.1 [31.0–139.1]	2550.1 [2033.0–3018.4]	−4.5 [−28.7 to 21.6]
Pakistan	244.8 [206.9–289.6]	183.3 [136.5–244.1]	187.1 [159.7–221.2]	39.0 [16.2–68.2]	179.5 [151.8–213.7]	144.9 [103.8–199.9]	153.2 [130.8–181.4]	25.7 [4.9–53.5]	6258.4 [5261.0–7389.5]	159.6 [115.7–215.2]	4266.6 [3609.1–5066.0]	27.1 [5.4–56.1]
Sri Lanka	31.0 [23.5–40.6]	173.2 [103.3–259.9]	124.0 [94.3–161.3]	26.8 [−4.9 to 66.0]	18.7 [14.3–24.2]	122.0 [65.2–190.9]	76.5 [59.1–98.8]	−4.0 [−27.9 to 24.6]	483.5 [363.6–630.1]	86.9 [37.6–147.7]	1907.5 [1436.3–2481.0]	−6.2 [−30.8 to 24.1]
**Western Asia**												
Armenia	9.5 [8.2–11.0]	52.8 [31.1–76.8]	237.4 [204.7–273.4]	9.7 [−5.8 to 26.6]	5.8 [4.9–6.8]	41.0 [19.1–64.4]	143.0 [121.0–166.0]	−3.5 [−18.3 to 12.5]	145.3 [122.1–170.2]	11.6 [−6.2 to 31.7]	3662.9 [3088.1–4296.8]	−14.5 [−28.1 to 0.6]
Azerbaijan	19.2 [16.7–22.3]	87.6 [63.1–118.5]	197.0 [172.9–228.5]	6.2 [−7.3 to 22.8]	11.9 [10.2–14.1]	71.3 [46.0–103.7]	131.6 [113.2–157.0]	0.0 [−14.7 to 18.6]	363.9 [309.1–432.8]	57.5 [34.2–86.1]	3493.7 [2998.1–4139.0]	−11.3 [−24.2 to 4.5]
Bahrain	1.8 [1.4–2.2]	412.5 [304.8–554.0]	179.4 [146.3–213.5]	−4.7 [−23.5 to 18.6]	0.8 [0.6–1.0]	221.3 [151.8–310.4]	109.8 [89.7–129.5]	−32.6 [−45.8 to −16.2]	24.6 [19.7–30.2]	214.2 [145.2–303.8]	2253.7 [1829.9–2708.6]	−36.8 [−49.9 to −20.2]
Cyprus	6.2 [5.6–6.9]	256.3 [213.4–305.0]	330.5 [298.5–365.8]	51.0 [32.7–71.1]	2.3 [2.0–2.5]	142.1 [108.0–184.7]	120.6 [107.4–134.0]	−3.0 [−16.9 to 12.6]	48.8 [43.6–54.2]	118.0 [88.5–153.1]	2628.7 [2345.7–2921.7]	−5.4 [−18.6 to 9.3]
Georgia	13.4 [11.6–15.4]	−2.0 [−16.4 to 13.8]	244.9 [211.7–282.9]	9.8 [−6.5 to 28.1]	8.3 [7.1–9.6]	2.1 [−14.1 to 20.2]	145.0 [123.4–168.8]	9.2 [−8.8 to 28.7]	213.7 [180.9–249.7]	−13.0 [−27.4 to 3.5]	4072.2 [3454.7–4753.9]	2.2 [−14.8 to 21.6]
Iraq	43.0 [33.8 to 53.4]	272.4 [186.8–389.4]	162.5 [129.2–197.2]	36.3 [5.5–72.6]	24.4 [19.1–29.8]	206.5 [136.2–296.4]	105.3 [84.0–125.6]	12.8 [−11.9 to 41.5]	780.1 [609.2–983.8]	177.6 [110.1–273.3]	2743.6 [2153.8–3383.7]	5.1 [−19.5 to 37.0]
Israel	33.5 [27.5–40.7]	166.5 [118.8–223.6]	300.7 [245.0–367.0]	14.2 [−6.7 to 39.4]	14.4 [13.3–15.2]	105.9 [95.1–115.3]	122.7 [113.2–129.1]	−17.2 [−20.7 to −13.7]	301.5 [284.3–318.8]	83.9 [75.2–93.6]	2764.3 [2617.4–2919.3]	−19.8 [−23.6 to −15.6]
Jordan	11.7 [10.1–13.7]	435.4 [345.0–551.1]	159.0 [137.7–185.4]	21.3 [0.8–46.1]	5.6 [4.8–6.6]	306.5 [234.8–403.4]	91.1 [77.6–106.3]	−8.4 [−23.9 to 12.8]	171.7 [146.5–203.3]	254.0 [192.8–337.8]	2184.9 [1870.9–2571.8]	−14.5 [−29.7 to 5.5]
Kuwait	4.4 [3.8–5.1]	307.7 [253.4–376.6]	150.1 [128.6–175.0]	16.0 [0.5–34.9]	1.6 [1.4–1.9]	230.1 [184.5–284.8]	71.0 [59.9–83.5]	−9.9 [−22.1 to 4.7]	47.5 [41.0–55.8]	161.1 [125.2–204.5]	1578.0 [1341.2–1847.0]	−20.4 [−31.6 to −7.0]
Lebanon	16.7 [14.0–20.6]	285.1 [209.5–403.3]	319.5 [268.3–394.1]	72.6 [39.3–124.7]	7.5 [6.4–9.5]	155.6 [107.4–246.9]	144.0 [122.1–183.5]	7.2 [−13.0 to 44.7]	183.8 [152.5–226.9]	119.3 [74.0–189.7]	3525.5 [2924.3–4353.2]	5.0 [−16.6 to 38.0]
Oman	3.3 [2.8–3.8]	281.9 [202.7–389.8]	161.5 [143.9–178.1]	47.8 [20.0–91.4]	1.4 [1.2–1.6]	142.1 [91.7–218.0]	89.5 [79.9–98.8]	5.9 [−14.6 to 39.6]	44.8 [37.8–52.4]	132.8 [81.3–206.9]	1989.1 [1742.3–2235.5]	−4.7 [−24.3 to 24.6]
Palestine	4.9 [4.3–5.6]	213.5 [147.2–298.6]	187.5 [162.8–213.8]	20.0 [−5.7 to 53.7]	2.9 [2.5–3.3]	160.2 [103.3–233.2]	127.2 [110.4–145.0]	1.7 [−19.8 to 30.5]	88.3 [76.6–101.4]	152.9 [99.0–226.4]	3065.6 [2656.2–3510.9]	−2.6 [−24.9 to 26.1]
Qatar	2.2 [1.7–2.9]	983.8 [705.2–1354.2]	237.0 [190.1–290.0]	45.5 [13.3–88.6]	0.8 [0.6–1.1]	517.1 [352.0–732.8]	143.9 [116.3–175.0]	4.5 [−18.1 to 34.4]	28.6 [21.9–36.6]	497.6 [337.6–708.8]	2636.7 [2081.8–3270.5]	−12.1 [−32.7 to 13.6]
Saudi Arabia	33.0 [26.1–41.6]	522.0 [370.1–730.2]	140.0 [114.9–170.5]	77.5 [39.2–130.6]	13.1 [10.4–16.3]	208.0 [128.2–324.9]	73.2 [61.1–87.8]	2.8 [−20.3 to 39.4]	460.7 [361.9–591.3]	230.6 [140.1–365.1]	1816.6 [1480.1–2241.2]	4.1 [−21.6 to 42.6]
Syria	14.8 [11.5–19.3]	116.1 [57.8–212.2]	117.0 [92.2–151.5]	17.7 [−12.3 to 63.1]	8.8 [6.7–11.5]	89.5 [36.6–169.3]	76.5 [59.9–98.7]	−3.6 [−28.7 to 34.3]	258.5 [197.5–344.1]	49.9 [5.9–118.8]	1932.1 [1493.6–2552.5]	−11.9 [−36.6 to 25.9]
Turkey	182.3 [149.3–220.8]	155.4 [103.7–223.9]	207.7 [171.2–250.2]	14.3 [−8.3 to 43.4]	104.1 [84.5–126.6]	92.7 [52.0–144.1]	120.4 [98.0–146.0]	−17.9 [−34.9 to 3.3]	2647.4 [2136.1–3261]	55.4 [21.6–100.7]	2974.5 [2409.8–3645.7]	−25.3 [−41.3 to −3.9]
United Arab Emirates	10.5 [7.5–14.2]	970.2 [674.0–1388.9]	196.4 [153.8–248.4]	10.8 [−12.9 to 43.5]	5.5 [3.9–7.4]	746.8 [511.1–1079.6]	141.0 [112.1–176.5]	−6.5 [−25.6 to 22.9]	209.8 [146.5–289.4]	729.9 [493.3–1065.6]	3263.2 [2515.5–4204.1]	−6.7 [−28.1 to 23.8]
Yemen	17.5 [13.4–22.6]	194.3 [115.2–317.2]	113.8 [89.1–144.3]	16.3 [−9.3 to 55.2]	12.7 [9.7–16.3]	179.1 [107.9–283.4]	93.0 [72.5–118.6]	8.2 [−15.4 to 43.9]	416.8 [307.7–548.0]	155.7 [79.4–280.1]	2378.0 [1812.0–3092.5]	3.9 [−23.3 to 44.4]

Note: The figures inside square bracket represent the 95% uncertainty interval. Data Source: Global Burden of Disease, Injuries and Risk Factors 2019 Study.

Between 1990 and 2019, incident cases doubled or more (percent change >100%) in 38/49 countries, deaths doubled or more in 32/49 countries and DALYs doubled or more in 21/49 countries (Table 1; See Supplementary Figures S1–S3 for temporal patterns of absolute counts in 49 countries, Appendix pp 54–56).

Country-wise decomposition analysis of changes in incident cases between 1990 and 2019 yielded interesting observations (Supplementary Table S13, Appendix pp 45–47). For several countries in Asia, large proportion of incidence increase came from population growth and aging, whereas increase was negated by changes in age-specific rates for a few countries. In case of Afghanistan, population growth led to incidence increase by 235.2% between 1990 and 2019, which was partially offset by the reduction due to population aging and age-specific rates. Among countries posting highest increases (e.g., Qatar and UAE), greatest contribution pertained to population growth with rest of the incidence change was due to population aging and changes in age-specific rates. In case of Armenia, the reduction in incident cases due to negative population growth were more than offset by population aging and changes in age-specific rates. In case of Japan with negligible population growth between 1990 and 2019, the major increase came from population aging.

Against rises in absolute counts, the age-standardized rates either stagnated or decreased in most countries, with countries such as South Korea and Singapore posting the maximum reduction in age-standardized mortality and DALYs rate between 1990 and 2019 (Supplementary Figures S4–S6 for temporal patterns of age-standardized rates of cancers in 49 Asian countries, Appendix pp 57–59).

Supplementary Figures S7–S9 display rank of 29 cancers in 49 countries in 2019 as per incident cases, deaths and DALYs, respectively, for both sexes combined (Appendix pp 60–62). In most countries, TBL, breast, CRC, stomach and other malignant neoplasms were among the top-5 ranked cancers in terms of incident cases (Appendix p 60). In terms of deaths and DALYs, leukemia, liver and pancreatic cancer also featured among top-5 cancers in some countries. For instance, in terms of death count, liver cancer was ranked as first in Thailand and Mongolia and leukemia was ranked as first in case of Syria and third in case of Yemen, Iraq and Afghanistan in 2019.

Among males, TBL, CRC, prostate and stomach cancer were among the top-5 most frequent cancers in most countries, and among females, breast, CRC, cervical and TBL cancer were the most frequent cancers in most countries in 2019 (Appendix pp 63–64).

### Age-wise distribution of cancer burden

**Fig. 3:**
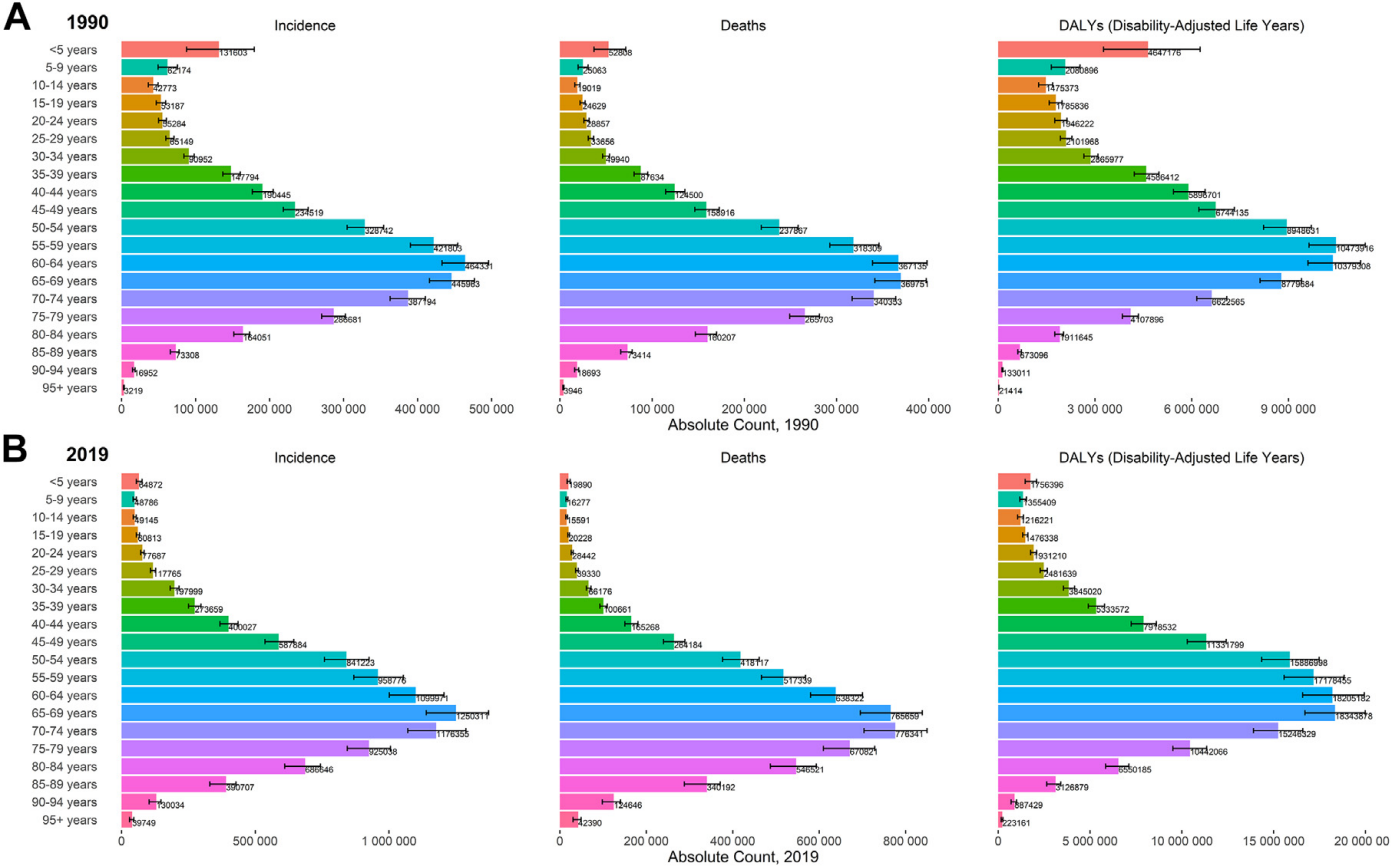
**Agewise Burden of Cancer in Asia A) 1990 B) 2019**. Incidence: Age-specific new cases; Deaths: Age-specific death counts; DALYs: Disability-adjusted-life-years. Data Source: Global Burden of Disease, Injuries and Risk Factors 2019 Study.

Fig. 3 displays the cancer burden by absolute count in 20 age groups for both sexes combined in Asia in 1990 and 2019. In 1990, there were two peaks: one below the age of 5 years and second among those between the ages of 55 and 69 years in terms of different metrics (See Supplementary Figures S12 and S13 for agewise burden among males and females, respectively, Appendix pp 65–66). Among children aged less than 5 years, the cancer burden decreased substantially between 1990 and 2019 (Fig. 3). Among other child and adolescent age groups (<19 years), cancer incidence either decreased or registered minimal increases between 1990 and 2019, whereas deaths and DALYs mostly decreased. Cancer burden increased most significantly in age groups from 45 to 84 years. Between 1990 and 2019, age-specific deaths grew 5-times, 7-times, and 11-times among 85–89, 90–94 and 95+ age groups, respectively.

Supplementary Figures S14–S16 (Appendix pp 67–69) display the proportion of incident cases of 29 cancers in different age groups. Cancer-wise, leukemia, brain and central nervous system cancer, lymphoma (Hodgkin and Non-Hodgkin), and testicular cancer accounted for a large proportion of cases in those below the age of 50 years; in contrast, cancers such as breast, cervical, ovarian, and prostate cancer had dominant cancer burden among those older than 50 years (Appendix pp 67–69).

### Relationship between age-standardized rates and SDI

**Fig. 4:**
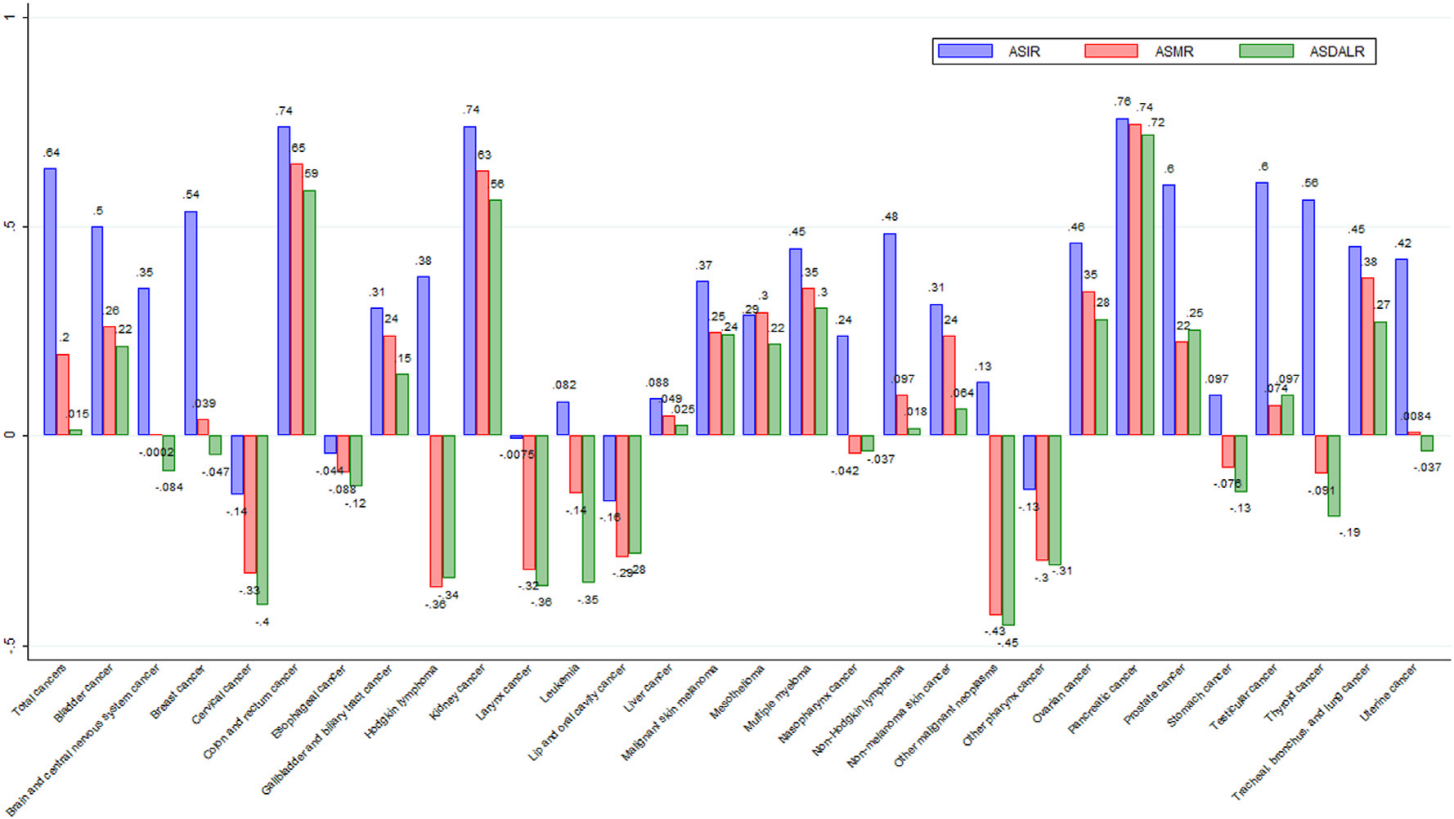
**Pairwise Correlation of Country-level age-standardized rates of 29 Cancers and Country-level Socio-demographic Index, 1990–2019**. Pairwise correlation were calculated based on SDI values of countries from 1990 to 2019 and corresponding country-level age-standardized rates of each cancer between 1990 and 2019. The numbers indicate pairwise correlation coefficients.

Fig. 4 shows the bivariate correlation between country-level age-standardized rates and SDI between 1990 and 2019 for each of the 29 cancers. SDI exhibited a positive correlation with country-level ASIR for all cancers except cervical (r=−0.14), esophageal (r=−0.044), larynx (*r* = −0.0075), lip and oral cavity (r=−0.16), and other pharynx cancer (r=−0.13). In case of these five cancers, increasing SDI is associated with lower cancer burden in terms of all three metrics. For CRC, SDI exhibited a strong positive correlation with ASIR (r=0.74), ASMR (r=0.65) and ASDALR (r=0.59), showing that the CRC burden increases with an increase in SDI. For few cancers such as Hodgkin Lymphoma, ASIR had a positive correlation with SDI, whereas ASMR and ASDALR had a negative correlation with SDI, implying that the incidence rate might increase with increasing SDI but the mortality and DALYs rates decrease with development (i.e., increasing SDI).

### Risk factors analysis

For both sexes combined, smoking was the top risk factor causing 33.1 million [28.8–37.8 million] DALYs due to cancers, followed by alcohol use (7.3 million [6.2–8.5 million]) and ambient particulate matter (PM) pollution (5.3 million [3.9–7.0 million]) in 2019 (Table 2). Risk attributable DALYs due to ambient PM pollution for both sexes combined registered the maximum increase (355.2% [200.4%–648.7%]) between 1990 and 2019. Between 1990 and 2019, risk-attributable cancer DALYs decreased the most in case of diet low in vegetables (−50.8% [−60.6% to 2.2%]) and household air pollution from solid fuels (−30.5% [−56.2% to 4.0%]). Among males, smoking (29.8 million [25.6–34.5 million]), alcohol use (6.5 million [5.4–7.6 million]) and ambient PM pollution (3.8 million [2.7 million–5.1 million]) were the leading risk factors, whereas unsafe sex (4.7 million [3.8–5.4 million]) caused the maximum DALYs due to cancers among females, followed by smoking (3.3 million [2.8–3.8 million]) and high body-mass index (2.3 million [1.2–3.8 million]). In terms of deaths too, smoking (1.4 million [1.3–1.6 million]), alcohol use (268,900 [227,800–312,700]) and ambient PM pollution (234,500 [170,900–302,800]) were the leading risk factors in 2019 for both sexes combined (Appendix pp 48–50). These were again the leading risk factors in terms of age-standardized DALYs rate in 2019 (Appendix pp 51–53).

**Table 2:** Cancer DALYs attributable to Risk Factors, 1990–2019.

Risk Factors	Dalys (in thousands), both sexes			Dalys (in thousands), males			Dalys (in thousands), females		
	1990	2019	Percent change, 1990–2019	1990	2019	Percent change, 1990–2019	1990	2019	Percent change, 1990–2019
**Behavioral Risks**									
Alcohol use	3799.2 [3190.8–4479.1]	7329.7 [6214.5–8513.4]	92.9 [59.6–134.5]	3255.6 [2658.7–3912.1]	6468.5 [5392.9–7597.5]	98.7 [60.2–146.4]	543.6 [452.6–641.5]	861.2 [723.9–1009.1]	58.4 [33.5–89.5]
Chewing tobacco	659.0 [522.1–809.1]	1412.5 [1089.8–1770.7]	114.3 [74.0–161.2]	417.4 [288.2–559.1]	820.5 [555.6–1114.2]	96.6 [46.3–157.4]	241.6 [188.2–305.7]	592.0 [444.4–761.3]	145.0 [95.0–200.1]
Diet high in processed meat	82.1 [30.0–122.7]	223.7 [70.0–352.6]	172.3 [103.8–225.9]	44.0 [16.1–66.1]	129.6 [39.2–207.1]	194.8 [112.8–265.5]	38.2 [13.9–57.7]	94.1 [30.6–149.2]	146.4 [91.4–192.6]
Diet high in red meat	246.2 [102.7–448.2]	866.6 [422.5–1422.6]	251.9 [188.4–405.0]	82.7 [16.2–189.2]	380.9 [97.1–730.4]	360.7 [230.8–791.8]	163.5 [72.0–266.4]	485.7 [256.7–738.8]	197.0 [143.6–301.9]
Diet high in sodium	1129.4 [27.5–4285.9]	1320.8 [33.1–5076.2]	16.9 [−2.1 to 35.4]	726.7 [16.9–2707.6]	907.9 [21.3–3406.2]	24.9 [0.3–52.7]	402.7 [10.4–1544.0]	412.9 [11.2–1634.5]	2.5 [−20.1 to 23.4]
Diet low in calcium	957.9 [759.1–1215.1]	2215.3 [1615.5–2939.3]	131.3 [96.3–166.1]	529.0 [414.2–682.1]	1352.8 [983.9–1803.8]	155.7 [102.6–208.8]	429.0 [328.5–550.3]	862.5 [622.4–1144.8]	101.1 [69.3–136.2]
Diet low in fiber	152.4 [64.2–266.4]	275.0 [114.8–508.6]	80.4 [52.4–106.4]	80.1 [34.2–138.8]	157.2 [65.5–284.3]	96.3 [53.4–135.3]	72.3 [30.5–126.6]	117.7 [48.9–218.2]	62.8 [36.6–89.8]
Diet low in fruits	1719.7 [935.5–2611.5]	2016.6 [1058.0–3200.9]	17.3 [−17.0 to 56.3]	1188.5 [648.4–1802.3]	1430.9 [717.7–2318.8]	20.4 [−17.1 to 67.0]	531.2 [288.6–813.9]	585.6 [323.2–873.8]	10.2 [−20.9 to 51.3]
Diet low in milk	917.2 [630.2–1200.6]	2499.8 [1676.4–3272.5]	172.5 [140.3–204.7]	492.3 [334.0–649.9]	1490.4 [999.5–2001.3]	202.8 [148.9–255.3]	424.9 [290.8–564.4]	1009.4 [662.5–1332.5]	137.5 [106–172.4]
Diet low in vegetables	404.0 [34.7–849.2]	198.7 [33.2–383.4]	−50.8 [−60.6 to 2.2]	274.2 [22.3–581.1]	127.2 [23.2–248.7]	−53.6 [−63.7 to 8.3]	129.8 [12.8–270.8]	71.5 [11.0–141.3]	−44.9 [−57.5 to −0.2]
Diet low in whole grains	736.7 [282.1–979.8]	2028.5 [771.5–2706.8]	175.3 [142.5–207.9]	401.3 [152.4–541.5]	1229.4 [473.5–1669.7]	206.3 [153.3–257.1]	335.4 [128.2–446.1]	799.2 [305.0–1078.7]	138.2 [105.1–173.3]
Drug use	842.9 [628.6–1097.5]	1065.2 [835.7–1327.2]	26.4 [5.0–55.4]	503.8 [397.9–627.0]	620.6 [484.2–768.2]	23.2 [−1.7 to 54.7]	339.1 [212.2–495.8]	444.6 [319.8–593.8]	31.1 [−3.8 to 82.2]
Low physical activity	188.3 [71.0–355.8]	534.8 [198.6–989.5]	184.0 [148.4–223.4]	70.3 [15.6–148.2]	227.8 [53.7–458.8]	223.8 [167.7–293.1]	118.0 [54.3–208.2]	307.1 [142.9–533.5]	160.3 [126.6–201.5]
Secondhand smoke	977.7 [623.3–1396.5]	2227.7 [1430.0–3208.0]	127.9 [92.8–165.4]	429.0 [253.3–660.7]	998.9 [571.3–1535.0]	132.8 [77.2–207.6]	548.7 [347.3–781.7]	1228.8 [785.2–1787.7]	124.0 [89.0–167.1]
Smoking	**17630.9[15556.7–19747.4]**	**33064.3[28758.7–37755.8]**	**87.5[57.1–123.8]**	**15954.1[13889.0–18069.8]**	**29796.0[25605.3–34487.5]**	**86.8[55.0–128.1]**	**1676.8[1423.9–1946.3]**	**3268.3[2788.2–3799.2]**	**94.9[64.5–132.3]**
Unsafe sex	3013.6 [2521.2–3998.7]	4693.9 [3779.6–5446.2]	55.8 [17.6–96.0]	–	–	–	3013.6 [2521.2–3998.7]	4693.9 [3779.6–5446.2]	55.8 [17.6–96.0]
**Environmental/occupational risk**									
Ambient particulate matter pollution	1176.4 [652.1–1816.1]	5354.8 [3888.0–6959.5]	355.2 [200.4–648.7]	897.2 [490.3–1409.8]	3825.0 [2679.7–5075.1]	326.3 [170.6–628.6]	279.2 [153.9–450.3]	1529.8 [1090.2–2023.8]	447.8 [247.8–800.3]
Household air pollution from solid fuels	2285.4 [1501.4–3191.2]	1589.5 [871.4–2560.8]	−30.5 [−56.2 to 4.0]	1592.4 [1033.4–2290.1]	1024.3 [525.1–1683.9]	−35.7 [−61.0 to 0.1]	693.0 [467.0–963.5]	565.2 [307.7–900.6]	−18.4 [−48.6 to 21.2]
Occupational exposure to arsenic	73.9 [22.4–124.0]	169.9 [57.0–287.4]	129.9 [91.3–184.2]	53.6 [15.4–89.9]	116.8 [38.6–202.1]	117.8 [67.0–179.3]	20.3 [6.1–35.2]	53.1 [18.1–90.7]	161.7 [113.1–238.7]
Occupational exposure to asbestos	434.3 [307.9–595.0]	1218.4 [865.6–1610.2]	180.5 [128.7–237.0]	335.1 [218.7–488.2]	996.7 [654.8–1393.6]	197.5 [134.6–259.3]	99.2 [60.0–167.9]	221.6 [138.3–300.8]	123.3 [31.6–246.4]
Occupational exposure to benzene	35.5 [10.6–59.3]	50.6 [14.4–83.8]	42.3 [21.9–70.3]	21.2 [6.4–35.5]	30.6 [8.6–51.1]	44.4 [21.8–79.9]	14.4 [4.1–24.4]	20.0 [5.7–33.9]	39.2 [8.6–76.4]
Occupational exposure to beryllium	3.0 [2.4–3.6]	6.8 [5.4–8.4]	128.7 [86.5–173.5]	2.2 [1.6–2.8]	4.6 [3.4–6.0]	113.2 [61.9–171.4]	0.8 [0.6–1.0]	2.2 [1.6–2.8]	170.4 [112.9–242.2]
Occupational exposure to cadmium	6.6 [5.4–8.0]	15.9 [12.8–19.1]	141.8 [96.6–191.9]	4.8 [3.7–6.1]	10.9 [8.2–14.0]	128.5 [75.1–193.6]	1.8 [1.4–2.3]	5.0 [3.7–6.5]	176.7 [119.7–252.7]
Occupational exposure to chromium	12.7 [10.9–14.8]	32.7 [27.5–38.8]	157.2 [110.1–209.2]	9.3 [7.6–11.3]	22.7 [17.8–28.5]	143.6 [85.4–211.8]	3.4 [2.7–4.1]	10.0 [7.8–12.6]	194.5 [134.8–272.6]
Occupational exposure to diesel engine exhaust	155.7 [129.8–185.7]	419.5 [354.3–498.6]	169.4 [118.9–222.6]	122.6 [97.2–151.2]	317.0 [253.7–391.7]	158.5 [102.1–225.7]	33.1 [26.2–40.8]	102.4 [80.4–127.7]	209.5 [148.9–286.9]
Occupational exposure to formaldehyde	30.6 [23.6–38.3]	39.1 [30.9–48.2]	27.8 [9.1–50.9]	19.6 [13.9–26.2]	27.3 [19.8–36.5]	39.6 [15.5–70.6]	11.0 [7.8–15.0]	11.7 [8.8–15.2]	6.8 [−16.2 to 39.5]
Occupational exposure to nickel	80.5 [12.6–183.6]	175.0 [29.3–401.2]	117.3 [76.7–169.9]	59.9 [9.3–140.7]	124.2 [21.2–287.8]	107.4 [57.9–173.5]	20.6 [3.6–48.0]	50.8 [9.1–116.8]	146.2 [96.2–219.1]
Occupational exposure to polycyclic aromatic hydrocarbons	44.5 [36.6–53.5]	114.1 [91.7–137.5]	156.1 [109.4–208.9]	32.8 [24.9–41.4]	79.5 [59.8–101.8]	142.7 [86.8–208.0]	11.8 [9.1–14.5]	34.6 [26.4–44.3]	193.5 [132.7–274.9]
Occupational exposure to silica	432.7 [202.0–680.3]	943.0 [421.3–1516.4]	117.9 [80.6–159.4]	332.4 [152.4–531.2]	715.5 [322.2–1124.8]	115.3 [69.4–167.8]	100.3 [45.3–156.7]	227.4 [94.0–365]	126.9 [81.4–184.6]
Occupational exposure to sulfuric acid	50.6 [21.0–92.0]	86.5 [36.1–160.8]	70.8 [46.4–99.4]	44.8 [18.6–81.3]	77.3 [32.0–144.5]	72.6 [45.9–105.2]	5.9 [2.4–10.5]	9.2 [3.8–17.0]	57.0 [34.4–87.8]
Occupational exposure to trichloroethylene	0.3 [0.1–0.6]	1.4 [0.3–2.5]	343.2 [266.9–442.4]	0.2 [0.0–0.4]	1.0 [0.2–1.9]	382.1 [274.2–523.8]	0.1 [0.0–0.2]	0.4 [0.1–0.7]	260.0 [195.1–339.9]
Residential radon	411.7 [79.0–808.6]	960.8 [187.6–1929.5]	133.4 [91.2–178.8]	299.3 [56.4–594.3]	672.0 [129.6–1362.1]	124.5 [73.8–183.1]	112.4 [21.8–221.9]	288.8 [54.4–579.5]	157.0 [113.2–211.4]
**Metabolic risks**									
High body-mass index	1530.7 [480.9–3324.1]	5163.0 [2559.2–8662.5]	237.3 [145.3–469.2]	870.3 [226.4–2010.8]	2899.2 [1267.3–5310.6]	233.1 [131.8–504.8]	660.4 [227.4–1430.6]	2263.8 [1156.2–3777.2]	242.8 [149.3–456.4]
High fasting plasma glucose	1176.2 [298.6–2530.4]	4185.4 [1087.4–8747.9]	255.8 [218.4–304.2]	655.8 [145.8–1478.0]	2301.0 [533.0–5127.1]	250.8 [193.7–324.2]	520.4 [134.1–1120.1]	1884.4 [495.8–4062.9]	262.1 [218.5–321.1]

Note: The figures inside square bracket represent the 95% uncertainty interval. Data Source: Global Burden of Disease, Injuries and Risk Factors 2019 Study. Bold denotes the maximum burden.

Notably, percent changes in terms of age-standardized DALYs rates were smaller than absolute changes between 1990 and 2019 (Table 2; Supplementary Table S15, Appendix pp 51–53). Among the leading risk factors in 1990, the age-standardized DALYs rate of ambient PM pollution increased the most between 1990 (54.5/100,000 [30.4–84.1]) and 2019 (109.0/100,000 [79.3–141.4]) such that its rank increased from 8th in 1990 to 3rd in 2019 (Appendix p 70). In contrast, the age-standardized DALYs rate of cancers due to household air pollution from solid fuels decreased substantially between 1990 (104.2/100,000 [68.5–145.0]) and 2019 (32.0/100,000 [17.5–51.6]) such that its rank decreased from 4th to 12th (Appendix p 70). Other risk factors such as high body-mass index and high fasting plasma glucose also witnessed substantial increases in age-standardized DALYs rate between 1990 and 2019 (Appendix pp 51–53; Appendix p 70). Few dietary risk factors such as diet low in milk and whole grains entered into top-10 risk factors, whereas diet high in sodium and diet low in fruits were no longer ranked among top-10 in 2019 (Appendix p 70). Similarly, as per ranking according to age-standardized mortality rate too, smoking, alcohol use and ambient PM pollution were the leading risk factors in 2019 (Appendix p 71).

In order to highlight which risk factors are more dominant at the country-level, we also present the rank of 34 risk factors as per age-standardized DALYs rate in 2019 (Supplementary Figures S19–S21, Appendix 72–74). At country-level too, smoking, alcohol use, unsafe sex, high body-mass index, ambient PM pollution and high fasting plasma glucose were among top-5 risk factors in majority of countries (Appendix pp 72–74).

## Discussion

With 9.4 million new cases and 5.6 million deaths in 2019, cancer has become a more significant public health threat in Asia. China, India, and Japan are the three leading countries in Asia in terms of number of new cases, deaths and DALYs. The ASIRs for majority of cancers were positively correlated with SDI. In Asia overall and in individual countries as well, TBL, breast, CRC, stomach and non-melanoma skin cancer were mostly among top-5 most frequent cancers in 2019 with few countries having leukemia, prostate, liver and pancreatic cancer among top-5 cancers. Testicular, kidney, prostate, and CRC posted the most significant increases in incidence rate between 1990 and 2019, whereas stomach, liver, esophageal and leukemia posted the most significant reduction in age-standardized mortality and DALYs rate between 1990 and 2019. Among the 34 risk factors, smoking, alcohol consumption, and ambient PM pollution remained the dominant risk factors and DALYs burden due to ambient PM pollution, high body-mass index and high fasting plasma glucose increased most notably between 1990 and 2019.

The burden of TBL is notably high in Asia with smoking being identified as the primary risk factor. Tobacco control, therefore, must be identified as a major policy target in Asia as it is the single biggest risk factor of several neoplasia prevalent in Asia.^4^^,^^21^ The burden of TBL cancer is also high among females in Asia, which traditionally have much lower smoking rates than females in the West, which can be partly explained by rising levels of ambient PM pollution and high, although decreasing, prevalence of household air pollution. Interestingly, cancer burden due to ambient PM pollution and household air pollution from burning solid fuels showed divergent trends in Asia. On the one hand, cancer DALYs and deaths due to household air pollution from burning solid fuels decreased substantially between 1990 and 2019; on the other hand, the cancer burden attributable to ambient PM pollution have increased most significantly among 34 risk factors in the last three decades. The rising cancer burden due to increasing ambient air pollution is concerning in Asia. As per State of Global Air Report,^24^ five out of the top 10 countries in terms of population-weighted annual average of PM_2.5_ in 2019 are present in Asia [India (1st), Nepal (2nd), Qatar (4th), Bangladesh (9th) and Pakistan (10th)]. The primary reasons for increasing air pollution in Asia are industry-led economic growth along with urbanisation, rural-to-urban migration and increasing usage of motor vehicles.

Apart from tobacco smoking and ambient air pollution, high prevalence of smokeless tobacco such as khaini, gutkha, betel quid and paan masala is a public health concern in South Asian countries such as India, Bangladesh, and Nepal.^25^ As per our study results, India alone accounted for 32.9% of global deaths and 28.1% of new cases of lip and oral cavity cancer in 2019. As per the global adult tobacco survey (GATS), 199.4 million adults in India consumed smokeless tobacco.^26^ Notably, more than 50% of the oral cancer burden has been attributed to smokeless tobacco (SMT),^27^ whose prevalence has grown in recent times in South Asia, including India.^27^^,^^28^ SMT not only increases the risk of oral cancers but also enhances the risk of esophageal and pancreatic cancer.^27^ Amidst the high burden of SMT-induced cancers, the SMT control policies such as taxes and regulatory mechanisms seem to be either inadequate or poorly implemented in South Asia, resulting in the unabated burden of SMT-induced diseases, including oral cancers.^29^

In Asia, breast cancer results in more than 300,000 deaths and more than 900,000 cases a year in 2019 and is ranked first in 46/49 Asian countries in terms of incident cases in females in 2019. Randomized control trials in developed countries have demonstrated the success of screening programs in detecting pre-cancerous and early cancer lesions.^30^^,^^31^^,^^32^ Several deaths due to breast cancer can be averted by early screening using breast mammograms; it, however, has also been found to be susceptible to high false-positive rates and overdiagnosis/overtreatment in HICs.^32^ Moreover, as mammographic screening might not be cost-effective in several low-resource settings in Asia, cost-effective approaches such as clinical breast examination and cancer awareness can lead to clinical downstaging of breast cancer and can potentially save lives.^33^^,^^34^^,^^35^^,^^36^

Among top cancers, burden of gastrointestinal cancers (GI) stayed notably high in most Asian countries. A previous study has also noted that Asia accounts for the largest proportion of the GI cancer burden worldwide.^37^ For majority of GI cancers, smoking, alcohol consumption, obesity, sedentary lifestyle, and infections remain the biggest risk factors.^37^ While incidence rate of esophagus, liver and stomach cancer has decreased between 1990 and 2019, the absolute count remains high, indicating that more efforts are required to reduce the burden of these cancers. Majority of liver cancer in Asia is hepatocellular carcinoma arising due to hepatitis B virus (HBV) and hepatitis C virus (HCV) infections.^38^ It has also been observed that liver cancer due to HBV and HCV might be decreasing worldwide, whereas liver cancer due to other etiologies such as alcohol use and NASH might be increasing.^39^ Particularly, liver cancer due to NASH is mostly linked with behavioral lifestyle factors such as obesity.^40^ Therefore, policies directed towards lifestyle interventions focussing on curtailing the obesity epidemic and promoting healthy lifestyles are pertinent in curbing the rising cancer burden of these cancers.

Several Asian countries have undergone tremendous economic growth in the last few decades. Economic growth has led to tremendous investment by governments like China in projects to improve access to clean drinking water, sanitation facilities, wastewater treatment, rural water supply and sanitation, and boosting general awareness and education. Recently, India launched a campaign named “Swachch Bharat Abhiyan” meaning “Clean India Mission” which seeks to build new toilets and to discourage people from open defecation. Improved water and sanitation can help reduce the transmission of Helicobacter pylori (*H. pylori*) and, in turn, potentially lower the risk of stomach cancer. Previous research has also found that due to improved access to clean drinking water and sanitation, significant reduction in the prevalence of *H. pylori* has occurred, which has been referred to as birth cohort effect.^41^ This reduction in *H. pylori* prevalence has been quoted as one of the biggest reasons for reduction in stomach cancer burden in the last three decades.^42^

Economic growth, however, has also resulted in changing lifestyles that has led to increase in burden of cancers such as CRC across Asian countries. Current study has also found that CRC age-standardized rates are positively linked with a country's development status. Importantly, high proportion of disease burden can be attributed to modifiable risk factors such as smoking, alcohol use, dietary habits (diet low in calcium, fibre, milk and high in red/processed meat), high body-mass index and sedentary lifestyles.^43^^,^^44^^,^^45^^,^^46^ Of note, CRC survival rates depend crucially on the stage of diagnosis and availability of therapy post-diagnosis; therefore, early detection of adenomas or adenomatous polyps through screening devices such as colonoscopy or stool-based methods holds the key in CRC management and control.^47^

However, mere availability of screening might not improve the survival rates if cancer treatments are either unavailable or unaffordable. In LMICs of Asia, oncologic infrastructure is either scarce or is not affordable, particularly in rural areas; this along with weak referral system results in a patient getting delayed diagnosis and treatment; hence lower survival rates ensue.^6^ Sullivan et al.^48^ found that in LMICs, including those in Asia, only a small percentage of patients with cancer have access to safe, timely, and affordable surgery. Moreover, critical adjunct treatment modalities accompanying surgery, such as pathology and imaging are also inadequate in LMICs.^48^ Furthermore, factors such as high treatment expenses and distance to oncologic care in LMICs results in therapy abandonment in case of several childhood cancers^49^^,^^50^^,^^51^; therefore, along with timely availability of cancer screening and treatment, its cost-effectiveness or coverage of treatment expenses must also be a policy priority.

As countries undergo development, a commonly observed pattern is the reduction in cancer burden in younger age groups and the increase in cancer burden associated with increasing life expectancy. Accordingly, we found a substantial reduction in cancer burden among those under the age of 5 years and burden of cancers such as leukemia which causes a substantial burden in younger age groups, decreased between 1990 and 2019. Simultaneously, the burden of cancers associated with longer lifespan such as prostate, pancreatic and breast cancer increased substantially between 1990 and 2019. Among females, cervical cancer is ranked second or among top-5 cancers in several Asian countries. The human papillomavirus (HPV) vaccine, introduced in 2006, has been proven to be effective in the prevention of cervical cancer^52^ and has led to the reduction of HPV-related deaths in HICs.^53^^,^^54^^,^^55^ Data from a meta-analysis of 4 studies (1 randomized controlled trial and 3 cross-sectional studies) and 13,285 participants showed 80% (risk ratio, 0.20; 95% confidence interval, 0.09–0.43) less likelihood of oral HPV16 infection.^56^ The authors state that because HPV-related oropharyngeal cancers have no obvious premalignant lesion precursor, prevention through HPV vaccination is of paramount importance.^56^ Therefore, vaccination against HPV and screening through cost-effective methods such as visual examination using acetic acid must be adopted and popularized in LMICs.^57^^,^^58^

The present study utilized estimates from the GBD study, the accuracy of which crucially depends upon quality of data from cancer registries. However, some low-income and conflict-ridden countries had low availability and quality of data from population-based cancer registries (PBCRs). Moreover, even if PBCR is present, cancer might be misdiagnosed or is not ascertained due to a lack of diagnostic facilities, which might lead to the wrong encoding of data. For instance, Lam et al.^59^ has estimated incidence deficit in case of childhood cancers such as leukemia and brain tumours where the symptoms are less obvious and advanced diagnostics are required. There can be issues such as several deaths being reflected as deaths from liver cancer, whereas cancer might have metastasized from some other primary site to the liver. Some recent studies, even in HICs, have also pointed toward this issue.^60^^,^^61^ Therefore, due to lack of quality data from PBCRs, under-reporting, and under-ascertainment of cases and cancer deaths, the estimates reported and examined in this paper are expected to be biased. GBD tries to overcome these limitations by utilizing data from other sources such as vital registration, verbal autopsy, and predictive covariates and data from hospital-based registries or neighbouring countries. Yet, we don't claim that GBD estimates can completely replace the ongoing efforts of boosting diagnostic facilities and data collection through PBCRs. Combating the cancer burden in the future requires a correct assessment of the cancer burden in present; therefore, quality cancer registration and boosting diagnostic facilities must be considered as the most instrumental component of any cancer control strategy, providing the data to inform effective cancer policy in Asia. Till the time quality data from PBCRs is made available for number of years, the assessment of time trends of GBD estimates can provide crucial information for the policymakers.

In conclusion, cancer incidence is ubiquitously increasing in Asia with mortality rates stagnating or decreasing for a few cancers in the last three decades. Among the modifiable risk factors, smoking, alcohol consumption, and ambient PM pollution remain the dominant risk factors and the cancer burden due to ambient PM pollution, high body-mass index and high fasting plasma glucose has increased most notably between 1990 and 2019. Therefore, tackling the increasing cancer burden in Asia requires effective primary and secondary prevention strategies along with access to timely and cost-effective screening, diagnostic, and therapeutic services.

## GBD 2019 Asia All Cancers Collaborators

Rajesh Sharma, Hedayat Abbastabar, Deldar Morad Abdulah, Hassan Abidi, Hassan Abolhassani, Zahra Abrehdari-Tafreshi, Abdorrahim Absalan, Hiwa Abubaker Ali, Eman Abu-Gharbieh, Juan Manuel Acuna, Nasrin Adib, Qorinah Estiningtyas Sakilah Adnani, Abbas Aghaei, Aqeel Ahmad, Sajjad Ahmad, Ali Ahmadi, Sepideh Ahmadi, Luai A Ahmed, Marjan Ajami, Hanadi Al Hamad, Syed Mahfuz Al Hasan, Fahad Mashhour Alanezi, Adel Ali Saeed Al-Gheethi, Mohammed Khaled Al-Hanawi, Abid Ali, Beriwan Abdulqadir Ali, Yousef Alimohamadi, Syed Mohamed Aljunid, Sadeq Ali Ali Al-Maweri, Saleh A Alqahatni, Mohammad AlQudah, Rajaa M Al-Raddadi, Ala'a B. Al-Tammemi, Alireza Ansari-Moghaddam, Sumadi Lukman Anwar, Razique Anwer, Muhammad Aqeel, Jalal Arabloo, Morteza Arab-Zozani, Hany Ariffin, Al Artaman, Judie Arulappan, Tahira Ashraf, Elaheh Askari, Mohammad Athar, Maha Moh'd Wahbi Atout, Sina Azadnajafabad, Muhammad Badar, Ashish D Badiye, Nayereh Baghcheghi, Sara Bagherieh, Ruhai Bai, Khuloud Bajbouj, Shrikala Baliga, Mainak Bardhan, Azadeh Bashiri, Pritish Baskaran, Saurav Basu, Uzma Iqbal Belgaumi, Amiel Nazer C Bermudez, Bharti Bhandari, Nikha Bhardwaj, Ajay Nagesh Bhat, Saeid Bitaraf, Archith Boloor, Milad Bonakdar Hashemi, Zahid A Butt, Joshua Chadwick, Jeffrey Shi Kai Chan, Vijay Kumar Chattu, Pankaj Chaturvedi, William C S Cho, Aso Mohammad Darwesh, Nihar Ranjan Dash, Amin Dehghan, Arkadeep Dhali, Mostafa Dianatinasab, Mahmoud Dibas, Abhinav Dixit, Shilpi Gupta Dixit, Fariba Dorostkar, Haneil Larson Dsouza, Iffat Elbarazi, Noha Mousaad Elemam, Waseem El-Huneidi, Eyad Elkord, Omar Abdelsadek Abdou Elmeligy, Mohammad Hassan Emamian, Ryenchindorj Erkhembayar, Rana Ezzeddini, Zehra Fadoo, Razana Faiz, Ildar Ravisovich Fakhradiyev, Aida Fallahzadeh, MoezAlIslam Ezzat Mahmoud Faris, Hossein Farrokhpour, Ali Fatehizadeh, Hamed Fattahi, Ginenus Fekadu, Takeshi Fukumoto, Abhay Motiramji Gaidhane, Nasrin Galehdar, Priyanka Garg, Fataneh Ghadirian, Mansour Ghafourifard, MohammadReza Ghasemi, Mohammad Ghasemi Nour, Fariba Ghassemi, Maryam Gholamalizadeh, Asadollah Gholamian, Elena Ghotbi, Mahaveer Golechha, Pouya Goleij, Sahil Goyal, Mohammed Ibrahim Mohialdeen Gubari, D Sanjeeva Gunasekera, Damitha Asanga Gunawardane, Sapna Gupta, Parham Habibzadeh, Helia Sadat Haeri Boroojeni, Esam S Halboub, Randah R Hamadeh, Rifat Hamoudi, Mehdi Harorani, Mohammad Hasanian, Treska S Hassan, Simon I Hay, Mohammad Heidari, Mahsa Heidari-Foroozan, Kamran Hessami, Kamal Hezam, Yuta Hiraike, Ramesh Holla, Mohammad Hoseini, Md Mahbub Hossain, Sahadat Hossain, Vivian Chia-rong Hsieh, Junjie Huang, Nawfal R Hussein, Bing-Fang Hwang, Farideh Iravanpour, Nahlah Elkudssiah Ismail, Masao Iwagami, Linda Merin J, Farhad Jadidi-Niaragh, Morteza Jafarinia, Mohammad Ali Jahani, Haitham Jahrami, Abhishek Jaiswal, Mihajlo Jakovljevic, Mahsa Jalili, Elham Jamshidi, Umesh Jayarajah, Shubha Jayaram, Sweety Suman Jha, Mohammad Jokar, Nitin Joseph, Ali Kabir, Md. Awal Kabir, Dler Hussein Kadir, Pradnya Vishal Kakodkar, Laleh R Kalankesh, Leila R Kalankesh, Rohollah Kalhor, Feroze Kaliyadan, Vineet Kumar Kamal, Zul Kamal, Ashwin Kamath, Sitanshu Sekhar Kar, Hanie Karimi, Navjot Kaur, Leila Keikavoosi-Arani, Mohammad Keykhaei, Yousef Saleh Khader, Himanshu Khajuria, Ejaz Ahmad Khan, M Nuruzzaman Khan, Maseer Khan, Moien AB Khan, Yusra H Khan, Shaghayegh Khanmohammadi, Moawiah Mohammad Khatatbeh, Sorour Khateri, Maryam Khayamzadeh, Hamid Reza Khayat Kashani, Min Seo Kim, Farzad Kompani, Hamid Reza Koohestani, Sindhura Lakshmi Koulmane Laxminarayana, Kewal Krishan, Narinder Kumar, Naveen Kumar, Tezer Kutluk, Ambily Kuttikkattu, Daphne Teck Ching Lai, Dharmesh Kumar Lal, Faris Hasan Lami, Savita Lasrado, Sang-woong Lee, Seung Won Lee, Yeong Yeh Lee, Yo Han Lee, Elvynna Leong, Ming-Chieh Li, Jue Liu, Farzan Madadizadeh, Ahmad R Mafi, Soleiman Mahjoub, Reza Malekzadeh, Ahmad Azam Malik, Iram Malik, Tauqeer Hussain Mallhi, Mohammad Ali Mansournia, Santi Martini, Elezebeth Mathews, Manu Raj Mathur, Jitendra Kumar Meena, Ritesh G Menezes, Reza Mirfakhraie, Seyed Kazem Mirinezhad, Mohammad Mirza-Aghazadeh-Attari, Prasanna Mithra, Ashraf Mohamadkhani, Soheil Mohammadi, Maryam Mohammadzadeh, Syam Mohan, Ali H Mokdad, Ahmed Al Montasir, Fateme Montazeri, Maryam Moradi, Mostafa Moradi Sarabi, Farhad Moradpour, Maliheh Moradzadeh, Paula Moraga, Abbas Mosapour, Majid Motaghinejad, Sumaira Mubarik, Jibran Sualeh Muhammad, Christopher J L Murray, Ahamarshan Jayaraman Nagarajan, Mohsen Naghavi, Shumaila Nargus, Zuhair S Natto, Biswa Prakash Nayak, Seyed Aria Nejadghaderi, Phuong The Nguyen, Robina Khan Niazi, Nafise Noroozi, Hassan Okati-Aliabad, Akinkunmi Paul Okekunle, Sokking Ong, Anu Mary Oommen, Jagadish Rao Padubidri, Ashok Pandey, Eun-Kee Park, Seoyeon Park, Siddhartha Pati, Shankargouda Patil, Rajan Paudel, Uttam Paudel, Majid Pirestani, Indrashis Podder, Ghazaleh Pourali, Mona Pourjafar, Akram Pourshams, Zahiruddin Quazi Syed, Raghu Anekal Radhakrishnan, Venkatraman Radhakrishnan, Mosiur Rahman, Shayan Rahmani, Vahid Rahmanian, Pushkal Sinduvadi Ramesh, Juwel Rana, Indu Ramachandra Rao, Sowmya J Rao, Sina Rashedi, Mohammad-Mahdi Rashidi, Nazila Rezaei, Negar Rezaei, Nima Rezaei, Saeid Rezaei, Mohsen Rezaeian, Gholamreza Roshandel, Chandan S N, Maha Mohamed Saber-Ayad, Siamak Sabour, Leila Sabzmakan, Basema Saddik, Umar Saeed, Sher Zaman Safi, Fatemeh Saheb Sharif-Askari, Amirhossein Sahebkar, Harihar Sahoo, Seyed Aidin Sajedi, Mirza Rizwan Sajid, Mohammad Amin Salehi, Amir Salek Farrokhi, Made Ary Sarasmita, Saman Sargazi, Gargi Sachin Sarode, Sachin C Sarode, Brijesh Sathian, Maheswar Satpathy, Prabhakar Semwal, Subramanian Senthilkumaran, Sadaf G Sepanlou, Melika Shafeghat, Saeed Shahabi, Ataollah Shahbandi, Fariba Shahraki-Sanavi, Masood Ali Shaikh, Mohammed Shannawaz, Rahim Ali Sheikhi, Parnian Shobeiri, Seyed Afshin Shorofi, Sunil Shrestha, Soraya Siabani, Garima Singh, Paramdeep Singh, Surjit Singh, Dhirendra Narain Sinha, Samarjeet Singh Siwal, Saraswathy Sreeram, Muhammad Suleman, Rizwan Suliankatchi Abdulkader, Iyad Sultan, Abida Sultana, Mohammad Tabish, Takahiro Tabuchi, Majid Taheri, Iman M Talaat, Arash Tehrani-Banihashemi, Mohamad-Hani Temsah, Pugazhenthan Thangaraju, Nihal Thomas, Nikhil Kenny Thomas, Amir Tiyuri, Ruoyan Tobe-Gai, Razie Toghroli, Marcos Roberto Tovani-Palone, Sana Ullah, Bhaskaran Unnikrishnan, Era Upadhyay, Sahel Valadan Tahbaz, Rohollah Valizadeh, Shoban Babu Varthya, Yasir Waheed, Song Wang, Dakshitha Praneeth Wickramasinghe, Nuwan Darshana Wickramasinghe, Hong Xiao, Naohiro Yonemoto, Mustafa Z Younis, Chuanhua Yu, Mazyar Zahir, Nazar Zaki, Maryam Zamanian, Zhi-Jiang Zhang, Hanqing Zhao, Osama A Zitoun, and Mohammad Zoladl.

## Affiliations

Humanities and Social Sciences (R Sharma PhD), National Institute of Technology Kurukshetra, Kurukshetra, Haryana, India; Advanced Diagnostic and Interventional Radiology Research Center (H Abbastabar PhD), Research Center for Immunodeficiencies (H Abolhassani PhD, Prof N Rezaei PhD), Non-communicable Diseases Research Center (S Azadnajafabad MD, M Keykhaei MD, F Montazeri MD, S Rahmani MD, M Rashidi MD, N Rezaei MD, N Rezaei PhD), School of Medicine (A Fallahzadeh MD, H Farrokhpour MD, H Karimi MD, S Khanmohammadi MD, S Mohammadi MD, M Shafeghat MD), Department of Ophthalmology (Prof F Ghassemi MD), Students' Scientific Research Center (SSRC) (M Keykhaei MD), Children's Medical Center (F Kompani MD), Digestive Diseases Research Institute (Prof R Malekzadeh MD, A Mohamadkhani PhD, Prof A Pourshams MD, S G Sepanlou MD), Department of Epidemiology and Biostatistics (M Mansournia PhD), Translational Ophthalmology Research Center, Farabi Eye Hospital (M Mohammadzadeh MD), Department of Pharmacology (N Noroozi DVM), Department of Cardiology (S Rashedi MD), Endocrinology and Metabolism Research Institute (N Rezaei PhD), Department of Medicine (M A Salehi MD, A Shahbandi MD (candidate)), Faculty of Medicine (P Shobeiri MD), Tehran University of Medical Sciences, Tehran, Iran; Community and Maternity Nursing Unit (D M Abdulah MPH), University of Duhok, Duhok, Iraq; Laboratory Technology Sciences Department (H Abidi PhD), Department of Nursing (M Zoladl PhD), Yasuj University of Medical Sciences, Yasuj, Iran; Department of Medical Biochemistry and Biophysics (H Abolhassani PhD), Department of Medicine (T S Hassan PhD), Karolinska Institute, Stockholm, Sweden; Cellular and Molecular Biology Department (Z Abrehdari-Tafreshi PhD), University of Tehran, Tehran, Iran; Medical Laboratory Sciences (A Absalan PhD), Khomein University of Medical Sciences, Khomein, Iran; Research and Development (A Absalan PhD), Satras Biotechnology Company, Tehran, Iran; Department of Banking and Finance (Prof H Abubaker Ali PhD), Department of Information Technology (A M Darwesh PhD), University of Human Development, Sulaymaniyah, Iraq; Clinical Sciences Department (E Abu-Gharbieh PhD, N R Dash FMD, M M Saber-Ayad MD, Prof I M Talaat PhD), Basic Medical Sciences (K Bajbouj PhD), Sharjah Institute for Medical Research (N M Elemam PhD, B Saddik PhD), Department of Basic Medical Sciences (W El-Huneidi PhD, J Muhammad PhD), Department of Clinical Nutrition and Dietetics (M E M Faris PhD), Clinical Sciences, College of Medicine (Prof R Hamoudi PhD), Sharjah Institute of Medical Sciences (F Saheb Sharif-Askari PhD), University of Sharjah, Sharjah, United Arab Emirates; Department of Clinical Medicine (Prof J M Acuna MD), American University of Antigua, Osbourn, Antigua and Barbuda; FIU Robert Stempel College of Public Health & Social Work (Prof J M Acuna MD), Florida International University, Miami, FL, USA; Veterinary Pathobiology (N Adib DVM), Shahid Bahonar University of Kerman, Kerman, Iran; Faculty of Medicine (Q E S Adnani PhD), Universitas Padjadjaran (Padjadjaran University), Bandung, Indonesia; Department of Epidemiology and Biostatistic (A Aghaei PhD), Social Determinants of Health Research Center, Research Institute for Health Development (A Aghaei PhD), School of Medicine (S Khateri MD), Social Determinants of Health Research Center (F Moradpour PhD), Kurdistan University of Medical Sciences, Sanandaj, Iran; Department of Medical Biochemistry (A Ahmad PhD), Department of Pharmacology (M Tabish MPharm), Shaqra University, Shaqra, Saudi Arabia; Department of Health and Biological Sciences (S Ahmad PhD), Abasyn University, Peshawar, Pakistan; Department of Natural Sciences (S Ahmad PhD), Lebanese American University, Beirut, Lebanon; Department of Epidemiology and Biostatistics (A Ahmadi PhD), Community-Oriented Nursing Midwifery Research Center (M Heidari PhD), Department of Health in Disasters and Emergencies (R Sheikhi BHlthSci), Shahrekord University of Medical Sciences, Shahrekord, Iran; Department of Epidemiology (A Ahmadi PhD, S Sabour PhD), School of Advanced Technologies in Medicine (S Ahmadi PhD), National Nutrition and Food Technology Research Institute (M Ajami PhD), Urology Department (M Bonakdar Hashemi MD), Psychiatric Nursing and Management Department (F Ghadirian PhD), Department of Medical Genetics (M Ghasemi PhD), Center for Comprehensive Genetic Services (M Ghasemi PhD), Cancer Research Center (M Gholamalizadeh PhD), Obstetrics and Gynecology Department (E Ghotbi MD), Oral and Maxillofacial Surgery Department OR Student Research Committee, School of Dentistry (H Haeri Boroojeni DMD), Medicine (M Heidari-Foroozan MD), Functional Neurosurgery Research Center (E Jamshidi PharmD), Department of Neurosurgery (H Khayat Kashani MD), Clinical Oncology (A R Mafi MD), Department of Genetics (R Mirfakhraie PhD), School of Medicine (F Montazeri MD, S Nejadghaderi MD, S Rahmani MD), Chronic Respiratory Disease Research Center, National Research Institute of Tuberculosis and Lung Diseases (NRITLD) (M Motaghinejad PhD), Social Determinants of Health Research Center (M Rashidi MD), Medical Ethics and Law Research Center (M Taheri PhD), Urology and Nephrology Research Center (M Zahir MD), Shahid Beheshti University of Medical Sciences, Tehran, Iran (M Khayamzadeh MD); Institute of Public Health (L A Ahmed PhD, I Elbarazi DrPH), Family Medicine Department (M A Khan MSc), Computer Science and Software Engineering/Big Data Analytics Center (Prof N Zaki PhD), United Arab Emirates University, Al Ain, United Arab Emirates; Department of Food and Nutrition Policy and Planning Research (M Ajami PhD), National Institute of Nutrition, Tehran, Iran; Geriatric and Long Term Care Department (H Al Hamad MD, B Sathian PhD), Rumailah Hospital (H Al Hamad MD), Hamad Medical Corporation, Doha, Qatar; Clinical Research Support Center (S Al Hasan PhD), Kagawa University Hospital, Miki-cho, Japan; Forensic Medicine Division (Prof R G Menezes MD), Imam Abdulrahman Bin Faisal University, Dammam, Saudi Arabia (F M Alanezi PhD); Global Centre for Environmental Remediation (A A S Al-Gheethi PhD), University of Newcastle, Newcastle, NSW, Australia; Cooperative Research Centre for Contamination Assessment and Remediation of the Environment, Newcastle, NSW, Australia (A A S Al-Gheethi PhD); Department of Health Services and Hospital Administration (M K Al-Hanawi PhD), Health Economics Research Group (M K Al-Hanawi PhD), Department of Community Medicine (R M Al-Raddadi PhD), Department of Pediatric Dentistry (Prof O A A Elmeligy PhD), Rabigh Faculty of Medicine (A A Malik PhD), Department of Dental Public Health (Z S Natto DrPH), King Abdulaziz University, Jeddah, Saudi Arabia; Department of Zoology (A Ali PhD), Abdul Wali Khan University Mardan, Mardan, Pakistan; Erbil Technical Health College (B A Ali PhD), Erbil Polytechnic University, Erbil, Iraq; School of Pharmacy (B A Ali PhD), Tishk International University, Erbil, Iraq; Pars Advanced and Minimally Invasive Medical Manners Research Center (Y Alimohamadi PhD), Health Management and Economics Research Center (J Arabloo PhD), Department of Medical Laboratory Sciences (F Dorostkar PhD), Minimally Invasive Surgery Research Center (A Kabir MD), The Five Senses Health Institute (S Rezaei MD), Trauma and Injury Research Center (M Taheri PhD), Preventive Medicine and Public Health Research Center (A Tehrani-Banihashemi PhD), Department of Community and Family Medicine (A Tehrani-Banihashemi PhD), Department of Epidemiology and Biostatistics (A Tiyuri MSc), Iran University of Medical Sciences, Tehran, Iran (M Moradi MD); Department of Health Policy and Management (Prof S M Aljunid PhD), Kuwait University, Kuwait, Kuwait; International Centre for Casemix and Clinical Coding (Prof S M Aljunid PhD), National University of Malaysia, Bandar Tun Razak, Malaysia; College of Dental Medicine (S A A Al-Maweri PhD), Qatar University, Doha, Qatar; Department of Medicine (S A Alqahatni MD), King Faisal Specialist Hospital & Research Center, Riyadh, Saudi Arabia; Department of Medicine (S A Alqahatni MD), Johns Hopkins University, Baltimore, MD, USA; Pathology and Microbiology (M AlQudah MD), Department of Public Health (Prof Y S Khader PhD), Jordan University of Science and Technology, Irbid, Jordan; CTAG (M AlQudah MD), Pediatric Services (I Sultan MD), King Hussein Cancer Center, Amman, Jordan; Applied Science Research Center (A Al-Tammemi MPH), Applied Science Private University, Amman, Jordan; Migration Health Division (A Al-Tammemi MPH), International Organization for Migration, Amman, Jordan; Department of Epidemiology and Biostatistics (Prof A Ansari-Moghaddam PhD), Health Promotion Research Center (H Okati-Aliabad PhD, F Shahraki-Sanavi PhD), Department of Biochemistry (S Sargazi PhD), Zahedan University of Medical Sciences, Zahedan, Iran; Department of Surgery (S Anwar PhD), Gadjah Mada University, Yogyakarta, Indonesia; Department of Pathology (R Anwer PhD), Imam Mohammad Ibn Saud Islamic University, Riyadh, Saudi Arabia; Department of Psychology (M Aqeel PhD, M Aqeel PhD), Foundation University Islamabad, Rawalpandi, Pakistan; Social Determinants of Health Research Center (M Arab-Zozani PhD), Department of Epidemiology and Biostatistics (A Tiyuri MSc), Birjand University of Medical Sciences, Birjand, Iran; Department of Paediatrics (Prof H Ariffin MD), University of Malaya, KUALA LUMPUR, Malaysia; University of Malaya Medical Centre (Prof H Ariffin MD), University of Malaya, Kuala Lumpur, Malaysia; Department of Health Sciences (A Artaman PhD), Zayed University, Dubai, United Arab Emirates; Department of Maternal and Child Health (J Arulappan DSc), Sultan Qaboos University, Muscat, Oman; University Institute of Radiological Sciences and Medical Imaging Technology (T Ashraf MS), University Institute of Public Health (A A Malik PhD, S Nargus PhD, S Nargus PhD), The University of Lahore, Lahore, Pakistan; Department of Nutrition (E Askari PhD), Department of Paramedical (N Galehdar PhD), Department of Clinical Biochemistry (M Moradi Sarabi PhD), Lorestan University of Medical Sciences, Khorramabad, Iran; Department of Medical Genetics (M Athar PhD), Science and Technology Unit (M Athar PhD), Umm Al-Qura University, Makkah, Saudi Arabia; Faculty of Nursing (M M W Atout PhD), Philadelphia University, Amman, Jordan; Gomal Center of Biochemistry and Biotechnology (M Badar PhD), Gomal University, Dera Ismail Khan, Pakistan; Department of Forensic Science (A D Badiye PhD), Government Institute of Forensic Science, Nagpur, India; Department of Nursing (N Baghcheghi PhD), Social Determinants of Health Research Center (H Koohestani PhD), Saveh University of Medical Sciences, Saveh, Iran; School of Medicine (S Bagherieh BSc, A Dehghan MD), Department of Environmental Health Engineering (A Fatehizadeh PhD), Isfahan University of Medical Sciences, Isfahan, Iran; School of Public Affairs (R Bai MD), Nanjing University of Science and Technology, Nanjing, China; Department of Microbiology (Prof S Baliga MD), Department of General Medicine (A N Bhat MD), Department of Internal Medicine (A Boloor MD), Forensic Medicine and Toxicology Department (H L Dsouza MD), Department of Community Medicine (N Joseph MD, P Mithra MD), Kasturba Medical College (Prof B Unnikrishnan MD), Manipal Academy of Higher Education, Mangalore, India; Miami Cancer Institute (M Bardhan MD), Baptist Health South Florida, Miami, FL, USA; Health Information Management (A Bashiri PhD), Maternal Fetal Medicine Research Center (K Hessami MD), Department of Environmental Health (M Hoseini PhD), Research Center for Health Sciences, Institute of Health (M Hoseini PhD), Shiraz Neuroscience Research Center (F Iravanpour PhD, M Jafarinia PhD), Non-communicable Disease Research Center (Prof R Malekzadeh MD, S G Sepanlou MD), Health Policy Research Center (S Shahabi PhD), Shiraz University of Medical Sciences, Shiraz, Iran; Department of Community Medicine and Family Medicine (P Baskaran MD), Department of Anatomy (Prof N Bhardwaj MD, Prof S G Dixit MD), Department of Physiology (Prof A Dixit MD), Department of Community Medicine (G Singh MD), Department of Pharmacology (S Singh MD, S B Varthya MD), All India Institute of Medical Sciences, Jodhpur, India; Academics (S Basu MD), Indian Institute of Public Health, Gurgaon, India; Department of Oral Pathology and Microbiology (U I Belgaumi MD), Krishna Vishwa Vidyapeeth deemed to be University, Karad, India; Department of Epidemiology and Biostatistics (A C Bermudez MD), University of the Philippines Manila, Manila, Philippines; Department of Epidemiology (A C Bermudez MD), Brown University, Providence, RI, USA; Physiology (B B Bhandari MD), Government Institute of Medical Sciences, Greater Noida, India; Department of Biostatistics and Epidemiology (Prof S Bitaraf PhD), Ahvaz Jundishapur University of Medical Sciences, Ahvaz, Iran; School of Public Health and Health Systems (Z A Butt PhD), School of Public Health Sciences (O A Zitoun MD), University of Waterloo, Waterloo, ON, Canada; Al Shifa School of Public Health (Z A Butt PhD), Al Shifa Trust Eye Hospital, Rawalpindi, Pakistan; ICMR School of Public Health (J Chadwick MD), Division of Epidemiology and Biostatistics (V K Kamal PhD), National Institute of Epidemiology, Chennai, India; Heart Failure and Structural Heart Disease Unit (J Chan MBChB), Cardiovascular Analytics Group, Hong Kong, China; Temerty Faculty of Medicine (V Chattu MDS), University of Toronto, Toronto, ON, Canada; Saveetha Dental College, SIMATS (V Chattu MDS), Centre of Molecular Medicine and Diagnostics (COMManD) (Prof S Patil PhD), Saveetha University, Chennai, India; Center for Cancer Epidemiology (Prof P Chaturvedi MD), Tata Memorial Hospital, Navi Mumbai, India; Department of Head Neck Surgery (Prof P Chaturvedi MD), Tata Memorial Hospital, Mumbai, India; Department of Clinical Oncology (W C S Cho DPhil), Queen Elizabeth Hospital, Hong Kong, China; GI Surgery (A Dhali MBBS), Institute of Post-Graduate Medical Education and Research and Seth Sukhlal Karnani Memorial Hospital, Kolkata, India; Department of Epidemiology (M Dianatinasab MSc), Sunway University, Malaysia; Department of Epidemiology (M Dianatinasab MSc), Maastricht University, Netherlands; Research Unit (M Dibas MD), Sulaiman Al Rajhi University, Qassim, Saudi Arabia; Forensic Medicine and Toxicology Department (H L Dsouza MD), Kasturba Medical College Mangalore, Mangalore, India; Natural & Medical Sciences Research Center (Prof E Elkord PhD), University of Nizwa, Nizwa, Oman; Biomedical Research Center, School of Science, Engineering and Environment (Prof E Elkord PhD), University of Salford, Salford, UK; Pediatric Dentistry and Dental Public Health Department (Prof O A A Elmeligy PhD), Pathology Department (Prof I M Talaat PhD), Alexandria University, Alexandria, Egypt; Ophthalmic Epidemiology Research Center (M Emamian PhD), Shahroud University of Medical Sciences, Shahroud, Iran; Department of International Cyber Education (R Erkhembayar MD), Mongolian National University of Medical Sciences, Ulaanbaatar, Mongolia; Clinical Biochemistry (R Ezzeddini PhD), Department of Clinical Biochemistry (A Mosapour PhD), Department of Parasitology and Entomology (M Pirestani PhD), Tarbiat Modares University, Tehran, Iran; Department of Oncology (Prof Z Fadoo MD), Aga Khan University, Karachi, Pakistan; Medicine and Society (R Faiz MPH), Independent Consultant, Male’, Maldives; Head of the Laboratory of Experimental Medicine (I R Fakhradiyev PhD), Kazakh National Medical University, Almaty, Kazakhstan; Endocrinology and Metabolism Population Sciences Institute (A Fallahzadeh MD), Endocrinology and Metabolism Research Institute (H Farrokhpour MD), Deparment of Epidemiology (M Heidari-Foroozan MD), Department of Epidemiology (S Khanmohammadi MD, S Nejadghaderi MD, S Rashedi MD), Department of International Studies (P Shobeiri MD), Non-Communicable Diseases Research Center (NCDRC), Tehran, Iran; Centre for Primary Health Care Network Management (H Fattahi PhD), Ministry of Health and Medical Education, Tehran, Iran; School of Pharmacy (G Fekadu MSc), Jockey Club School of Public Health and Primary Care (J Huang MD), The Chinese University of Hong Kong, Hong Kong, China; Department of Pharmacy (G Fekadu MSc), Wollega University, Nekemte, Ethiopia; Department of Dermatology (T Fukumoto PhD), Kobe University, Kobe, Japan; Department of Community Medicine (Prof A M Gaidhane MD, Prof Z Quazi Syed PhD), Datta Meghe Institute of Medical Sciences, Wardha, India; Obstetrics and Gynaecology (P Garg MD), Department of Radiodiagnosis (P Singh MD), All India Institute of Medical Sciences, Bathinda, India; Department of Medical Surgical Nursing (M Ghafourifard PhD), Department of Immunology (F Jadidi-Niaragh PhD), School of Management and Medical Informatics (L R Kalankesh PhD), Liver and Gastrointestinal Disease Research Center (S Mirinezhad PhD), Department of Radiology (M Mirza-Aghazadeh-Attari MD), Tabriz University of Medical Sciences, Tabriz, Iran; E-Learning Center (M Ghasemi Nour MD), Metabolic Syndrome Research Center (G Pourali MD), International UNESCO Center for Health-related Basic Sciences and Human Nutrition (G Pourali MD), Applied Biomedical Research Center (A Sahebkar PhD), Biotechnology Research Center (A Sahebkar PhD), Mashhad University of Medical Sciences, Mashhad, Iran; Young Researchers and Elite Club (A Gholamian MSc), Islamic Azad University, Rasht, Iran; Department of Biology (A Gholamian MSc), Department of Microbiology (S Valadan Tahbaz PhD), Islamic Azad University, Tehran, Iran; Health Systems and Policy Research Department (M Golechha PhD), Indian Institute of Public Health, Gandhinagar, India; Department of Genetics (P Goleij MSc), Sana Institute of Higher Education, Sari, Iran; Universal Scientific Education and Research Network (USERN) (P Goleij MSc), Department of Health Education and Health Promotion (S Siabani PhD), Kermanshah University of Medical Sciences, Kermanshah, Iran; Community Medicine Department (S Goyal MD), Maulana Azad Medical College, New Delhi, India; Department of Family and Community Medicine (M I M Gubari PhD), University Of Sulaimani, Sulaimani, Iraq; Department of Paediatrics (D S Gunasekera MD), National Cancer Institute, Maharagama, Sri Lanka; Department of Community Medicine (D A Gunawardane MD), University of Peradeniya, Kandy, Sri Lanka; Toxicology Department (S Gupta MSc), Shriram Institute for Industrial Research, Delhi, India; School of Medicine (P Habibzadeh MD), University of Maryland, Baltimore, MD, USA; College of Dentistry (E S Halboub PhD), Epidemiology Department (M Khan MD), Substance Abuse and Toxicology Research Center (S Mohan PhD), Jazan University, Jazan, Saudi Arabia; School of Dentistry (E S Halboub PhD), Sana'a University, Sana'a, Yemen; Department of Family and Community Medicine (Prof R R Hamadeh PhD), College of Medicine and Medical Sciences (H Jahrami PhD), Arabian Gulf University, Manama, Bahrain; Surgical Biotechnology, Royal Free Hospital, UCL (Prof R Hamoudi PhD), Department of Behavioural Science and Health (S Hossain MS), University College London, London, UK; Department of Nursing (M Harorani MSc), Department of Radiology (M Hasanian MD), Department of Epidemiology (M Zamanian PhD), Arak University of Medical Sciences, Arak, Iran; Research Centre (T S Hassan PhD), Department of Statistics (D H Kadir PhD), Salahaddin University, Erbil, Iraq; Institute for Health Metrics and Evaluation (Prof S I Hay FMedSci, A H Mokdad PhD, Prof C J L Murray DPhil, Prof M Naghavi PhD), Department of Health Metrics Sciences, School of Medicine (Prof S I Hay FMedSci, A H Mokdad PhD, Prof C J L Murray DPhil, Prof M Naghavi PhD), University of Washington, Seattle, WA, USA; Maternal Fetal Care Center (K Hessami MD), Department of Health Policy and Oral Epidemiology (Z S Natto DrPH), Harvard University, Boston, MA, USA; Department of Microbiology (K Hezam PhD), Taiz University, Taiz, Yemen; School of Medicine (K Hezam PhD), Nankai University, Tianjin, China; Division for Health Service Promotion (Y Hiraike PhD), University of Tokyo, Tokyo, Japan; Kasturba Medical College, Mangalore (R Holla MD, A Kamath MD), Manipal College of Dental Sciences (Prof R A Radhakrishnan PhD), Department of Nephrology (I Rao DM), Department of Pathology, Kasturba Medical College, Mangalore (S Sreeram MD), Manipal Academy of Higher Education, Manipal, India (A Kamath MD); Social and Environmental Health Research (M Hossain MPH), Nature Study Society of Bangladesh, Khulna, Bangladesh; Department of Health Promotion and Community Health Sciences (M Hossain MPH), Texas A&M University, College Station, TX, USA; Department of Public Health and Informatics (S Hossain MS), Jahangirnagar University, Dhaka, Bangladesh; Department of Health Services Administration (V Hsieh PhD), Department of Occupational Safety and Health (Prof B Hwang PhD), China Medical University, Taichung, Taiwan; Department of Biomolecular Sciences (N R Hussein PhD), University of Zakho, Zakho, Iraq; Department of Occupational Therapy (Prof B Hwang PhD), Asia University, Taichung, Taiwan; Department of Clinical Pharmacy & Pharmacy Practice (Prof N Ismail PhD), Asian Institute of Medicine, Science and Technology, Kedah, Malaysia; Malaysian Academy of Pharmacy, Puchong, Malaysia (Prof N Ismail PhD); Department of Health Services Research (M Iwagami PhD), University of Tsukuba, Tsukuba, Japan; Department of Non-Communicable Disease Epidemiology (M Iwagami PhD), London School of Hygiene & Tropical Medicine, London, UK; Department of Orthodontics & Dentofacial Orthopedics (L J BDS), Department of Oral Pathology and Microbiology (Prof G S Sarode PhD, Prof S C Sarode PhD), Dr. D.Y. Patil Dental College and Hospital, Pune, India; Social Determinants of Health Research Center (M A Jahani PhD), Cellular and Molecular Biology Research Center (Prof S Mahjoub PhD), Department of Clinical Biochemistry (Prof S Mahjoub PhD), Department of Biochemistry (A Mosapour PhD), Babol University of Medical Sciences, Babol, Iran; Ministry of Health (H Jahrami PhD), Ministry of Health, Manama, Bahrain; Centre for Community Medicine (A Jaiswal MD), Department of Preventive Oncology (J K Meena MD), All India Institute of Medical Sciences, New Delhi, India; Institute of Advanced Manufacturing Technologies (Prof M Jakovljevic PhD), Peter the Great St. Petersburg Polytechnic University, St. Petersburg, Russia; Institute of Comparative Economic Studies (Prof M Jakovljevic PhD), Hosei University, Tokyo, Japan; Department of Microbiology (M Jalili MSc), Research Center for Molecular Medicine (M Pourjafar PhD), Hamadan University of Medical Sciences, Hamadan, Iran; Division of Pulmonary Medicine (E Jamshidi PharmD), Lausanne University Hospital (CHUV), Lausanne, Switzerland; Postgraduate Institute of Medicine (U Jayarajah MD), Department of Surgery (D P Wickramasinghe MD), University of Colombo, Colombo, Sri Lanka; Department of Surgery (U Jayarajah MD), National Hospital, Colombo, Sri Lanka; Department of Biochemistry (Prof S Jayaram MBBS), Government Medical College, Mysuru, India; Dr. B. C. Roy Multi-Speciality Medical Research Centre (S S Jha MD), Indian Institute of Technology Kharagpur, Kharagpur, India; Zoonoses Research Center (M Jokar DVM), Islamic Azad University, Karaj, Iran; Department of Clinical Sciences (M Jokar DVM), Jahrom University of Medical Sciences, Jahrom, Iran; Deparment of Social Work (M Kabir PhD), Pabna University of Science and Technology, Pabna, Bangladesh; Independent Consultant, Pune, India (P V Kakodkar MDS); Social Determinants of Health Research Center (L R Kalankesh PhD), Gonabad University of Medical Sciences, Gonabad, Iran; Institute for Prevention of Non-communicable Diseases (R Kalhor PhD), Health Services Management Department (R Kalhor PhD), Qazvin University of Medical Sciences, Qazvin, Iran; Dermatology Department (F Kaliyadan MD), King Faisal University, Hofuf, Saudi Arabia; Biostatistics (V K Kamal PhD), Indian Council of Medical Research, New Delhi, India; Department of Pharmacy (Z Kamal PhD), Shaheed Benazir Bhutto University, Dir Upper, Pakistan; School of Pharmacy (Z Kamal PhD), Shanghai Jiao Tong University, Shanghai, China; Preventive & Social Medicine (Prof S S Kar MD), Jawaharlal Institute of Postgraduate Medical Education and Research, Puducherry, India; Department of ENT (N Kaur MS), Dr. B. R. Ambedkar State Institute of Medical Sciences (AIMS), Mohali, India; Department of Healthcare Services Management (L Keikavoosi-Arani PhD), Non-communicable Diseases Research Center (L Sabzmakan PhD), Alborz University of Medical Sciences, Karaj, Iran; Amity Institute of Forensic Sciences (H Khajuria PhD, B P Nayak PhD), Amity Institute of Public Health (M Shannawaz PhD), Amity University, Noida, India; Department of Epidemiology and Biostatistics (E A Khan MPH), Health Services Academy, Islamabad, Pakistan; Population Science Department (M Khan PhD), Jatiya Kabi Kazi Nazrul Islam University, Mymensingh, Bangladesh; Department of Public Health (M Khan PhD), University of Sydney, Sydney, NSW, Australia; Primary Care Department (M A Khan MSc), NHS North West London, London, UK; Clinical Pharmacy (Y H Khan PhD), Department of Clinical Pharmacy (T Mallhi PhD), Jouf University, Sakaka, Saudi Arabia; Department of Basic Medical Sciences (M M Khatatbeh PhD), Yarmouk University, Irbid, Jordan; The Iranian Academy of Medical Sciences, Tehran, Iran (M Khayamzadeh MD); Cardiovascular Disease Initiative (M Kim MD), Broad Institute of MIT and Harvard, Cambridge, MA, USA; Kasturba Medical College, Udupi, India (S Koulmane Laxminarayana MD); Department of Anthropology (Prof K Krishan PhD), Panjab University, Chandigarh, India; Department of Orthopaedics (Prof N Kumar MS), Medanta Hospital, Lucknow, India; Amity Institute of Biotechnology (N Kumar PhD, E Upadhyay PhD), Amity University Rajasthan, Jaipur, India; Department of Pediatric Oncology (Prof T Kutluk MD), Hacettepe University, Ankara, Türkiye; Department of Nephrology (A Kuttikkattu MD), Pushpagiri Institute of Medical Sciences and Research Centre, Thiruvalla, India; School of Digital Science (D T C Lai PhD), Institute of Applied Data Analytics (D T C Lai PhD), Universiti Brunei Darussalam, Gadong, Brunei; Health Policy Research (M R Mathur PhD), Public Health Foundation of India, Gurugram, India (D K Lal MD); Department of Community and Family Medicine (F H Lami PhD), University of Baghdad, Baghdad, Iraq; Department of Otorhinolaryngology (S Lasrado MS), Father Muller Medical College, Mangalore, India; Pattern Recognition and Machine Learning Lab (Prof S Lee PhD), Gachon University, Seongnam, South Korea; Department of Precision Medicine (Prof S W Lee MD), Sungkyunkwan University, Suwon-si, South Korea; Department of Medicine (Prof Y Lee PhD), School of Medical Sciences (Prof Y Lee PhD), University of Science Malaysia, Kota Bharu, Malaysia; Department of Preventive Medicine (Prof Y Lee PhD), Korea University, Seoul, South Korea; Faculty of Science (E Leong PhD), Universiti Brunei Darussalam (University of Brunei Darussalam), Bandar Seri Begawan, Brunei; Department of Health Promotion and Health Education (M Li PhD), National Taiwan Normal University, Taipei, Taiwan; Department of Epidemiology and Biostatistics (Prof J Liu PhD), Peking University, Beijing, China; Department of Biostatistics and Epidemiology (F Madadizadeh PhD), Yazd University of Medical Sciences, Yazd, Iran; Material Science Programme (I Malik PhD), Indian Institute of Technology Kanpur, Kanpur, India; Faculty of Public Health (S Martini PhD), Universitas Airlangga (Airlangga University), Surabaya, Indonesia; Indonesian Public Health Association, Surabaya, Indonesia (S Martini PhD); Department of Public Health and Community Medicine (E Mathews PhD), Central University of Kerala, Kasaragod, India; Institute of Population Health Sciences (M R Mathur PhD), University of Liverpool, Liverpool, UK; Social Determinants of Health Center (M Mirza-Aghazadeh-Attari MD), Urmia University of Medical Sciences, Urmia, Iran (R Valizadeh PhD); Center for Transdisciplinary Research (S Mohan PhD), Saveetha Institute of Medical and Technical Science, Chennai, India; Department of Medicine (A A Montasir FMD), TMSS Medical College, Bogura, Bangladesh; Department of Medicine (A A Montasir FMD), Sofia Ismail Memorial Medical Centre, Bogura, Bangladesh; Joint, Bone, Connective tissue, Rheumatology Research Center (JBCRC) (M Moradzadeh PhD), Golestan Research Center of Gastroenterology and Hepatology (G Roshandel PhD), Neurology Department (S Sajedi MD), Golestan University of Medical Sciences, Gorgan, Iran; Computer, Electrical, and Mathematical Sciences and Engineering Division (P Moraga PhD), King Abdullah University of Science and Technology, Thuwal, Saudi Arabia; Department of Epidemiology and Biostatistics (S Mubarik MS, Prof C Yu PhD), School of Medicine (Z Zhang PhD), Wuhan University, Wuhan, China; Research and Analytics Department (A J Nagarajan MTech), Initiative for Financing Health and Human Development, Chennai, India; Department of Research and Analytics (A J Nagarajan MTech), Bioinsilico Technologies, Chennai, India; Institute for Cancer Control (P T Nguyen MPH), National Cancer Center, Chuo-ku, Japan; Graduate school of public health (P T Nguyen MPH), St. Luke's International University, Chuo-ku, Japan; International Islamic University Islamabad, Islamabad, Pakistan (R K Niazi PhD); Department of Food and Nutrition (A P Okekunle PhD), Seoul National University, Seoul, South Korea; College of Medicine (A P Okekunle PhD), University of Ibadan, Ibadan, Nigeria; Non-communicable Disease Prevention Unit (S Ong FAMS), Ministry of Health, Bandar Seri Begawan, Brunei; Early Detection & Cancer Prevention Services (S Ong FAMS), Pantai Jerudong Specialist Centre, Bandar Seri Begawan, Brunei; Department of Community Health (Prof A M Oommen MD), Department of Endocrinology, Diabetes and Metabolism (Prof N Thomas PhD), Christian Medical College and Hospital (CMC), Vellore, India; Department of Forensic Medicine and Toxicology (J Padubidri MD), Kasturba Medical College, Mangalore, Mangalore, India; Research Department (A Pandey MPH), Research Section (U Paudel PhD), Nepal Health Research Council, Kathmandu, Nepal; Research Department (A Pandey MPH), Public Health Research Society Nepal, Kathmandu, Nepal; Department of Medical Humanities and Social Medicine (Prof E Park PhD), Kosin University, Busan, South Korea; Yonsei University College of Medicine, Seodaemun-gu, South Korea (S Park MD); Skills Innovation and Academic Network (SIAN) Institute (S Pati PhD), Association for Biodiversity Conservation and Research (ABC), Odisha, India; College of Dental Medicine (Prof S Patil PhD), Roseman University of Health Sciences, South Jordan, Utah, USA; Central Department of Public Health (R Paudel MPH), Faculty of Humanities and Social Sciences (U Paudel PhD), Tribhuvan University, Kathmandu, Nepal; Department of Dermatology (I Podder MD), College of Medicine and Sagore Dutta Hospital, Kolkata, India; Department of Medical Oncology (Prof V Radhakrishnan MD), Cancer Institute (W.I.A), Chennai, India; Department of Population Science and Human Resource Development (M Rahman DrPH), University of Rajshahi, Rajshahi, Bangladesh; Department of Public Health (V Rahmanian PhD), Torbat Jam Faculty of Medical Sciences, Torbat Jam, Iran; Department of Biochemistry (P S Ramesh PhD), JSS Medical College, JSS Academy of Higher Education & Research, Mysore, India; Department of Epidemiology, Biostatistics and Occupational Health (J Rana MPH), McGill University, Montreal, QC, Canada; Research and Innovation Division (J Rana MPH), South Asian Institute for Social Transformation (SAIST), Dhaka, Bangladesh; Department of Oral Pathology (S Rao MDS), Sharavathi Dental College and Hospital, Shimogga, India; Network of Immunity in Infection, Malignancy and Autoimmunity (NIIMA) (Prof N Rezaei PhD), Universal Scientific Education and Research Network (USERN), Tehran, Iran; Eye and Skull Base Research Centers (S Rezaei MD), Rassoul Akram Hospital, Tehran, Iran; Department of Epidemiology and Biostatistics (Prof M Rezaeian PhD), Rafsanjan University of Medical Sciences, Rafsanjan, Iran; Oral and Maxillofacial Surgery (C S N PhD), Jagadguru Sri Shivarathreeswara University, Mysore, India; Department of Medical Pharmacology (M M Saber-Ayad MD), Cairo University, Giza, Egypt; Multidisciplinary Laboratory Foundation University School of Health Sciences (FUSH) (Prof U Saeed PhD), Foundation University, Islamabad, Pakistan; International Center of Medical Sciences Research (ICMSR), Islamabad, Pakistan (Prof U Saeed PhD); Faculty of Medicine, Bioscience and Nursing (S Z Safi PhD), MAHSA University, Selangor, Malaysia; Interdisciplinary Research Centre in Biomedical Materials (IRCBM) (S Z Safi PhD), COMSATS Institute of Information Technology, Lahore, Pakistan; Department of Development Studies (H Sahoo PhD), International Institute for Population Sciences, Mumbai, India; Department of Statistics (M R Sajid PhD), University of Gujrat, Pakistan, Gujrat, Pakistan; Department of Immunology (A Salek Farrokhi PhD), Pasteur Institute of Iran, Tehran, Iran; Pharmacy Study Program (M A Sarasmita PharmD), Udayana University, Badung, Indonesia; Department of Clinical Pharmacy (M A Sarasmita PharmD), Taipei Medical University, Taipei, Taiwan; Faculty of Health & Social Sciences (B Sathian PhD), Bournemouth University, Bournemouth, UK; UGC Centre of Advanced Study in Psychology (M Satpathy PhD), Utkal University, Bhubaneswar, India; Udyam-Global Association for Sustainable Development, Bhubaneswar, India (M Satpathy PhD); Department of Life Sciences (P Semwal PhD), Graphic Era Deemed to be University, Dehradun, India; Emergency Department (S Senthilkumaran MD), Manian Medical Centre, Erode, India; Independent Consultant, Karachi, Pakistan (M A Shaikh MD); Department of Medical-Surgical Nursing (S Shorofi PhD), Mazandaran University of Medical Sciences, Sari, Iran; Department of Nursing and Health Sciences (S Shorofi PhD), Flinders University, Adelaide, SA, Australia; School of Pharmacy (S Shrestha PharmD), Monash University, Selangor Darul Ehsan, Malaysia; School of Health (S Siabani PhD), University of Technology Sydney, Sydney, NSW, Australia; Department of Community Medicine (G Singh MD), Lady Hardinge Medical College, New Delhi, India; Department of Epidemiology (D N Sinha PhD), School of Preventive Oncology registered as a Trust, Patna, India; Department of Epidemiology (D N Sinha PhD), Healis Sekhsaria Institute for Public Health, Mumbai, India; Department of Chemistry (S S Siwal PhD), Maharishi Markandeshwar (Deemed to be University), Mullana- Ambala, India, Mullana, India; Center for Biotechnology and Microbiology (M Suleman PhD), University of Swat, Mingora, Pakistan; School of Life Sciences (M Suleman PhD), Xiamen University, Xiamen, China; National Institute of Epidemiology (R Suliankatchi Abdulkader MD), Indian Council of Medical Research, Chennai, India; Pediatrics (I Sultan MD), University of Jordan, Amman, Jordan; Mental Health Research (A Sultana MD), Independent Consultant, Khulna, Bangladesh; Division of Global Mental Health (A Sultana MD), EviSyn Health, Khulna, Bangladesh; Cancer Control Center (T Tabuchi MD), Osaka International Cancer Institute, Osaka, Japan; Pediatric Intensive Care Unit (M Temsah MD), King Saud University, Riyadh, Saudi Arabia; Department of Pharmacology (P Thangaraju MD), All India Institute of Medical Sciences, Raipur, India; Department of Gastroenterology (N K Thomas MD), PSG Institute of Medical Sciences and Research, Coimbatore, India; Department of Social Security Empirical Research (Prof R Tobe-Gai PhD), National Institute of Population and Social Security Research, Tokyo, Japan; Social Determinants of Health (R Toghroli PhD), Hormozgan University of Medical Sciences, Bandar Abbas, Iran; Saveetha Dental College and Hospitals (M R Tovani-Palone PhD), Saveetha Institute of Medical and Technical Sciences (SIMATS), Chennai, India; SRM College of Pharmacy (M R Tovani-Palone PhD), SRM Institute of Science and Technology (SRMIST), Chennai, India; Department of Zoology (S Ullah PhD), Division of Science and Technology (S Ullah PhD), University of Education, Lahore, Lahore, Pakistan; Clinical Cancer Research Center (S Valadan Tahbaz PhD), Milad General Hospital, Tehran, Iran; Office of Research, Innovation, and Commercialization (ORIC) (Prof Y Waheed PhD), Shaheed Zulfiqar Ali Bhutto Medical University (SZABMU), Islamabad, Pakistan; Gilbert and Rose-Marie Chagoury School of Medicine (Prof Y Waheed PhD), Lebanese American University, Byblos, Lebanon; Department of Gastroenterology (S Wang PhD), The First Affiliated Hospital of USTC, University of Science and Technology of China, Hefei, China; Department of Community Medicine (N D Wickramasinghe MD), Rajarata University of Sri Lanka, Anuradhapura, Sri Lanka; School of Public Health (H Xiao PhD), Zhejiang University, Zhejiang, China; Department of Public Health Science (H Xiao PhD), Fred Hutchinson Cancer Research Center, Seattle, WA, USA; Department of Neuropsychopharmacology (N Yonemoto PhD), National Center of Neurology and Psychiatry, Kodaira, Japan; Department of Public Health (N Yonemoto PhD), Juntendo University, Tokyo, Japan; Department of Health Policy and Management (Prof M Z Younis PhD), Jackson State University, Jackson, MS, USA; School of Business & Economics (Prof M Z Younis PhD), Universiti Putra Malaysia (University of Putra Malaysia), Kuala Lumpur, Malaysia; College of Traditional Chinese Medicine (H Zhao MD), Hebei University, Baoding, China; College of Medicine (O A Zitoun MD), Sulaiman Alrajhi University, Al Bukairiyah, Saudi Arabia.

## Contributors

Please see Appendix (pp 1–4) for more detailed information about individual author contributions to the research, divided into the following categories: managing the overall research enterprise; writing the first draft of the manuscript; primary responsibility for applying analytical methods to produce estimates; primary responsibility for seeking, cataloguing, extracting, or cleaning data; designing or coding figures and tables; providing data or critical feedback on data sources; developing methods or computational machinery; providing critical feedback on methods or results; drafting the manuscript or revising it critically for important intellectual content; and managing the estimation or publications process.

## Data sharing statement

Data used in this article are available for download on the Global Health Data Exchange tool, which is permitted to be used, shared, modified, or built on by non-commercial users via the Open Data Commons Attribution License. All GBD 2019 data are publicly available and can be downloaded via the Global Health Data Exchange tool (https://vizhub.healthdata.org/gbd-results/) and from the GBD Compare Visualisation Tool (https://vizhub.healthdata.org/gbd-compare).

## Declaration of interests

R Bai reports support for the present manuscript from in part by the National Natural Science Foundation of China (grant number: 72204112), the Social Science Fund of Jiangsu Province (grant: number 21GLD008), and the Fundamental Research Funds for the Central Universities (grant number: 30923011101); the content of this paper is solely the responsibility of the authors and does not necessarily represent the official views of the funders; the funder had no role in the study design, data collection, data analysis, data interpretation, or writing of the report. N E Ismail reports leadership or fiduciary roles in board, society, committee or advocacy groups, paid or unpaid with Malaysian Academy of Pharmacy, Malaysia as an unpaid Bursar outside the submitted work. K Krishan acknowledges non-financial support from the UGC Centre of Advanced Study, CAS II, awarded to the Department of Anthropology, Panjab University, Chandigarh, India; all outside the submitted work. MC Li reports support by the National Science and Technology Council in Taiwan (112-2410-H-003-031); and other support from Journal of the American Heart Association as Technical Editor, Frontiers in Public Health as Review Editor, and BMC Public Health as Editorial Board Member; all outside the submitted work. E Mathews reports grants or contracts from Wellcome DBT India Alliance, outside the submitted work.
