# Supplementary material for: Temporal patterns of cancer burden in Asia, 1990–2019: a systematic examination for the Global Burden of Disease 2019 study
Source: Lancet Reg Health Southeast Asia. 2024 Jan 2;21:100333. doi: 10.1016/j.lansea.2023.100333 (PMC10866992; doi:10.1016/j.lansea.2023.100333)
Supplement: Supplementary Material [file mmc1.docx]

# Writing the first draft of the manuscript

Rajesh Sharma

# Providing data or critical feedback on data sources

Rajesh Sharma, Hedayat Abbastabar, Hassan Abidi, Hassan Abolhassani, Hiwa Abubaker Ali, Qorinah Estiningtyas Sakilah Adnani, Sajjad Ahmad, Ali Ahmadi, Sepideh Ahmadi, Hanadi Al Hamad, Fahad Mashhour Alanezi, Abid Ali, Beriwan Abdulqadir Ali, Syed Mohamed Aljunid, Saleh A Alqahatni, Ala'a B. Al-Tammemi, Sumadi Lukman Anwar, Jalal Arabloo, Hany Ariffin, Al Artaman, Judie Arulappan, Tahira Ashraf, Maha Moh'd Wahbi Atout, Ashish D Badiye, Sara Bagherieh, Mainak Bardhan, Uzma Iqbal Belgaumi, Ajay Nagesh Bhat, Archith Boloor, Joshua Chadwick, Vijay Kumar Chattu, William C S Cho, Aso Mohammad Darwesh, Arkadeep Dhali, Mostafa Dianatinasab, Mahmoud Dibas, Shilpi Gupta Dixit, Fariba Dorostkar, Haneil Larson Dsouza, Noha Mousaad Elemam, Mohammad Hassan Emamian, Zehra Fadoo, Ildar Ravisovich Fakhradiyev, Hossein Farrokhpour, Ali Fatehizadeh, Takeshi Fukumoto, Fataneh Ghadirian, Mansour Ghafourifard, Elena Ghotbi, Mahaveer Golechha, Pouya Goleij, Sahil Goyal, Sapna Gupta, Rifat Hamoudi, Treska S Hassan, Nawfal R Hussein, Farideh Iravanpour, Nahlah Elkudssiah Ismail, Linda Merin J, Morteza Jafarinia, Mohammad Ali Jahani, Haitham Jahrami, Mihajlo Jakovljevic, Mahsa Jalili, Shubha Jayaram, Dler Hussein Kadir, Leila R Kalankesh, Rohollah Kalhor, Zul Kamal, Yousef Saleh Khader, Himanshu Khajuria, M Nuruzzaman Khan, Maseer Khan, Moien AB Khan, Yusra H Khan, Shaghayegh Khanmohammadi, Sorour Khateri, Sindhura Lakshmi Koulmane Laxminarayana, Kewal Krishan, Daphne Teck Ching Lai, Dharmesh Kumar Lal, Savita Lasrado, Sang-woong Lee, Seung Won Lee, Yo Han Lee, Jue Liu, Iram Malik, Tauqeer Hussain Mallhi, Mohammad Ali Mansournia, Manu Raj Mathur, Jitendra Kumar Meena, Ritesh G Menezes, Seyed Kazem Mirinezhad, Prasanna Mithra, Ali H Mokdad, Maryam Moradi, Sumaira Mubarik, Jibran Sualeh Muhammad, Ahamarshan Jayaraman Nagarajan, Mohsen Naghavi, Zuhair S Natto, Biswa Prakash Nayak, Robina Khan Niazi, Sokking Ong, Jagadish Rao Padubidri, Ashok Pandey, Siddhartha Pati, Shankargouda Patil, Zahiruddin Quazi Syed, Raghu Anekal Radhakrishnan, Pushkal Sinduvadi Ramesh, Juwel Rana, Sowmya J Rao, Sina Rashedi, Nima Rezaei, Gholamreza Roshandel, Siamak Sabour, Basema Saddik, Umar Saeed, Sher Zaman Safi, Mirza Rizwan Sajid, Saman Sargazi, Brijesh Sathian, Maheswar Satpathy, Prabhakar Semwal, Masood Ali Shaikh, Mohammed Shannawaz, Parnian Shobeiri, Sunil Shrestha, Soraya Siabani, Garima Singh, Paramdeep Singh, Samarjeet Singh Siwal, Saraswathy Sreeram, Muhammad Suleman, Iyad Sultan, Mohammad Tabish, Takahiro Tabuchi, Iman M Talaat, Pugazhenthan Thangaraju, Nihal Thomas, Nikhil Kenny Thomas, Amir Tiyuri, Razie Toghroli, Marcos Roberto Tovani-Palone, Sana Ullah, Bhaskaran Unnikrishnan, Era Upadhyay, Sahel Valadan Tahbaz, Shoban Babu Varthya, Yasir Waheed, Naohiro Yonemoto, Mustafa Z Younis, Chuanhua Yu, Zhi-Jiang Zhang, and Mohammad Zoladl.

# Developing methods or computational machinery

Rajesh Sharma, Hiwa Abubaker Ali, Qorinah Estiningtyas Sakilah Adnani, Ali Ahmadi, Ala'a B. Al-Tammemi, Morteza Arab-Zozani, Judie Arulappan, Milad Bonakdar Hashemi, Aso Mohammad Darwesh, Mostafa Dianatinasab, Ali Fatehizadeh, Mohammad Hasanian, Simon I Hay, Mohammad Heidari, Farideh Iravanpour, Linda Merin J, Morteza Jafarinia, Mahsa Jalili, Dler Hussein Kadir, Leila Keikavoosi-Arani, M Nuruzzaman Khan, Sorour Khateri, Sang-woong Lee, Ali H Mokdad, Christopher J L Murray, Mohsen Naghavi, Zahiruddin Quazi Syed, Umar Saeed, Mohammad Amin Salehi, Maheswar Satpathy, Mohammed Shannawaz, Samarjeet Singh Siwal, and Muhammad Suleman.

# Providing critical feedback on methods or results

Rajesh Sharma, Hedayat Abbastabar, Deldar Morad Abdulah, Hassan Abidi, Hassan Abolhassani, Zahra Abrehdari-Tafreshi, Abdorrahim Absalan, Hiwa Abubaker Ali, Eman Abu-Gharbieh, Juan Manuel Acuna, Nasrin Adib, Qorinah Estiningtyas Sakilah Adnani, Abbas Aghaei, Aqeel Ahmad, Sajjad Ahmad, Ali Ahmadi, Luai A Ahmed, Hanadi Al Hamad, Syed Mahfuz Al Hasan, Fahad Mashhour Alanezi, Adel Ali Saeed Al-Gheethi, Mohammed Khaled Al-Hanawi, Abid Ali, Yousef Alimohamadi, Syed Mohamed Aljunid, Ala'a B. Al-Tammemi, Alireza Ansari-Moghaddam, Sumadi Lukman Anwar, Razique Anwer, Muhammad Aqeel, Jalal Arabloo, Morteza Arab-Zozani, Hany Ariffin, Al Artaman, Judie Arulappan, Tahira Ashraf, Mohammad Athar, Maha Moh'd Wahbi Atout, Sina Azadnajafabad, Muhammad Badar, Ashish D Badiye, Nayereh Baghcheghi, Sara Bagherieh, Ruhai Bai, Shrikala Baliga, Mainak Bardhan, Pritish Baskaran, Saurav Basu, Uzma Iqbal Belgaumi, Amiel Nazer C Bermudez, Bharti Bhandari, Nikha Bhardwaj, Ajay Nagesh Bhat, Archith Boloor, Milad Bonakdar Hashemi, Zahid A Butt, Joshua Chadwick, Vijay Kumar Chattu, Pankaj Chaturvedi, William C S Cho, Aso Mohammad Darwesh, Nihar Ranjan Dash, Amin Dehghan, Arkadeep Dhali, Mostafa Dianatinasab, Mahmoud Dibas, Abhinav Dixit, Fariba Dorostkar, Haneil Larson Dsouza, Noha Mousaad Elemam, Waseem El-Huneidi, Eyad Elkord, Omar Abdelsadek Abdou Elmeligy, Mohammad Hassan Emamian, Rana Ezzeddini, Zehra Fadoo, Razana Faiz, Ildar Ravisovich Fakhradiyev, Aida Fallahzadeh, MoezAlIslam Ezzat Mahmoud Faris, Hossein Farrokhpour, Ali Fatehizadeh, Hamed Fattahi, Ginenus Fekadu, Takeshi Fukumoto, Abhay Motiramji Gaidhane, Nasrin Galehdar, Priyanka Garg, Fataneh Ghadirian, Mansour Ghafourifard, Mohammad Ghasemi Nour, Maryam Gholamalizadeh, Asadollah Gholamian, Elena Ghotbi, Mahaveer Golechha, Sahil Goyal, Mohammed Ibrahim Mohialdeen Gubari, Damitha Asanga Gunawardane, Sapna Gupta, Parham Habibzadeh, Esam S Halboub, Randah R Hamadeh, Rifat Hamoudi, Mehdi Harorani, Treska S Hassan, Simon I Hay, Mohammad Heidari, Mahsa Heidari-Foroozan, Kamran Hessami, Kamal Hezam, Yuta Hiraike, Ramesh Holla, Mohammad Hoseini, Md Mahbub Hossain, Sahadat Hossain, Vivian Chia-rong Hsieh, Nawfal R Hussein, Bing-Fang Hwang, Farideh Iravanpour, Nahlah Elkudssiah Ismail, Masao Iwagami, Linda Merin J, Farhad Jadidi-Niaragh, Morteza Jafarinia, Haitham Jahrami, Abhishek Jaiswal, Mihajlo Jakovljevic, Mahsa Jalili, Elham Jamshidi, Umesh Jayarajah, Shubha Jayaram, Sweety Suman Jha, Mohammad Jokar, Nitin Joseph, Ali Kabir, Md. Awal Kabir, Dler Hussein Kadir, Pradnya Vishal Kakodkar, Laleh R Kalankesh, Leila R Kalankesh, Rohollah Kalhor, Feroze Kaliyadan, Vineet Kumar Kamal, Zul Kamal, Ashwin Kamath, Sitanshu Sekhar Kar, Hanie Karimi, Navjot Kaur, Leila Keikavoosi-Arani, Mohammad Keykhaei, Yousef Saleh Khader, Himanshu Khajuria, Ejaz Ahmad Khan, M Nuruzzaman Khan, Maseer Khan, Moien AB Khan, Yusra H Khan, Shaghayegh Khanmohammadi, Moawiah Mohammad Khatatbeh, Sorour Khateri, Min Seo Kim, Farzad Kompani, Hamid Reza Koohestani, Sindhura Lakshmi Koulmane Laxminarayana, Kewal Krishan, Narinder Kumar, Naveen Kumar, Ambily Kuttikkattu, Daphne Teck Ching Lai, Dharmesh Kumar Lal, Savita Lasrado, Sang-woong Lee, Seung Won Lee, Yeong Yeh Lee, Yo Han Lee, Elvynna Leong, Ming-Chieh Li, Jue Liu, Farzan Madadizadeh, Ahmad R Mafi, Soleiman Mahjoub, Reza Malekzadeh, Ahmad Azam Malik, Tauqeer Hussain Mallhi, Mohammad Ali Mansournia, Santi Martini, Manu Raj Mathur, Jitendra Kumar Meena, Ritesh G Menezes, Mohammad Mirza-Aghazadeh-Attari, Prasanna Mithra, Ashraf Mohamadkhani, Soheil Mohammadi, Maryam Mohammadzadeh, Syam Mohan, Ali H Mokdad, Ahmed Al Montasir, Fateme Montazeri, Maryam Moradi, Mostafa Moradi Sarabi, Farhad Moradpour, Maliheh Moradzadeh, Abbas Mosapour, Majid Motaghinejad, Sumaira Mubarik, Jibran Sualeh Muhammad, Christopher J L Murray, Ahamarshan Jayaraman Nagarajan, Mohsen Naghavi, Shumaila Nargus, Zuhair S Natto, Biswa Prakash Nayak, Seyed Aria Nejadghaderi, Phuong The Nguyen, Robina Khan Niazi, Nafise Noroozi, Hassan Okati-Aliabad, Akinkunmi Paul Okekunle, Anu Mary Oommen, Jagadish Rao Padubidri, Ashok Pandey, Eun-Kee Park, Seoyeon Park, Siddhartha Pati, Shankargouda Patil, Rajan Paudel, Uttam Paudel, Ghazaleh Pourali, Akram Pourshams, Zahiruddin Quazi Syed, Raghu Anekal Radhakrishnan, Venkatraman Radhakrishnan, Mosiur Rahman, Shayan Rahmani, Vahid Rahmanian, Pushkal Sinduvadi Ramesh, Juwel Rana, Indu Ramachandra Rao, Sowmya J Rao, Sina Rashedi, Mohammad-Mahdi Rashidi, Nazila Rezaei, Negar Rezaei, Nima Rezaei, Saeid Rezaei, Mohsen Rezaeian, Gholamreza Roshandel, Chandan S N, Maha Mohamed Saber-Ayad, Siamak Sabour, Leila Sabzmakan, Basema Saddik, Umar Saeed, Sher Zaman Safi, Fatemeh Saheb Sharif-Askari, Harihar Sahoo, Seyed Aidin Sajedi, Mirza Rizwan Sajid, Mohammad Amin Salehi, Amir Salek Farrokhi, Saman Sargazi, Gargi Sachin Sarode, Sachin C Sarode, Brijesh Sathian, Maheswar Satpathy, Prabhakar Semwal, Subramanian Senthilkumaran, Sadaf G Sepanlou, Melika Shafeghat, Ataollah Shahbandi, Fariba Shahraki-Sanavi, Masood Ali Shaikh, Mohammed Shannawaz, Rahim Ali Sheikhi, Parnian Shobeiri, Seyed Afshin Shorofi, Sunil Shrestha, Soraya Siabani, Garima Singh, Paramdeep Singh, Surjit Singh, Dhirendra Narain Sinha, Samarjeet Singh Siwal, Saraswathy Sreeram, Muhammad Suleman, Rizwan Suliankatchi Abdulkader, Iyad Sultan, Abida Sultana, Majid Taheri, Iman M Talaat, Mohamad-Hani Temsah, Pugazhenthan Thangaraju, Nihal Thomas, Nikhil Kenny Thomas, Amir Tiyuri, Ruoyan Tobe-Gai, Razie Toghroli, Marcos Roberto Tovani-Palone, Sana Ullah, Bhaskaran Unnikrishnan, Era Upadhyay, Sahel Valadan Tahbaz, Rohollah Valizadeh, Shoban Babu Varthya, Yasir Waheed, Song Wang, Dakshitha Praneeth Wickramasinghe, Nuwan Darshana Wickramasinghe, Hong Xiao, Naohiro Yonemoto, Mustafa Z Younis, Chuanhua Yu, Mazyar Zahir, Nazar Zaki, Maryam Zamanian, Hanqing Zhao, Osama A Zitoun, and Mohammad Zoladl.

# Drafting the work or revising it critically for important intellectual content

Rajesh Sharma, Hedayat Abbastabar, Hassan Abidi, Hassan Abolhassani, Eman Abu-Gharbieh, Juan Manuel Acuna, Nasrin Adib, Qorinah Estiningtyas Sakilah Adnani, Ali Ahmadi, Sepideh Ahmadi, Luai A Ahmed, Marjan Ajami, Mohammed Khaled Al-Hanawi, Abid Ali, Sadeq Ali Ali Al-Maweri, Saleh A Alqahatni, Mohammad AlQudah, Rajaa M Al-Raddadi, Ala'a B. Al-Tammemi, Razique Anwer, Jalal Arabloo, Morteza Arab-Zozani, Hany Ariffin, Judie Arulappan, Elaheh Askari, Mohammad Athar, Maha Moh'd Wahbi Atout, Sina Azadnajafabad, Muhammad Badar, Ashish D Badiye, Sara Bagherieh, Shrikala Baliga, Mainak Bardhan, Azadeh Bashiri, Saurav Basu, Uzma Iqbal Belgaumi, Ajay Nagesh Bhat, Saeid Bitaraf, Milad Bonakdar Hashemi, Joshua Chadwick, Jeffrey Shi Kai Chan, Vijay Kumar Chattu, Pankaj Chaturvedi, William C S Cho, Nihar Ranjan Dash, Amin Dehghan, Arkadeep Dhali, Mostafa Dianatinasab, Mahmoud Dibas, Abhinav Dixit, Shilpi Gupta Dixit, Haneil Larson Dsouza, Iffat Elbarazi, Noha Mousaad Elemam, Omar Abdelsadek Abdou Elmeligy, Mohammad Hassan Emamian, Rana Ezzeddini, Zehra Fadoo, Aida Fallahzadeh, MoezAlIslam Ezzat Mahmoud Faris, Ali Fatehizadeh, Takeshi Fukumoto, Nasrin Galehdar, Priyanka Garg, Fataneh Ghadirian, Mansour Ghafourifard, MohammadReza Ghasemi, Mohammad Ghasemi Nour, Fariba Ghassemi, Elena Ghotbi, Sahil Goyal, D Sanjeeva Gunasekera, Damitha Asanga Gunawardane, Sapna Gupta, Parham Habibzadeh, Helia Sadat Haeri Boroojeni, Esam S Halboub, Randah R Hamadeh, Rifat Hamoudi, Treska S Hassan, Simon I Hay, Kamran Hessami, Kamal Hezam, Yuta Hiraike, Ramesh Holla, Md Mahbub Hossain, Sahadat Hossain, Vivian Chia-rong Hsieh, Junjie Huang, Farideh Iravanpour, Nahlah Elkudssiah Ismail, Linda Merin J, Morteza Jafarinia, Haitham Jahrami, Abhishek Jaiswal, Mihajlo Jakovljevic, Mahsa Jalili, Umesh Jayarajah, Shubha Jayaram, Nitin Joseph, Ali Kabir, Laleh R Kalankesh, Feroze Kaliyadan, Ashwin Kamath, Hanie Karimi, Navjot Kaur, Yousef Saleh Khader, Himanshu Khajuria, Ejaz Ahmad Khan, M Nuruzzaman Khan, Maseer Khan, Moien AB Khan, Yusra H Khan, Sorour Khateri, Maryam Khayamzadeh, Hamid Reza Khayat Kashani, Min Seo Kim, Farzad Kompani, Sindhura Lakshmi Koulmane Laxminarayana, Kewal Krishan, Narinder Kumar, Naveen Kumar, Tezer Kutluk, Faris Hasan Lami, Savita Lasrado, Yeong Yeh Lee, Elvynna Leong, Farzan Madadizadeh, Ahmad R Mafi, Soleiman Mahjoub, Reza Malekzadeh, Ahmad Azam Malik, Iram Malik, Tauqeer Hussain Mallhi, Elezebeth Mathews, Jitendra Kumar Meena, Ritesh G Menezes, Reza Mirfakhraie, Mohammad Mirza-Aghazadeh-Attari, Soheil Mohammadi, Maryam Mohammadzadeh, Ali H Mokdad, Ahmed Al Montasir, Fateme Montazeri, Maryam Moradi, Mostafa Moradi Sarabi, Paula Moraga, Abbas Mosapour, Majid Motaghinejad, Jibran Sualeh Muhammad, Christopher J L Murray, Ahamarshan Jayaraman Nagarajan, Mohsen Naghavi, Shumaila Nargus, Zuhair S Natto, Biswa Prakash Nayak, Seyed Aria Nejadghaderi, Robina Khan Niazi, Akinkunmi Paul Okekunle, Anu Mary Oommen, Jagadish Rao Padubidri, Shankargouda Patil, Uttam Paudel, Majid Pirestani, Indrashis Podder, Ghazaleh Pourali, Mona Pourjafar, Zahiruddin Quazi Syed, Venkatraman Radhakrishnan, Shayan Rahmani, Indu Ramachandra Rao, Sowmya J Rao, Nazila Rezaei, Nima Rezaei, Saeid Rezaei, Gholamreza Roshandel, Maha Mohamed Saber-Ayad, Leila Sabzmakan, Basema Saddik, Umar Saeed, Fatemeh Saheb Sharif-Askari, Amirhossein Sahebkar, Mirza Rizwan Sajid, Amir Salek Farrokhi, Made Ary Sarasmita, Gargi Sachin Sarode, Sachin C Sarode, Maheswar Satpathy, Sadaf G Sepanlou, Melika Shafeghat, Saeed Shahabi, Mohammed Shannawaz, Parnian Shobeiri, Seyed Afshin Shorofi, Sunil Shrestha, Paramdeep Singh, Dhirendra Narain Sinha, Samarjeet Singh Siwal, Saraswathy Sreeram, Muhammad Suleman, Iyad Sultan, Abida Sultana, Mohammad Tabish, Takahiro Tabuchi, Majid Taheri, Iman M Talaat, Arash Tehrani-Banihashemi, Mohamad-Hani Temsah, Pugazhenthan Thangaraju, Nihal Thomas, Marcos Roberto Tovani-Palone, Bhaskaran Unnikrishnan, Era Upadhyay, Sahel Valadan Tahbaz, Shoban Babu Varthya, Yasir Waheed, Dakshitha Praneeth Wickramasinghe, Nuwan Darshana Wickramasinghe, Naohiro Yonemoto, Mazyar Zahir, Maryam Zamanian, Hanqing Zhao, Osama A Zitoun, and Mohammad Zoladl.

# Managing the estimation or publications process

Rajesh Sharma, Simon I Hay, Ali H Mokdad, Christopher J L Murray and Mohsen Naghavi

# Supplementary Tables

**Supplementary Table S1 List of Countries in Asia as per United Nations Statistical Division**

| **Sub-region Name** | **Country or Area** |
| --- | --- |
| Southern Asia | Afghanistan |
| Western Asia | Armenia |
| Western Asia | Azerbaijan |
| Western Asia | Bahrain |
| Southern Asia | Bangladesh |
| Southern Asia | Bhutan |
| South-eastern Asia | Brunei Darussalam |
| South-eastern Asia | Cambodia |
| Eastern Asia | China |
| Western Asia | Cyprus |
| Eastern Asia | Democratic People's Republic of Korea |
| Western Asia | Georgia |
| Southern Asia | India |
| South-eastern Asia | Indonesia |
| Southern Asia | Iran (Islamic Republic of) |
| Western Asia | Iraq |
| Western Asia | Israel |
| Eastern Asia | Japan |
| Western Asia | Jordan |
| Central Asia | Kazakhstan |
| Western Asia | Kuwait |
| Central Asia | Kyrgyzstan |
| South-eastern Asia | Lao People's Democratic Republic |
| Western Asia | Lebanon |
| South-eastern Asia | Malaysia |
| Southern Asia | Maldives |
| Eastern Asia | Mongolia |
| South-eastern Asia | Myanmar |
| Southern Asia | Nepal |
| Western Asia | Oman |
| Southern Asia | Pakistan |
| South-eastern Asia | Philippines |
| Western Asia | Qatar |
| Eastern Asia | Republic of Korea |
| Western Asia | Saudi Arabia |
| South-eastern Asia | Singapore |
| Southern Asia | Sri Lanka |
| Western Asia | State of Palestine |
| Western Asia | Syrian Arab Republic |
| Central Asia | Tajikistan |
| South-eastern Asia | Thailand |
| South-eastern Asia | Timor-Leste |
| Western Asia | Türkiye |
| Central Asia | Turkmenistan |
| Western Asia | United Arab Emirates |
| Central Asia | Uzbekistan |
| South-eastern Asia | Viet Nam |
| Western Asia | Yemen |

Source: United Nations Statistics Division. Taiwan is not shown separately from China in the list of nations by UN. GBD produces estimates for Taiwan and China, therefore, Taiwan is categorized in Eastern Asia region.

**Supplementary Table S2 List of Cancers, Global Burden of Disease 2019 Study**

| **Sr No** | **Cancer Groups** | **Additional Etiologies available in GBD 2019** |
| --- | --- | --- |
| 1 | Bladder Cancer |  |
| 2 | Brain and central nervous system Cancer |  |
| 3 | Breast Cancer |  |
| 4 | Cervical Cancer |  |
| 5 | Colon and rectum Cancer |  |
| 6 | Esophageal Cancer |  |
| 7 | Gallbladder and biliary tract Cancer |  |
| 8 | Hodgkin lymphoma |  |
| 9 | Kidney Cancer |  |
| 10 | Larynx Cancer |  |
| 11 | Leukemia | 1.     Acute lymphoid leukemia |
|  |  | 2.     Chronic lymphoid leukemia |
|  |  | 3.     Acute myeloid leukemia |
|  |  | 4.     chronic myeloid leukemia |
|  |  | 5.     other leukemia |
| 12 | Lip and oral cavity Cancer |  |
| 13 | Liver Cancer | 1.     Primary Liver Cancer Due to Hepatitis B |
|  |  | 2.     Primary Liver Cancer Due to Hepatitis C |
|  |  | 3.     Primary Liver Cancer Due to Alcohol Use |
|  |  | 4.     Primary Liver Cancer Due to NASH |
|  |  | 5.     Primary Liver Cancer Due to Other Causes |
| 14 | Malignant skin melanoma Cancer |  |
| 15 | Mesothelioma Cancer |  |
| 16 | Multiple myeloma Cancer |  |
| 17 | Nasopharynx Cancer |  |
| 18 | Non-Hodgkin lymphoma |  |
| 19 | Non-melanoma skin Cancer | 1. Squamous-cell Carcinoma 2. Basal-cell Carcinoma |
| 20 | Other malignant neoplasms Cancer |  |
| 21 | Other neoplasms | 1.     Myelodysplastic, myeloproliferative, and other hematopoietic neoplasms |
|  |  | 2.     Benign and in situ intestinal neoplasms |
|  |  | 3.     Benign and in situ cervical and uterine neoplasms |
|  |  | 4.     Other benign and in situ neoplasms |
| 22 | Other pharynx Cancer |  |
| 23 | Ovarian Cancer |  |
| 24 | Pancreatic Cancer |  |
| 25 | Prostate Cancer |  |
| 26 | Stomach Cancer |  |
| 27 | Testicular Cancer |  |
| 28 | Thyroid Cancer |  |
| 29 | Tracheal, bronchus, and lung Cancer |  |
| 30 | Uterine Cancer |  |

**Supplementary Table S3 Cancer Registry Sources Used in GBD 2019 Estimation**

| **Location** | **Registry** | **Years available from registry** | **Years used for incidence** | **Years available for MIR** | **Years used for MIR** |
| --- | --- | --- | --- | --- | --- |
| Bahrain | National Registry | 1998-2012 | 15 | 1998-2012 | 0 |
| Brunei Darussalam | National Registry | 2010-2012 | 3 | 2010-2012 | 0 |
| China | Zhongshan County | 2004, 2006-2007 | 3 | 2004, 2006-2007 | 0 |
| China | Beijing | 1990-2013 | 23 | 1990-2012 | 20 |
| China | Chuzhou District, Huai'an | 2004, 2006-2007 | 3 | 2004, 2006-2007 | 0 |
| China | Hefei | 2010-2013 | 3 | 2010-2012 | 0 |
| China | Ci County | 1990-2012 | 23 | 1990-2012 | 12 |
| China | Qianxi County | 2009, 2011, 2013 | 1 | 2009 | 1 |
| China | Shexian County | 2003-2013 | 10 | 2003-2012 | 6 |
| China | Tongling | 2008-2013 | 5 | 2008-2012 | 2 |
| China | Ma'anshan | 2003-2013 | 10 | 2003-2012 | 7 |
| China | Jiulongpo District, Chongqing | 2004, 2007, 2009, 2011, 2013 | 3 | 2004, 2007, 2009 | 3 |
| China | Changle | 1990-2009, 2011, 2013 | 20 | 1990-2009 | 19 |
| China | Sihui | 1998-2009, 2011, 2013 | 12 | 1998-2009 | 12 |
| China | Xiamen City | 2009, 2011 | 1 | 2009 | 1 |
| China | Jingtai County | 2009, 2011, 2013 | 1 | 2009 | 0 |
| China | Liangzhou District | 2008-2009, 2011, 2013 | 2 | 2008-2009 | 2 |
| China | Wuwei | 2004 | 1 | 2004 | 0 |
| China | Guangzhou City | 2000-2013 | 13 | 2000-2012 | 10 |
| China | Hong Kong Special Administrative Region of China | 1974-2013 | 40 | 1974-2013 | 11 |
| China | Baoding | 2009, 2011, 2013 | 1 | 2009 | 1 |
| China | Liuzhou | 2009-2013 | 4 | 2009-2012 | 1 |
| China | Fusui County | 1990-1997, 2003-2009, 2011, 2013 | 15 | 1990-1997, 2003-2009 | 7 |
| China | Zhuhai | 2010-2012 | 3 | 2010-2012 | 0 |
| China | Jiangmen | 2010-2013 | 3 | 2010-2012 | 0 |
| China | Zhongshan | 1998-2013 | 15 | 1998-2012 | 12 |
| China | Shenzen City | 2004, 2011, 2013 | 1 | 2004 | 0 |
| China | Feixi County | 2009, 2011, 2013 | 1 | 2009 | 1 |
| China | Daoli District, Harbin City | 2005-2009, 2011, 2013 | 5 | 2005-2009 | 5 |
| China | Hai'an County | 2009, 2011, 2013 | 1 | 2009 | 1 |
| China | Haimen | 2003-2013 | 10 | 2003-2012 | 7 |
| China | Huai'an District, Huai'an | 1998-2009, 2013 | 12 | 1998-2009 | 12 |
| China | Huaiyin District, Huai'an | 2009-2013 | 4 | 2009-2012 | 1 |
| China | Zhanggong District | 2009 | 1 | 2009 | 1 |
| China | Dehui | 2009, 2011, 2013 | 1 | 2009 | 1 |
| China | Yanji | 2009, 2011, 2013 | 1 | 2009 | 1 |
| China | Hengdong County | 2009-2012 | 4 | 2009-2012 | 1 |
| China | Anshan | 1998-2013 | 15 | 1998-2012 | 12 |
| China | Dalian City | 1998-2009, 2011 | 12 | 1998-2009 | 12 |
| China | Dandong | 2008-2009, 2011, 2013 | 2 | 2008-2009 | 2 |
| China | Donggang | 2009, 2011 | 1 | 2009 | 1 |
| China | Shenyang City | 2003-2012 | 10 | 2003-2012 | 7 |
| China | Zhuanghe | 2009, 2011, 2013 | 1 | 2009 | 1 |
| China | Yunmeng County | 2009, 2011, 2013 | 1 | 2009 | 1 |
| China | Benxi | 2003-2011, 2013 | 9 | 2003-2011 | 7 |
| China | Xining | 2009, 2011, 2013 | 1 | 2009 | 1 |
| China | Guanyun County | 2004, 2007-2013 | 7 | 2004, 2007-2012 | 2 |
| China | Donghai County | 2004, 2009, 2011, 2013 | 2 | 2004, 2009 | 1 |
| China | Suzhou | 2006-2009, 2011, 2013 | 4 | 2006-2009 | 4 |
| China | Sheyang County | 2008-2013 | 5 | 2008-2012 | 2 |
| China | Qidong County | 1990-2009, 2011 | 20 | 1990-2009 | 20 |
| China | Lianyungang | 2004, 2007-2013 | 7 | 2004, 2007-2012 | 3 |
| China | Jintan District | 2003-2007, 2009, 2011, 2013 | 6 | 2003-2007, 2009 | 6 |
| China | Jinhu County | 2007-2009, 2011, 2013 | 3 | 2007-2009 | 3 |
| China | Ganyu | 2004, 2011, 2013 | 1 | 2004 | 0 |
| China | Jianhu County | 2003-2013 | 10 | 2003-2012 | 7 |
| China | Yueyanglou | 2009-2012 | 4 | 2009-2012 | 0 |
| China | Qidong County | 1983-1997, 2003-2012 | 25 | 1983-1997, 2003-2012 | 0 |
| China | Wuxi | 2006, 2010-2013 | 4 | 2006, 2010-2012 | 1 |
| China | Dafeng | 2003-2009, 2011, 2013 | 7 | 2003-2009 | 7 |
| China | Xuyi County | 2009, 2011, 2013 | 1 | 2009 | 1 |
| China | Yangzhong | 1998-2009, 2011, 2013 | 12 | 1998-2009 | 12 |
| China | Chifeng | 2009, 2011 | 1 | 2009 | 1 |
| China | Feicheng | 1998-2009, 2011, 2013 | 12 | 1998-2009 | 12 |
| China | Changle | 2004, 2006-2007 | 3 | 2004, 2006-2007 | 0 |
| China | Wuhan City | 1990-2012 | 23 | 1990-2012 | 17 |
| China | Nangang District, Harbin City | 1992-2013 | 21 | 1992-2012 | 13 |
| China | Xianju County | 2009-2012 | 4 | 2009-2012 | 1 |
| China | Shangyu | 2009, 2011 | 1 | 2009 | 1 |
| China | Jiaxing | 2000-2013 | 13 | 2000-2012 | 10 |
| China | Jiashan County | 1990-2013 | 23 | 1990-2012 | 20 |
| China | Hangzhou City | 2000-2013 | 13 | 2000-2012 | 10 |
| China | Haining | 1998-2009, 2011, 2013 | 12 | 1998-2009 | 12 |
| China | Shangzhi | 2009, 2011, 2013 | 1 | 2009 | 1 |
| China | Linzhou | 1990-2013 | 23 | 1990-2012 | 20 |
| China | Xiping County | 2009-2013 | 4 | 2009-2012 | 1 |
| China | Yanshi | 2009-2013 | 4 | 2009-2012 | 1 |
| China | Linqu County | 1998-2009, 2011 | 12 | 1998-2009 | 12 |
| China | Taixing | 2004-2005, 2007-2009, 2011, 2013 | 5 | 2004-2005, 2007-2009 | 5 |
| China | Tianjin | 1981-2004, 2011, 2013 | 24 | 1981-2004 | 15 |
| China | Qingyang District, Chengdu | 2009, 2011, 2013 | 1 | 2009 | 1 |
| China | Shanghai | 1975, 1978-2013 | 36 | 1975, 1978-2012 | 20 |
| China | Yanting County | 1998-2013 | 15 | 1998-2012 | 12 |
| China | Wenshang County | 2009, 2011 | 1 | 2009 | 1 |
| China | Ziliujing District | 2009 | 1 | 2009 | 1 |
| China | Yangquan | 2009, 2011, 2013 | 1 | 2009 | 1 |
| China | Yangcheng County | 2003-2009, 2011, 2013 | 7 | 2003-2009 | 7 |
| China | Gejiu | 2004, 2008, 2011, 2013 | 2 | 2004, 2008 | 1 |
| China | Kunes County | 2009 | 1 | 2009 | 1 |
| Cyprus | National Registry | 1998-2012 | 15 | 1998-2012 | 4 |
| Cyprus | South West | 1998-2012 | 15 | 1998-2012 | 0 |
| India | Wardha | 2010-2014 | 5 |  | 0 |
| India | Karunagappally | 1991-2007 | 24 |  | 0 |
| India | Kollam | 2006-2014 | 9 |  | 0 |
| India | Tamil Nadu | 2012-2013 | 2 |  | 0 |
| India | Tripura | 2010-2014 | 5 |  | 0 |
| India | Kamrup Urban | 2005-2014 | 10 |  | 0 |
| India | Silchar | 2005-2006 | 5 |  | 0 |
| India | Delhi | 1990-1996, 1998-2009, 2012 | 17 |  | 0 |
| India | Ahmedabad Urban | 2009-2010, 2012-2013 | 18 |  | 0 |
| India | Bangalore | 1982-2012 | 31 |  | 0 |
| India | Kerala, Urban | | 22 |  | 0 |
| India | Bhopal | 1990-1996, 2004-2013 | 10 |  | 0 |
| India | Barshi Rural | 1988-1992, 2003-2014 | 17 |  | 0 |
| India | Barshi Expanded | 1990-1996, 2009, 2012 | 2 |  | 0 |
| India | Mumbai | 1964-1966, 1968-1975, 1978-2012 | 43 |  | 0 |
| India | Nagpur | 1980-1982, 1993-2002, 2005-2009, 2012-2013 | 20 |  | 0 |
| India | Pune | 1973-1982, 1993-2013 | 31 |  | 0 |
| India | Manipur Excl Imphal West | 2009-2010, 2012-2014 | 7 |  | 0 |
| India | Aurangabad | 2005-2010, 2012-2014 | 9 |  | 0 |
| India | S.A.S Nagar District | 2013-2014 | 2 |  | 0 |
| India | Patiala District | 2012-2014 | 3 |  | 0 |
| India | Ahmedabad Rural | 2009-2010 | 16 |  | 0 |
| India | Kolkata | 2005-2009, 2012 | 6 |  | 0 |
| India | Chennai | 1982-2013 | 32 |  | 0 |
| India | Chandigarh Union Territory | 2013-2014 | 2 |  | 0 |
| India | Pasighat | 2012-2014 | 3 |  | 0 |
| India | Naharlagun Excl Papum Pare | 2012-2014 | 3 |  | 0 |
| India | Papum Pare | 2012-2014 | 3 |  | 0 |
| India | Cachar | 2007-2014 | 8 |  | 0 |
| India | Sikkim | 2003-2014 | 12 |  | 0 |
| India | Ahmedabad | 1983-1987, 1993-1997, 2004-2005, 2008-2011 | 16 |  | 0 |
| India | Trivandrum | 1991-2002, 2005-2014 | 22 |  | 0 |
| India | Dibrugarh | 2005-2014 | 10 |  | 0 |
| India | Imphal | 2005-2006, 2009-2010, 2012-2014 | 5 |  | 0 |
| India | Aizawl | 2005-2008, 2012-2014 | 6 |  | 0 |
| India | Sangrur District | 2013-2014 | 2 |  | 0 |
| India | Manipur | 2006-2010 | 5 |  | 0 |
| India | Mizoram Excl Aizawl | 2005-2006, 2012-2014 | 5 |  | 0 |
| India | Mansa District | 2013-2014 | 2 |  | 0 |
| India | Nagaland | 2010, 2012-2014 | 4 |  | 0 |
| India | Mizoram | 2003-2012 | 10 |  | 0 |
| India | Meghalaya | 2010-2014 | 5 |  | 0 |
| India | Dindigul Ambilikkai | 2003-2013 | 11 |  | 0 |
| Iran (Islamic Republic of) | Bushehr | 2001-2010 | 10 | 2001-2010 | 0 |
| Iran (Islamic Republic of) | Alborz | 2002-2010 | 9 | 2002-2010 | 0 |
| Iran (Islamic Republic of) | Khorasan-e-Razavi | 2000-2010 | 11 | 2000-2010 | 0 |
| Iran (Islamic Republic of) | West Azarbayejan | 2000-2010 | 11 | 2000-2010 | 0 |
| Iran (Islamic Republic of) | East Azarbayejan | 2000-2010 | 11 | 2000-2010 | 0 |
| Iran (Islamic Republic of) | Ardebil | 2000-2010 | 11 | 2000-2010 | 0 |
| Iran (Islamic Republic of) | Golestan | 1996-2000, 2004-2011 | 12 | 1996-2000, 2005-2011 | 0 |
| Iran (Islamic Republic of) | North Khorasan | 2000, 2002-2010 | 10 | 2000, 2002-2010 | 0 |
| Iran (Islamic Republic of) | National Registry | 2003-2007 | 5 | 2003-2007 | 0 |
| Iran (Islamic Republic of) | South Khorasan | 2001-2010 | 10 | 2001-2010 | 0 |
| Iran (Islamic Republic of) | Khuzestan | 2000-2010 | 11 | 2000-2010 | 0 |
| Iran (Islamic Republic of) | Kohgiluyeh and Boyer-Ahmad | 2000-2010 | 11 | 2000-2010 | 0 |
| Iran (Islamic Republic of) | Kurdistan | 2000-2010 | 11 | 2000-2010 | 0 |
| Iran (Islamic Republic of) | Lorestan | 2000-2010 | 11 | 2000-2010 | 0 |
| Iran (Islamic Republic of) | Markazi | 2001-2010 | 10 | 2001-2010 | 0 |
| Iran (Islamic Republic of) | Mazandaran | 2000-2010 | 11 | 2000-2010 | 0 |
| Iran (Islamic Republic of) | Ardabil | 1985, 1996-1999, 2006-2008 | 8 | 1985, 1996-1999, 2006-2008 | 0 |
| Iran (Islamic Republic of) | Qom | 2000-2010 | 11 | 2000-2010 | 0 |
| Iran (Islamic Republic of) | Semnan | 2000-2010 | 11 | 2000-2010 | 0 |
| Iran (Islamic Republic of) | Sistan and Baluchistan | 2001-2010 | 10 | 2001-2010 | 0 |
| Iran (Islamic Republic of) | Tehran | 2000-2010 | 11 | 2000-2010 | 0 |
| Iran (Islamic Republic of) | Yazd | 2000-2010 | 11 | 2000-2010 | 0 |
| Iran (Islamic Republic of) | Zanjan | 2000-2010 | 11 | 2000-2010 | 0 |
| Iran (Islamic Republic of) | Qazvin | 2000-2010 | 11 | 2000-2010 | 0 |
| Iran (Islamic Republic of) | Kermanshah | 2000-2010 | 11 | 2000-2010 | 0 |
| Iran (Islamic Republic of) | Kerman | 2000-2010 | 11 | 2000-2010 | 0 |
| Iran (Islamic Republic of) | Isfahan | 2000-2010 | 11 | 2000-2010 | 0 |
| Iran (Islamic Republic of) | Ilam | 2000-2010 | 11 | 2000-2010 | 0 |
| Iran (Islamic Republic of) | Hormozgan | 2001-2010 | 10 | 2001-2010 | 0 |
| Iran (Islamic Republic of) | Hamadan | 2000-2010 | 11 | 2000-2010 | 0 |
| Iran (Islamic Republic of) | Golestan | 2000-2010 | 11 | 2000-2010 | 0 |
| Iran (Islamic Republic of) | Gilan | 2000-2010 | 11 | 2000-2010 | 0 |
| Iran (Islamic Republic of) | Fars | 2000-2010 | 11 | 2000-2010 | 0 |
| Iran (Islamic Republic of) | Chahar Mahaal and Bakhtiari | 2000-2010 | 11 | 2000-2010 | 0 |
| Iraq | National Registry | 2007, 2009, 2011 | 3 | 2007, 2009, 2011 | 0 |
| Israel | National Registry | 1960-1963, 1990-2012 | 27 | 1960-1963, 1990-2012 | 0 |
| Japan | Tochigi | 2008-2012 | 5 | 2008-2012 | 0 |
| Japan | Nagasaki | 1973-2012 | 40 | 1973-2012 | 0 |
| Japan | Saga | 1984-1986, 1988-1997, 2003-2007 | 18 | 1984-1986, 1988-1997, 2003-2007 | 0 |
| Japan | Fukuoka | 1974-1975 | 2 | 1974-1975 | 0 |
| Japan | Hiroshima | 1978-2000, 2003-2012 | 33 | 1978-2000, 2003-2012 | 0 |
| Japan | Okayama | 1966, 1969 | 2 | 1966, 1969 | 0 |
| Japan | Osaka | 1963-2002, 2008-2012 | 45 | 1963-2002, 2008-2012 | 0 |
| Japan | Aichi | 1998-2012 | 15 | 1998-2012 | 1 |
| Japan | Fukui | 1998-2012 | 15 | 1998-2012 | 0 |
| Japan | Niigata | 2003-2012 | 10 | 2003-2012 | 0 |
| Japan | Miyagi | 1959-1960, 1962-1964, 1968-1971, 1973-2010 | 47 | 1959-1960, 1962-1964, 1968-1971, 1973-2010 | 0 |
| Japan | Yamagata | 1983-2002, 2008-2012 | 25 | 1983-2002, 2008-2012 | 0 |
| Japan | National Registry | 1975-2010 | 36 | 1958-2013 | 36 |
| Jordan | National Registry | 1996-1998, 2001-2008 | 8 | 2001-2008 | 0 |
| Kuwait | National Registry | 1979-2012 | 34 | 1979-2012 | 0 |
| Kyrgyzstan | National Registry | 1986-1987 | 2 | 1986-1987 | 0 |
| Lebanon | National Registry | 1998, 2005-2007 | 4 | 1998, 2005-2007 | 0 |
| Malaysia | National Registry | 2003 | 1 | 2003 | 0 |
| Malaysia | Penang | 1998-2002, 2004-2010 | 12 | 1998-2002, 2004-2010 | 0 |
| Malaysia | Sarawak | 1998-2002 | 5 | 1998-2002 | 0 |
| Mongolia | National Registry | 2003-2007 | 5 | 2003-2007 | 0 |
| Oman | National Registry | 1993-2013 | 21 | 1993-2013 | 0 |
| Pakistan | Karachi | 1995-2002 | 8 | 1995-2002 | 0 |
| Pakistan | South Karachi | 1995-2002 | 8 | 1995-2002 | 0 |
| Philippines | Rizal | 1978-1987, 1993-1997 | 15 | 1978-1987, 1993-1997 | 0 |
| Philippines | Manila | 1983-2002 | 20 | 1983-2002 | 0 |
| Qatar | National Registry | 2003-2012 | 10 | 2003-2012 | 0 |
| Republic of Korea | Daejeon | 1998-2012 | 15 | 1998-2012 | 0 |
| Republic of Korea | Busan | 1996-2012 | 17 | 1996-2012 | 0 |
| Republic of Korea | Daegu | 1997-2012 | 16 | 1997-2012 | 0 |
| Republic of Korea | Gwangju | 1998-2012 | 15 | 1998-2012 | 0 |
| Republic of Korea | Incheon | 1998-2012 | 15 | 1998-2012 | 0 |
| Republic of Korea | Jejudo | 2000-2002, 2004-2012 | 12 | 2000-2002, 2004-2012 | 0 |
| Republic of Korea | National Registry | 1999-2012 | 14 | 1999-2012 | 0 |
| Republic of Korea | Seoul | 1993-2012 | 20 | 1993-2012 | 0 |
| Republic of Korea | Ulsan | 1999-2012 | 14 | 1999-2012 | 0 |
| Republic of Korea | Kangwha County | 1986-1997 | 12 | 1986-1997 | 0 |
| Saudi Arabia | Riyadh | 1994-2012 | 19 | 1994-2012 | 0 |
| Saudi Arabia | National Registry | 1994-1996, 2006-2012 | 10 | 1994-1996, 2006-2012 | 7 |
| Singapore | National Registry | 1950-1961, 1968-2015 | 60 | 1950-1961, 1968-2015 | 0 |
| Sri Lanka | National Registry | 2001-2005 | 5 | 2001-2005 | 0 |
| State of Palestine | West Bank | 2010-2011 | 2 | 2010-2011 | 0 |
| Taiwan (Province of China) | National Registry | 1980-2009 | 30 | 1980-2009 | 28 |
| Thailand | Udon Thani | 2001-2003 | 3 | 2001-2003 | 0 |
| Thailand | Songkhla | 1993-1996, 1998-2012 | 19 | 1993-1996, 1998-2012 | 0 |
| Thailand | Lopburi Province | 2009-2012 | 4 | 2009-2012 | 0 |
| Thailand | Bangkok | 1995-1997, 2001-2012 | 15 | 1995-1997, 2001-2012 | 0 |
| Thailand | Chiang Mai | 1983-2012 | 30 | 1983-2012 | 0 |
| Thailand | Chonburi | 2001-2011 | 11 | 2001-2011 | 0 |
| Thailand | Surat Thani | 2001-2003 | 3 | 2001-2003 | 0 |
| Thailand | Khon Kaen | 1985-2012 | 22 | 1988-1997, 2001-2012 | 0 |
| Thailand | Lop Buri | 2001-2003 | 3 | 2001-2003 | 0 |
| Thailand | Nakhon Phanom | 2001-2003 | 3 | 2001-2003 | 0 |
| Thailand | Prachuap Khiri | 2001-2003 | 3 | 2001-2003 | 0 |
| Thailand | Rayong | 2001-2003 | 3 | 2001-2003 | 0 |
| Thailand | Lampang | 1993-2013 | 21 | 1993-2013 | 0 |
| Thailand | Ubon Ratchathani | 2001-2003 | 3 | 2001-2003 | 0 |
| Turkey | Nine Provinces Combined (Ankara, Antalya, Bursa, Edirne, Erzurum, Eskisehir, Izmir, Samsun, Trabzon) | 2009-2014 | 6 | 2009-2014 | 0 |
| Turkey | Izmir | 1993-1996, 1998-2012 | 15 | 1998-2012 | 0 |
| Turkey | Eskisehir | 2002-2005, 2008-2012 | 9 | 2002-2005, 2008-2012 | 0 |
| Turkey | Erzurum | 2002-2005, 2010-2012 | 7 | 2002-2005, 2010-2012 | 0 |
| Turkey | Edirne | 2002-2012 | 11 | 2002-2012 | 0 |
| Turkey | Antalya | 1998-2012 | 15 | 1998-2012 | 0 |
| Turkey | Ankara | 2002-2005 | 4 | 2002-2005 | 0 |
| Turkey | Bursa | 2008-2012 | 5 | 2008-2012 | 0 |
| Turkey | Trabzon | 2002-2003, 2005-2012 | 10 | 2002-2003, 2005-2012 | 0 |
| Turkey | Samsun | 2002-2005, 2008-2012 | 9 | 2002-2005, 2008-2012 | 0 |
| Turkey | 8 Registries Combined (Ankara, Antalya, Bursa, Edirne, Erzurum, Izmir, Samsun, Trabzon) | 1992-2012 | 21 | 1992-2012 | 0 |
| Viet Nam | Ho Chi Minh | 1995-1998, 2009-2012 | 8 | 1995-1998, 2009-2012 | 0 |
| Viet Nam | Hanoi | 1991-1997 | 7 | 1991-1997 | 0 |
| Yemen | Aden | 1997-2011 | 5 | 2007-2011 | 0 |

Footnote: Cancer registry incidence data is only used for estimating MIRs, column “Years used for MIR” if both incidence and mortality data are available.

**Supplementary Table S4 Risk-outcome Pairs Included in GBD 2019 Study**

| **Risk Factor** | **Cause** |
| --- | --- |
| Alcohol use | Breast cancer |
| Alcohol use | Colon and rectum cancer |
| Alcohol use | Esophageal cancer |
| Alcohol use | Larynx cancer |
| Alcohol use | Lip and oral cavity cancer |
| Alcohol use | Liver cancer |
| Alcohol use | Nasopharynx cancer |
| Alcohol use | Other pharynx cancer |
| Ambient particulate matter pollution | Tracheal, bronchus, and lung cancer |
| Chewing tobacco | Esophageal cancer |
| Chewing tobacco | Lip and oral cavity cancer |
| Diet high in processed meat | Colon and rectum cancer |
| Diet high in red meat | Breast cancer |
| Diet high in red meat | Colon and rectum cancer |
| Diet high in sodium | Stomach cancer |
| Diet low in calcium | Colon and rectum cancer |
| Diet low in fiber | Colon and rectum cancer |
| Diet low in fruits | Esophageal cancer |
| Diet low in fruits | Tracheal, bronchus, and lung cancer |
| Diet low in milk | Colon and rectum cancer |
| Diet low in vegetables | Esophageal cancer |
| Diet low in whole grains | Colon and rectum cancer |
| Drug use | Liver cancer |
| High body-mass index | Breast cancer |
| High body-mass index | Colon and rectum cancer |
| High body-mass index | Esophageal cancer |
| High body-mass index | Gallbladder and biliary tract cancer |
| High body-mass index | Kidney cancer |
| High body-mass index | Leukemia |
| High body-mass index | Liver cancer |
| High body-mass index | Multiple myeloma |
| High body-mass index | Non-Hodgkin lymphoma |
| High body-mass index | Ovarian cancer |
| High body-mass index | Pancreatic cancer |
| High body-mass index | Thyroid cancer |
| High body-mass index | Uterine cancer |
| High fasting plasma glucose | Bladder cancer |
| High fasting plasma glucose | Breast cancer |
| High fasting plasma glucose | Colon and rectum cancer |
| High fasting plasma glucose | Liver cancer |
| High fasting plasma glucose | Ovarian cancer |
| High fasting plasma glucose | Pancreatic cancer |
| High fasting plasma glucose | Tracheal, bronchus, and lung cancer |
| Household air pollution from solid fuels | Tracheal, bronchus, and lung cancer |
| Low physical activity | Breast cancer |
| Low physical activity | Colon and rectum cancer |
| Occupational exposure to arsenic | Tracheal, bronchus, and lung cancer |
| Occupational exposure to asbestos | Larynx cancer |
| Occupational exposure to asbestos | Mesothelioma |
| Occupational exposure to asbestos | Ovarian cancer |
| Occupational exposure to asbestos | Tracheal, bronchus, and lung cancer |
| Occupational exposure to benzene | Leukemia |
| Occupational exposure to beryllium | Tracheal, bronchus, and lung cancer |
| Occupational exposure to cadmium | Tracheal, bronchus, and lung cancer |
| Occupational exposure to chromium | Tracheal, bronchus, and lung cancer |
| Occupational exposure to diesel engine exhaust | Tracheal, bronchus, and lung cancer |
| Occupational exposure to formaldehyde | Leukemia |
| Occupational exposure to formaldehyde | Nasopharynx cancer |
| Occupational exposure to nickel | Tracheal, bronchus, and lung cancer |
| Occupational exposure to polycyclic aromatic hydrocarbons | Tracheal, bronchus, and lung cancer |
| Occupational exposure to silica | Tracheal, bronchus, and lung cancer |
| Occupational exposure to sulfuric acid | Larynx cancer |
| Occupational exposure to trichloroethylene | Kidney cancer |
| Residential radon | Tracheal, bronchus, and lung cancer |
| Secondhand smoke | Breast cancer |
| Secondhand smoke | Tracheal, bronchus, and lung cancer |
| Smoking | Bladder cancer |
| Smoking | Breast cancer |
| Smoking | Cervical cancer |
| Smoking | Colon and rectum cancer |
| Smoking | Esophageal cancer |
| Smoking | Kidney cancer |
| Smoking | Larynx cancer |
| Smoking | Leukemia |
| Smoking | Lip and oral cavity cancer |
| Smoking | Liver cancer |
| Smoking | Nasopharynx cancer |
| Smoking | Other pharynx cancer |
| Smoking | Pancreatic cancer |
| Smoking | Prostate cancer |
| Smoking | Stomach cancer |
| Smoking | Tracheal, bronchus, and lung cancer |
| Unsafe sex | Cervical cancer |

**Supplementary Table S5 Theoretical Minimum Risk Exposure Level (TMREL) by Risk Factors and Cancers**

| **Type of Risk Factor** | **Risk Factor** | **Exposure Definition** | **TMREL** | **Cancers** |
| --- | --- | --- | --- | --- |
| Environmental/Occupational Risks | Ambient particulate matter pollution | Annual average daily exposure to outdoor air concentrations of particulate matter with an aerodynamic diameter of ≤2·5 µm (PM2·5), measured in μg/m³ | Joint theoretical minimum risk exposure level for both household and ambient particulate matter pollution is a uniform distribution between 2·4 and 5·9 μg/m³, with burden attributed proportionally between household and particulate matter pollution on the basis of source of PM2·5 exposure in excess of theoretical minimum risk exposure level | Tracheal, bronchus, and lung cancer |
| Environmental/Occupational Risks | Household air pollution from solid fuels | Individual exposure to PM2·5 due to use of solid cooking fuel | See ambient particulate matter pollution | Tracheal, bronchus, and lung cancer |
| Environmental/Occupational Risks | Residential radon | Average daily exposure to indoor air radon levels measured in becquerels (radon disintegrations per second) per cubic metre (Bq/m³) | 10 Bq/m³, corresponding to the outdoor concentration of radon | Tracheal, bronchus, and lung cancer |
| Environmental/Occupational Risks | Occupational exposure to asbestos | Proportion of the population with cumulative lifetime exposure to occupational asbestos | No occupational exposure to asbestos | Larynx cancer, Tracheal, bronchus, and lung cancer; Ovarian cancer; Mesothelioma |
| Environmental/Occupational Risks | Occupational exposure to arsenic | Proportion of the population ever exposed to arsenic at work or through their occupation | No occupational exposure to arsenic | Tracheal, bronchus, and lung cancer |
| Environmental/Occupational Risks | Occupational exposure to benzene | Proportion of the population ever exposed to benzene at work or through their occupation | No occupational exposure to benzene | Leukemia |
| Environmental/Occupational Risks | Occupational exposure to beryllium | Proportion of the population ever exposed to beryllium at work or through their occupation | No occupational exposure to beryllium | Tracheal, bronchus, and lung cancer |
| Environmental/Occupational Risks | Occupational exposure to cadmium | Proportion of the population ever exposed to cadmium at work or through their occupation | No occupational exposure to cadmium | Tracheal, bronchus, and lung cancer |
| Environmental/Occupational Risks | Occupational exposure to chromium | Proportion of the population ever exposed to chromium at work or through their occupation | No occupational exposure to chromium | Tracheal, bronchus, and lung cancer |
| Environmental/Occupational Risks | Occupational exposure to diesel engine exhaust | Proportion of the population ever exposed to diesel engine exhaust at work or through their occupation | No occupational exposure to diesel engine exhaust | Tracheal, bronchus, and lung cancer |
| Environmental/Occupational Risks | Occupational exposure to formaldehyde | Proportion of the population ever exposed to formaldehyde at work or through their occupation | No occupational exposure to formaldehyde | Nasopharynx cancer; Leukemia |
| Environmental/Occupational Risks | Occupational exposure to nickel | Proportion of the population ever exposed to nickel at work or through their occupation | No occupational exposure to nickel | Tracheal, bronchus, and lung cancer |
| Environmental/Occupational Risks | Occupational exposure to polycyclic aromatic hydrocarbons | Proportion of the population ever exposed to polycyclic aromatic hydrocarbons at work or through their occupation | No occupational exposure to polycyclic aromatic hydrocarbons | Tracheal, bronchus, and lung cancer |
| Environmental/Occupational Risks | Occupational exposure to silica | Proportion of the population ever exposed to silica at work or through their occupation | No occupational exposure to silica | Tracheal, bronchus, and lung cancer |
| Environmental/Occupational Risks | Occupational exposure to sulfuric acid | Proportion of the population ever exposed to sulphuric acid at work or through their occupation | No occupational exposure to sulfuric acid | Larynx cancer |
| Environmental/Occupational Risks | Occupational exposure to trichloroethylene | Proportion of the population ever exposed to trichloroethylene at work or through their occupation | No occupational exposure to trichloroethylene | Kidney cancer |
| Behavioural Risks | Smoking | Prevalence of current use of any smoked tobacco product and prevalence of former use of any smoked tobacco product; among current smokers, cigarette equivalents smoked per smoker per day and cumulative pack-years of exposure; among former smokers, number of years since quitting | All individuals are lifelong non-smokers | Lip and oral cavity cancer;  Nasopharynx cancer; Other pharynx cancer;  Oesophageal cancer; Stomach cancer; Colon and rectum cancer;  Liver cancer; Pancreatic cancer; Larynx cancer; Tracheal, bronchus, and lung cancer; Breast cancer; Cervical cancer; Prostate cancer; Kidney cancer; Bladder cancer; Leukemia |
| Behavioural Risks | Chewing tobacco | Current use of any chewing tobacco product | All individuals are lifelong non-users of chewing tobacco products | Lip and oral cavity cancer;  Oesophageal cancer |
| Behavioural Risks | Secondhand smoke | Average daily exposure to air particulate matter from second-hand smoke with an aerodynamic diameter smaller than 2·5 µg, measured in µg/m³, among non-smokers | No second-hand smoke exposure | Tracheal, bronchus, and lung cancer; Breast cancer |
| Behavioural Risks | Alcohol use | Average daily alcohol consumption of pure alcohol (measured in g per day) in current drinkers who had consumed alcohol during the past 12 months | Estimated distribution 0–10 g per day | Lip and oral cavity cancer;  Nasopharynx cancer; Other pharynx cancer;  Oesophageal cancer; Colon and rectum cancer;  Liver cancer;  Larynx cancer; Breast cancer |
| Behavioural Risks | Drug use | Proportion of the population dependent upon opioids, cannabis, cocaine, or amphetamines; proportion of the population who have ever injected drugs | No drug use | Liver Cancer |
| Behavioural Risks | Diet low in fruits | Average daily consumption of fruits (fresh, frozen, cooked, canned, or dried, excluding fruit juices and salted or pickled fruits) | Consumption of fruit 200–300 g per day | Oesophageal cancer; Tracheal, bronchus, and lung cancer |
| Behavioural Risks | Diet low in vegetables | Average daily consumption of vegetables (fresh, frozen, cooked, canned, or dried, excluding legumes and salted or pickled vegetables, juices, nuts and seeds, and starchy vegetables such as potatoes or corn) | Consumption of vegetables 290–430 g per day | Oesophageal cancer |
| Behavioural Risks | Diet low in whole grains | Average daily consumption of whole grains (bran, germ, and endosperm in their natural proportion) from breakfast cereals, bread, rice, pasta, biscuits, muffins, tortillas, pancakes, and other sources | Consumption of whole grains 100–150 g per day | Colon and rectum cancer |
| Behavioural Risks | Diet low in milk | Average daily consumption of milk, including nonfat, low-fat, and full-fat milk, excluding soy milk and other plant derivatives | Consumption of milk 350–520 g per day | Colon and rectum cancer |
| Behavioural Risks | Diet high in red meat | Average daily consumption of red meat (beef, pork, lamb, and goat but excluding poultry, fish, eggs, and all processed meats) | Consumption of red meat 18–27 g per day | Colon and rectum cancer;  Breast Cancer |
| Behavioural Risks | Diet high in processed meat | Average daily consumption of meat preserved by smoking, curing, salting, or addition of chemical preservatives | Consumption of processed meat 0–4 g per day | Colon and rectum cancer |
| Behavioural Risks | Diet low in fibre | Average daily intake of fibre from all sources including fruits, vegetables, grains, legumes, and pulses | Consumption of fibre 19–28 g per day | Colon and rectum cancer |
| Behavioural Risks | Diet low in calcium | Average daily intake of calcium from all sources, including milk, yogurt, and cheese | Consumption of calcium 1·0–1·5 g per day | Colon and rectum cancer |
| Behavioural Risks | Diet high in sodium | 24-h urinary sodium measured in g per day | 24-h urinary sodium 1–5 g per day | Stomach cancer |
| Behavioural Risks | Unsafe sex | Proportion of the population with exposure to sexual encounters that convey the risk of disease | No exposure to disease-causing pathogen through sex | Cervical cancer |
| Behavioural Risks | Low physical activity | Average weekly physical activity at work, home, transport-related and recreational measured by MET min per week | All adults experience 3000–4500 MET min per week | Colon and rectum cancer;  Breast cancer |
| Metabolic Risks | High fasting plasma glucose | Serum fasting plasma glucose measured in mmol/L | 4·8–5·4 mmol/L | Colon and rectum cancer;  Pancreatic cancer; Tracheal, bronchus, and lung cancer; Breast cancer; Ovarian cancer; Bladder cancer |
| Metabolic Risks | High body-mass index | Body-mass index, measured in kg/m² | 20–25 kg/m² | Oesophageal cancer; Colon and rectum cancer;  Liver cancer; Gallbladder and biliary tract cancer; Pancreatic cancer; Breast cancer; Uterine cancer; Ovarian cancer; Kidney cancer; Thyroid cancer;  Non-Hodgkin lymphoma;  Multiple myeloma; Leukemia |

**Supplementary Table S6 Ranking of Total Cancer Absolute Disability-Adjusted Life Years (DALYs) in 2019 among the 22 Level 2**

**Categories of Disease in the Global Burden of Disease (GBD) Study**

|  | **Absolute DALYs, millions (95% UI)** | |  | **Rank** | |
| --- | --- | --- | --- | --- | --- |
| **GBD level 2 Cause of Disease or Injury** | **1990** | **2019** | **Percent Change, 1990-2019** | **1990** | **2019** |
| Cardiovascular diseases | 144.9  [135.8 - 153.9] | 243.0  [224.6 - 260.7] | 67.7  [52.2 to 82.2] | 3 | 1↑ |
| **Total Cancers** | **86.2**  **[76.8 - 92.9]** | **144.7**  **[132.7 – 156.5]** | **67.9**  **[51.1 to 88.4]** | **7** | **2**↑ |
| Maternal and neonatal disorders | 188.6  [176.1 - 202.3] | 102.8  [89.9 - 118.4] | -45.5  [-52.8 to -36.9] | 2 | 3↓ |
| Musculoskeletal disorders | 45.5  [32.9 - 60.2] | 86.3  [62.2 – 114.0] | 89.5  [83.3 to 96.4] | 11 | 4↑ |
| Other non-communicable diseases | 87.7  [68.3 - 108.3] | 78.9  [63.1 - 99.9] | -10.0  [-28.2 to 13.8] | 6 | 5↑ |
| Respiratory infections and tuberculosis | 198.2  [181.2 - 218.6] | 75.4  [68.6 - 82.9] | -62.0  [-66.9 to -56.8] | 1 | 6↓ |
| Chronic respiratory diseases | 60.5  [50.9 - 65.8] | 71.0  [64.7 - 77.6] | 17.3  [5.5 to 38.7] | 9 | 7↑ |
| Mental disorders | 43.9  [32.5 - 57.2] | 69.0  [51 - 89.7] | 57.3  [53.7 to 60.7] | 12 | 8↑ |
| Diabetes and kidney diseases | 28.2  [25.4 - 31.4] | 64.9  [56.9 - 73.6] | 129.8  [115.2 to 144.7] | 18 | 9↑ |
| Unintentional injuries | 79.6  [71.1 - 86.9] | 60.4  [51.5 - 68.9] | -24.2  [-32.7 to -13.3] | 8 | 10↓ |
| Neurological disorders | 30.2  [16.0 - 50.2] | 53.6  [29.2 - 89.8] | 77.8  [62.5 to 104.4] | 17 | 11↑ |
| Digestive diseases | 42.8  [39.3 - 46.1] | 48.1  [43.8 - 52.9] | 12.4  [2.7 to 26.6] | 13 | 12↑ |
| Transport injuries | 42.7  [39.5 - 48.1] | 47.2  [41.5 - 52.2] | 10.5  [-3.6 to 21.9] | 14 | 13↑ |
| Sense organ diseases | 22.4  [15.6 - 31.3] | 43.2  [29.5 - 60.7] | 92.5  [84.2 to 99.3] | 19 | 14↑ |
| Enteric infections | 116.6  [96.7 - 138.6] | 42.0  [31.9 - 54.2] | -64.0  [-71.1 to -55.0] | 4 | 15↓ |
| Self-harm and interpersonal violence | 37.3  [33.5 - 40.3] | 34.9  [32 - 38.1] | -6.4  [-14.6 to 3.5] | 15 | 16↓ |
| Nutritional deficiencies | 57.5  [43.4 - 74.7] | 28.8  [20.5 - 39.1] | -49.9  [-64 to -34.3] | 10 | 17↓ |
| Skin and subcutaneous diseases | 18.4  [12.2 - 26.8] | 25.0  [16.4 - 37.2] | 35.6  [32.4 to 38.6] | 20 | 18↑ |
| Other infectious diseases | 104.4  [79.1 - 141.7] | 22.0  [18.6 - 26.7] | -78.9  [-83.9 to -73.2] | 5 | 19↓ |
| Substance use disorders | 10.7  [8.3 - 13.2] | 14.6  [11.3 - 18.4] | 37.2  [30.4 to 43.8] | 21 | 20↑ |
| Neglected tropical diseases and malaria | 33.7  [22.2 - 52.3] | 11.2  [8.2 - 15.1] | -66.7  [-77.3 to -54.3] | 16 | 21↓ |
| HIV/AIDS and sexually transmitted infections | 4.4  [2.0 - 8.3] | 10.0  [8.2 - 13.1] | 128.4  [47.0 to 324.4] | 22 | 22= |

DALYs = Disability-adjusted Life Years. The GBD study organized diseases and injuries into a hierarchy that was mutually exclusive and collectively exhaustive. More details of this hierarchy were previously published. The other non-communicable diseases include congenital birth defects; urinary diseases and male infertility; gynecological diseases; hemoglobinopathies and haemolytic anemias; endocrine, metabolic, blood, and immune disorders; oral disorders; and sudden infant death syndrome. The other infectious diseases include meningitis; encephalitis; diphtheria; whooping cough; tetanus; measles; varicella and herpes zoster; acute hepatitis; and other unspecified infectious diseases. UI = uncertainty interval.

**Supplementary Table S7 Absolute Count of Cancer Burden by Sex in 2019 in Asia**

|  | **Incidence (in thousands)** | | | **Deaths (in thousands)** | | | **DALYs (in thousands)** | | |
| --- | --- | --- | --- | --- | --- | --- | --- | --- | --- |
| **Cancer Type** | **Both Sexes** | **Males** | **Females** | **Both Sexes** | **Males** | **Females** | **Both Sexes** | **Males** | **Females** |
| Total Cancers | 9377.4  [8592.0 – 10203.5] | 5149.2  [4576.6 – 5821.6] | 4228.2  [3794.6 – 4702.1] | 5576.4  [5079.1 – 6049.2] | 3276.0  [2900.4 – 3677.6] | 2300.4  [2040.1 – 2565.0] | 144736.7  [132723.7 – 156480.6] | 83790.7  [74273.6 – 94143.1] | 60946.0  [54550.0 – 67799.8] |
| Bladder | 209.8  [188.9 – 231.5] | 165.7  [146.4 – 186.4] | 44.1  [38.6 – 49.8] | 94.3  [85.6 – 103.3] | 70.2  [63.0 – 77.9] | 24.1  [20.7 – 27.0] | 1900.4  [1734.9 – 2083.0] | 1443.8  [1297.6 – 1616.1] | 456.7  [404.4 – 511.8] |
| Brain and central nervous system | 188.7  [142.4 – 214.5] | 99.9  [66.2 – 122.4] | 88.8  [66.8 – 105.3] | 129.5  [97.0 – 146.9] | 73.1  [47.2 – 89.4] | 56.4  [41.2 – 66.8] | 4776.4  [3639.0 – 5463.4] | 2767.9  [1817.8 – 3404.5] | 2008.5  [1521.0 – 2385.8] |
| Breast | 927.8  [828.4 – 1039.3] | 12.9  [10.8 – 15.1] | 914.9  [815.8 – 1025.5] | 344.5  [308.1 – 382.2] | 6.7  [5.7 – 7.7] | 337.8  [301.5 – 375.3] | 11070.3  [9937.3 – 12316.2] | 181.0  [155.0 – 208.9] | 10889.3  [9751.4 – 12107.3] |
| Cervical | 297.4  [238.3 – 343.9] |  | 297.4  [238.3 – 343.9] | 146.5  [118.9 – 170.8] |  | 146.5  [118.9 – 170.8] | 4693.9  [3779.6 – 5446.2] |  | 4693.9  [3779.6 – 5446.2] |
| Colon and rectum | 1123.7  [1013.5 – 1242.7] | 677.5  [591.9 – 779.1] | 446.1  [388.2 – 505.6] | 560.4  [504.6 – 609.8] | 321.2  [283.4 – 359.5] | 239.2  [207.8 – 268.0] | 13366.6  [12116.8 – 14566.7] | 7970.5  [7027.4 – 8969.7] | 5396.0  [4757.2 – 6051.6] |
| Esophageal | 391.2  [332.6 – 449.3] | 284.6  [233.3 – 339.6] | 106.6  [83.6 – 125.4] | 361.1  [307.6 – 411.8] | 266.2  [218.5 – 315.0] | 94.9  [76.4 – 111.5] | 8409.0  [7201.3 – 9643.9] | 6379.0  [5255.6 – 7615.3] | 2030.0  [1698.3 – 2364.6] |
| Gallbladder and biliary tract | 124.2  [96.0 – 138.0] | 56.7  [41.4 – 64.9] | 67.5  [48.5 – 78.1] | 111.3  [86.7 – 123.3] | 50.2  [38.1 – 56.8] | 61.1  [43.7 – 70.2] | 2383.1  [1897.6 – 2639.7] | 1098.2  [859.9 – 1251.8] | 1284.9  [922.2 – 1497.9] |
| Hodgkin lymphoma | 38.0  [31.5 – 43.0] | 24.9  [19.5 – 29.8] | 13.1  [10.1 – 15.6] | 13.3  [11.1 – 16.6] | 9.0  [7.0 – 12.0] | 4.2  [3.3 – 5.5] | 571.7  [481.3 – 712.3] | 393.4  [303.4 – 522.1] | 178.3  [140.9 – 238.7] |
| Kidney | 129.8  [116.4 – 145.7] | 87.1  [76.0 – 100.0] | 42.8  [37.8 – 48.8] | 59.7  [53.7 – 66.7] | 40.3  [35.7 – 45.6] | 19.3  [16.9 – 22.1] | 1558.9  [1407.6 – 1747.2] | 1075.6  [944.8 – 1222.7] | 483.3  [429.8 – 554.2] |
| Larynx | 117.3  [105.7 – 131.0] | 100.2  [88.9 – 112.9] | 17.1  [15.3 – 19.3] | 74.2  [67.5 – 82.1] | 62.5  [56.0 – 70.3] | 11.7  [10.3 – 13.3] | 1999.6  [1823.2 – 2221.6] | 1686.7  [1505.1 – 1905.2] | 312.9  [276.1 – 355.7] |
| Leukemia | 323.5  [287.4 – 358.5] | 174.4  [141.8 – 201.4] | 149.1  [127.3 – 169.6] | 167.8  [148.8 – 187.5] | 94.4  [77.8 – 110.6] | 73.4  [63.6 – 83.1] | 6444.6  [5729.5 – 7189.1] | 3650.9  [2984.7 – 4288.6] | 2793.8  [2457.9 – 3168.2] |
| Lip and oral cavity | 239.8  [212.0 – 267.5] | 152.5  [129.7 – 176.2] | 87.2  [76.5 – 98.9] | 139.3  [123.3 – 156.8] | 90.3  [77.0 – 104.4] | 48.9  [42.3 – 56.4] | 3954.8  [3500.3 – 4470.9] | 2638.6  [2226.6 – 3049.8] | 1316.2  [1147.2 – 1514.2] |
| Liver | 381.9  [342.4 – 429.6] | 274.1  [238.0 – 317.1] | 107.8  [92.9 – 123.7] | 340.6  [305.2 – 376.9] | 239.3  [208.0 – 271.4] | 101.3  [87.2 – 115.2] | 9071.1  [8083.1 – 10145.7] | 6703.3  [5724.2 – 7679.0] | 2367.9  [2046.8 – 2718.2] |
| Malignant skin melanoma | 50.7  [37.9 – 60.0] | 26.9  [18.1 – 34.0] | 23.7  [17.0 – 29.6] | 14.0  [10.6 – 16.3] | 7.4  [5.1 – 9.5] | 6.5  [4.5 – 8.0] | 401.9  [304.9 – 467.8] | 221.8  [151.8 – 282.3] | 180.1  [125.1 – 222.0] |
| Mesothelioma | 11.4  [10.1 – 12.7] | 7.9  [7.0 – 8.7] | 3.5  [2.5 – 4.2] | 9.7  [8.7 – 10.7] | 6.7  [6.1 – 7.4] | 3.0  [2.2 – 3.6] | 254.1  [225.8 – 285.0] | 172.6  [155.5 – 194.2] | 81.4  [56.6 – 100.4] |
| Multiple myeloma | 53.3  [43.3 – 60.2] | 29.6  [22.3 – 34.9] | 23.7  [19.2 – 27.8] | 40.7  [33.3 – 45.8] | 22.3  [16.8 – 26.3] | 18.4  [14.7 – 21.6] | 989.9  [802.5 – 1121.6] | 558.7  [415.6 – 661.5] | 431.2  [337.6 – 508.2] |
| Nasopharynx | 150.7  [130.3 – 173.9] | 111.1  [92.4 – 132.3] | 39.7  [33.4 – 47.5] | 58.5  [52.7 – 64.6] | 42.2  [37.1 – 47.6] | 16.4  [14.2 – 18.7] | 1905.8  [1725.2 – 2096.5] | 1386.4  [1222.0 – 1563.6] | 519.4  [455.7 – 590.6] |
| Non-Hodgkin lymphoma | 214.7  [193.7 – 238.8] | 131.5  [116.3 – 149.4] | 83.3  [73.1 – 93.9] | 126.1  [115.0 – 137.9] | 75.4  [67.7 – 83.9] | 50.8  [45.2 – 56.6] | 3732.2  [3425.6 – 4084.1] | 2318.4  [2073.6 – 2588.4] | 1413.8  [1269.0 – 1575.4] |
| Non-melanoma skin | 525.7  [461.0 – 589.0] | 245.4  [214.6 – 279.1] | 280.3  [246.1 – 313.7] | 28.3  [25.3 – 30.8] | 16.6  [14.8 – 18.3] | 11.7  [9.6 – 13.6] | 592.7  [533.7 – 647.0] | 355.6  [316.3 – 395.8] | 237.1  [198.0 – 275.5] |
| Other malignant neoplasms | 455.0  [387.2 – 507.1] | 263.4  [204.7 – 305.9] | 191.7  [169.8 – 214.1] | 236.9  [194.9 – 265.5] | 134.2  [101.0 – 157.4] | 102.8  [89.4 – 114.5] | 7889.7  [6667.7 – 8760.0] | 4520.7  [3548.8 – 5249.2] | 3369.0  [2986.3 – 3738.9] |
| Other pharynx | 99.8  [87.8 – 111.8] | 74.5  [63.7 – 85.4] | 25.3  [21.1 – 29.8] | 83.1  [72.7 – 94.2] | 62.2  [52.5 – 73.2] | 20.9  [17.3 – 24.9] | 2373.6  [2064.8 – 2690.6] | 1778.4  [1491.8 – 2092.7] | 595.2  [492.9 – 717.1] |
| Ovarian | 144.0  [117.2 – 168.9] |  | 144.0  [117.2 – 168.9] | 92.4  [76.7 – 108.0] |  | 92.4  [76.7 – 108.0] | 2730.0  [2239.2 – 3220.0] |  | 2730.0  [2239.2 – 3220.0] |
| Pancreatic | 250.0  [224.1 – 274.3] | 138.6  [121.4 – 156.6] | 111.4  [96.6 – 126.0] | 249.8  [226.0 – 273.3] | 137.4  [121.5 – 155.2] | 112.4  [97.7 – 126.1] | 5692.2  [5152.4 – 6267.2] | 3315.4  [2901.2 – 3786.4] | 2376.8  [2089.1 – 2684.4] |
| Prostate | 380.9  [329.9 – 461.9] | 380.9  [329.9 – 461.9] |  | 162.0  [138.3 – 194.5] | 162.0  [138.3 – 194.5] |  | 2918.1  [2506.8 – 3518.5] | 2918.1  [2506.8 – 3518.5] |  |
| Stomach | 930.1  [821.9 – 1052.2] | 642.3  [546.6 – 756.1] | 287.9  [249.0 – 327.2] | 672.8  [597.0 – 746.9] | 443.8  [379.9 – 509.6] | 229.0  [198.5 – 261.5] | 15889.6  [14119.9 – 17684.4] | 10640.0  [9057.4 – 12271.0] | 5249.6  [4581.0 – 6016.8] |
| Testicular | 40.2  [35.4 – 46.9] | 40.2  [35.4 – 46.9] |  | 5.2  [4.6 – 5.9] | 5.2  [4.6 – 5.9] |  | 266.0  [236.1 – 300.7] | 266.0  [236.1 – 300.7] |  |
| Thyroid | 127.0  [111.1 – 139.7] | 40.0  [33.6 – 45.2] | 87.0  [73.2 – 97.3] | 27.0  [24.0 – 29.4] | 11.5  [9.8 – 12.9] | 15.5  [13.1 – 17.3] | 751.0  [665.1 – 819.8] | 316.8  [272.4 – 354.5] | 434.2  [362.8 – 486.7] |
| Tracheal, bronchus, and lung | 1305.5  [1150.2 – 1455.4] | 906.6  [771.0 – 1049.2] | 398.9  [339.7 – 464.5] | 1190.5  [1052.8 – 1330.5] | 825.7  [706.2 – 949.8] | 364.8  [312.1 – 421.1] | 27120.5  [23951.9 – 30414.2] | 19032.9  [16188.4 – 21991.6] | 8087.6  [6962.5 – 9358.9] |
| Uterine | 145.4  [122.6 – 173.4] |  | 145.4  [122.6 – 173.4] | 36.9  [31.5 – 44.7] |  | 36.9  [31.5 – 44.7] | 1028.9  [865.6 – 1219.0] |  | 1028.9  [865.6 – 1219.0] |

DALYs = Disability-adjusted Life Years. The figures inside square bracket represent the 95% uncertainty interval. Data Source: Global Burden of Disease, Injuries and Risk Factors 2019 Study

**Supplementary Table S8 Age-standardized Rates of Cancer Burden by Sex in 2019 in Asia**

|  | **Age-standardized Incidence Rate (per 100,000)** | | | **Age-standardized Mortality Rate (per 100,000)** | | | **Age-standardized DALYs Rate (per 100,000)** | | |
| --- | --- | --- | --- | --- | --- | --- | --- | --- | --- |
| **Cancer Type** | **Both Sexes** | **Males** | **Females** | **Both Sexes** | **Males** | **Females** | **Both Sexes** | **Males** | **Females** |
| Total Cancers | 197.6  [181.0 – 214.4] | 230.5  [205.4 – 258.7] | 171.1  [153.5 – 190.3] | 120.7  [110.1 – 130.7] | 152.8  [135.3 – 170.3] | 94.2  [83.7 – 105.1] | 2970.5  [2722.6 – 3206.5] | 3552.1  [3155.2 – 3974.4] | 2451.4  [2197.1 – 2723.0] |
| Bladder | 4.6  [4.1 – 5.0] | 7.8  [6.9 – 8.8] | 1.8  [1.6 – 2.1] | 2.2  [2.0 – 2.4] | 3.8  [3.4 – 4.2] | 1.0  [0.9 – 1.2] | 40.7  [37.2 – 44.6] | 66.4  [59.8 – 73.9] | 18.6  [16.4 – 20.9] |
| Brain and central nervous system | 4.0  [3.0 – 4.6] | 4.3  [2.9 – 5.3] | 3.8  [2.8 – 4.4] | 2.7  [2.1 – 3.1] | 3.2  [2.0 – 3.8] | 2.3  [1.7 – 2.8] | 102.8  [78.2 – 117.8] | 118.2  [77.6 – 144.7] | 87.1  [66.3 – 103.2] |
| Breast | 18.5  [16.5 – 20.7] | 0.5  [0.5 – 0.6] | 35.9  [32.0 – 40.2] | 7.1  [6.4 – 7.9] | 0.3  [0.3 – 0.3] | 13.4  [12.0 – 14.9] | 218.1  [195.7 – 242.3] | 7.5  [6.4 – 8.6] | 425.9  [381.4 – 474.1] |
| Cervical |  |  | 11.7  [9.4 – 13.5] |  |  | 5.8  [4.7 – 6.7] |  |  | 183.3  [147.7 – 212.5] |
| Colon and rectum | 23.9  [21.5 – 26.4] | 30.3  [26.6 – 34.5] | 18.2  [15.7 – 20.6] | 12.5  [11.2 – 13.6] | 15.4  [13.7 – 17.2] | 10.0  [8.6 – 11.2] | 275.5  [249.6 – 299.4] | 339.5  [300.6 – 381.0] | 216.1  [190.7 – 242.1] |
| Esophageal | 8.2  [7.0 – 9.4] | 12.6  [10.3 – 14.9] | 4.3  [3.4 – 5.1] | 7.7  [6.6 – 8.8] | 12.0  [9.9 – 14.2] | 3.9  [3.1 – 4.6] | 170.5  [145.9 – 194.9] | 265.9  [219.9 – 314.8] | 80.9  [67.5 – 94.1] |
| Gallbladder and biliary tract | 2.8  [2.1 – 3.1] | 2.8  [2.0 – 3.2] | 2.8  [2.0 – 3.2] | 2.5  [1.9 – 2.8] | 2.5  [1.9 – 2.8] | 2.6  [1.8 – 2.9] | 49.7  [39.3 – 54.9] | 48.2  [37.1 – 54.6] | 51.3  [36.8 – 59.6] |
| Hodgkin lymphoma | 0.8  [0.7 – 0.9] | 1.1  [0.9 – 1.3] | 0.6  [0.4 – 0.7] | 0.3  [0.2 – 0.3] | 0.4  [0.3 – 0.5] | 0.2  [0.1 – 0.2] | 12.2  [10.2 – 15.1] | 16.6  [12.8 – 21.9] | 7.7  [6.0 – 10.3] |
| Kidney | 2.7  [2.4 – 3.0] | 3.8  [3.3 – 4.3] | 1.8  [1.6 – 2.0] | 1.3  [1.2 – 1.5] | 1.9  [1.7 – 2.1] | 0.8  [0.7 – 0.9] | 32.4  [29.3 – 36.2] | 45.5  [40.2 – 51.5] | 20.1  [17.9 – 22.9] |
| Larynx | 2.4  [2.1 – 2.7] | 4.2  [3.8 – 4.8] | 0.7  [0.6 – 0.8] | 1.5  [1.4 – 1.7] | 2.7  [2.4 – 3.1] | 0.5  [0.4 – 0.5] | 39.7  [36.2 – 44.0] | 68.4  [61.1 – 77.2] | 12.3  [10.9 – 14.0] |
| Leukemia | 7.1  [6.4 – 7.9] | 7.8  [6.4 – 9.0] | 6.5  [5.6 – 7.4] | 3.7  [3.3 – 4.1] | 4.3  [3.6 – 5.0] | 3.1  [2.7 – 3.5] | 142.3  [126.9 – 159.2] | 160.5  [132.3 – 188.8] | 124.5  [110 – 140.3] |
| Lip and oral cavity | 4.9  [4.3 – 5.4] | 6.4  [5.4 – 7.3] | 3.5  [3.1 – 4.0] | 2.9  [2.6 – 3.3] | 3.9  [3.4 – 4.5] | 2.0  [1.7 – 2.3] | 79.0  [70.1 – 89.2] | 106.4  [90.2 – 122.7] | 52.4  [45.7 – 60.1] |
| Liver | 8.0  [7.2 – 8.9] | 11.8  [10.3 – 13.5] | 4.4  [3.8 – 5.0] | 7.2  [6.5 – 7.9] | 10.5  [9.2 – 11.9] | 4.2  [3.6 – 4.7] | 182.8  [163.3 – 203.8] | 272.5  [234.1 – 311.0] | 95.4  [82.7 – 109.1] |
| Malignant skin melanoma | 1.1  [0.8 – 1.3] | 1.2  [0.8 – 1.5] | 1.0  [0.7 – 1.2] | 0.3  [0.2 – 0.4] | 0.4  [0.2 – 0.4] | 0.3  [0.2 – 0.3] | 8.3  [6.3 – 9.6] | 9.3  [6.4 – 11.8] | 7.3  [5.1 – 9.0] |
| Mesothelioma | 0.2  [0.2 – 0.3] | 0.4  [0.3 – 0.4] | 0.1  [0.1 – 0.2] | 0.2  [0.2 – 0.2] | 0.3  [0.3 – 0.3] | 0.1  [0.1 – 0.1] | 5.1  [4.6 – 5.8] | 7.2  [6.6 – 8.1] | 3.2  [2.2 – 4.0] |
| Multiple myeloma | 1.1  [0.9 – 1.3] | 1.3  [1.0 – 1.5] | 1.0  [0.8 – 1.1] | 0.9  [0.7 – 1.0] | 1.0  [0.8 – 1.2] | 0.7  [0.6 – 0.9] | 20.1  [16.3 – 22.8] | 23.5  [17.6 – 27.6] | 17.1  [13.4 – 20.1] |
| Nasopharynx | 3.0  [2.6 – 3.4] | 4.5  [3.7 – 5.3] | 1.6  [1.3 – 1.9] | 1.2  [1.1 – 1.3] | 1.7  [1.5 – 2.0] | 0.7  [0.6 – 0.7] | 37.6  [34.1 – 41.3] | 54.9  [48.4 – 61.7] | 20.6  [18.1 – 23.4] |
| Non-Hodgkin lymphoma | 4.6  [4.2 – 5.2] | 6.0  [5.3 – 6.7] | 3.5  [3.1 – 3.9] | 2.7  [2.5 – 3.0] | 3.4  [3.1 – 3.8] | 2.1  [1.9 – 2.3] | 77.8  [71.4 – 85.0] | 97.9  [88.0 – 109.0] | 58.3  [52.5 – 64.8] |
| Non-melanoma skin | 11.3  [10.0 – 12.7] | 11.0  [9.7 – 12.5] | 11.5  [10.1 – 12.8] | 0.7  [0.6 – 0.7] | 0.9  [0.8 – 0.9] | 0.5  [0.4 – 0.6] | 12.6  [11.3 – 13.7] | 15.9  [14.2 – 17.6] | 9.6  [8.0 – 11.2] |
| Other malignant neoplasms | 9.8  [8.4 – 10.9] | 11.7  [9.0 – 13.5] | 8.2  [7.3 – 9.2] | 5.2  [4.2 – 5.8] | 6.1  [4.6 – 7.2] | 4.3  [3.8 – 4.8] | 169.5  [144.4 – 187.9] | 194.4  [152.5 – 225.1] | 145.7  [128.9 – 161.2] |
| Other pharynx | 2.0  [1.8 – 2.3] | 3.1  [2.7 – 3.5] | 1.0  [0.8 – 1.2] | 1.7  [1.5 – 1.9] | 2.6  [2.2 – 3.1] | 0.8  [0.7 – 1.0] | 46.8  [40.7 – 53.0] | 71.0  [59.8 – 83.5] | 23.4  [19.4 – 28.2] |
| Ovarian |  |  | 5.7  [4.7 – 6.7] |  |  | 3.7  [3.1 – 4.3] |  |  | 107.2  [88.0 – 126.7] |
| Pancreatic | 5.4  [4.8 – 5.9] | 6.3  [5.6 – 7.1] | 4.6  [4.0 – 5.2] | 5.5  [4.9 – 6.0] | 6.4  [5.7 – 7.1] | 4.7  [4.0 – 5.2] | 116.5  [105.5 – 128.0] | 139.2  [122.5 – 158.2] | 94.7  [83.4 – 106.9] |
| Prostate |  | 18.4  [15.9 – 22.2] |  |  | 9.2  [7.7 – 11.0] |  |  | 143.2  [122.6 – 172.3] |  |
| Stomach | 19.8  [17.5 – 22.3] | 28.7  [24.6 – 33.6] | 11.8  [10.2 – 13.4] | 14.7  [13.0 – 16.2] | 20.7  [17.8 – 23.6] | 9.5  [8.2 – 10.8] | 324.7  [288.6 – 360.6] | 449.2  [384.6 – 514.9] | 209.7  [183.2 – 239.9] |
| Testicular |  | 1.7  [1.5 – 2.0] |  |  | 0.2  [0.2 – 0.3] |  |  | 11.1  [9.8 – 12.5] |  |
| Thyroid | 2.6  [2.2 – 2.8] | 1.6  [1.4 – 1.9] | 3.5  [2.9 – 3.9] | 0.6  [0.5 – 0.6] | 0.5  [0.5 – 0.6] | 0.6  [0.5 – 0.7] | 15.4  [13.6 – 16.8] | 13.3  [11.4 – 14.9] | 17.5  [14.7 – 19.6] |
| Tracheal, bronchus, and lung | 27.8  [24.6 – 31.0] | 41.2  [35.3 – 47.3] | 16.2  [13.7 – 18.8] | 25.8  [22.8 – 28.7] | 38.3  [33.0 – 43.8] | 15.0  [12.8 – 17.3] | 553.7  [488.6 – 619.8] | 806.4  [688.1 – 928.3] | 321.2  [276.6 – 371.3] |
| Uterine |  |  | 5.6  [4.8 – 6.7] |  |  | 1.5  [1.3 – 1.8] |  |  | 40.2  [33.7 – 47.7] |

DALYs = Disability-adjusted Life Years. The figures inside square bracket represent the 95% uncertainty interval. Data Source: Global Burden of Disease, Injuries and Risk Factors 2019 Study

**Supplementary Table S9 Cancer Rank as per Age-standardized Rate in Asia**

|  | **Age-standardized Incidence Rate (per 100,000) (95% UI)** | | | | **Age-standardized Mortality Rate (per 100,000) (95% UI)** | | | | **Age-standardized DALYs Rate (per 100,000) (95% UI)** | | | |
| --- | --- | --- | --- | --- | --- | --- | --- | --- | --- | --- | --- | --- |
| **Cancer Type** | **1990** | **2019** | **Rank 1990** | **Rank 2019** | **1990** | **2019** | **Rank 1990** | **Rank 2019** | **1990** | **2019** | **Rank 1990** | **Rank 2019** |
| Bladder cancer | 3.5  [3.3 – 3.8] | 4.6  [4.1 – 5.0] | 13 | 15↓ | 2.4  [2.2 – 2.6] | 2.2  [2.0 – 2.4] | 15 | 16↓ | 45.7  [41.4 – 49.7] | 40.7  [37.2 – 44.6] | 18 | 18= |
| Brain and central nervous system cancer | 3.3  [2.7 – 4.5] | 4.0  [3.0 – 4.6] | 15 | 16↓ | 2.8  [2.2 – 3.7] | 2.7  [2.1 – 3.1] | 13 | 13= | 115.0  [86.2 – 162.9] | 102.8  [78.2 – 117.8] | 10 | 10= |
| Breast cancer | 10.8  [10.0 – 11.7] | 18.5  [16.5 – 20.7] | 6 | 4↑ | 6.3  [5.8 – 6.8] | 7.1  [6.4 – 7.9] | 6 | 6= | 194.6  [179.4 – 211.6] | 218.1  [195.7 – 242.3] | 7 | 4↑ |
| Cervical cancer | 6.3  [5.3 – 8.4] | 5.9  [4.7 – 6.8] | 10 | 11↓ | 4.0  [3.3 – 5.3] | 3.0  [2.4 – 3.5] | 9 | 11↓ | 122.9  [103.4 – 163.6] | 92.0  [74.2 – 106.6] | 9 | 11↓ |
| Colon and rectum cancer | 14.0  [13.2 – 14.8] | 23.9  [21.5 – 26.4] | 4 | 2↑ | 10.1  [9.4 – 10.9] | 12.5  [11.2 – 13.6] | 5 | 3↑ | 231.8  [214.8 – 249.1] | 275.5  [249.6 – 299.4] | 6 | 3↑ |
| Esophageal cancer | 11.7  [8.6 – 13.1] | 8.2  [7.0 – 9.4] | 5 | 8↓ | 12.0  [8.7 – 13.5] | 7.7  [6.6 – 8.8] | 4 | 4= | 280.7  [199.7 – 317.9] | 170.5  [145.9 – 194.9] | 4 | 6↓ |
| Gallbladder and biliary tract cancer | 2.7  [2.5 – 3.3] | 2.8  [2.1 – 3.1] | 16 | 20↓ | 2.6  [2.3 – 3.2] | 2.5  [1.9 – 2.8] | 14 | 15↓ | 53.1  [47.5 – 67.3] | 49.7  [39.3 – 54.9] | 17 | 16↑ |
| Hodgkin lymphoma | 0.9  [0.7 – 1] | 0.8  [0.7 – 0.9] | 26 | 28↓ | 0.5  [0.4 – 0.6] | 0.3  [0.2 – 0.3] | 26 | 27↓ | 20.9  [14.1 – 24.2] | 12.2  [10.2 – 15.1] | 23 | 26↓ |
| Kidney cancer | 1.4  [1.3 – 1.5] | 2.7  [2.4 – 3.0] | 24 | 21↑ | 0.9  [0.8 – 0.9] | 1.3  [1.2 – 1.5] | 22 | 20↑ | 23.0  [21.1 – 25.0] | 32.4  [29.3 – 36.2] | 22 | 21↑ |
| Larynx cancer | 2.4  [2.2 – 2.6] | 2.4  [2.1 – 2.7] | 18 | 23↓ | 2.0  [1.9 – 2.2] | 1.5  [1.4 – 1.7] | 17 | 19↓ | 53.5  [48.8 – 57.9] | 39.7  [36.2 – 44.0] | 16 | 19↓ |
| Leukemia | 8.8  [6.8 – 10.5] | 7.1  [6.4 – 7.9] | 7 | 10↓ | 5.3  [4.5 – 6.1] | 3.7  [3.3 – 4.1] | 8 | 10↓ | 244.5  [200.2 – 292.9] | 142.3  [126.9 – 159.2] | 5 | 8↓ |
| Lip and oral cavity cancer | 4.0  [3.6 – 4.4] | 4.9  [4.3 – 5.4] | 12 | 13↓ | 2.8  [2.5 – 3.0] | 2.9  [2.6 – 3.3] | 12 | 12= | 74.3  [67.4 – 81.9] | 79.0  [70.1 – 89.2] | 12 | 12= |
| Liver cancer | 14.1  [12.4 – 15.9] | 8.0  [7.2 – 8.9] | 3 | 9↓ | 14.1  [12.5 – 15.9] | 7.2  [6.5 – 7.9] | 3 | 5↓ | 401.0  [352.2 – 459.2] | 182.8  [163.3 – 203.8] | 3 | 5↓ |
| Malignant skin melanoma | 0.7  [0.6 – 1.0] | 1.1  [0.8 – 1.3] | 27 | 26↑ | 0.3  [0.3 – 0.5] | 0.3  [0.2 – 0.4] | 27 | 26↑ | 9.4  [7.2 – 12.6] | 8.3  [6.3 – 9.6] | 27 | 27= |
| Mesothelioma | 0.2  [0.2 – 0.3] | 0.2  [0.2 – 0.3] | 29 | 29= | 0.2  [0.2 – 0.2] | 0.2  [0.2 – 0.2] | 28 | 28= | 4.9  [4.0 – 6.4] | 5.1  [4.6 – 5.8] | 29 | 29= |
| Multiple myeloma | 0.9  [0.8 – 1.2] | 1.1  [0.9 – 1.3] | 25 | 25= | 0.8  [0.7 – 1.0] | 0.9  [0.7 – 1.0] | 23 | 22↑ | 19.3  [16.9 – 24.6] | 20.1  [16.3 – 22.8] | 24 | 23↑ |
| Nasopharynx cancer | 2.2  [2.0 – 2.5] | 3.0  [2.6 – 3.4] | 20 | 17↑ | 2.0  [1.8 – 2.1] | 1.2  [1.1 – 1.3] | 18 | 21↓ | 62.4  [56.0 – 68.4] | 37.6  [34.1 – 41.3] | 15 | 20↓ |
| Non-Hodgkin lymphoma | 2.7  [2.5 – 3.0] | 4.6  [4.2 – 5.2] | 17 | 14↑ | 2.2  [2.1 – 2.4] | 2.7  [2.5 – 3.0] | 16 | 14↑ | 68.5  [61.9 – 75.5] | 77.8  [71.4 – 85.0] | 13 | 13= |
| Non-melanoma skin cancer | 7.5  [6.7 – 8.5] | 11.3  [10.0 – 12.7] | 8 | 5↑ | 0.6  [0.5 – 0.7] | 0.7  [0.6 – 0.7] | 24 | 24= | 12.0  [10.7 – 13.2] | 12.6  [11.3 – 13.7] | 26 | 25↑ |
| Other malignant neoplasms | 6.9  [6.0 – 7.7] | 9.8  [8.4 – 10.9] | 9 | 6↑ | 5.4  [4.6 – 6.0] | 5.2  [4.2 – 5.8] | 7 | 8↓ | 182.4  [156.9 – 200.2] | 169.5  [144.4 – 187.9] | 8 | 7↑ |
| Other pharynx cancer | 1.6  [1.4 – 1.8] | 2.0  [1.8 – 2.3] | 22 | 24↓ | 1.5  [1.4 – 1.7] | 1.7  [1.5 – 1.9] | 19 | 18↑ | 42.4  [38.2 – 48.7] | 46.8  [40.7 – 53.0] | 19 | 17↑ |
| Ovarian cancer | 1.8  [1.6 – 2.4] | 2.9  [2.4 – 3.4] | 21 | 18↑ | 1.3  [1.1 – 1.7] | 1.9  [1.6 – 2.2] | 20 | 17↑ | 37.6  [32.2 – 50.3] | 54.2  [44.5 – 63.9] | 20 | 15↑ |
| Pancreatic cancer | 3.4  [3.2 – 3.6] | 5.4  [4.8 – 5.9] | 14 | 12↑ | 3.5  [3.2 – 3.7] | 5.5  [4.9 – 6.0] | 11 | 7↑ | 77.6  [71.5 – 84.0] | 116.5  [105.5 – 128.0] | 11 | 9↑ |
| Prostate cancer | 5.1  [4.3 – 5.9] | 8.3  [7.2 – 10.1] | 11 | 7↑ | 3.9  [3.3 – 4.6] | 3.9  [3.3 – 4.6] | 10 | 9↑ | 65.6  [54.4 – 75.8] | 64.5  [55.3 – 77.6] | 14 | 14= |
| Stomach cancer | 28.1  [26.0 – 30.2] | 19.8  [17.5 – 22.3] | 1 | 3↓ | 25.7  [23.6 – 27.8] | 14.7  [13.0 – 16.2] | 1 | 2↓ | 610.5  [560.9 – 663.8] | 324.7  [288.6 – 360.6] | 1 | 2↓ |
| Testicular cancer | 0.4  [0.3 – 0.5] | 0.9  [0.8 – 1.0] | 28 | 27↑ | 0.1  [0.1 – 0.1] | 0.1  [0.1 – 0.1] | 29 | 29= | 5.0  [4.5 – 5.5] | 5.6  [5.0 – 6.4] | 28 | 28= |
| Thyroid cancer | 1.5  [1.3 – 1.7] | 2.6  [2.2 – 2.8] | 23 | 22↑ | 0.6  [0.5 – 0.7] | 0.6  [0.5 – 0.6] | 25 | 25= | 14.8  [13.4 – 17.4] | 15.4  [13.6 – 16.8] | 25 | 24↑ |
| Tracheal, bronchus, and lung cancer | 22.6  [20.7 – 24.6] | 27.8  [24.6 – 31.0] | 2 | 1↑ | 22.7  [20.8 – 24.8] | 25.8  [22.8 – 28.7] | 2 | 1↑ | 537.7  [490.1 – 590.4] | 553.7  [488.6 – 619.8] | 2 | 1↑ |
| Uterine cancer | 2.2  [1.8 – 2.6] | 2.9  [2.4 – 3.4] | 19 | 19= | 1.1  [0.9 – 1.3] | 0.8  [0.7 – 0.9] | 21 | 23↓ | 28.4  [22.5 – 32.9] | 20.5  [17.2 – 24.3] | 21 | 22↓ |

DALYs = Disability-adjusted Life Years; UI = Uncertainty Interval. Data Source: Global Burden of Disease, Injuries and Risk Factors 2019 Study

**Supplementary Table S10 Decomposition Analysis of Cancer Trends in Asia, 1990 to 2019**

|  | **Change in Incident Cases, 1990 to 2019 (%)** | | | | | | | | | | | |
| --- | --- | --- | --- | --- | --- | --- | --- | --- | --- | --- | --- | --- |
|  | **Both Sexes** | | | | **Males** | | | | **Females** | | | |
| **Cancer Type** | **Due to Population Growth** | **Due to Population Aging** | **Due to change in incidence rates** | **Overall Change** | **Due to Population Growth** | **Due to Population Aging** | **Due to change in incidence rates** | **Overall Change** | **Due to Population Growth** | **Due to Population Aging** | **Due to change in incidence rates** | **Overall Change** |
| Bladder cancer | 42.7 | 111.0 | 76.7 | 230.4 | 41.6 | 111.9 | 100.6 | 254.2 | 43.8 | 113.3 | 6.7 | 163.8 |
| Brain and central nervous system cancer | 42.7 | 29.7 | 38.0 | 110.4 | 41.6 | 28.0 | 25.0 | 94.6 | 43.8 | 32.1 | 55.7 | 131.7 |
| Breast cancer | 42.7 | 75.3 | 155.3 | 273.3 | 41.6 | 102.1 | 129.5 | 273.2 | 43.8 | 76.8 | 152.7 | 273.4 |
| Cervical cancer |  |  |  |  |  |  |  |  | 43.8 | 67.4 | -17.1 | 94.2 |
| Colon and rectum cancer | 42.7 | 100.1 | 172.1 | 314.9 | 41.6 | 97.1 | 224.8 | 363.6 | 43.8 | 103.7 | 110.2 | 257.8 |
| Esophageal cancer | 42.7 | 100.7 | -74.5 | 69.0 | 41.6 | 97.0 | -52.9 | 85.8 | 43.8 | 107.1 | -114.8 | 36.1 |
| Gallbladder and biliary tract cancer | 42.7 | 114.0 | -1.1 | 155.6 | 41.6 | 110.3 | 19.9 | 171.9 | 43.8 | 116.4 | -17.0 | 143.3 |
| Hodgkin lymphoma | 42.7 | 43.7 | -12.1 | 74.4 | 41.6 | 45.3 | -15.7 | 71.3 | 43.8 | 41.0 | -4.3 | 80.5 |
| Kidney cancer | 42.7 | 65.9 | 208.6 | 317.2 | 41.6 | 69.5 | 264.6 | 375.8 | 43.8 | 60.4 | 129.2 | 233.5 |
| Larynx cancer | 42.7 | 92.8 | -3.1 | 132.5 | 41.6 | 92.0 | 2.7 | 136.3 | 43.8 | 93.6 | -25.1 | 112.3 |
| Leukemia | 42.7 | 0.8 | -23.7 | 19.9 | 41.6 | 5.0 | -17.4 | 29.2 | 43.8 | -3.4 | -29.9 | 10.5 |
| Lip and oral cavity cancer | 42.7 | 83.1 | 51.6 | 177.5 | 41.6 | 81.9 | 51.4 | 175.0 | 43.8 | 84.2 | 53.8 | 181.9 |
| Liver cancer | 42.7 | 83.2 | -101.9 | 24.0 | 41.6 | 78.3 | -95.1 | 24.9 | 43.8 | 92.3 | -114.2 | 21.9 |
| Malignant skin melanoma | 42.7 | 77.1 | 97.4 | 217.2 | 41.6 | 77.7 | 110.6 | 230.0 | 43.8 | 76.5 | 83.5 | 203.9 |
| Mesothelioma | 42.7 | 89.4 | 10.0 | 142.1 | 41.6 | 93.9 | 48.6 | 184.2 | 43.8 | 83.6 | -45.7 | 81.7 |
| Multiple myeloma | 42.7 | 101.5 | 45.4 | 189.7 | 41.6 | 98.7 | 70.6 | 210.9 | 43.8 | 104.4 | 18.7 | 167.0 |
| Nasopharynx cancer | 42.7 | 68.3 | 73.5 | 184.5 | 41.6 | 67.1 | 115.3 | 224.0 | 43.8 | 69.0 | -0.8 | 112.1 |
| Non-Hodgkin lymphoma | 42.7 | 61.3 | 144.5 | 248.4 | 41.6 | 57.7 | 157.2 | 256.5 | 43.8 | 66.8 | 125.8 | 236.4 |
| Non-melanoma skin cancer | 42.7 | 110.2 | 141.0 | 294.0 | 41.6 | 98.3 | 178.7 | 318.6 | 43.8 | 118.7 | 112.1 | 274.7 |
| Other malignant neoplasms | 42.7 | 40.7 | 77.8 | 161.2 | 41.6 | 43.3 | 90.9 | 175.8 | 43.8 | 37.7 | 62.0 | 143.5 |
| Other neoplasms | 42.7 | 32.5 | -11.8 | 63.4 | 41.6 | 37.6 | -7.9 | 71.4 | 43.8 | 29.0 | -15.1 | 57.7 |
| Other pharynx cancer | 42.7 | 85.8 | 62.4 | 190.9 | 41.6 | 85.8 | 77.2 | 204.7 | 43.8 | 83.9 | 29.1 | 156.8 |
| Ovarian cancer |  |  |  |  |  |  |  |  | 43.8 | 70.5 | 129.5 | 243.8 |
| Pancreatic cancer | 42.7 | 105.8 | 139.4 | 287.9 | 41.6 | 100.4 | 131.7 | 273.8 | 43.8 | 112.7 | 150.4 | 306.9 |
| Prostate cancer |  |  |  |  | 41.6 | 132.1 | 177.9 | 351.7 |  |  |  |  |
| Stomach cancer | 42.7 | 98.1 | -74.5 | 66.4 | 41.6 | 96.8 | -58.0 | 80.4 | 43.8 | 100.5 | -102.6 | 41.7 |
| Testicular cancer |  |  |  |  | 41.6 | 1.6 | 181.0 | 224.2 |  |  |  |  |
| Thyroid cancer | 42.7 | 60.9 | 153.7 | 257.3 | 41.6 | 67.3 | 268.6 | 377.6 | 43.8 | 59.6 | 116.8 | 220.2 |
| Tracheal, bronchus, and lung cancer | 42.7 | 99.3 | 48.5 | 190.5 | 41.6 | 98.7 | 39.5 | 179.8 | 43.8 | 101.7 | 72.7 | 218.2 |
| Uterine cancer |  |  |  |  |  |  |  |  | 43.8 | 83.4 | 62.9 | 190.1 |

Source: Author’s Calculation using estimates from Global Burden of Disease, Injuries and Risk Factors 2019 Study

**Supplementary Table S11 Cancer Burden by Country, Males**

|  | **Incidence (95% UI)** | | | | **Deaths (95% UI)** | | | | **Disability-adjusted Life Years (95% UI)** | | | |
| --- | --- | --- | --- | --- | --- | --- | --- | --- | --- | --- | --- | --- |
| **Location** | **Counts, 2019 (in thousands)** | **Percent change, 1990-2019** | **Age-standardized rate (per 100,000) 2019** | **Percent change, 1990-2019** | **Counts, 2019 (in thousands)** | **Percent change, 1990-2019** | **Age-standardized rate (per 100,000)**  **2019** | **Percent change, 1990-2019** | **Counts, 2019 (in thousands)** | **Percent change, 1990-2019** | **Age-standardized rate (per 100,000)**  **2019** | **Percent change, 1990-2019** |
| ***Central Asia*** | | | | | | | | | | | | |
| Kazakhstan | 16.7  [14.7 – 18.9] | 4.8  [-8.0 to 19.0] | 234.5  [207.8 – 262.2] | -24.3  [-33.0 to -14.7] | 11.2  [9.7 – 12.7] | -11.8  [-23.9 to 0.9] | 166.1  [145.5 – 188.2] | -35.7  [-44.1 to -27.0] | 317.0  [276.5 – 361.9] | -19.2  [-30.2 to -7.2] | 4074.9  [3569.0 – 4633.2] | -40.7  [-48.7 to -32.4] |
| Kyrgyzstan | 3.2  [2.9 – 3.6] | 8.7  [-3.8 to 21.4] | 154.7  [138.8 – 172.3] | -31.1  [-38.6 to -23.4] | 2.2  [1.9 – 2.5] | -3.7  [-15.8 to 9.1] | 111.5  [98.8 – 125.6] | -38.0  [-45.5 to -30.0] | 66.2  [58.2 – 74.7] | -8.6  [-20.4 to 4.0] | 2844.1  [2509.4 – 3208.3] | -43.4  [-50.5 to -35.9] |
| Tajikistan | 3.9  [3.3 – 4.8] | 47.2  [24.0 to 79.2] | 161.0  [137.1 – 193.0] | -14.5  [-27.7 to 2.8] | 2.8  [2.3 – 3.5] | 39.0  [13.8 to 73.6] | 129.9  [108.4 – 161.6] | -14.9  [-30.5 to 5.0] | 93.6  [77.4 – 116.6] | 36.1  [11.4 to 69.7] | 3128.2  [2569.6 – 3884.9] | -27.3  [-40.0 to -9.7] |
| Turkmenistan | 3.1  [2.6 – 3.7] | 65.4  [38.9 to 98.5] | 162.4  [136.3 – 194.2] | -18.8  [-31.6 to -3.5] | 1.9  [1.6 – 2.4] | 41.3  [14.7 to 75.2] | 110.1  [89.8 – 136.2] | -31.9  [-44.6 to -16.4] | 64.4  [52.6 – 79.5] | 37.9  [11.9 to 71.0] | 3041.8  [2475.3 – 3749.2] | -31.1  [-43.7 to -14.9] |
| Uzbekistan | 16.9  [14.5 – 19.4] | 75.3  [50.9 to 101.5] | 170.4  [148.6 – 192.2] | -2.7  [-14.6 to 9.9] | 11.0  [9.2 – 12.7] | 57.5  [32.3 to 84.2] | 123.3  [105.1 – 140.7] | -9.3  [-22.1 to 3.9] | 377.3  [317.8 – 437.9] | 56.8  [31.9 to 83.0] | 3154.0  [2673.1 – 3636.4] | -17.7  [-29.9 to -4.8] |
| ***Eastern Asia*** | | | | | | | | | | | | |
| China | 2860.6  [2318.2 – 3485.4] | 178.9  [119.2 to 261.5] | 309.6  [254.5 – 372.2] | 28.4  [2.6 to 64.5] | 1746.2  [1407.5 – 2143.6] | 95.7  [49.6 to 157.0] | 197.2  [160.9 – 237.8] | -12.7  [-32.0 to 12.7] | 43473.3  [34868.5 – 53748.4] | 57.3  [19.7 to 106.6] | 4521.8  [3674.7 – 5517.8] | -22.4  [-40.2 to 1.3] |
| Japan | 503.6  [411.2 – 610.7] | 96.4  [61.9 to 137.2] | 333.5  [273.9 – 404.3] | -6.7  [-23.2 to 12.8] | 253.5  [227.7 – 267.6] | 76.7  [63.3 to 84.3] | 152.3  [138.9 – 159.8] | -26.0  [-30.1 to -23.5] | 4391.2  [4074.6 – 4595.9] | 28.5  [21.3 to 33.1] | 3119.6  [2935.4 – 3250.6] | -31.8  [-34.5 to -29.8] |
| Mongolia | 3.9  [3.1 – 4.9] | 112.4  [62.8 to 179.9] | 393.4  [321.7 – 480.4] | 3.0  [-19.3 to 32.4] | 3.4  [2.7 – 4.4] | 104.7  [54.5 to 174.2] | 379.7  [308.0 – 463.3] | 2.6  [-20.0 to 32.9] | 100.4  [78.2 – 130.4] | 112.2  [58.2 to 188.8] | 8625.2  [6852.0 – 10949.6] | -2.8  [-26.5 to 30.1] |
| North Korea | 26.8  [21.9 – 31.8] | 72.2  [32.8 to 119.6] | 199.8  [167.3 – 232.7] | -10.3  [-28.3 to 10.3] | 22.6  [18.5 – 26.7] | 78.8  [38.9 to 126.7] | 175.6  [146.9 – 203.7] | -12.4  [-29.2 to 6.4] | 651.5  [516.2 – 794.6] | 53.4  [14.4 to 99.9] | 4518.5  [3665.8 – 5415.4] | -16.5  [-35.9 to 6.1] |
| South Korea | 126.9  [107.6 – 148.0] | 235.2  [181.4 to 293.2] | 330.4  [282.6 – 383.4] | 15.2  [-3.4 to 34.5] | 63.0  [58.3 – 67.0] | 117.0  [98.6 to 134.0] | 170.2  [156.8 – 181.5] | -28.8  [-37.7 to -23.8] | 1410.7  [1318.2 – 1504.5] | 57.0  [45.4 to 71.0] | 3526.4  [3281.3 – 3760.1] | -40.4  [-46.0 to -35.9] |
| Taiwan [Province of China] | 65.8  [51.6 – 85.3] | 248.5  [172.9 to 348.1] | 369.9  [291.1 – 476.9] | 71.7  [35.4 to 119.3] | 34.7  [27.6 – 44.5] | 164.1  [109.0 to 236.2] | 191.4  [152.1 – 244.2] | 19.8  [-5.2 to 52.0] | 830.2  [651.8 – 1075.2] | 117.5  [70.1 to 182.3] | 4660.8  [3667.1 – 5982.8] | 12.9  [-11.6 to 45.6] |
| ***South-eastern Asia*** | | | | | | | | | | | | |
| Brunei Darussalam | 0.4  [0.4 – 0.5] | 166.4  [117.0 to 233.1] | 345.5  [300.3 – 397.9] | 2.5  [-14.8 to 25.1] | 0.3  [0.2 – 0.3] | 125.3  [85.4 to 177.6] | 257.8  [227.6 – 291.4] | -11.1  [-25.2 to 7.8] | 7.6  [6.5 – 8.8] | 109.1  [70.6 to 158.7] | 5076.2  [4406.2 – 5822.0] | -17.1  [-31.1 to 1.8] |
| Cambodia | 8.7  [6.9 – 10.1] | 141.1  [76.6 to 222.9] | 174.5  [139.9 – 201.4] | 11.1  [-13.7 to 37.6] | 7.5  [5.9 – 8.6] | 135.3  [78.5 to 200.2] | 162.5  [130.5 – 186.1] | 3.9  [-19.1 to 27.1] | 215.1  [169.4 – 256.1] | 90.6  [36.4 to 162.8] | 3829.9  [3047.9 – 4468.1] | -5.5  [-28.1 to 20.4] |
| Indonesia | 138.8  [112.8 – 168.0] | 164.9  [107.4 to 235.2] | 140.7  [115.7 – 167.5] | 39.6  [11.9 to 71.9] | 116.0  [93.7 – 140.5] | 159.3  [101.7 to 226.8] | 127.6  [104.9 – 151.5] | 32.9  [5.1 to 63.3] | 3246.6  [2618.7 – 4046.3] | 113.4  [61.8 to 175.5] | 2948.6  [2413.6 – 3567.1] | 18.6  [-6.6 to 48.7] |
| Lao PDR | 2.9  [2.2 – 3.5] | 63.9  [17.1 to 130.0] | 135.1  [105.1 – 162.9] | -14.0  [-34.9 to 12.9] | 2.5  [1.9 – 3.1] | 60.3  [18.6 to 115.8] | 128.6  [100.7 – 153.2] | -17.3  [-36.8 to 6.7] | 75.8  [56.3 – 95.1] | 40.6  [0.3 to 102.5] | 3100.2  [2337.3 – 3805.4] | -25.5  [-45.3 to 1.3] |
| Malaysia | 23.7  [19.1 – 29.1] | 245.3  [172.6 to 331.5] | 176.5  [143.1 – 215.3] | 23.8  [-2.1 to 52.6] | 16.5  [13.2 – 20.2] | 187.8  [127.5 to 256.5] | 130.6  [106.5 – 158.6] | 2.1  [-18.9 to 25.6] | 440.3  [351.9 – 544.3] | 151.9  [96.5 to 215.5] | 3093.2  [2478.1 – 3812.5] | -3.6  [-24.3 to 20.3] |
| Myanmar | 29.8  [24.3 – 36.9] | 70.8  [27.8 to 139.7] | 149.0  [124.2 – 181.3] | 1.5  [-21.1 to 35.4] | 25.9  [21.4 – 32.0] | 64.5  [25.7 to 125.0] | 138.3  [116.4 – 167.6] | -5.0  [-25.6 to 27.3] | 734.7  [584.6 – 924.2] | 41.2  [2.9 to 107.2] | 3353.3  [2714.6 – 4170.1] | -12.3  [-34.2 to 22.3] |
| Philippines | 52.9  [41.6 – 67.7] | 114.8  [62.2 to 181.7] | 139.6  [110.8 – 178.8] | -6.3  [-29.4 to 25.4] | 40.8  [31.7 – 52.3] | 104.4  [52.9 to 174.3] | 116.8  [91.7 – 148.6] | -13.1  [-34.5 to 16.8] | 1263.9  [990.2 – 1609.4] | 86.2  [41.7 to 142.9] | 2982.7  [2325.2 – 3787.7] | -15.8  [-36.3 to 12.7] |
| Singapore | 8.9  [7.2 – 11.0] | 188.5  [134.2 to 258.1] | 245.6  [200.6 – 301.4] | -18.5  [-33.7 to 0.1] | 3.8  [3.5 – 4.0] | 94.9  [82.5 to 105.2] | 110.8  [101.8 – 117.2] | -47.2  [-50.6 to -44.3] | 86.8  [81.6 – 92.0] | 62.6  [52.9 to 72.6] | 2323.4  [2167.7 – 2471.2] | -51.7  [-54.7 to -48.6] |
| Thailand | 86.2  [64.5 – 112.7] | 166.6  [96.7 to 256.1] | 190.6  [143.5 – 247.7] | 3.8  [-22.0 to 38.3] | 65.3  [48.7 – 85.6] | 143.8  [78.7 to 227.7] | 144.2  [108.4 – 188.6] | -12.0  [-34.8 to 17.4] | 1673.3  [1241.7 – 2211.1] | 101.1  [45.9 to 173.4] | 3626.4  [2726.6 – 4762.5] | -13.8  [-36.5 to 16.0] |
| Timor-Leste | 0.5  [0.3 – 0.6] | 194.5  [107.6 to 309.0] | 118.6  [83.7 – 144.4] | 23.4  [-6.5 to 59.3] | 0.4  [0.3 – 0.5] | 206.7  [123.2 to 304.4] | 111.8  [78.3 – 135.8] | 18.2  [-10.1 to 51.5] | 12.0  [7.9 – 15.1] | 124.8  [55.0 to 228.2] | 2632.7  [1767.8 – 3282.2] | 8.5  [-20.7 to 43.2] |
| Viet Nam | 88.4  [71.4 – 106.3] | 220.3  [137.9 to 316.0] | 214.8  [177.1 – 253.1] | 38.8  [5.5 to 78.3] | 63.6  [51.9 – 75.8] | 155.2  [90.6 to 232.3] | 166.3  [139.1 – 194.2] | 12.3  [-14.1 to 45.8] | 1817.6  [1453.2 – 2216.6] | 150.5  [82.7 to 232.8] | 4061.0  [3297.8 – 4864.9] | 9.0  [-18.9 to 42.7] |
| ***Southern Asia*** | | | | | | | | | | | | |
| Afghanistan | 10.7  [8.2 – 13.7] | 73.3  [35.1 to 128.5] | 152.4  [120.7 – 187.7] | -3.9  [-26.2 to 22.1] | 8.9  [6.8 – 11.5] | 58.1  [22.1 to 105.8] | 143.9  [112.4 – 177.5] | -6.3  [-28.2 to 18.8] | 318.6  [237.2 – 426.1] | 88.1  [45.5 to 161.2] | 3573.0  [2721.0 – 4598.3] | -9.9  [-32.4 to 19.4] |
| Bangladesh | 69.7  [50.1 – 91.0] | 90.8  [36.9 to 161.3] | 101.9  [73.2 – 132.6] | -17.6  [-38.9 to 9.0] | 60.7  [43.3 – 79.8] | 83.9  [33.1 to 148.6] | 91.9  [65.8 – 120.8] | -23.8  [-43.1 to 0.1] | 1678.5  [1198.5 – 2200.6] | 48.8  [1.2 to 113.3] | 2352.6  [1685.0 – 3083.1] | -28.0  [-48.1 to -3.0] |
| Bhutan | 0.3  [0.2 – 0.4] | 150.3  [73.6 to 277.1] | 102.7  [77.2 – 129.8] | 20.0  [-13.8 to 66.4] | 0.3  [0.2 – 0.3] | 143.4  [71.3 to 252.0] | 93.0  [71.3 – 117.5] | 9.8  [-20.4 to 49.1] | 6.9  [4.9 – 9.2] | 79.0  [16.6 to 192.1] | 2208.8  [1612.1 – 2881.4] | 0.2  [-30.7 to 44.4] |
| India | 563.0  [464.9 – 675.8] | 142.8  [89.2 to 207.3] | 99.9  [82.9 – 119.2] | 7.3  [-14.9 to 35.2] | 468.5  [383.6 – 566.7] | 132.2  [84.0 to 193.3] | 87.7  [72.7 – 105.6] | -1.4  [-21.9 to 24.0] | 13369.3  [10937.0 – 16164.3] | 97.6  [54.4 to 152.2] | 2207.9  [1810.1 – 2665.4] | -3.8  [-23.2 to 21.1] |
| Iran | 67.4  [58.9 – 73.5] | 196.8  [160.9 to 257.8] | 184.0  [161.6 – 200.5] | 20.8  [8.4 to 47.7] | 38.9  [35.3 – 42.3] | 145.0  [117.9 to 202.7] | 113.0  [102.6 – 122.6] | -8.5  [-17.3 to 15.3] | 1000.2  [889.6 – 1098.7] | 89.9  [64.1 to 130.6] | 2627.1  [2348.5 – 2880.7] | -11.8  [-21.5 to 9.1] |
| Maldives | 0.2  [0.2 – 0.2] | 214.2  [137.7 to 318.0] | 109.1  [91.8 – 127.7] | 4.2  [-17.6 to 34.1] | 0.1  [0.1 – 0.1] | 137.3  [82.7 to 214.9] | 76.8  [64.0 – 89.9] | -22.6  [-38.7 to 0.2] | 3.4  [2.8 – 4.0] | 93.6  [41.1 to 168.4] | 1703.5  [1433.4 – 2009.7] | -30.6  [-46.3 to -9.0] |
| Nepal | 12.6  [9.9 – 15.0] | 121.2  [59.7 to 190.8] | 118.2  [93.2 – 138.4] | 12.7  [-17.4 to 46.6] | 11.4  [9.0 – 13.4] | 119.3  [59.3 to 188.1] | 113.2  [89.3 – 132.2] | 7.6  [-20.2 to 40.2] | 318.6  [247.0 – 381.9] | 70.9  [20.0 to 132.7] | 2746.2  [2133.9 – 3275.0] | -3.0  [-30.1 to 28.6] |
| Pakistan | 109.0  [84.1 – 142.7] | 141.8  [83.9 to 221.3] | 167.0  [128.9 – 218.7] | 27.0  [-3.5 to 68.5] | 88.0  [68.8 – 112.9] | 114.8  [61.6 to 183.9] | 149.0  [115.5 – 190.3] | 18.4  [-10.1 to 54.9] | 3039.0  [2382.5 – 3869.3] | 133.8  [77.2 to 210.7] | 4032.5  [3156.5 – 5148.9] | 20.0  [-9.5 to 59.0] |
| Sri Lanka | 14.4  [10.8 – 18.9] | 157.6  [93.0 to 244.7] | 128.8  [97.4 – 166.9] | 29.8  [-1.7 to 71.8] | 9.4  [7.1 – 12.4] | 107.2  [54.8 to 178.7] | 87.6  [66.7 – 114.4] | 0.8  [-23.9 to 34.2] | 244.9  [180.8 – 322.0] | 81.3  [34.9 to 145.8] | 2119.4  [1576.6 – 2773.6] | -1.2  [-25.8 to 32.6] |
| ***Western Asia*** | | | | | | | | | | | | |
| Armenia | 4.7  [4.1 – 5.5] | 56.1  [34.0 to 80.0] | 272.4  [234.6 – 314.4] | 11.3  [-3.9 to 27.9] | 3.3  [2.8 – 3.8] | 42.2  [20.6 to 65.7] | 189.6  [161.1 – 219.8] | -1.5  [-16.4 to 14.4] | 81.8  [68.9 – 96.0] | 14.1  [-3.7 to 33.8] | 4637.9  [3922.5 – 5425.1] | -11.9  [-25.2 to 2.8] |
| Azerbaijan | 9.8  [8.1 – 12.1] | 83.9  [50.4 to 129.3] | 227.7  [191.5 – 277.6] | -1.2  [-18.2 to 20.8] | 6.9  [5.6 – 8.7] | 71.7  [37.7 to 117.0] | 171.4  [140.9 – 212.6] | -7.2  [-25.1 to 15.9] | 210.9  [169.3 – 264.7] | 55.9  [22.7 to 101.4] | 4387.5  [3566.4 – 5481.6] | -16.1  [-32.7 to 6.2] |
| Bahrain | 0.9  [0.7 – 1.2] | 382.0  [269.9 to 532.3] | 181.1  [145.2 – 217.0] | -18.7  [-35.7 to 3.8] | 0.5  [0.4 – 0.6] | 206.2  [134.0 to 302.4] | 123.2  [99.2 – 147.4] | -40.7  [-53.4 to -24.5] | 14.0  [11.0 – 17.5] | 205.2  [134.2 to 301.6] | 2347.3  [1868.8 – 2845.3] | -43.7  [-56.2 to -27.5] |
| Cyprus | 3.3  [2.9 – 3.8] | 283.0  [228.9 to 348.4] | 367.9  [320.5 – 417.4] | 60.0  [37.2 to 87.3] | 1.3  [1.1 – 1.4] | 155.2  [120.5 to 205.5] | 147.5  [130.0 – 165.7] | 4.1  [-10.3 to 22.9] | 27.4  [24.3 – 30.5] | 132.0  [101.3 to 174.5] | 3059.2  [2710.8 – 3411.8] | -0.3  [-14.1 to 17.5] |
| Georgia | 6.8  [5.9 – 7.8] | 10.4  [-6.4 to 29.5] | 289.4  [249.1 – 332.1] | 19.0  [0.8 to 39.2] | 4.8  [4.1 – 5.5] | 10.8  [-7.7 to 31.4] | 201.7  [171.0 – 233.0] | 15.0  [-4.0 to 36.1] | 124.7  [105.2 – 145.4] | -5.3  [-21.7 to 13.0] | 5363.8  [4537.7 – 6230.4] | 8.8  [-9.6 to 29.4] |
| Iraq | 20.2  [15.9 – 24.4] | 234.0  [150.2 to 339.6] | 170.4  [135.8 – 201.3] | 27.5  [-1.2 to 61.4] | 13.0  [10.2 – 15.7] | 187.3  [116.8 to 274.0] | 125.1  [99.7 – 146.7] | 10.0  [-14.7 to 37.8] | 386.7  [299.7 – 482.1] | 154.9  [84.6 to 245.7] | 2926.2  [2297.6 – 3549.2] | 0.2  [-24.6 to 30.1] |
| Israel | 16.7  [13.6 – 20.4] | 179.5  [127.7 to 239.9] | 322.8  [262.2 – 392.2] | 19.5  [-2.6 to 45.5] | 7.5  [7.0 – 8.1] | 109.8  [98.4 to 121.2] | 143.3  [132.9 – 153.2] | -13.4  [-17.7 to -8.8] | 158.9  [149.6 – 169.6] | 96.5  [85.9 to 107.7] | 3107.7  [2930.6 – 3313.6] | -14.7  [-19.1 to -9.7] |
| Jordan | 5.9  [4.7 – 7.2] | 477.6  [348.9 to 643.3] | 161.0  [130.0 – 195.2] | 24.0  [-3.9 to 59.4] | 3.1  [2.5 – 3.8] | 342.1  [233.0 to 478.6] | 99.7  [79.0 – 121.0] | -5.9  [-28.1 to 23.3] | 91.1  [72.5 – 112.3] | 284.6  [194.1 to 402.9] | 2274.2  [1793.5 – 2795.7] | -10.3  [-32.1 to 17.6] |
| Kuwait | 2.4  [1.9 – 2.9] | 348.2  [258.8 to 446.0] | 154.8  [124.7 – 189.0] | 31.3  [7.0 to 59.6] | 1.0  [0.8 – 1.3] | 265.6  [198.5 to 347.7] | 79.1  [64.1 – 96.3] | 1.0  [-16.7 to 22.3] | 27.4  [21.9 – 33.9] | 172.8  [119.0 to 237.5] | 1686.1  [1355.8 – 2068.9] | -11.0  [-27.9 to 9.6] |
| Lebanon | 8.1  [6.8 – 10.2] | 256.8  [189.0 to 385.4] | 342.7  [286.7 – 431.7] | 67.9  [37.0 to 126.4] | 4.0  [3.3 – 5.1] | 139.5  [93.2 to 244.2] | 169.4  [143.5 – 219.5] | 5.7  [-13.9 to 49.8] | 91.1  [74.7 – 114.4] | 103.9  [60.9 to 188.1] | 3827.0  [3137.7 – 4818.2] | 4.1  [-17.2 to 45.8] |
| Oman | 1.8  [1.5 – 2.1] | 258.8  [175.5 to 374.4] | 160.6  [140.0 – 183.2] | 32.2  [5.0 to 69.7] | 0.8  [0.6 – 0.9] | 130.8  [75.2 to 211.0] | 97.1  [84.3 – 110.5] | -3.1  [-24.2 to 27.3] | 25.8  [20.8 – 32.1] | 122.3  [64.0 to 206.1] | 2018.2  [1723.5 – 2358.0] | -12.8  [-32.8 to 16.1] |
| Palestine | 2.3  [2.0 – 2.7] | 192.8  [129.4 to 272.9] | 201.7  [175.4 – 230.5] | 11.0  [-12.3 to 40.9] | 1.5  [1.3 – 1.8] | 146.9  [91.2 to 217.2] | 155.1  [135.2 – 176.2] | -3.0  [-23.6 to 23.3] | 45.6  [39.6 – 52.2] | 144.6  [87.9 to 220.3] | 3399.3  [2948.9 – 3879.0] | -8.3  [-29.6 to 18.8] |
| Qatar | 1.4  [1.0 – 1.8] | 1070.6  [739.5 to 1519.4] | 211.3  [165.7 – 263.0] | 25.0  [-7.5 to 66.8] | 0.5  [0.4 – 0.7] | 569.0  [371.7 to 845.7] | 136.8  [108.3 – 169.0] | -11.0  [-32.7 to 17.2] | 18.7  [14.0 – 24.4] | 542.9  [356.5 to 810.8] | 2437.2  [1888.4 – 3075.2] | -21.8  [-42.5 to 7.2] |
| Saudi Arabia | 16.2  [12.9 – 20.7] | 435.8  [296.9 to 636.9] | 129.1  [107.0 – 159.6] | 52.7  [18.8 to 106.2] | 7.3  [5.9 – 9.3] | 192.1  [116.8 to 305.9] | 74.2  [62.3 – 91.9] | -5.2  [-25.0 to 28.9] | 247.3  [195.5 – 319.2] | 217.4  [130.3 to 357.9] | 1739.1  [1429.1 – 2170.1] | -2.9  [-25.9 to 34.9] |
| Syrian Arab Republic | 7.7  [6.0 – 10.2] | 110.7  [52.6 to 205.4] | 122.2  [96.0 – 158.8] | 14.1  [-15.9 to 61.8] | 4.9  [3.8 – 6.5] | 86.1  [33.0 to 166.0] | 83.5  [65.1 – 108.4] | -5.9  [-32.1 to 34.4] | 139.2  [106.1 – 184.0] | 46.4  [2.1 to 117.7] | 2073.1  [1600.0 – 2720.9] | -12.4  [-37.7 to 27.6] |
| Turkey | 107.4  [87.6 – 130.2] | 152.8  [97.8 to 228.1] | 259.2  [212.3 – 312.1] | 11.9  [-12.1 to 42.4] | 65.5  [52.9 – 79.7] | 90.0  [44.3 to 146.8] | 162.4  [131.4 – 197.3] | -18.5  [-37.2 to 4.6] | 1673.5  [1348.3 – 2052.9] | 58.4  [19.4 to 111.9] | 3917.1  [3170.0 – 4788.6] | -24.5  [-42.6 to -0.6] |
| United Arab Emirates | 7.3  [5.1 – 10.2] | 1084.6  [733.5 to 1585.7] | 202.4  [154.2 – 258.0] | 8.9  [-16.6 to 49.2] | 4.0  [2.8 – 5.6] | 847.7  [564.1 to 1279.2] | 151.1  [117.1 – 191.6] | -7.7  [-28.7 to 28.0] | 153.6  [105.1 – 215.8] | 814.5  [530.0 to 1229.3] | 3388.2  [2549.6 – 4408.9] | -7.0  [-30.9 to 29.5] |
| Yemen | 8.8  [6.7 – 11.2] | 184.6  [107.4 to 304.6] | 125.8  [97.8 – 159.2] | 6.5  [-17.8 to 41.3] | 7.0  [5.3 – 9.0] | 173.4  [100.8 to 285.0] | 110.3  [84.7 – 140.5] | -0.2  [-23.0 to 33.9] | 215.6  [157.9 – 281.0] | 145.0  [66.9 to 273.3] | 2615.0  [1948.3 – 3391.1] | -2.7  [-27.6 to 36.4] |

UI = Uncertainty Interval. Data Source: Global Burden of Disease, Injuries and Risk Factors 2019 Study

**Supplementary Table S12 Cancer Burden by Country, Females**

|  | **Incidence (95% UI)** | | | | **Deaths (95% UI)** | | | | **Disability-adjusted Life Years (95% UI)** | | | |
| --- | --- | --- | --- | --- | --- | --- | --- | --- | --- | --- | --- | --- |
| **location** | **Counts,**  **2019 (in thousands)** | **Percent change, 1990-2019** | **Age-standardized rate (per 100,000) 2019** | **Percent change, 1990-2019** | **Counts, 2019 (in thousands)** | **Percent change, 1990-2019** | **Age-standardized rate (per 100,000)**  **2019** | **Percent change, 1990-2019** | **Counts,**  **2019 (in thousands)** | **Percent change, 1990-2019** | **Age-standardized rate (per 100,000)**  **2019** | **Percent change, 1990-2019** |
| ***CENTRAL Asia*** | | | | | | | | | | | | |
| Kazakhstan | 19.8  [17.4 – 22.3] | 21.7  [7.3 to 37.6] | 192.0  [169.7 – 215.9] | -7.1  [-17.9 to 4.7] | 9.9  [8.6 – 11.2] | -3.4  [-15.6 to 10.3] | 98.0  [85.8 – 111.6] | -25.5  [-34.6 to -15.0] | 282.3  [245.4 – 324.0] | -4.5  [-17.2 to 9.8] | 2721.0  [2369.3 – 3117.9] | -27.0  [-36.6 to -16.2] |
| Kyrgyzstan | 3.8  [3.4 – 4.3] | 24.2  [9.6 to 40.3] | 137.8  [123.1 – 154.5] | -17.0  [-26.5 to -6.6] | 2.0  [1.7 – 2.2] | 6.5  [-8.8 to 21.9] | 75.4  [65.9 – 85.2] | -25.2  [-35.9 to -14.5] | 58.9  [51.1 – 67.2] | 4.7  [-10.3 to 20.9] | 2055.2  [1788.4 – 2342.2] | -32.1  [-41.9 to -21.9] |
| Tajikistan | 4.4  [3.6 – 5.2] | 71.8  [42.5 to 111.7] | 146.9  [125.1 – 175.4] | 0.1  [-15.5 to 21.6] | 2.5  [2.1 – 3.1] | 59.1  [28.4 to 101.7] | 97.0  [80.8 – 117.5] | 0.7  [-18.0 to 26.8] | 86.4  [70.4 – 106.7] | 60.4  [28.2 to 104.2] | 2586.4  [2129.1 – 3171.4] | -12.4  [-29.3 to 10.6] |
| Turkmenistan | 3.3  [2.8 – 4.1] | 71.6  [42.7 to 111.1] | 143.6  [120.3 – 173.1] | -7.8  [-22.6 to 12.0] | 1.7  [1.4 – 2.1] | 41.2  [13.5 to 76.3] | 75.5  [61.5 – 93.8] | -26.0  [-40.0 to -8.0] | 56.8  [45.4 – 71.6] | 42.6  [11.5 to 80.5] | 2362.0  [1887.8 – 2962.1] | -21.8  [-38.1 to -2.4] |
| Uzbekistan | 22.7  [19.4 – 26.2] | 123.8  [90.3 to 161.5] | 171.9  [149.3 – 196.2] | 21.8  [5.5 to 39.6] | 11.3  [9.5 – 13.2] | 90.4  [59.4 to 123.9] | 98.3  [84.6 – 112.8] | 13.7  [-2.9 to 30.9] | 395.0  [329.7 – 464.5] | 98.1  [64.4 to 133.8] | 2753.9  [2326.9 – 3213.8] | 4.6  [-12.1 to 22.2] |
| ***EASTERN Asia*** | | | | | | | | | | | | |
| China | 1897.5  [1552.0 – 2295.7] | 157.7  [98.4 to 235.6] | 191.9  [157.1 – 230.9] | 19.2  [-8.0 to 53.9] | 965.7  [776.2 – 1184.5] | 70.9  [31.4 to 121.7] | 95.8  [77.3 – 116.9] | -27.0  [-43.6 to -5.8] | 23867.0  [19203.8 – 29411.9] | 37.4  [5.4 to 78.5] | 2402.1  [1953.2 – 2937.9] | -33.7  [-48.6 to -14.2] |
| Japan | 383.7  [303.4 – 460.4] | 97.0  [58.9 to 134.7] | 237.6  [190.3 – 287.2] | 10.4  [-10.1 to 33.1] | 184.2  [144.9 – 206.2] | 87.9  [58.2 to 104.9] | 82.0  [70.3 – 88.5] | -21.6  [-28.4 to -17.5] | 2937.4  [2512.2 – 3186.1] | 28.8  [14.1 to 37.3] | 1941.6  [1769.9 – 2052.9] | -24.2  [-28.5 to -21.2] |
| Mongolia | 3.4  [2.7 – 4.3] | 119.6  [71.7 to 185.4] | 259.8  [208.7 – 323.4] | 0.8  [-20.2 to 28.7] | 2.5  [2.0 – 3.2] | 103.3  [55.9 to 166.6] | 219.5  [172.0 – 273.2] | 0.2  [-21.5 to 29.9] | 70.4  [54.1 – 91.3] | 99.6  [49.8 to 166.3] | 5017.1  [3880.6 – 6405.5] | -11.4  [-32.2 to 16.9] |
| North Korea | 25.7  [20.6 – 32.3] | 53.1  [12.0 to 102.2] | 145.8  [115.4 – 185.6] | -9.4  [-34.1 to 20.2] | 18.2  [14.8 – 22.6] | 64.1  [23.3 to 110.2] | 99.3  [80.1 – 123.5] | -12.3  [-33.7 to 11.6] | 493.0  [388.2 – 624.6] | 37.5  [-0.4 to 82.3] | 2845.3  [2234.2 – 3638.1] | -15.4  [-38.9 to 12.4] |
| South Korea | 94.2  [79.5 – 110.2] | 218.8  [166.6 to 274.6] | 209.9  [177.6 – 244.9] | 29.7  [8.5 to 52.3] | 40.4  [35.3 – 44.0] | 110.6  [83.0 to 130.6] | 82.2  [72.7 – 89.3] | -27.1  [-35.0 to -20.2] | 837.9  [760.1 – 902.4] | 41.9  [27.7 to 54.0] | 1889.3  [1729.5 – 2028.5] | -38.3  [-44.3 to -33.0] |
| Taiwan | 46.7  [36.8 – 60.6] | 247.4  [174.8 to 346.3] | 241.6  [190.2 – 313.3] | 47.0  [15.3 to 90.4] | 22.0  [17.5 – 28.0] | 196.4  [137.2 to 276.0] | 105.3  [83.7 – 134.2] | 6.3  [-15.3 to 35.0] | 498.2  [392.8 – 645.8] | 118.8  [72.8 to 181.7] | 2578.0  [2021.6 – 3341.7] | -5.0[-25.7 to 22] |
| ***SOUTH-EASTERN Asia*** | | | | | | | | | | | | |
| Brunei Darussalam | 0.5  [0.5 – 0.6] | 219.1  [162.1 to 294.5] | 309.9  [271.6 – 355.9] | 13.5  [-4.1 to 35.5] | 0.3  [0.2 – 0.3] | 173.8  [129.3 to 227.3] | 178.9  [160.5 – 199.8] | -4.1  [-18.0 to 12.3] | 8.2  [7.1 – 9.5] | 144.1  [99.9 to 199.5] | 4463.3  [3927.9 – 5081.9] | -9.3  [-24.1 to 8.1] |
| Cambodia | 10.2  [8.2 – 12.4] | 148.3  [84.6 to 242.9] | 138.9  [112.7 – 167.0] | 11.5  [-15.0 to 43.9] | 7.1  [5.7 – 8.5] | 132.4  [77.0 to 202.3] | 102.4  [83.8 – 121.3] | -0.5  [-22.0 to 25.0] | 217.7  [173.6 – 267.5] | 82.6  [34.2 to 156.1] | 2870.2  [2313.5 – 3503.9] | -10.3  [-32.5 to 18.4] |
| Indonesia | 171.4  [129.9 – 219.2] | 133.9  [79.8 to 202.9] | 140.6  [106.7 – 178.3] | 21.3  [-5.9 to 53.8] | 112.9  [82.5 – 146.5] | 122.6  [68.4 to 183.0] | 100.8  [73.7 – 128.6] | 12.5  [-14.1 to 41.2] | 3498.3  [2582.8 – 4552.3] | 86.0  [41.9 to 140.6] | 2762.3  [2040.3 – 3573.3] | 0.4  [-22.6 to 28.2] |
| Lao PDR | 3.5  [2.6 – 4.5] | 78.6  [28.1 to 152.9] | 134.2  [102.4 – 171.1] | -7.8  [-31.3 to 25.7] | 2.4  [1.8 – 3.1] | 65.1  [20.7 to 127.2] | 102.7  [79.7 – 129.6] | -15.1  [-35.9 to 14.8] | 80.1  [59.6 – 104.8] | 46.9  [2.6 to 114.8] | 2896.7  [2191.0 – 3746.3] | -23.0  [-44.6 to 7.3] |
| Malaysia | 27.1  [21.6 – 33.5] | 236.6  [166.5 to 323.2] | 195.3  [156.7 – 240.3] | 31.0  [3.6 to 64.3] | 14.6  [11.7 – 17.8] | 171.0  [113.6 to 239.1] | 113.6  [91.5 – 138.1] | 3.4  [-18.9 to 29.1] | 407.6  [321.7 – 504.1] | 135.6  [85.0 to 193.9] | 2870.6  [2282.2 – 3539.8] | -3.9  [-24.5 to 20.1] |
| Myanmar | 36.8  [29.5 – 46.3] | 52.7  [10.0 to 113.1] | 136.0  [110.1 – 171.0] | -17.3  [-38.1 to 11.0] | 26.6  [21.8 – 33.3] | 49.7  [13.6 to 99.9] | 103.1  [85.8 – 128.4] | -21.6  [-39.5 to 2.7] | 794.9  [622.4 – 1023.9] | 20.9  [-13.0 to 70.1] | 2853.1  [2258.4 – 3642.5] | -32.4  [-50.3 to -7.5] |
| Philippines | 65.9  [50.8 – 84.3] | 141.5  [83.3 to 224.2] | 143.9  [111.7 – 184.5] | -1.8  [-25.4 to 31.9] | 40.3  [31.4 – 51.4] | 128.7  [73.7 to 201.4] | 95.0  [74.2 – 120.0] | -13.2  [-33.3 to 13.9] | 1291.7  [994.5 – 1654.3] | 109.6  [59.8 to 174.5] | 2724.1  [2107.3 – 3481.8] | -9.8  [-31.2 to 18.4] |
| Singapore | 8.6  [7.1 – 10.6] | 206.0  [151.9 to 276.9] | 220.6  [182.2 – 270.2] | 0.7  [-17.3 to 23.3] | 3.1  [2.8 – 3.4] | 120.6  [99.6 to 135.1] | 78.8  [70.2 – 84.3] | -34.5  [-40.3 to -30.2] | 73.5  [67.6 – 78.4] | 81.1  [65.9 to 93.7] | 1892.9  [1740.6 – 2020.9] | -38.4  [-43.6 to -34.2] |
| Thailand | 80.2  [60.5 – 104.4] | 147.3  [83.9 to 229.1] | 153.0  [115.2 – 198.2] | 0.1  [-25.5 to 33.4] | 50.1  [38.5 – 64.3] | 127.8  [71.8 to 202.9] | 91.9  [70.7 – 118.0] | -19.0  [-38.8 to 7.5] | 1238.7  [929.4 – 1611.6] | 78.9  [31.5 to 139.5] | 2377.1  [1781.2 – 3091.2] | -23.2  [-43.4 to 2.7] |
| Timor-Leste | 0.5  [0.4 – 0.7] | 146.2  [61.8 to 269.4] | 123.8  [91.9 – 155.6] | 12.5  [-21.2 to 48.9] | 0.4  [0.3 – 0.5] | 155.3  [77.5 to 249.1] | 95.3  [74.9 – 118.7] | 3.5  [-23.8 to 33.8] | 11.9  [8.0 – 15.3] | 94.6  [16.5 to 202.9] | 2575.5  [1836.7 – 3299.0] | -4.3  [-35.7 to 30.3] |
| Viet Nam | 86.8  [67.4 – 109.0] | 227.9  [142.1 to 335.3] | 160.0  [124.7 – 199.1] | 46.5  [9.0 to 93.3] | 48.7  [38.6 – 60.0] | 145.5  [85.6 to 221.9] | 93.1  [74.0 – 114.6] | 10.7  [-16.4 to 43.9] | 1357.4  [1058.4 – 1703.8] | 130.7  [68.7 to 209.6] | 2485.1  [1943.9 – 3098.1] | 5.3  [-22.6 to 41.4] |
| ***SOUTHERN Asia*** | | | | | | | | | | | | |
| Afghanistan | 16.2  [12.6 – 21.3] | 127.8  [79.2 to 196.7] | 185.4  [146.4 – 235.4] | 2.8  [-20.4 to 33.9] | 12.3  [9.7 – 15.9] | 107.8  [59.9 to 175.3] | 162.7  [128.0 – 207.2] | 0.1  [-23.5 to 31.3] | 476.8  [371.8 – 630.7] | 124.0  [73.1 to 200.0] | 4669.8  [3653.3 – 6012.9] | -5.4  [-28.6 to 29.0] |
| Bangladesh | 64.8  [50.4 – 80.9] | 137.6  [73.1 to 243.0] | 95.0  [74.4 – 118.8] | -2.2  [-26.5 to 33.9] | 45.1  [35.1 – 56.7] | 120.7  [61.7 to 208.5] | 70.7  [55.2 – 89.2] | -13.8  [-34.9 to 15.8] | 1413.8  [1107.9 – 1767.3] | 74.6  [25.2 to 158.4] | 1994.5  [1561.1 – 2481.2] | -20.5  [-41.8 to 12.0] |
| Bhutan | 0.3  [0.2 – 0.4] | 120.4  [52.5 to 259.8] | 105.7  [80.4 – 133.0] | 17.4  [-14.3 to 76.3] | 0.2  [0.2 – 0.3] | 102.6  [45.8 to 213.6] | 79.5  [61.9 – 99.4] | 4.1  [-21.2 to 48.8] | 6.3  [4.6 – 8.1] | 59.7  [5.8 to 171.7] | 2086.3  [1552.7 – 2657.2] | -8.2  [-35.2 to 45.4] |
| India | 649.6  [525.7 – 784.3] | 179.0  [121.2 to 245.7] | 106.6  [86.3 – 128.0] | 18.2  [-6.4 to 46.9] | 461.0  [372.5 – 566.6] | 164.8  [107.6 to 233.6] | 79.3  [64.3 – 97.4] | 5.3  [-18.2 to 33.6] | 13779.1  [11095 – 16945.4] | 118.7  [71.7 to 175.2] | 2193.9  [1771.2 – 2694.7] | 0.6  [-21.5 to 26.2] |
| Iran | 57.1  [52.9 – 61.1] | 212.4  [174.1 to 258.8] | 146.7  [135.4 – 157.2] | 27.7  [13.4 to 44.2] | 27.8  [25.6 – 29.9] | 159.1  [128.4 to 199.2] | 78.1  [71.3 – 84.3] | -4.3  [-16.6 to 10.2] | 798.2  [732.4 – 854.3] | 98.6  [70.9 to 133.3] | 2009.9  [1846.1 – 2155.8] | -10.0  [-20.4 to 2.8] |
| Maldives | 0.2  [0.2 – 0.2] | 167.7  [95.6 to 335.6] | 125.6  [106.5 – 147.2] | -10.5  [-31.8 to 35.8] | 0.1  [0.1 – 0.1] | 110.4  [55.4 to 227.7] | 70.4  [59.4 – 82.5] | -34.9  [-49.5 to -4.6] | 2.6  [2.2 – 3.0] | 52.8  [10.5 to 148.6] | 1655.0  [1397.6 – 1944.7] | -44.2  [-58.4 to -13.7] |
| Nepal | 13.9  [11.0 – 17.4] | 150.7  [82.3 to 237.4] | 111.3  [88.1 – 137.8] | 15.5  [-14.1 to 52.1] | 10.1  [8.0 – 12.5] | 137.6  [76.2 to 211.1] | 86.5  [68.7 – 106.5] | 6.7  [-20.3 to 38.9] | 308.9  [241.1 – 385.1] | 90.7  [38.1 to 162.5] | 2360.8  [1856.7 – 2928.8] | -6.0  [-31.1 to 24.7] |
| Pakistan | 135.8  [105.4 – 176.3] | 228.7  [149.0 to 342.8] | 208.3  [165.1 – 268.2] | 52.1  [16.5 to 105.1] | 91.4  [71.9 – 117.7] | 183.0  [112.9 to 285.0] | 157.8  [125.6 – 199.3] | 35.7  [3.5 to 81.2] | 3219.5  [2482.3 – 4130.0] | 189.7  [117.4 to 294.4] | 4511.2  [3525.9 – 5796.0] | 35.5  [1.6 to 84.0] |
| Sri Lanka | 16.7  [12.6 – 21.8] | 188.3  [112.3 to 278.5] | 121.9  [92.4 – 159.6] | 26.5  [-6.4 to 65.8] | 9.3  [7.1 – 12.0] | 139.1  [78.2 to 209.3] | 68.4  [52.4 – 88.0] | -5.8  [-29.8 to 21.1] | 238.6  [179.9 – 312.3] | 93.1  [41.6 to 153.3] | 1741.0  [1317.7 – 2278.2] | -9.3  [-33.5 to 19.1] |
| ***Western Asia*** | | | | | | | | | | | | |
| Armenia | 4.8  [4.1 –  5.5] | 49.8  [28.3 to 74.8] | 214.8  [183.6 – 248.3] | 7.2  [-8.5 to 25.0] | 2.6  [2.2 – 3.0] | 39.4  [17.9 to 63.5] | 110.0  [92.7 – 128.2] | -6.4  [-21.2 to 9.7] | 63.5  [53.0 – 74.8] | 8.5  [-9.4 to 28.6] | 2927.7  [2451.8 – 3436.7] | -18.0  [-31.4 to -3.0] |
| Azerbaijan | 9.3  [7.8 – 11.1] | 91.7  [57.9 to 128.9] | 175.1  [147.0 – 207.8] | 11.2  [-8.3 to 32.6] | 5.0  [4.1 – 6.1] | 70.7  [35.9 to 109.6] | 101.3  [82.6 – 124.4] | 4.7  [-17.2 to 28.9] | 153.1  [124.4 – 185.2] | 59.7  [28.3 to 94.9] | 2769.3  [2273.2 – 3347.3] | -7.4  [-25.2 to 11.9] |
| Bahrain | 0.8  [0.7 – 1.0] | 451.5  [339.5 to 597.0] | 192.6  [160.6 – 228.9] | 18.4  [-4.4 to 46.2] | 0.3  [0.3 – 0.4] | 245.5  [174.8 to 332.7] | 100.5  [84.2 – 117.9] | -19.3  [-34.5 to -1.0] | 10.6  [8.6 – 12.9] | 227.1  [157.6 to 315.3] | 2305.2  [1904.5 – 2758.1] | -24.9  [-40.4 to -6.2] |
| Cyprus | 2.9  [2.5 – 3.3] | 229.9  [180.4 to 286.4] | 298.8  [260.2 – 342.1] | 41.6  [19.6 to 66.0] | 1.0  [0.9 – 1.1] | 126.7  [89.6 to 165.0] | 98.3  [87.6 – 108.4] | -10.2  [-24.9 to 5.7] | 21.5  [19.2 – 24.0] | 102.5  [73.4 to 134.2] | 2242.4  [2003.7 – 2505.3] | -11.8  [-24.6 to 2.9] |
| Georgia | 6.6  [5.7 – 7.6] | -12.2  [-25.9 to 2.8] | 217.6  [186.2 – 253.1] | 0.2  [-16.5 to 17.8] | 3.5  [3.0 – 4.1] | -7.8  [-22.8 to 9.0] | 106.2  [90.0 – 124.2] | -1.1  [-17.5 to 17.5] | 89.0  [74.7 – 104.2] | -21.9  [-35.2 to -6.5] | 3107.8  [2608.9 – 3648.4] | -7.1  [-23.2 to 11.8] |
| Iraq | 22.9  [17.7 – 29.4] | 314.4  [206.7 to 466.1] | 158.9  [124.7 – 200.1] | 48.1  [10.0 to 101.4] | 11.3  [8.8 – 14.3] | 231.8  [143.5 to 356.2] | 89.4  [70.9 – 110.2] | 18.3  [-12.1 to 58.6] | 393.4  [302.9 – 507.0] | 204.2  [118.8 to 326.2] | 2614.1  [2020.2 – 3336.4] | 12.0  [-18.8 to 55.6] |
| Israel | 16.8  [13.8 – 20.5] | 154.8  [109.4 to 211.7] | 285.4  [231.6 – 349.8] | 9.7  [-10.3 to 34.5] | 6.9  [6.2 – 7.3] | 101.7  [87.2 to 113.4] | 106.4  [97.1 – 112.3] | -20.8  [-25.4 to -16.8] | 142.6  [132.3 – 151.5] | 71.6  [60.1 to 82.0] | 2477.9  [2314.3 – 2628.4] | -24.9  [-29.6 to -20.6] |
| Jordan | 5.8  [4.7 – 7.3] | 398.5  [284.9 to 553.1] | 158.6  [129.2 – 196.3] | 18.1  [-8.4 to 54.6] | 2.5  [2.0 – 3.1] | 269.5  [185.6 to 392.1] | 82.0  [67.0 – 101.4] | -12.7  [-31.9 to 14.8] | 80.6  [64.5 – 101.5] | 224.8  [147.8 to 335.0] | 2104.4  [1696.4 – 2633.1] | -19.3  [-38.0 to 8.1] |
| Kuwait | 2.0  [1.6 – 2.4] | 268.0  [203.8 to 351.4] | 140.5  [116.3 – 168.6] | -7.6  [-23.3 to 11.3] | 0.6  [0.5 – 0.7] | 184.4  [135.2 to 246.5] | 58.3  [47.8 – 69.8] | -29.4  [-41.0 to -14.7] | 20.1  [16.5 – 24.4] | 146.7  [103.7 to 202.5] | 1405.7  [1163.1 – 1693] | -36.1  [-47.1 to -22.6] |
| Lebanon | 8.6  [7.0 – 10.9] | 316.5  [222.3 to 442.5] | 301.5  [246.9 – 381.4] | 80.2  [39.8 to 134.4] | 3.5  [2.9 – 4.6] | 176.5  [119.1 to 264.3] | 123.9  [103.1 – 161.7] | 11.3  [-11.7 to 47.2] | 92.8  [75.3 – 117.5] | 136.9  [81.4 to 207.8] | 3277.4  [2663.5 – 4144.9] | 7.7  [-17.7 to 39.8] |
| Oman | 1.5  [1.3 – 1.8] | 312.2  [210.9 to 462.5] | 173.5  [149.7 – 197.8] | 67.8  [28.7 to 132.4] | 0.6  [0.5 – 0.7] | 158.7  [96.4 to 262.9] | 85.5  [73.8 – 97.7] | 16.2  [-11.9 to 65.6] | 19.0  [15.8 – 22.1] | 148.8  [84.0 to 241.7] | 2061.4  [1743.7 – 2370.3] | 7.4  [-18.9 to 51.6] |
| Palestine | 2.6  [2.2 – 3.0] | 234.9  [158.6 to 342.4] | 181.6  [155.8 – 209.8] | 33.4  [3.6 to 76.9] | 1.4  [1.2 – 1.6] | 177.2  [115.2 to 275.5] | 109.2  [93.4 – 125.4] | 11.9  [-13.5 to 51.8] | 42.6  [36.6 – 49.2] | 162.6  [102.8 to 250.6] | 2827.9  [2422.5 – 3271.2] | 5.4  [-19.0 to 43.0] |
| Qatar | 0.9  [0.7 – 1.1] | 873.5  [621.5 to 1193.1] | 318.4  [261.7 – 383.0] | 78.6  [37.1 to 134.2] | 0.3  [0.2 – 0.3] | 435.2  [301.6 to 613.6] | 166.1  [138.5 – 196.0] | 24.7  [-4.8 to 64.3] | 10.0  [7.8 – 12.5] | 427.9  [288.7 to 617.2] | 3311.4  [2679.8 – 4014.2] | 2.6  [-21.5 to 34.3] |
| Saudi Arabia | 16.8  [13.0 – 21.7] | 635.6  [415.4 to 925.5] | 157.6  [125.5 – 197.0] | 114.1  [55.3 to 193.4] | 5.7  [4.5 – 7.3] | 230.9  [137.2 to 370.8] | 72.5  [58.3 – 90.5] | 12.7  [-17.7 to 62.3] | 213.4  [164.7 – 274.6] | 247.5  [147.6 to 398.0] | 1943.7  [1537.6 – 2452.6] | 12.6  [-19.2 to 60.3] |
| Syrian Arab Republic | 7.0  [5.4 – 9.2] | 122.5  [59.4 to 223.8] | 110.6  [86.2 – 142.8] | 22.3  [-11.7 to 71.3] | 3.8  [2.9 – 5.0] | 93.9  [38.2 to 177.1] | 69.1  [53.8 – 87.4] | 0.6  [-26.7 to 42.8] | 119.4  [90.6 – 158.2] | 54.1  [6.9 to 129.0] | 1776.8  [1364.6 – 2327.0] | -10.9  [-36.9 to 28.6] |
| Turkey | 74.9  [61.5 – 90.4] | 159.2  [104.5 to 231.0] | 163.1  [134.5 – 196.3] | 18.5  [-5.4 to 49.0] | 38.6  [31.5 – 46.9] | 97.6  [54.3 to 152.2] | 84.2  [68.8 – 102.0] | -15.8  [-33.9 to 6.6] | 973.9  [784.4 – 1195.0] | 50.5  [16.0 to 95.6] | 2124.5  [1719.9 – 2595.0] | -25.8  [-43.1 to -4.6] |
| United Arab Emirates | 3.2  [2.4 – 4.3] | 775.5  [516.9 to 1104.7] | 193.1  [153.5 – 244.6] | 7.4  [-19.4 to 46.0] | 1.4  [1.1 – 1.9] | 551.1  [366.1 to 812.2] | 122.0  [98.6 – 151.6] | -13.5  [-34.9 to 19.2] | 56.2  [41.6 – 74.9] | 562.4  [371.2 to 822.1] | 3142.3  [2474.1 – 4000.8] | -12.1  [-35.1 to 22.6] |
| Yemen | 8.7  [6.6 – 11.4] | 204.7  [115.0 to 348.5] | 102.9  [80.1 – 134.5] | 22.1  [-6.6 to 66.4] | 5.7  [4.3 – 7.5] | 186.4  [109.1 to 300.2] | 76.9  [59.7 – 99.8] | 11.2  [-14.7 to 50.1] | 201.2  [148.0 – 265.5] | 168.3  [83.2 to 302.4] | 2154.1  [1634.3 – 2862.7] | 9.0  [-20.5 to 55.3] |

UI = Uncertainty Interval. Data Source: Global Burden of Disease, Injuries and Risk Factors 2019 Study

**Supplementary Table S13 Country-wise Decomposition Analysis of Cancer Trends in Asia, 1990 to 2019**

|  | **Change in Incident Cases, 1990 to 2019 (%)** | | | | | | | | | | | |
| --- | --- | --- | --- | --- | --- | --- | --- | --- | --- | --- | --- | --- |
|  | **Both Sexes** | | | | **Males** | | | | **Females** | | | |
| **Country** | **Population Growth** | **Change in Age Structure** | **Change in Incidence Rates** | **Overall Change (%)** | **Due to Population Growth** | **Change in Age Structure** | **Change in Incidence Rates** | **Overall Change (%)** | **Population Growth** | **Change in Age Structure** | **Change in Incidence Rates** | **Overall Change (%)** |
| Afghanistan | 235.2 | -113.6 | -19.0 | 102.6 | 247.7 | -157.4 | -17.0 | 73.3 | 223.0 | -83.6 | -11.6 | 127.8 |
| Armenia | -11.6 | 50.9 | 13.5 | 52.8 | -12.9 | 56.3 | 12.7 | 56.1 | -10.3 | 47.2 | 12.8 | 49.8 |
| Azerbaijan | 40.2 | 45.9 | 1.5 | 87.6 | 43.7 | 54.6 | -14.4 | 83.9 | 36.8 | 40.3 | 14.6 | 91.7 |
| Bahrain | 184.0 | 254.4 | -25.9 | 412.5 | 204.8 | 287.3 | -110.1 | 382.0 | 155.2 | 214.3 | 82.0 | 451.5 |
| Bangladesh | 46.0 | 93.9 | -29.2 | 110.8 | 40.1 | 93.4 | -42.7 | 90.8 | 52.3 | 94.9 | -9.5 | 137.6 |
| Bhutan | 23.2 | 77.7 | 33.3 | 134.2 | 21.6 | 92.3 | 36.5 | 150.3 | 25.0 | 68.2 | 27.2 | 120.4 |
| Brunei Darussalam | 69.1 | 109.0 | 15.7 | 193.8 | 67.4 | 103.8 | -4.9 | 166.4 | 70.9 | 118.2 | 30.0 | 219.1 |
| Cambodia | 60.0 | 73.2 | 11.7 | 144.9 | 64.4 | 66.1 | 10.6 | 141.1 | 56.0 | 72.3 | 20.0 | 148.3 |
| China | 20.2 | 97.7 | 52.2 | 170.1 | 18.8 | 103.0 | 57.1 | 178.9 | 21.6 | 92.6 | 43.5 | 157.7 |
| Cyprus | 68.8 | 66.9 | 120.6 | 256.3 | 65.8 | 73.7 | 143.5 | 283.0 | 71.9 | 63.5 | 94.5 | 229.9 |
| Georgia | -33.5 | 20.5 | 11.0 | -2.0 | -32.9 | 24.8 | 18.6 | 10.4 | -34.0 | 18.4 | 3.4 | -12.2 |
| India | 62.5 | 69.7 | 28.6 | 160.9 | 42.1 | 46.6 | 11.3 | 142.8 | 65.1 | 71.6 | 42.3 | 179.0 |
| Indonesia | 40.0 | 59.2 | 47.6 | 146.8 | 41.5 | 58.6 | 64.8 | 164.9 | 38.4 | 58.4 | 37.0 | 133.9 |
| Iran | 44.0 | 99.7 | 60.0 | 203.7 | 43.1 | 100.6 | 53.0 | 196.8 | 44.9 | 97.5 | 70.0 | 212.4 |
| Iraq | 139.4 | 41.9 | 91.2 | 272.4 | 140.3 | 36.8 | 57.0 | 234.0 | 138.4 | 47.1 | 128.8 | 314.4 |
| Israel | 87.6 | 46.2 | 32.7 | 166.5 | 49.5 | 25.6 | 24.9 | 179.5 | 86.5 | 44.9 | 23.4 | 154.8 |
| Japan | 1.5 | 86.9 | 8.2 | 96.6 | 0.6 | 102.9 | -7.2 | 96.4 | 2.4 | 78.0 | 16.6 | 97.0 |
| Jordan | 208.4 | 140.3 | 86.6 | 435.4 | 214.6 | 149.8 | 113.1 | 477.6 | 201.6 | 130.0 | 66.9 | 398.5 |
| Kazakhstan | 12.4 | 23.9 | -22.9 | 13.3 | 12.3 | 33.4 | -41.0 | 4.8 | 12.4 | 18.5 | -9.2 | 21.7 |
| Kuwait | 151.6 | 117.6 | 38.4 | 307.7 | 135.2 | 118.6 | 94.4 | 348.2 | 173.6 | 148.5 | -54.0 | 268.0 |
| Kyrgyzstan | 46.5 | 11.0 | -40.9 | 16.6 | 48.3 | 18.3 | -57.8 | 8.7 | 44.7 | 7.4 | -28.0 | 24.2 |
| Lao PDR | 72.4 | 31.2 | -32.0 | 71.6 | 76.5 | 27.9 | -40.6 | 63.9 | 68.5 | 33.4 | -23.3 | 78.6 |
| Lebanon | 58.1 | 64.9 | 162.1 | 285.1 | 54.0 | 57.3 | 145.5 | 256.8 | 62.2 | 69.4 | 184.9 | 316.5 |
| Malaysia | 77.3 | 92.4 | 70.9 | 240.6 | 81.7 | 100.1 | 63.5 | 245.3 | 72.8 | 86.3 | 77.5 | 236.6 |
| Maldives | 124.6 | 101.5 | -37.1 | 189.0 | 163.9 | 50.1 | 0.2 | 214.2 | 83.2 | 128.6 | -44.1 | 167.7 |
| Mongolia | 57.3 | 65.5 | -7.1 | 115.7 | 55.3 | 57.0 | 0.1 | 112.4 | 59.2 | 71.8 | -11.5 | 119.6 |
| Myanmar | 33.0 | 50.9 | -23.6 | 60.3 | 29.1 | 45.7 | -4.0 | 70.8 | 36.9 | 54.5 | -38.6 | 52.7 |
| Nepal | 55.7 | 57.5 | 22.6 | 135.8 | 48.0 | 54.9 | 18.3 | 121.2 | 63.4 | 62.0 | 25.2 | 150.7 |
| North Korea | 24.6 | 52.0 | -14.4 | 62.3 | 31.0 | 63.5 | -22.4 | 72.2 | 18.8 | 46.5 | -12.2 | 53.1 |
| Oman | 135.9 | 34.8 | 111.2 | 281.9 | 160.6 | 25.0 | 73.1 | 258.8 | 101.3 | 48.9 | 162.0 | 312.2 |
| Pakistan | 98.6 | 0.6 | 84.1 | 183.3 | 94.3 | -8.7 | 56.2 | 141.8 | 103.3 | 10.3 | 115.0 | 228.7 |
| Palestine | 139.4 | 29.5 | 44.5 | 213.5 | 142.1 | 31.8 | 18.9 | 192.8 | 136.7 | 24.9 | 73.3 | 234.9 |
| Philippines | 77.2 | 61.8 | -10.2 | 128.8 | 78.4 | 55.8 | -19.4 | 114.8 | 75.9 | 65.4 | 0.2 | 141.5 |
| Qatar | 543.5 | 198.3 | 242.0 | 983.8 | 618.7 | 242.4 | 209.5 | 1070.6 | 392.2 | 182.9 | 298.5 | 873.5 |
| Saudi Arabia | 122.7 | 76.0 | 323.3 | 522.0 | 132.5 | 68.2 | 235.1 | 435.8 | 110.2 | 91.5 | 433.9 | 635.6 |
| Singapore | 86.0 | 148.8 | -38.0 | 196.9 | 88.8 | 180.8 | -81.1 | 188.5 | 83.1 | 122.0 | 0.9 | 206.0 |
| South Korea | 20.4 | 146.0 | 61.6 | 228.0 | 21.2 | 186.3 | 27.7 | 235.2 | 19.7 | 120.7 | 78.4 | 218.8 |
| Sri Lanka | 26.9 | 86.3 | 60.0 | 173.2 | 22.0 | 74.1 | 61.6 | 157.6 | 31.9 | 94.4 | 62.1 | 188.3 |
| Syrian Arab Republic | 12.4 | 75.1 | 28.7 | 116.1 | 7.0 | 79.8 | 23.9 | 110.7 | 18.0 | 70.3 | 34.1 | 122.5 |
| Taiwan | 15.8 | 102.5 | 129.8 | 248.0 | 11.1 | 90.5 | 146.8 | 248.5 | 20.8 | 111.2 | 115.4 | 247.4 |
| Tajikistan | 76.6 | 16.2 | -33.6 | 59.2 | 79.5 | 21.0 | -53.2 | 47.2 | 73.7 | 14.7 | -16.6 | 71.8 |
| Thailand | 23.3 | 131.1 | 2.5 | 157.0 | 21.3 | 139.8 | 5.5 | 166.6 | 25.2 | 122.7 | -0.6 | 147.3 |
| Timor-Leste | 70.5 | 70.3 | 26.2 | 167.1 | 67.2 | 84.2 | 43.0 | 194.5 | 74.0 | 58.7 | 13.6 | 146.2 |
| Turkey | 36.1 | 88.3 | 31.0 | 155.4 | 35.6 | 92.5 | 24.7 | 152.8 | 36.6 | 81.1 | 41.5 | 159.2 |
| Turkmenistan | 37.2 | 54.5 | -23.1 | 68.6 | 41.9 | 59.7 | -36.2 | 65.4 | 32.6 | 51.3 | -12.3 | 71.6 |
| United Arab Emirates | 393.7 | 347.1 | 229.5 | 970.2 | 454.1 | 357.8 | 272.7 | 1084.6 | 282.9 | 329.3 | 163.3 | 775.5 |
| Uzbekistan | 60.8 | 31.6 | 7.7 | 100.1 | 62.2 | 35.7 | -22.7 | 75.3 | 59.3 | 30.1 | 34.4 | 123.8 |
| Viet Nam | 41.8 | 85.9 | 96.3 | 224.1 | 44.9 | 86.5 | 88.9 | 220.3 | 38.9 | 84.4 | 104.6 | 227.9 |
| Yemen | 129.5 | 31.3 | 33.5 | 194.3 | 127.7 | 42.3 | 14.6 | 184.6 | 131.2 | 27.1 | 46.4 | 204.7 |

Source: Author’s Calculations using estimates from Global Burden of Diseases, Injuries and Risk Factors 2019 Study

**Supplementary Table S14 Cancer Deaths attributable to Risk Factors, 1990-2019**

|  | **Deaths**  **(in thousands)**  **Both Sexes** | | | **Deaths**  **(in Thousands)**  **Males** | | | **Deaths**  **(in Thousands)**  **Females** | | |
| --- | --- | --- | --- | --- | --- | --- | --- | --- | --- |
| **Risk Factors** | **1990** | **2019** | **Percent Change, 1990-2019** | **1990** | **2019** | **Percent Change, 1990-2019** | **1990** | **2019** | **Percent Change, 1990-2019** |
| ***Behavioural risks*** | | | | | | | | | |
| Alcohol use | 126.2  [107.7 – 148.0] | 268.9  [227.8 – 312.7] | 113.0  [76.6 to 155.4] | 106.9  [88.5 – 127.5] | 234.9  [196.2 – 277.1] | 119.7  [77.1 to 171.8] | 19.3  [15.9 – 22.8] | 33.9  [28.1 – 40.0] | 75.7  [47.4 to 110.9] |
| Chewing tobacco | 22.6  [18.1 – 27.8] | 51.9  [40.4 – 64.5] | 129.1  [87.0 to 176.9] | 13.8  [9.6 – 18.5] | 28.2  [19.3 – 38.3] | 104.2  [52.6 to 168.0] | 8.8  [6.8 – 11.3] | 23.6  [17.8 – 30.4] | 168.1  [115.0 to 225.0] |
| Diet high in processed meat | 3.1  [1.1 – 4.6] | 9.3  [2.9 – 14.6] | 201.9  [131.8 to 259.2] | 1.6  [0.6 – 2.4] | 5.2  [1.6 – 8.2] | 223.6  [137.3 to 296.9] | 1.5  [0.5 – 2.2] | 4.1  [1.3 – 6.5] | 178.3  [114.5 to 231.3] |
| Diet high in red meat | 8.2  [3.3 – 15.5] | 31.6  [13.7 – 55.1] | 284.9  [218.9 to 449.0] | 2.9  [0.6 – 6.7] | 14.3  [3.4 – 28.2] | 389.2  [258.2 to 816.4] | 5.3  [2.3 – 9.1] | 17.3  [8.8 – 27.9] | 227.3  [171.0 to 341.9] |
| Diet high in sodium | 41.7  [1.0 – 158.3] | 55.7  [1.4 – 214.2] | 33.4  [9.7 to 54.1] | 26.6  [0.6 – 100.2] | 37.7  [0.9 – 142.4] | 41.6  [12.9 to 73.0] | 15.1  [0.4 – 58.1] | 18.0  [0.5 – 71.5] | 19.0  [-7.9 to 41.9] |
| Diet low in calcium | 35.5  [28.0 – 45.0] | 93.7  [68.2 – 123.9] | 164.3  [125.6 to 203.1] | 19.2  [15.1 – 24.6] | 55.0  [40.2 – 73.3] | 186.8  [130.2 to 245.3] | 16.3  [12.5 – 20.9] | 38.7  [27.7 – 51.5] | 137.8  [101.3 to 177.0] |
| Diet low in fiber | 5.4  [2.3 – 9.5] | 11.4  [4.8 – 21.1] | 109.3  [77.0 to 138.6] | 2.8  [1.2 – 4.8] | 6.1  [2.6 – 11.2] | 120.3  [75.5 to 164.3] | 2.7  [1.1 – 4.7] | 5.2  [2.2 – 9.6] | 97.7  [66.6 to 128.5] |
| Diet low in fruits | 65.1  [34.8 – 99.2] | 85.6  [44.4 – 136.8] | 31.4  [-8.6 to 75.9] | 43.8  [23.7 – 66.3] | 59.7  [29.6 – 97.4] | 36.4  [-7.0 to 88.8] | 21.3  [11.2 – 32.9] | 25.8  [13.9 – 39.2] | 21.3  [-16.3 to 69.3] |
| Diet low in milk | 33.9  [23.2 – 44.2] | 105.0  [69.7 – 137.6] | 209.4  [173.9 to 244.2] | 17.8  [12.1 – 23.4] | 60.2  [40.0 – 80.3] | 237.4  [181.1 to 291.7] | 16.1  [11.0 – 21.3] | 44.9  [29.3 – 59.2] | 178.4  [142.9 to 218.5] |
| Diet low in vegetables | 15.8  [1.4 – 33.1] | 7.9  [1.4 – 15.3] | -50.0  [-60.1 to 10.8] | 10.3  [0.8 – 21.9] | 5.0  [0.9 – 9.8] | -51.7  [-62.3 to 14.4] | 5.4  [0.5 – 11.3] | 2.9  [0.5 – 5.7] | -46.9  [-59.0 to 3.7] |
| Diet low in whole grains | 27.3  [10.5 – 36.4] | 85.2  [32.7 – 113.6] | 212.5  [176.4 to 248.4] | 14.5  [5.5 – 19.6] | 49.7  [19.0 – 67.4] | 241.8  [185.4 to 297.7] | 12.7  [4.9 – 16.9] | 35.5  [13.6 – 48.2] | 179.1  [141.3 to 218.2] |
| Drug use | 30.7  [22.7 – 39.8] | 46.2  [35.9 – 58.2] | 50.2  [23.4 to 84.0] | 18.0  [14.2 – 22.5] | 26.3  [20.8 – 32.4] | 46.2  [16.5 to 82.7] | 12.7  [7.9 – 18.7] | 19.8  [13.8 – 27.1] | 55.9  [14.9 to 112.6] |
| Low physical activity | 8.9  [3.2 – 16.7] | 29.3  [10.6 – 54.6] | 228.3  [186.3 to 275.9] | 3.5  [0.7 – 7.1] | 12.5  [3.0 – 25.4] | 259.7  [199.4 to 332.4] | 5.4  [2.3 – 9.6] | 16.7  [7.2 – 29.5] | 208.2  [164.5 to 259.4] |
| Secondhand smoke | 34.7  [22.3 – 49.8] | 90.9  [57.3 – 132.3] | 161.8  [120.0 to 206.6] | 16.4  [9.7 – 25.3] | 44.1  [25.1 – 67.9] | 168.2  [104.6 to 255.0] | 18.3  [11.7 – 25.9] | 46.8  [29.9 – 67.9] | 156.1  [114.4 to 206.5] |
| Smoking | 681.4  [607.2 – 756.9] | 1446.8  [1267.2 – 1643.2] | 112.3  [80.0 to 150.8] | 608.7  [535.5 – 684.1] | 1283.3  [1107.0 – 1474.9] | 110.8  [74.2 to 155.5] | 72.6  [62.4 – 84.4] | 163.6  [139.2 – 188.6] | 125.2  [88.6 to 168.2] |
| Unsafe sex |  |  |  |  |  |  | 87.1  [73.4 – 115.9] | 146.5  [118.9 – 170.8] | 68.2  [27.5 to 110.9] |
| ***Environmental/occupational risks*** | | | | | | | | | |
| Ambient particulate matter pollution | 44.5  [24.9 – 68.7] | 234.5  [170.9 – 302.8] | 426.6  [249.8 to 764.1] | 33.7  [18.7 – 52.5] | 165.6  [116.2 – 218.8] | 391.2  [218.0 to 719.1] | 10.8  [6.0 – 17.3] | 68.9  [49.1 – 90.7] | 537.2  [304.4 to 932.7] |
| Household air pollution from solid fuels | 84.2  [55.5 – 117.0] | 66.1  [35.9 – 106.5] | -21.6  [-51.2 to 17.5] | 58.5  [38.1 – 83.8] | 42.1  [21.4 – 69.1] | -28.1  [-56.8 to 12.7] | 25.7  [17.3 – 35.8] | 24.0  [13.0 – 38.5] | -6.8  [-41.9 to 40.3] |
| Occupational exposure to arsenic | 2.4  [0.7 – 4.0] | 6.0  [2.0 – 10.3] | 151.0  [108.9 to 208.8] | 1.7  [0.5 – 2.9] | 4.1  [1.3 – 7.2] | 137.3  [82.7 to 204.7] | 0.7  [0.2 – 1.1] | 1.9  [0.6 – 3.2] | 187.5  [134.7 to 272.7] |
| Occupational exposure to asbestos | 21.0  [15.0 – 28.3] | 70.0  [49.5 – 92.6] | 232.7  [174.2 to 289.7] | 16.5  [10.7 – 23.8] | 57.4  [37.3 – 79.9] | 247.8  [178.5 to 313.1] | 4.5  [2.9 – 7.4] | 12.6  [7.9 – 17.2] | 177.6  [62.5 to 312.4] |
| Occupational exposure to benzene | 0.7  [0.2 – 1.2] | 1.1  [0.3 – 1.8] | 54.9  [33.3 to 84.4] | 0.4  [0.1 – 0.7] | 0.6  [0.2 – 1.1] | 56.3  [31.8 to 94.4] | 0.3  [0.1 – 0.5] | 0.4  [0.1 – 0.7] | 52.9  [20.2 to 93.1] |
| Occupational exposure to beryllium | 0.1  [0.1 – 0.1] | 0.2  [0.2 – 0.3] | 150.5  [105.1 to 199.8] | 0.1  [0.1 – 0.1] | 0.2  [0.1 – 0.2] | 132.3  [76.1 to 195.6] | 0  [0 – 0] | 0.1  [0.1 – 0.1] | 199.5  [136.9 to 281.0] |
| Occupational exposure to cadmium | 0.2  [0.2 – 0.3] | 0.6  [0.4 – 0.7] | 164.1  [115.3 to 218.4] | 0.2  [0.1 – 0.2] | 0.4  [0.3 – 0.5] | 148.6  [90.3 to 221.0] | 0.1  [0 – 0.1] | 0.2  [0.1 – 0.2] | 205.1  [143.5 to 289.7] |
| Occupational exposure to chromium | 0.4  [0.3 – 0.5] | 1.1  [1.0 – 1.4] | 180.5  [129.6 to 236.7] | 0.3  [0.2 – 0.4] | 0.8  [0.6 – 1.0] | 164.8  [101.5 to 239.3] | 0.1  [0.1 – 0.1] | 0.4  [0.3 – 0.4] | 223.6  [159.1 to 311.2] |
| Occupational exposure to diesel engine exhaust | 5.0  [4.1 – 5.9] | 14.6  [12.3 – 17.3] | 193.2  [138.6 to 252.3] | 3.9  [3.1 – 4.9] | 11.0  [8.8 – 13.6] | 180.2  [119.0 to 252.5] | 1.1  [0.8 – 1.3] | 3.6  [2.8 – 4.5] | 241.6  [176.0 to 328.9] |
| Occupational exposure to formaldehyde | 0.6  [0.5 – 0.8] | 0.9  [0.7 – 1.1] | 35.5  [16.0 to 60.6] | 0.4  [0.3 – 0.5] | 0.6  [0.4 – 0.8] | 46.9  [21.1 to 81.6] | 0.2  [0.2 – 0.3] | 0.3  [0.2 – 0.3] | 14.5  [-10.1 to 49.1] |
| Occupational exposure to nickel | 2.6  [0.4 – 6.0] | 6.2  [1.0 – 14.1] | 137.4  [94.3 to 194.5] | 1.9  [0.3 – 4.6] | 4.4  [0.7 – 10.2] | 125.8  [72.3 to 196.1] | 0.7  [0.1 – 1.6] | 1.8  [0.3 – 4.2] | 171.2  [116.0 to 251.7] |
| Occupational exposure to polycyclic aromatic hydrocarbons | 1.4  [1.2 – 1.7] | 4.0  [3.2 – 4.8] | 179.3  [128.6 to 236.7] | 1.0  [0.8 – 1.3] | 2.8  [2.1 – 3.6] | 163.7  [103.4 to 240.1] | 0.4  [0.3 – 0.5] | 1.2  [0.9 – 1.6] | 222.7  [158.3 to 312.3] |
| Occupational exposure to silica | 14.0  [6.5 – 22.2] | 33.3  [14.8 – 53.6] | 137.7  [97.8 to 182.5] | 10.8  [5.0 – 17.2] | 25.2  [11.3 – 39.8] | 133.9  [84.6 to 190.8] | 3.2  [1.5 – 5.1] | 8.1  [3.4 – 13.0] | 150.3  [101.7 to 213.9] |
| Occupational exposure to sulfuric acid | 1.5  [0.6 – 2.8] | 2.7  [1.1 – 5.1] | 77.3  [52.2 to 107.1] | 1.4  [0.6 – 2.5] | 2.4  [1.0 – 4.6] | 79.0  [51.5 to 111.9] | 0.2  [0.1 – 0.3] | 0.3  [0.1 – 0.6] | 64.4  [40.3 to 97.7] |
| Occupational exposure to trichloroethylene | 0  [0 – 0] | 0  [0 – 0.1] | 359.2  [281.8 to 459.8] | 0  [0 – 0] | 0  [0 – 0.1] | 397.6  [286.1 to 545.5] | 0  [0 – 0] | 0  [0 – 0] | 280.4  [212.3 to 364.6] |
| Residential radon | 15.2  [2.9 – 29.7] | 41.6  [8.1 – 84.2] | 173.3  [125.5 to 225.1] | 11.0  [2.1 – 22.1] | 28.8  [5.5 – 58.6] | 161.1  [103.2 to 227.9] | 4.2  [0.8 – 8.4] | 12.8  [2.4 – 25.6] | 205.4  [153.7 to 267.0] |
| ***Metabolic risks*** | | | | | | | | | |
| High body-mass index | 53.9  [16.8 – 116.6] | 196.7  [96.0 – 336.4] | 265.3  [170.9 to 497.2] | 29.0  [7.6 – 66.2] | 107.0  [45.4 – 199.2] | 268.5  [163.0 to 559.5] | 24.8  [8.5 – 54.0] | 89.8  [44.4 – 151.1] | 261.7  [169.1 to 470.6] |
| High fasting plasma glucose | 49.6  [12.6 – 105.7] | 193.7  [51.1 – 402.8] | 290.4  [249.7 to 341.5] | 28.0  [6.3 – 62.6] | 108.4  [25.5 – 238.9] | 286.8  [227.2 to 362.4] | 21.6  [5.6 – 46.2] | 85.3  [22.4 – 182.7] | 295.0  [248.1 to 358.2] |

The figures inside square bracket represent the 95% uncertainty interval. Data Source: Global Burden of Disease, Injuries and Risk Factors 2019 Study

**Supplementary Table S15 Age-standardised Rates of Cancer DALYs due to 34 risk factors**

|  | **Both Sexes [95% UI]** | | | **Males [95% UI]** | | | **Females [95% UI]** | | |
| --- | --- | --- | --- | --- | --- | --- | --- | --- | --- |
| Risk Factors | **1990** | **2019** | **Percent change, 1990-2019** | **1990** | **2019** | **Percent change, 1990-2019** | **1990** | **2019** | **Percent change, 1990-2019** |
| *Behavioral Risks* | | | | | | | | | |
| Alcohol use | 165.4  [140.3 – 195.3] | 145.5  [123.3 – 169.1] | -12.0  [-27.0 to 5.9] | 282.2  [233.0 – 336.7] | 261.6  [219.1 – 307.3] | -7.3  [-25.0 to 14.5] | 48.8  [40.6 – 57.5] | 33.9  [28.6 – 39.7] | -30.5  [-41.4 to -17.0] |
| Chewing tobacco | 29.4  [23.4 – 36.1] | 28.1  [21.7 – 35.2] | -4.5  [-22.3 to 16.0] | 36.6  [25.2 – 48.8] | 32.8  [22.3 – 44.5] | -10.4  [-33.2 to 17.4] | 22.0  [17.1 – 27.8] | 23.3  [17.5 – 29.9] | 5.7  [-15.9 to 29.1] |
| Diet high in processed meat | 3.9  [1.4 – 5.8] | 4.6  [1.4 – 7.2] | 18.7  [-9.6 to 41.7] | 4.2  [1.5 – 6.4] | 5.5  [1.7 – 8.8] | 31.0  [-4.7 to 61.0] | 3.6  [1.3 – 5.4] | 3.8  [1.2 – 5.9] | 5.4  [-17.2 to 24.3] |
| Diet high in red meat | 10.9  [4.5 – 20.2] | 17.4  [8.4 – 28.7] | 59.0  [30.1 to 131.2] | 7.7  [1.5 – 17.7] | 15.8  [3.9 – 30.5] | 104.7  [48.5 to 294.1] | 14.3  [6.2 – 23.6] | 19.1  [10.1 – 29.2] | 33.6  [9.1 to 81.7] |
| Diet high in sodium | 51.8  [1.3 – 196.7] | 26.9  [0.7 – 103.3] | -48.1  [-56.5 to -40.0] | 67.7  [1.6 – 253.5] | 38.2  [0.9 – 143.7] | -43.6  [-54.5 to -31.4] | 36.5  [0.9 – 139.6] | 16.4  [0.4 – 65.2] | -55.0  [-64.8 to -46.1] |
| Diet low in calcium | 44.6  [35.3 – 56.6] | 45.6  [33.4 – 60.4] | 2.3  [-12.9 to 16.8] | 50.2  [39.4 – 64.4] | 57.6  [42.0 – 76.7] | 14.9  [-8.2 to 38.0] | 39.5  [30.4 – 50.6] | 34.5  [24.9 – 45.8] | -12.7  [-25.9 to 2.0] |
| Diet low in fiber | 7.0  [2.9 – 12.2] | 5.7  [2.4 – 10.5] | -18.9  [-30.9 to -7.6] | 7.4  [3.2 – 12.9] | 6.7  [2.8 – 12.1] | -10.3  [-29.3 to 6.6] | 6.6  [2.8 – 11.6] | 4.7  [2.0 – 8.7] | -28.2  [-39.3 to -16.7] |
| Diet low in fruits | 79.7  [43.0 – 121.2] | 40.9  [21.5 – 64.9] | -48.6  [-63.7 to -31.4] | 110.5  [60.1 – 167.4] | 60.0  [30.1 – 97.3] | -45.8  [-62.5 to -25.0] | 49.5  [26.6 – 76.0] | 23.2  [12.8 – 34.7] | -53.1  [-66.5 to -35.7] |
| Diet low in milk | 42.7  [29.2 – 55.9] | 51.4  [34.3 – 67.4] | 20.2  [6.2 to 33.7] | 46.7  [31.7 – 61.3] | 63.3  [42.4 – 84.6] | 35.7  [12.6 to 57.3] | 39.2  [26.8 – 51.9] | 40.3  [26.4 – 53.2] | 2.8  [-10.6 to 17.7] |
| Diet low in vegetables | 19.0  [1.6 – 39.9] | 4.0  [0.7 – 7.7] | -79.0  [-83.1 to -55.6] | 25.8  [2.1 – 54.7] | 5.3  [1.0 – 10.4] | -79.7  [-84.0 to -52.7] | 12.3  [1.2 – 25.7] | 2.8  [0.4 – 5.6] | -77.2  [-82.4 to -58.0] |
| Diet low in whole grains | 34.4  [13.2 – 45.7] | 41.7  [15.9 – 55.7] | 21.4  [7.3 to 35.4] | 38.1  [14.5 – 51.4] | 52.3  [20.1 – 70.8] | 37.2  [14.0 to 59.0] | 31.0  [11.8 – 41.2] | 31.9  [12.2 – 43.0] | 3.1  [-10.7 to 18.2] |
| Drug use | 38.1  [28.3 – 49.6] | 21.6  [17.1 – 27.3] | -43.2  [-52.7 to -30.3] | 45.6  [35.9 – 56.6] | 26.1  [20.5 – 32.2] | -42.7  [-54.1 to -27.9] | 30.8  [19.4 – 44.9] | 17.5  [12.5 – 23.5] | -43.1  [-58.1 to -22.0] |
| Low physical activity | 10.3  [3.9 – 19.3] | 11.8  [4.4 – 21.8] | 14.4  [1.2 to 28.8] | 8.6  [1.9 – 17.8] | 11.0  [2.7 – 22.2] | 27.3  [8.1 to 49.9] | 12.0  [5.4 – 21.1] | 12.6  [5.8 – 21.8] | 5.2  [-8.0 to 21.4] |
| Secondhand smoke | 44.0  [28.1 – 62.9] | 44.9  [28.9 – 64.8] | 2.2  [-13.6 to 18.8] | 40.8  [24.0 – 62.5] | 42.4  [24.1 – 65.2] | 3.9  [-20.8 to 37.1] | 48.0  [30.4 – 68.0] | 48.2  [30.8 – 70.0] | 0.5  [-14.9 to 19.4] |
| Smoking | 827.6  [735.9 – 922.6] | 672.4  [586.5 – 766.5] | -18.8  [-31.5 to -3.3] | 1523.8  [1335.5 – 1707.8] | 1257.4  [1086.4 – 1449.7] | -17.5  [-31.5 to 0.2] | 162.1  [138.4 – 187.9] | 130.8  [111.9 – 151.9] | -19.3  [-31.8 to -4.0] |
| Unsafe sex |  |  |  |  |  |  | 248.8  [209.1 – 331.2] | 183.3  [147.7 – 212.5] | -26.3  [-44.6 to -7.4] |
| *Environmental/occupational risk* | | | | | | | | | |
| Ambient particulate matter pollution | 54.5  [30.4 – 84.1] | 109.0  [79.3 – 141.4] | 100.2  [32.7 to 227.5] | 84.5  [46.7 – 131.7] | 161.5  [113.4 – 213.7] | 91.1  [23.3 to 221.4] | 25.7  [14.3 – 41.4] | 60.6  [43.2 – 80.2] | 135.7  [50.6 to 285.3] |
| Household air pollution from solid fuels | 104.2  [68.5 – 145.0] | 32.0  [17.5 – 51.6] | -69.3  [-80.7 to -54.1] | 147.2  [95.9 – 211.3] | 42.4  [21.7 – 69.7] | -71.2  [-82.6 to -55.1] | 62.7  [42.3 – 87.4] | 22.3  [12.1 – 35.5] | -64.5  [-77.6 to -47.2] |
| Occupational exposure to arsenic | 3.2  [0.9 – 5.3] | 3.3  [1.1 – 5.6] | 4.3  [-13.4 to 29.2] | 4.5  [1.3 – 7.6] | 4.6  [1.5 – 7.9] | 1.0  [-22.4 to 29.8] | 1.8  [0.5 – 3.0] | 2.0  [0.7 – 3.5] | 16.5  [-5.2 to 50.9] |
| Occupational exposure to asbestos | 23.4  [16.7 – 31.5] | 26.7  [19.0 – 35.3] | 14.2  [-6.0 to 35.6] | 39.8  [25.8 – 57.3] | 48.4  [32.0 – 67.5] | 21.6  [-2.6 to 44.8] | 9.8  [6.0 – 16.0] | 9.1  [5.7 – 12.3] | -7.6  [-46.0 to 39.0] |
| Occupational exposure to benzene | 1.1  [0.3 – 1.9] | 1.0  [0.3 – 1.7] | -11.6  [-24.2 to 5.6] | 1.3  [0.4 – 2.2] | 1.2  [0.3 – 2.0] | -9.8  [-23.9 to 12.2] | 0.9  [0.3 – 1.6] | 0.8  [0.2 – 1.4] | -14.0  [-32.9 to 8.5] |
| Occupational exposure to beryllium | 0.1  [0.1 – 0.2] | 0.1  [0.1 – 0.2] | 4.3  [-14.9 to 24.2] | 0.2  [0.1 – 0.2] | 0.2  [0.1 – 0.2] | -0.7  [-24.2 to 26.3] | 0.1  [0.1 – 0.1] | 0.1  [0.1 – 0.1] | 20.9  [-4.2 to 52.2] |
| Occupational exposure to cadmium | 0.3  [0.2 – 0.3] | 0.3  [0.2 – 0.4] | 10.1  [-10.3 to 32.0] | 0.4  [0.3 – 0.5] | 0.4  [0.3 – 0.5] | 6.4  [-18.6 to 36.6] | 0.2  [0.1 – 0.2] | 0.2  [0.1 – 0.3] | 23.6  [-1.3 to 57.0] |
| Occupational exposure to chromium | 0.5  [0.5 – 0.6] | 0.6  [0.5 – 0.8] | 17.1  [-4.0 to 40.4] | 0.8  [0.6 – 1.0] | 0.9  [0.7 – 1.1] | 13.4  [-13.3 to 45.1] | 0.3  [0.2 – 0.4] | 0.4  [0.3 – 0.5] | 31.5  [5.3 to 66.2] |
| Occupational exposure to diesel engine exhaust | 6.6  [5.5 – 7.9] | 8.1  [6.9 – 9.6] | 22.7  [0 to 46.8] | 10.3  [8.2 – 12.7] | 12.4  [9.9 – 15.3] | 20.4  [-5.3 to 50.9] | 2.8  [2.3 – 3.5] | 3.9  [3.1 – 4.9] | 38.4  [11.9 to 72.7] |
| Occupational exposure to formaldehyde | 1.0  [0.8 – 1.3] | 0.8  [0.6 – 1.0] | -25.9  [-36.9 to -13.3] | 1.3  [0.9 – 1.8] | 1.1  [0.8 – 1.4] | -19.0  [-32.3 to -2.1] | 0.8  [0.5 – 1.0] | 0.5  [0.4 – 0.6] | -37.3  [-50.5 to -18.1] |
| Occupational exposure to nickel | 3.4  [0.5 – 7.9] | 3.4  [0.6 – 7.8] | -1.2  [-19.5 to 22.9] | 5.1  [0.8 – 12.0] | 4.9  [0.8 – 11.3] | -3.7  [-26.4 to 27.6] | 1.8  [0.3 – 4.2] | 2.0  [0.4 – 4.5] | 9.7  [-12.3 to 42.4] |
| Occupational exposure to polycyclic aromatic hydrocarbons | 1.9  [1.6 – 2.3] | 2.2  [1.8 – 2.7] | 16.7  [-4.1 to 40.2] | 2.7  [2.1 – 3.5] | 3.1  [2.3 – 4.0] | 13.0  [-13.0 to 44.4] | 1.0  [0.8 – 1.3] | 1.3  [1.0 – 1.7] | 31.1  [5.1 to 67.0] |
| Occupational exposure to silica | 18.5  [8.6 – 29.1] | 18.3  [8.2 – 29.4] | -1.0  [-17.8 to 17.6] | 28.1  [12.9 – 44.9] | 28.1  [12.6 – 44.1] | -0.1  [-21.3 to 24.2] | 8.7  [3.9 – 13.5] | 8.8  [3.6 – 14.1] | 1.1  [-19.1 to 26.6] |
| Occupational exposure to sulfuric acid | 2.1  [0.9 – 3.9] | 1.7  [0.7 – 3.1] | -22.1  [-33.2 to -9.1] | 3.7  [1.5 – 6.7] | 3.0  [1.2 – 5.6] | -19.5  [-31.8 to -4.4] | 0.5  [0.2 – 0.9] | 0.4  [0.1 – 0.7] | -29.4  [-39.7 to -15.5] |
| Occupational exposure to trichloroethylene | 0  [0 – 0] | 0  [0 – 0] | 104.9  [70.0 to 150.5] | 0  [0 – 0] | 0  [0 – 0.1] | 127.9  [77.1 to 194.7] | 0  [0 – 0] | 0  [0 – 0] | 62.5  [33.2 to 98.6] |
| Residential radon | 18.8  [3.6 – 36.7] | 19.5  [3.8 – 39.3] | 4.1  [-14.4 to 24.0] | 27.7  [5.2 – 55.6] | 28.3  [5.5 – 57.5] | 2.0  [-20.2 to 27.6] | 10.2  [2.0 – 20.4] | 11.4  [2.2 – 22.9] | 12.6  [-6.6 to 35.9] |
| *Metabolic Risks* | | | | | | | | | |
| High body-mass index | 68.6  [21.6 – 148.7] | 102.9  [50.8 – 173.3] | 49.9  [9.9 to 150.3] | 76.4  [19.9 – 174.6] | 118.0  [51.2 – 217.3] | 54.5  [8.5 to 179.4] | 60.5  [20.9 – 130.5] | 88.2  [44.9 – 147.0] | 45.7  [6.6 to 134.6] |
| High fasting plasma glucose | 58.3  [14.8 – 124.8] | 86.6  [22.6 – 180.7] | 48.5  [32.7 to 67.9] | 68.3  [15.4 – 152.5] | 100.8  [23.5 – 223.2] | 47.4  [25.0 to 75.9] | 49.8  [12.9 – 106.8] | 74.7  [19.6 – 160.8] | 50.0  [32.0 to 73.6] |

UI = Uncertainty Interval; DALYs: Disability-adjusted Life Years. The age-standardized rates are presented per 100,000 and were calculated using GBD reference population standard.

Data Source: Global Burden of Disease, Injuries and Risk Factors 2019 Study.

# Supplementary Figures

**Supplementary Figure S1 Temporal Patterns of Cancer Incident Cases by Country, 1990-2019**

**
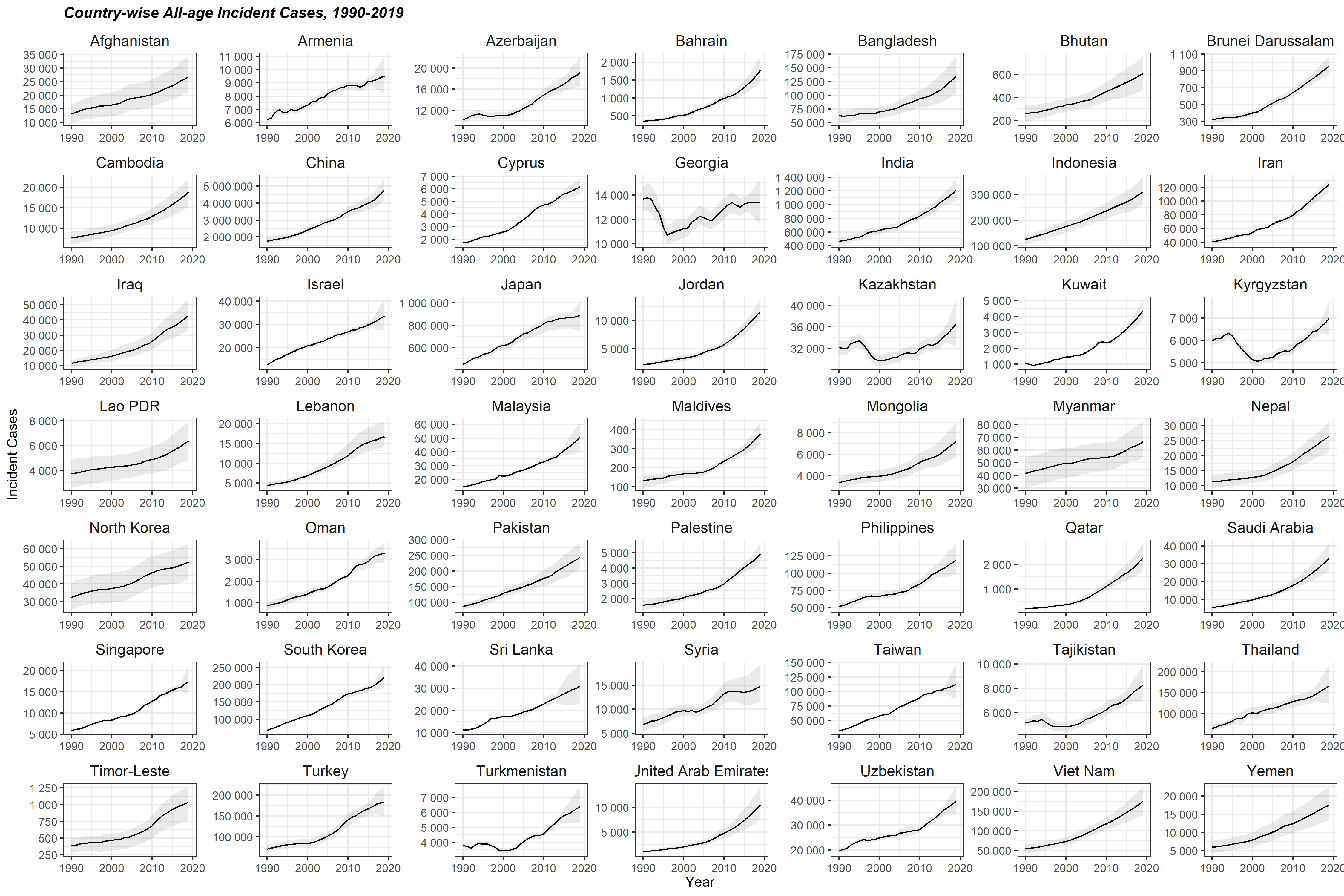
**

Grey Band represents 95% uncertainty interval. Data Source: Global Burden of Disease, Injuries and Risk Factors 2019 Study

**Supplementary Figure S2 Temporal Patterns of Cancer Deaths by Country, 1990-2019**

**
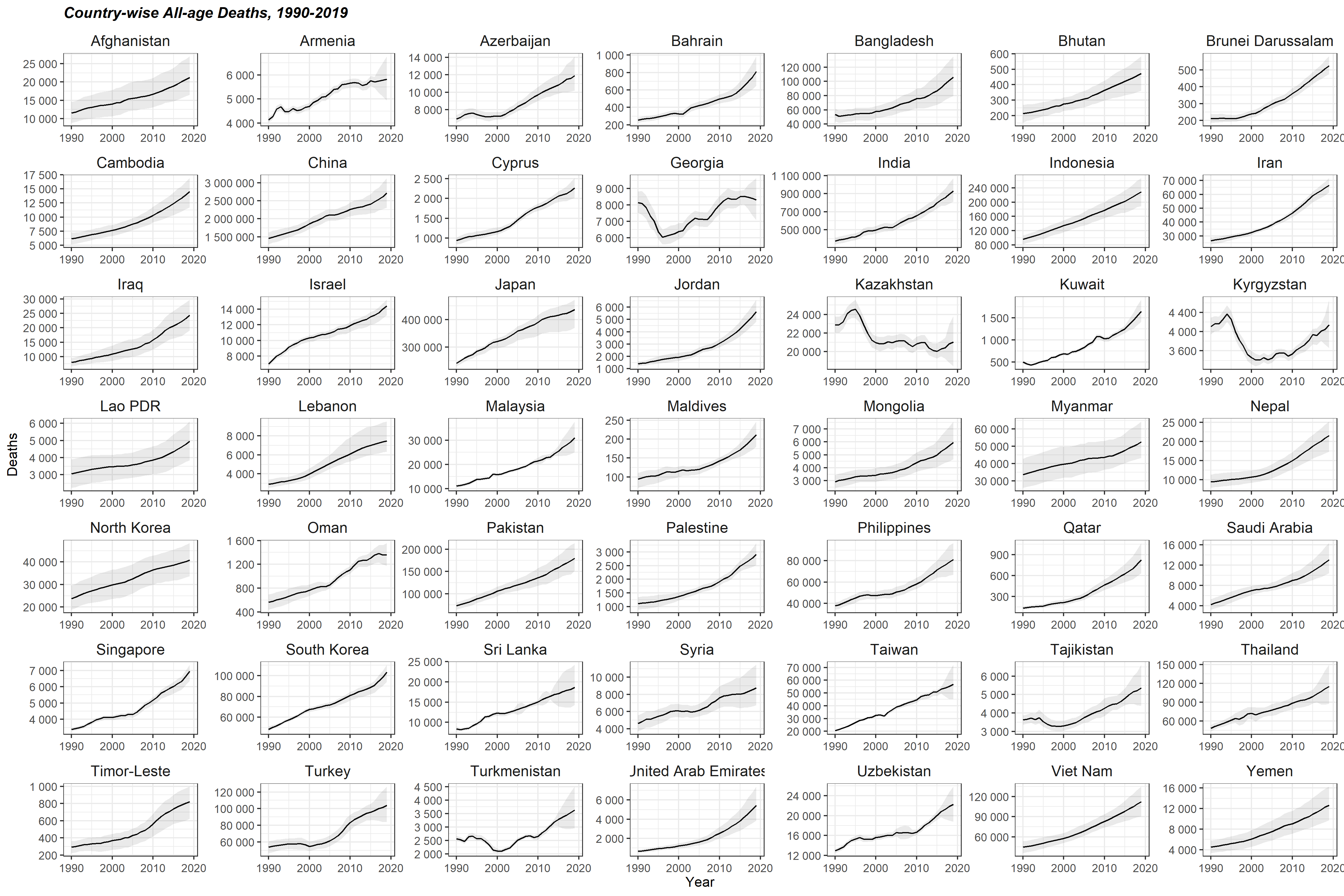
**

Grey Band represents 95% uncertainty interval. Data Source: Global Burden of Disease, Injuries and Risk Factors 2019 Study

**Supplementary Figure S3 Temporal Patterns of Cancer DALYs by Country, 1990-2019
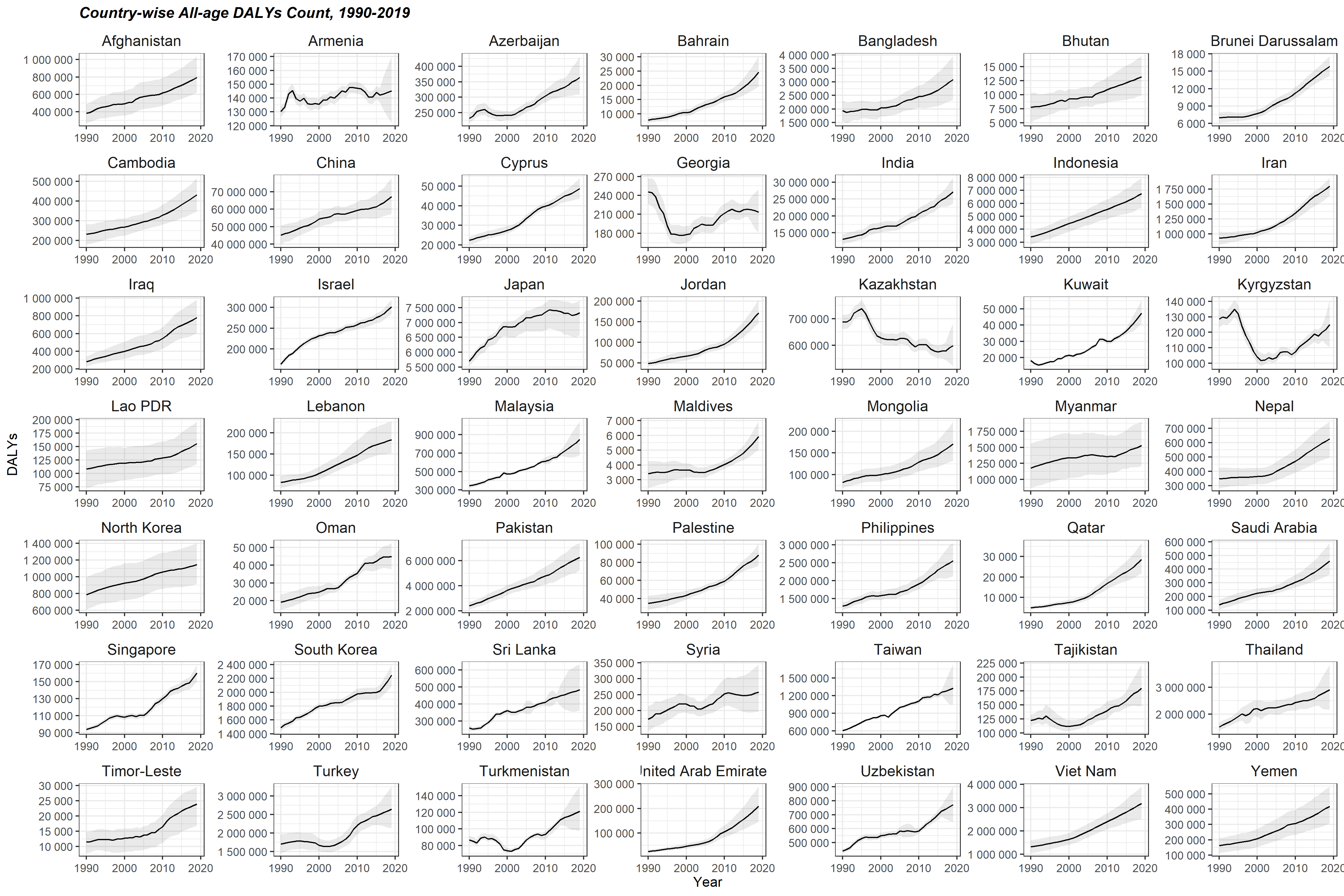
**

DALYs: Disability-adjusted Life Years. Grey Band represents 95% uncertainty interval. Data Source: Global Burden of Disease, Injuries and Risk Factors 2019 Study

**Supplementary Figure S4 Temporal Patterns of Cancer by Age-standardized Incidence Rate by Country, 1990-2019**

**
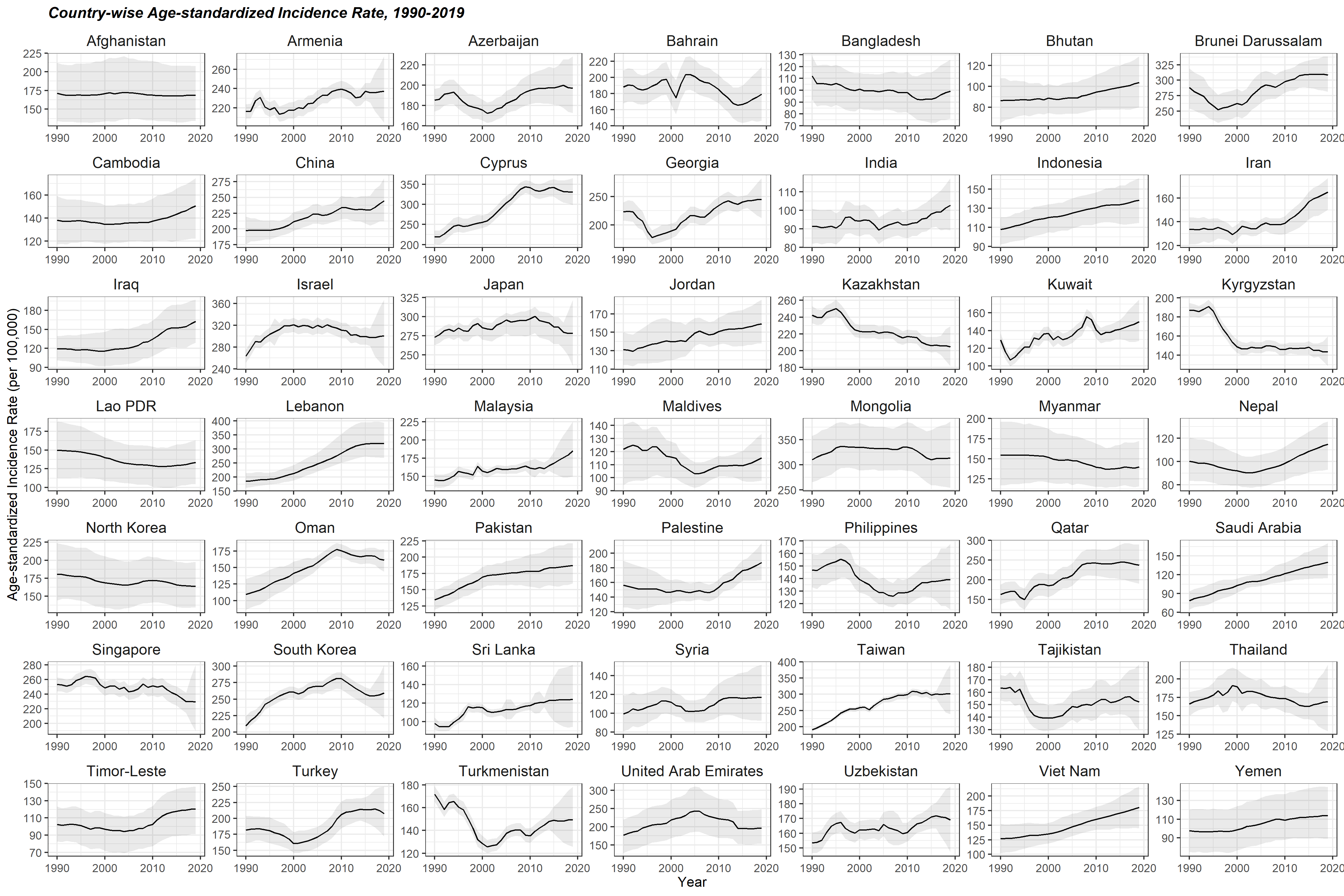
**

Grey Band represents 95% uncertainty interval. Data Source: Global Burden of Disease, Injuries and Risk Factors 2019 Study

**Supplementary Figure S5 Temporal Patterns of Cancer by Age-standardized Mortality Rate by Country, 1990-2019**

**
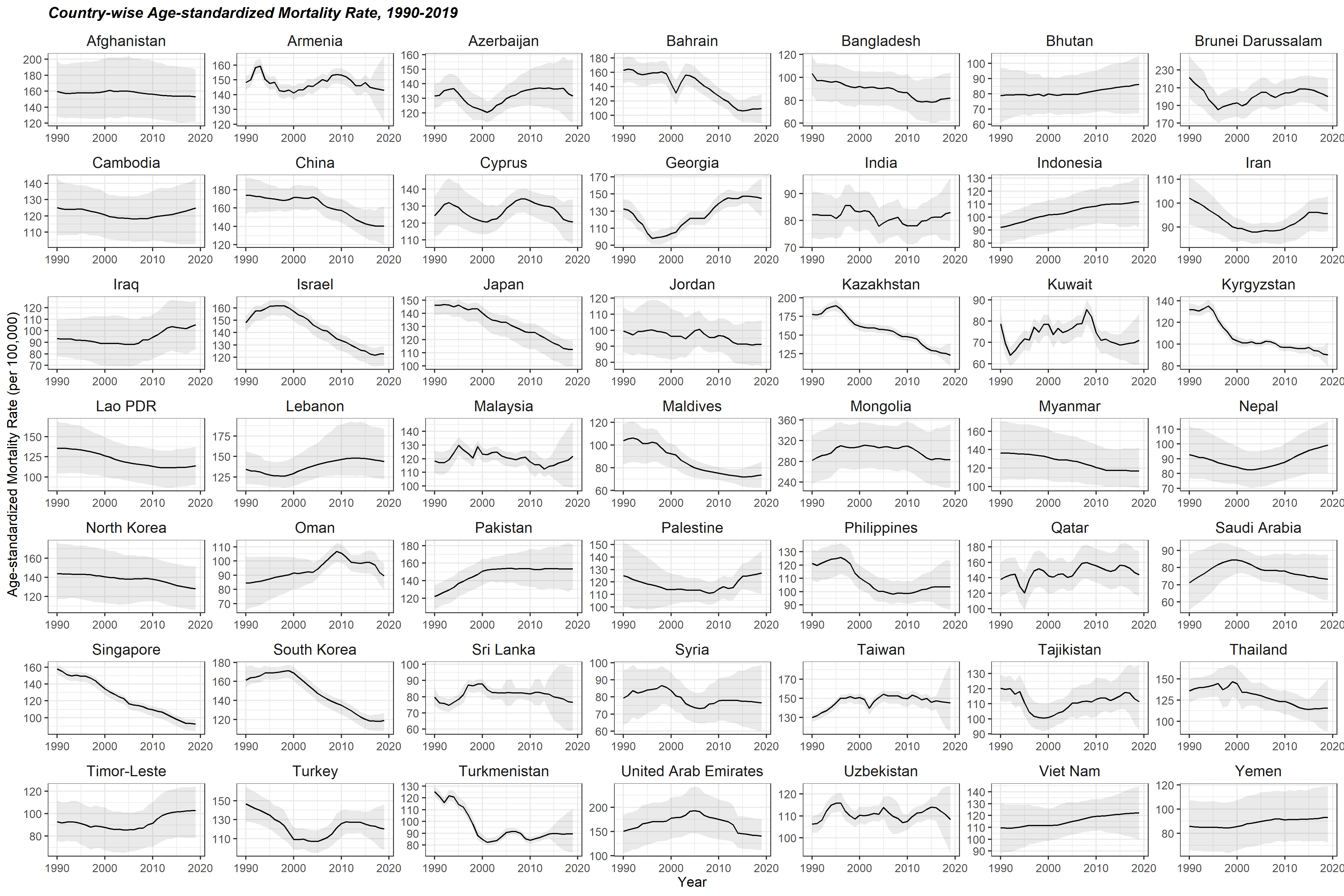
**

Grey Band represents 95% uncertainty interval. Data Source: Global Burden of Disease, Injuries and Risk Factors 2019 Study

**Supplementary Figure S6 Temporal Patterns of Cancer by Age-standardized DALYs Rate by Country, 1990-2019**

**
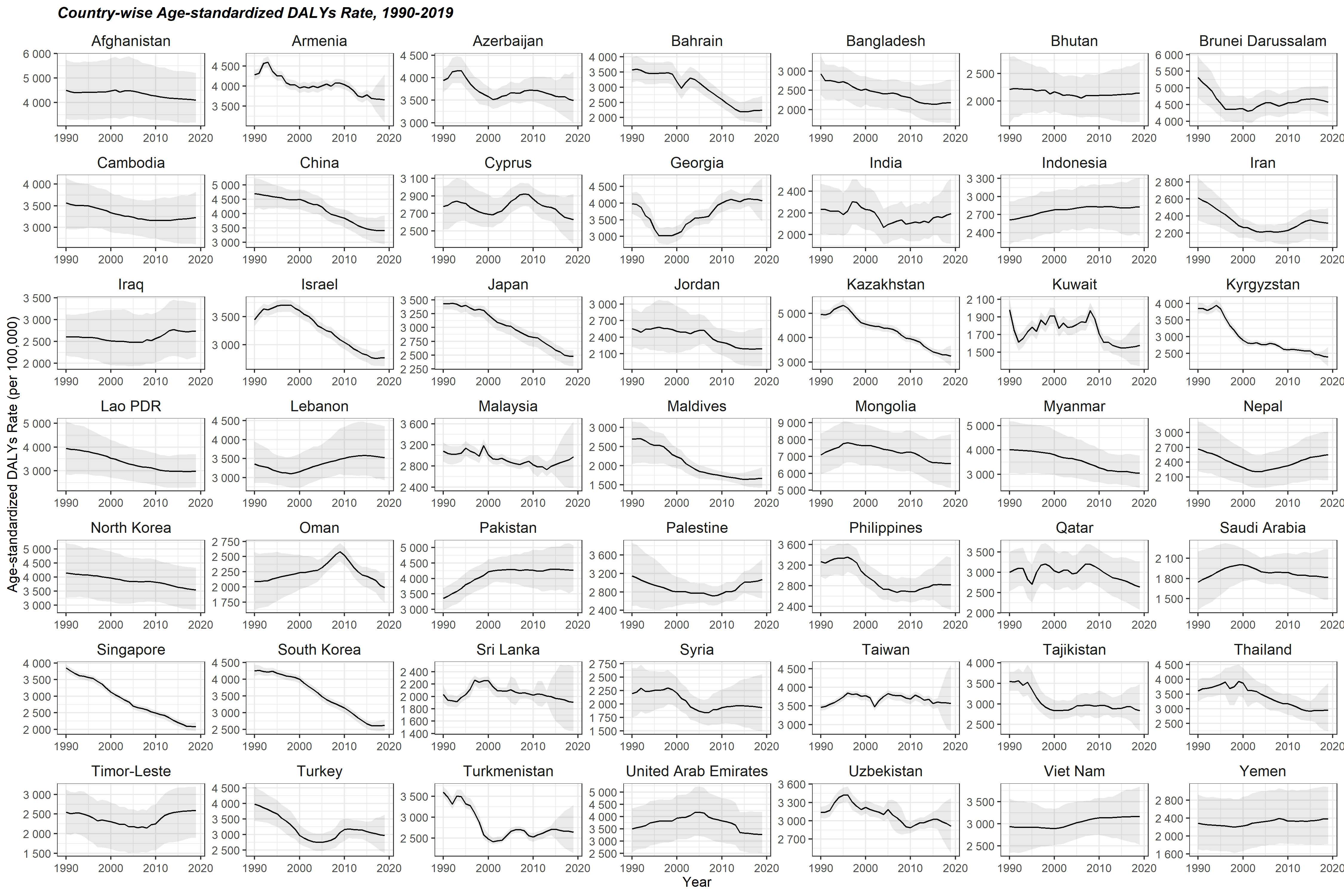
**

DALYs: Disability-adjusted Life Years. Grey Band represents 95% uncertainty interval. Data Source: Global Burden of Disease, Injuries and Risk Factors 2019 Study

**Supplementary Figure S7 Country-wise Rank of 29 Cancers in 49 Asian Countries as per Cancer Incident Cases in 2019, Both Sexes Combined**

**
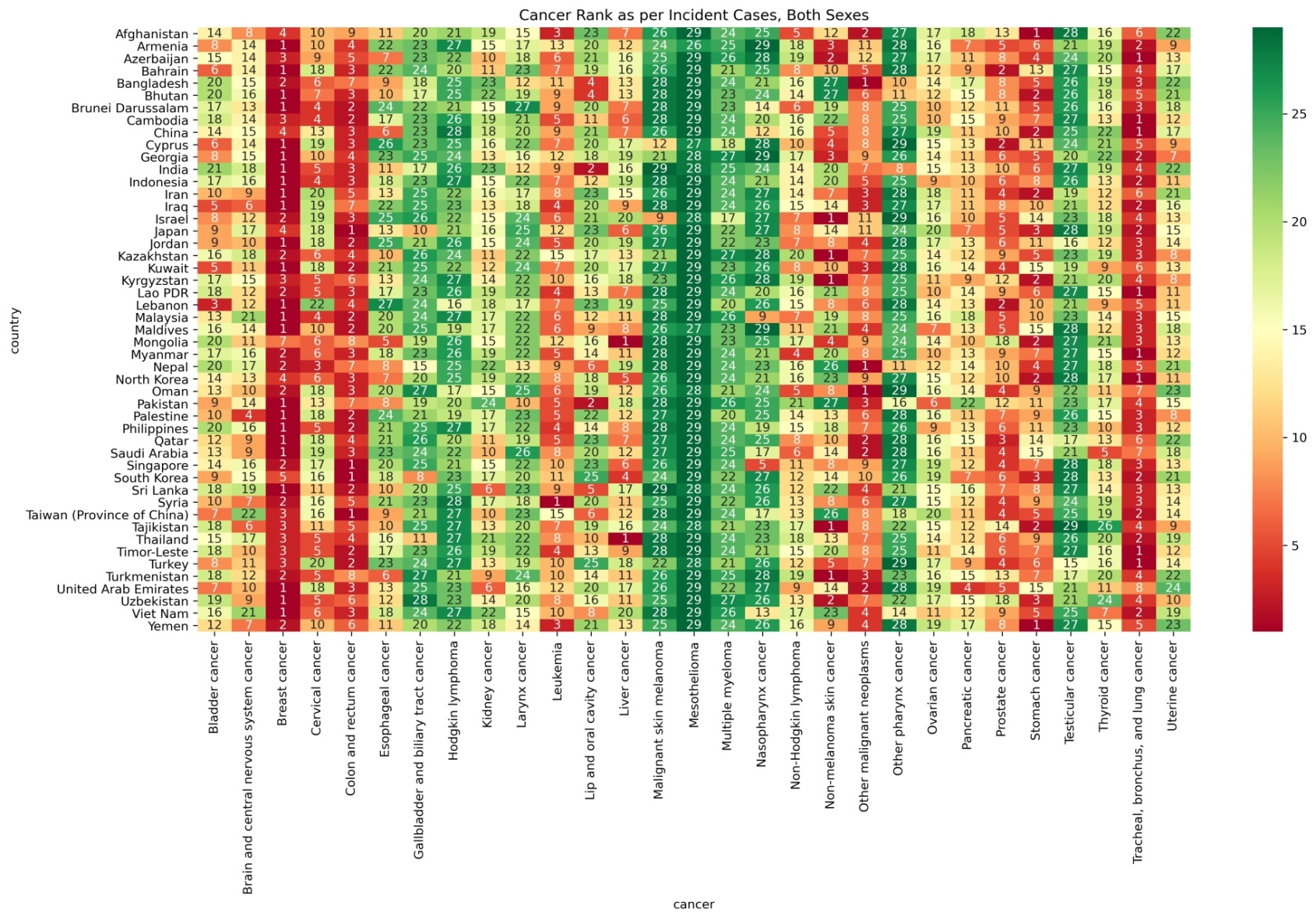
**

The numbers in the cells represent cancer rank in particular country. The cancer rank increases from green to red. Data Source: Global Burden of Disease, Injuries and Risk Factors 2019 Study

**Supplementary Figure S8 Country-wise Rank of 29 Cancers in 49 Asian Countries as per Cancer Deaths in 2019, Both Sexes Combined**

**
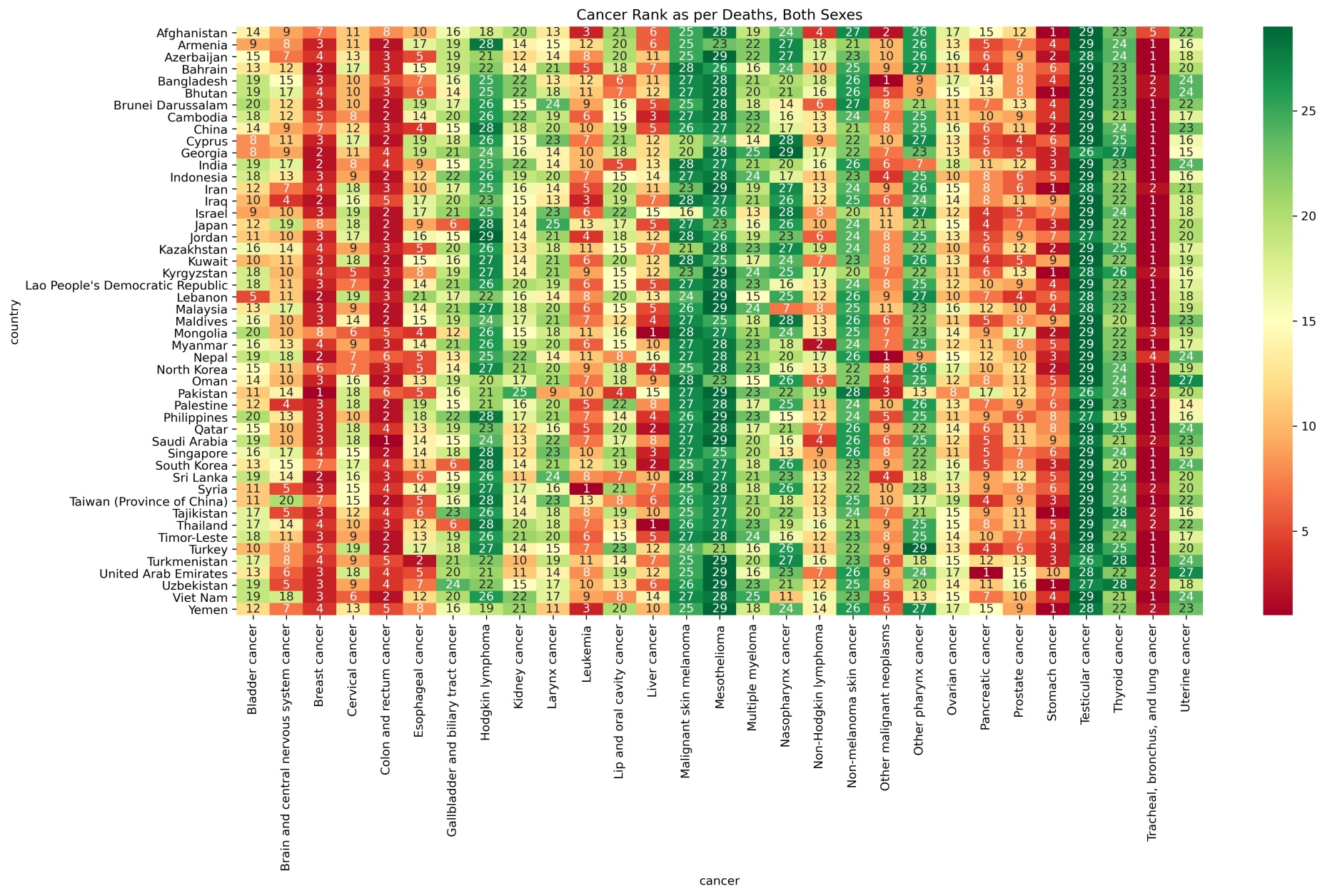
**

The numbers in the cells represent cancer rank in particular country. The cancer rank increases from green to red. Data Source: Global Burden of Disease, Injuries and Risk Factors 2019 Study

**Supplementary Figure S9 Country-wise Rank of 29 Cancers in 49 Asian Countries as per Cancer DALYs in 2019, Both Sexes Combined**

**
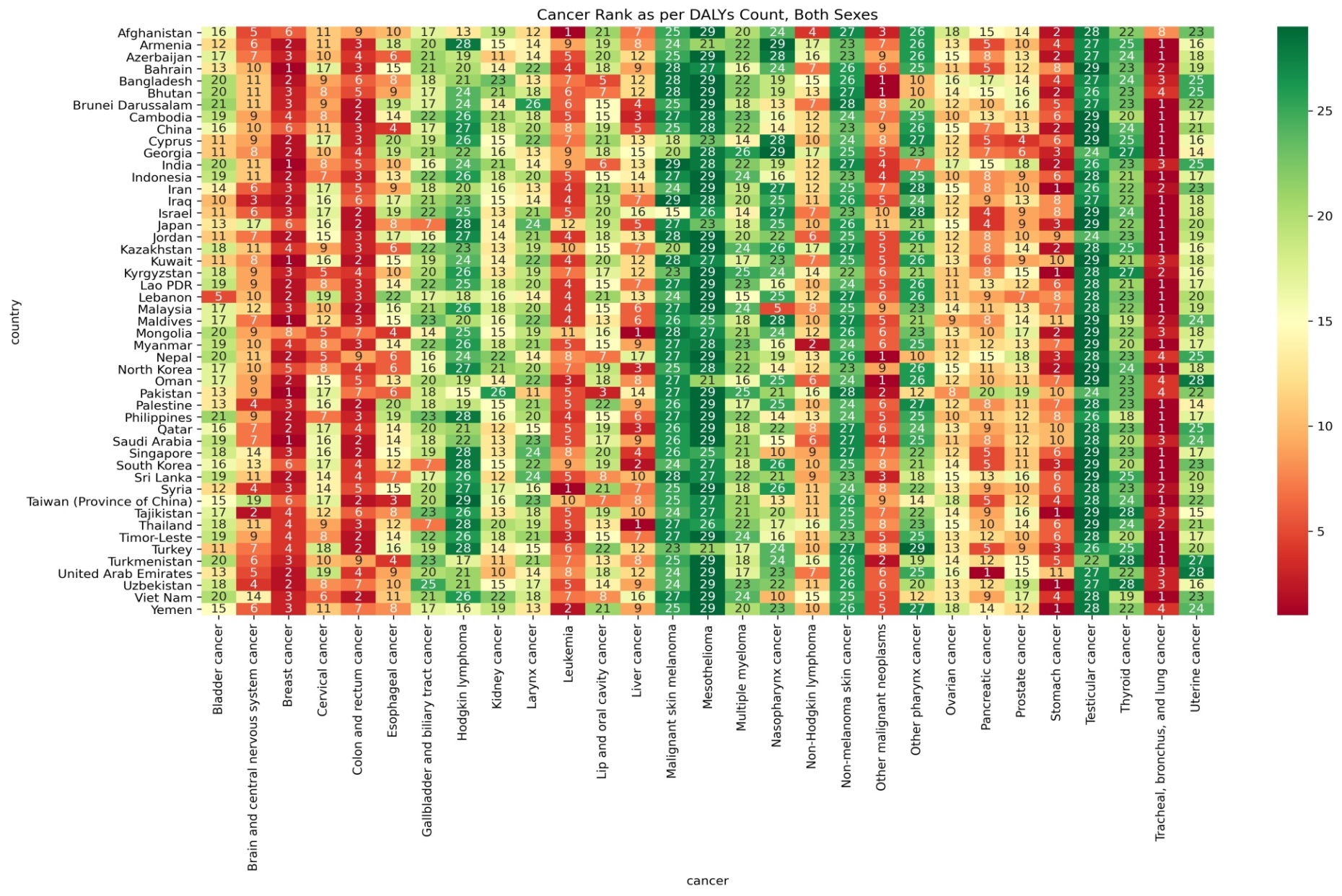
**

The numbers in the cells represent cancer rank in particular country. The cancer rank increases from green to red. Data Source: Global Burden of Disease, Injuries and Risk Factors 2019 Study

**Supplementary Figure S10 Country-wise Rank of 26 Cancers in 49 Asian Countries as per Cancer Incident Cases in 2019, Males**

**
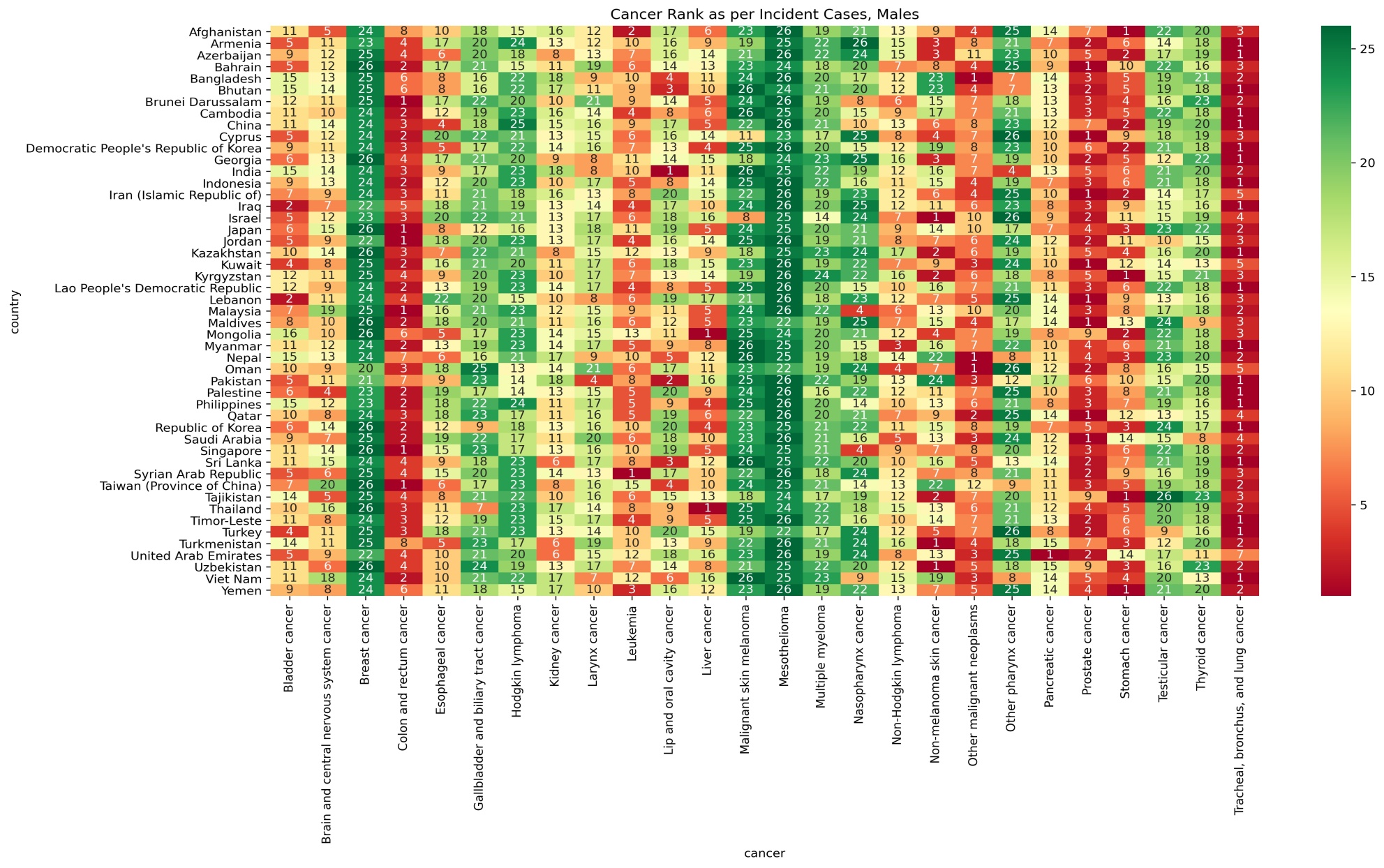
**

The numbers in the cells represent cancer rank in particular country. The cancer rank increases from green to red. Data Source: Global Burden of Disease, Injuries and Risk Factors 2019 Study

**Supplementary Figure S11 Country-wise Rank of 27 Cancers in 49 Asian Countries as per Cancer Incident Cases in 2019, Females**

**
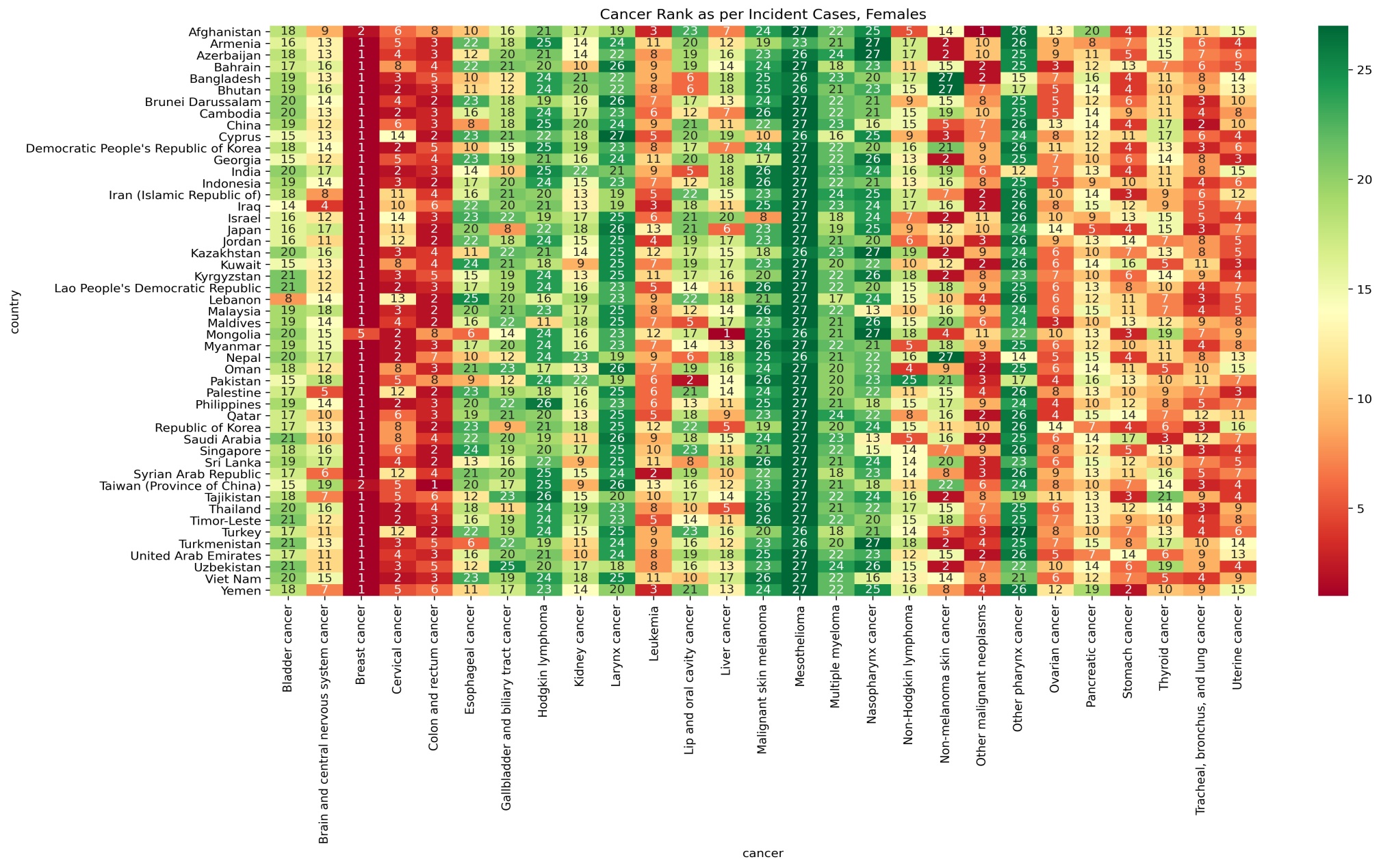
**

The numbers in the cells represent cancer rank in particular country. The cancer rank increases from green to red. Data Source: Global Burden of Disease, Injuries and Risk Factors 2019 Study

**Supplementary Figure S12 Agewise Burden of Cancer in Asia, Males. A) 1990 B) 2019**

**
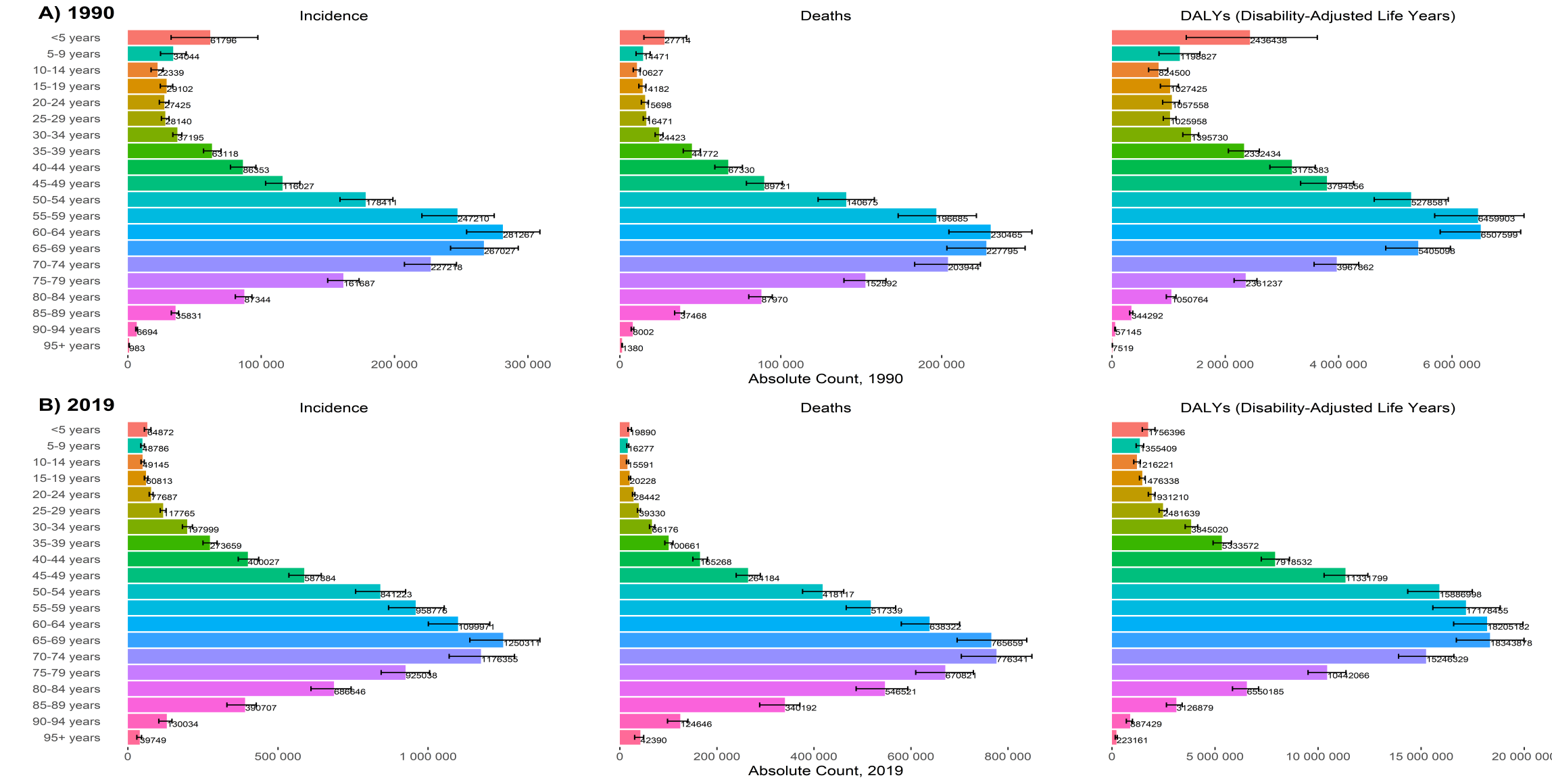
**

Incidence: Age-specific new cases; Deaths: Age-specific death counts; DALYs: Disability-adjusted-life-years. Data Source: Global Burden of Disease, Injuries and Risk Factors 2019 Study

**Supplementary Figure S13 Agewise Burden of Cancer in Asia, Females. A) 1990 B) 2019**

**
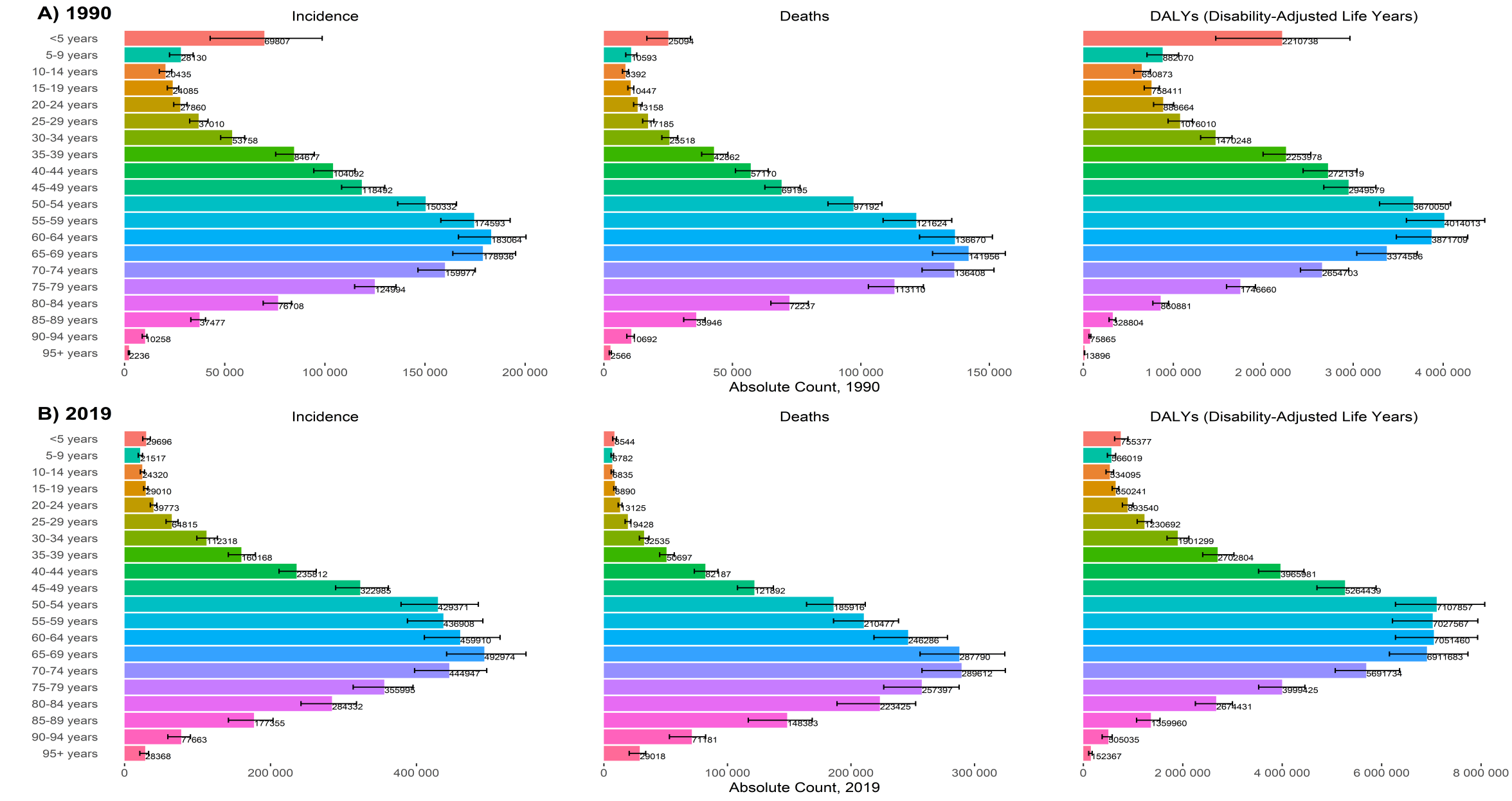
**

Incidence: Age-specific new cases; Deaths: Age-specific death counts; DALYs: Disability-adjusted-life-years. Data Source: Global Burden of Disease, Injuries and Risk Factors 2019 Study

**Supplementary Figure S14 Cancer Incidence in Different Age Groups in Asia, Both Sexes Combined**

**
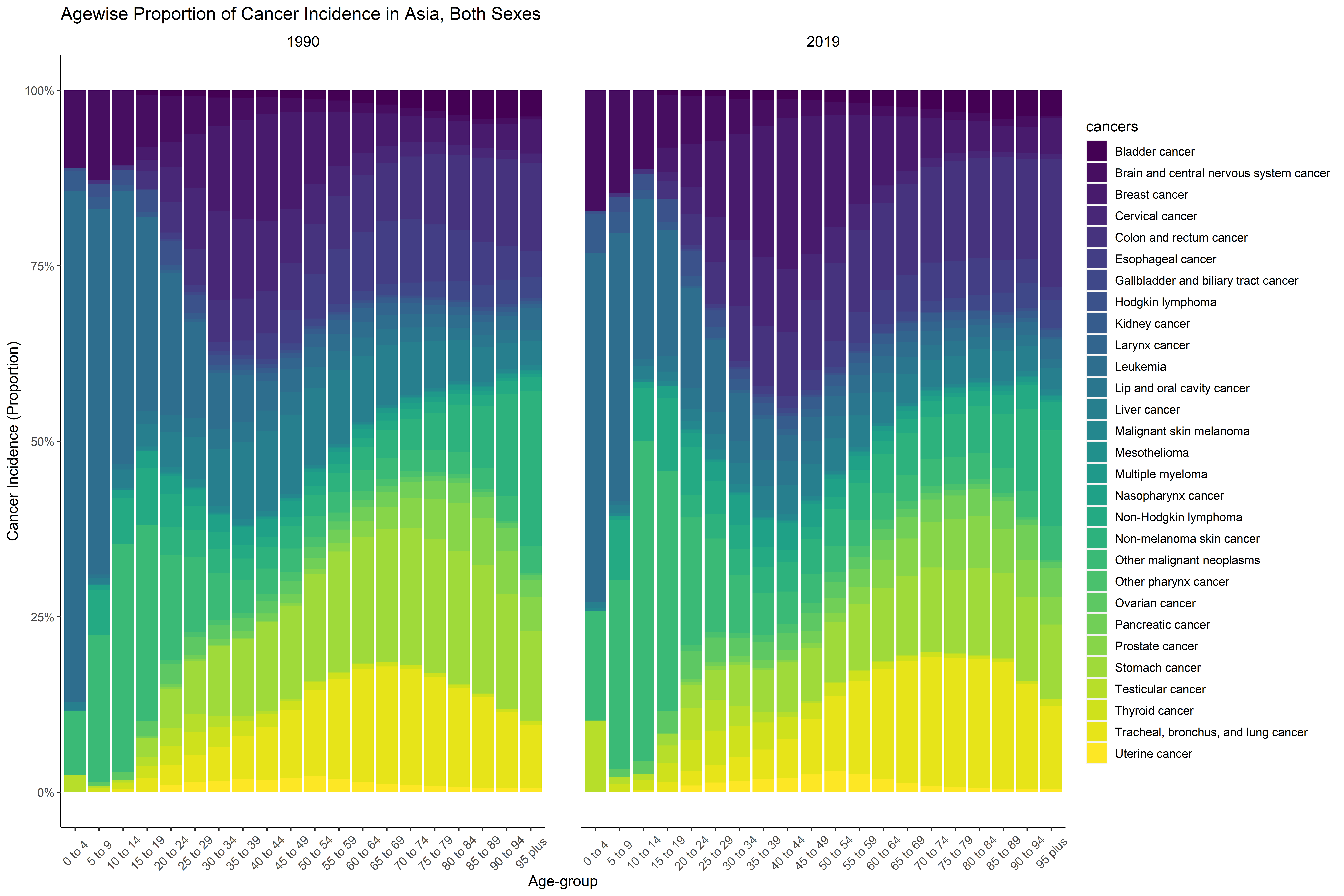
**

Data Source: Global Burden of Disease, Injuries and Risk Factors 2019 Study

**Supplementary Figure S15 Cancer Incidence in Different Age Groups in Asia, Males**

**
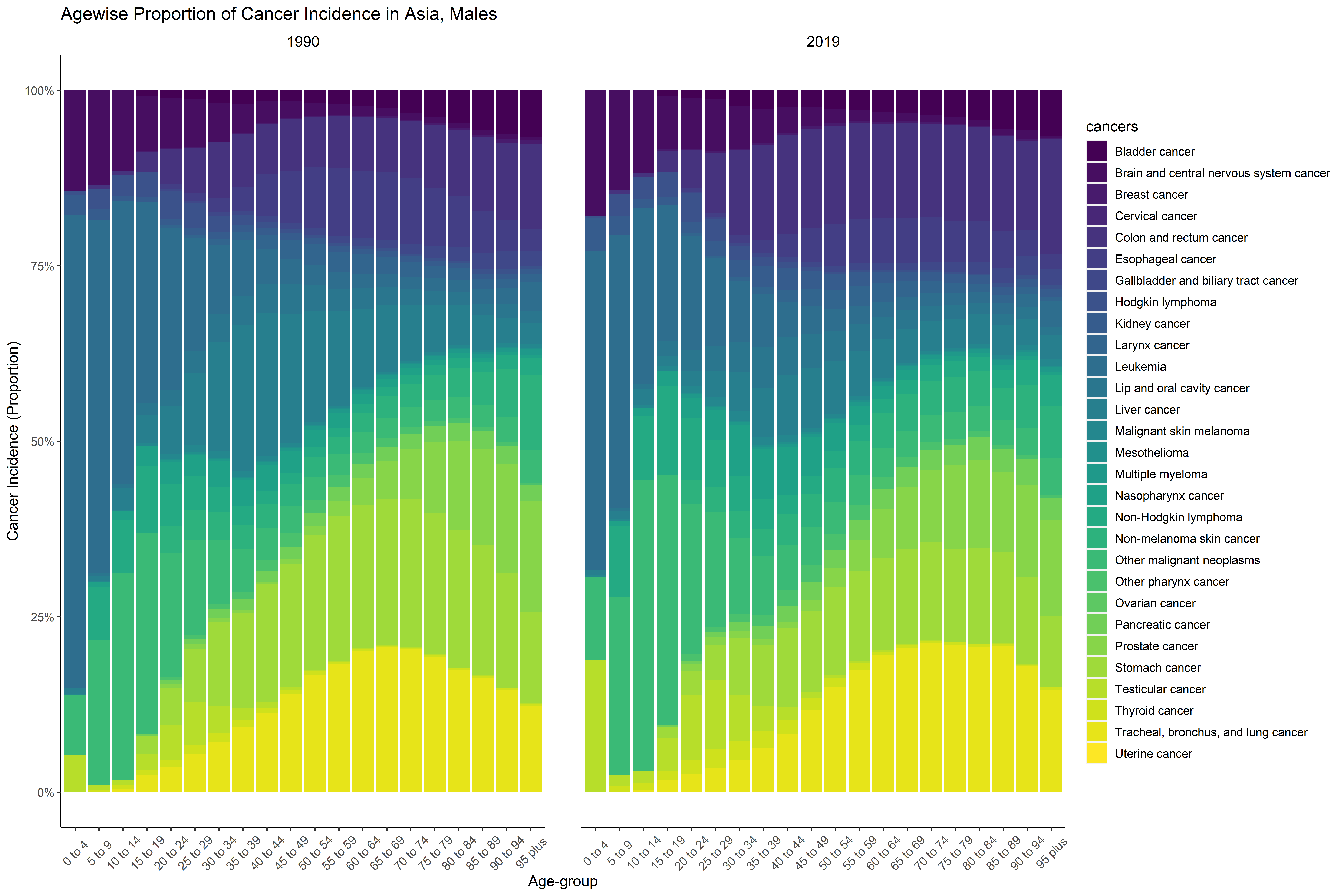
**

Data Source: Global Burden of Disease, Injuries and Risk Factors 2019 Study

**Supplementary Figure S16 Cancer Incidence in Different Age Groups in Asia, Females**

**
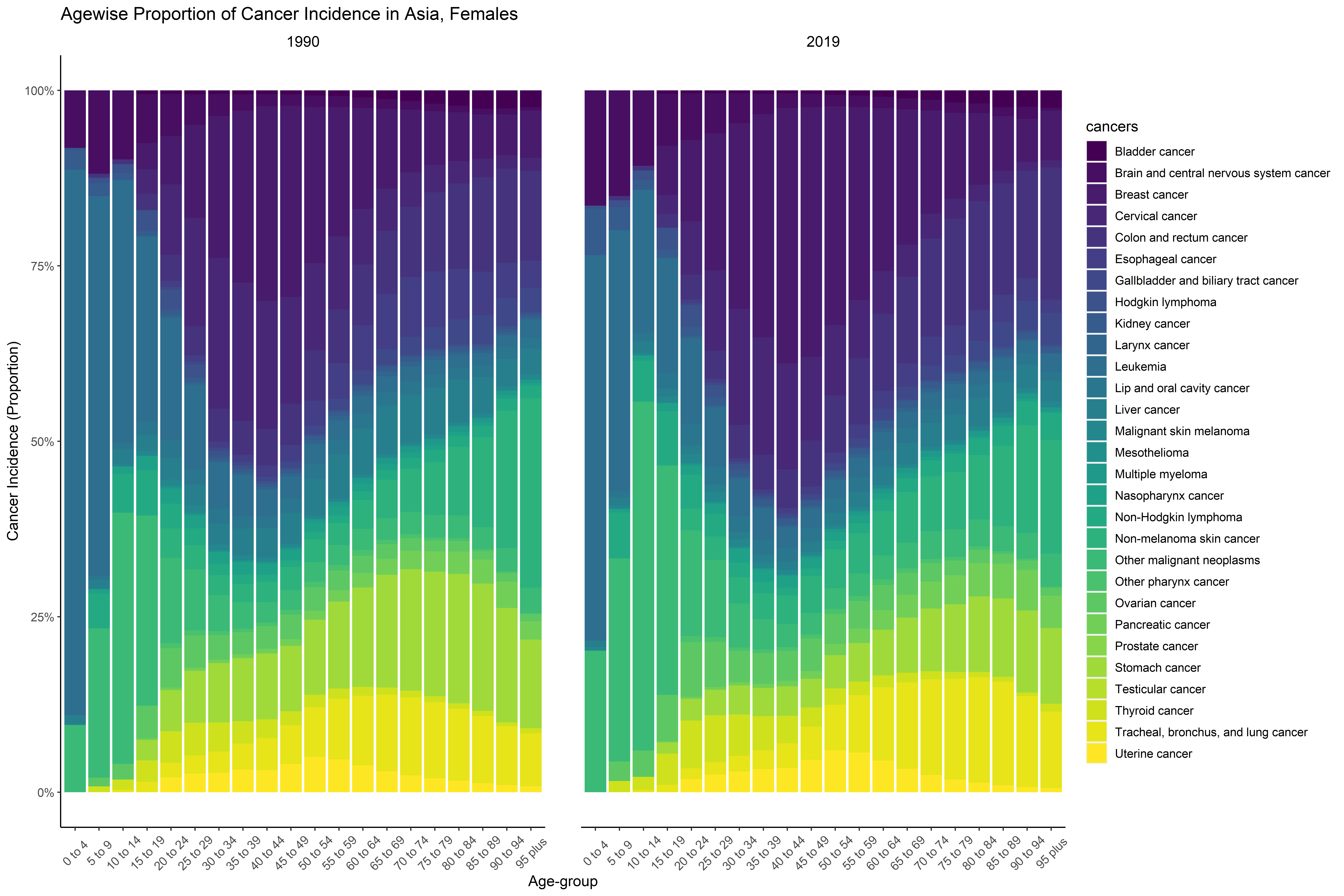
**

Data Source: Global Burden of Disease, Injuries and Risk Factors 2019 Study

**Supplementary Figure S17 Top-10 Risk Factors as per Age-standardized DALYs Rate in 1990 and 2019**


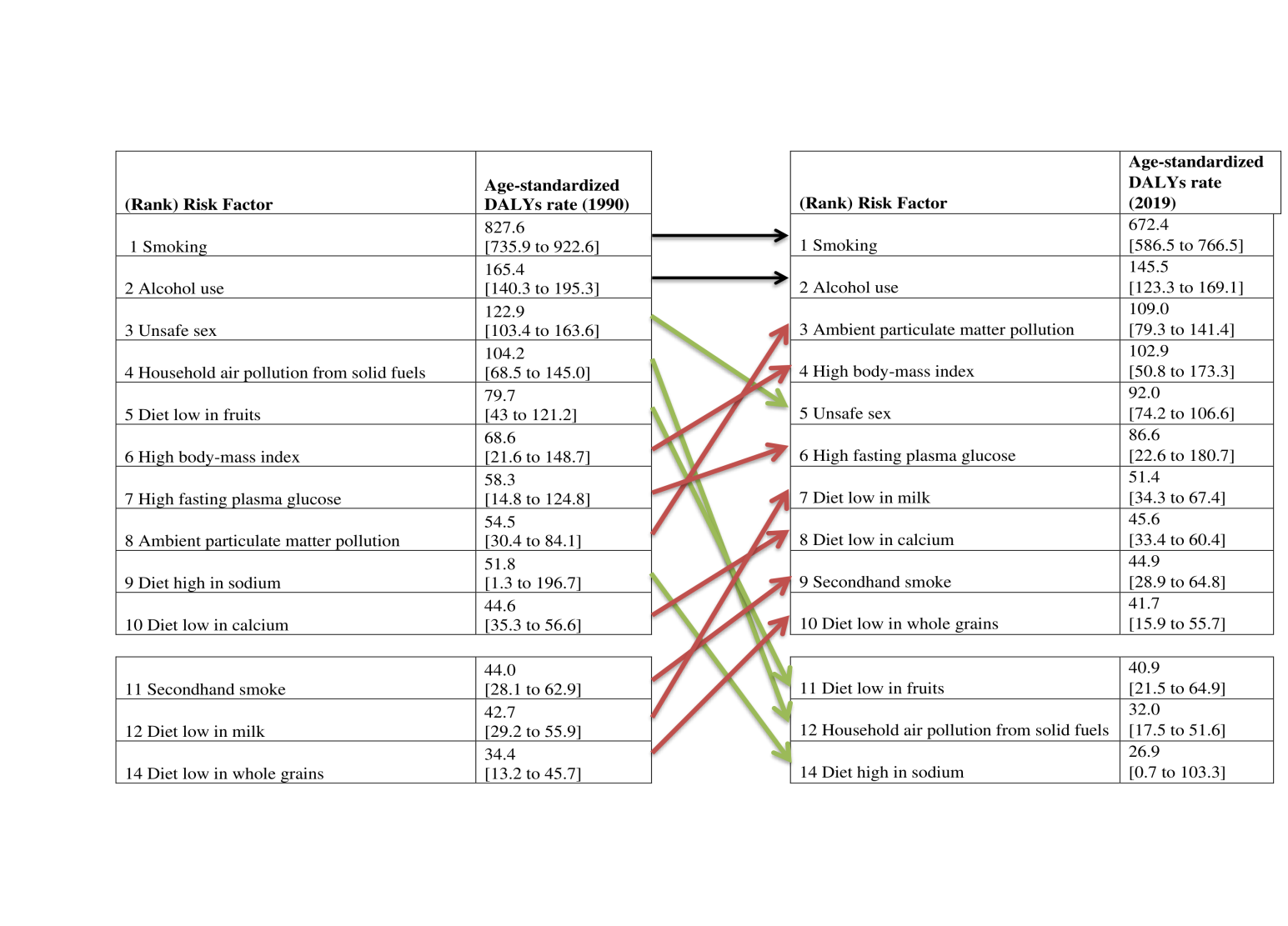


DALYs: Disability-adjusted Life Years. The numbers inside parenthesis represent 95% uncertainty interval. Data Source: Global Burden of Disease, Injuries and Risk Factors 2019 Study

**Supplementary Figure S18 Top-10 Risk Factors as per Age-standardized Mortality Rate in 1990 and 2019**


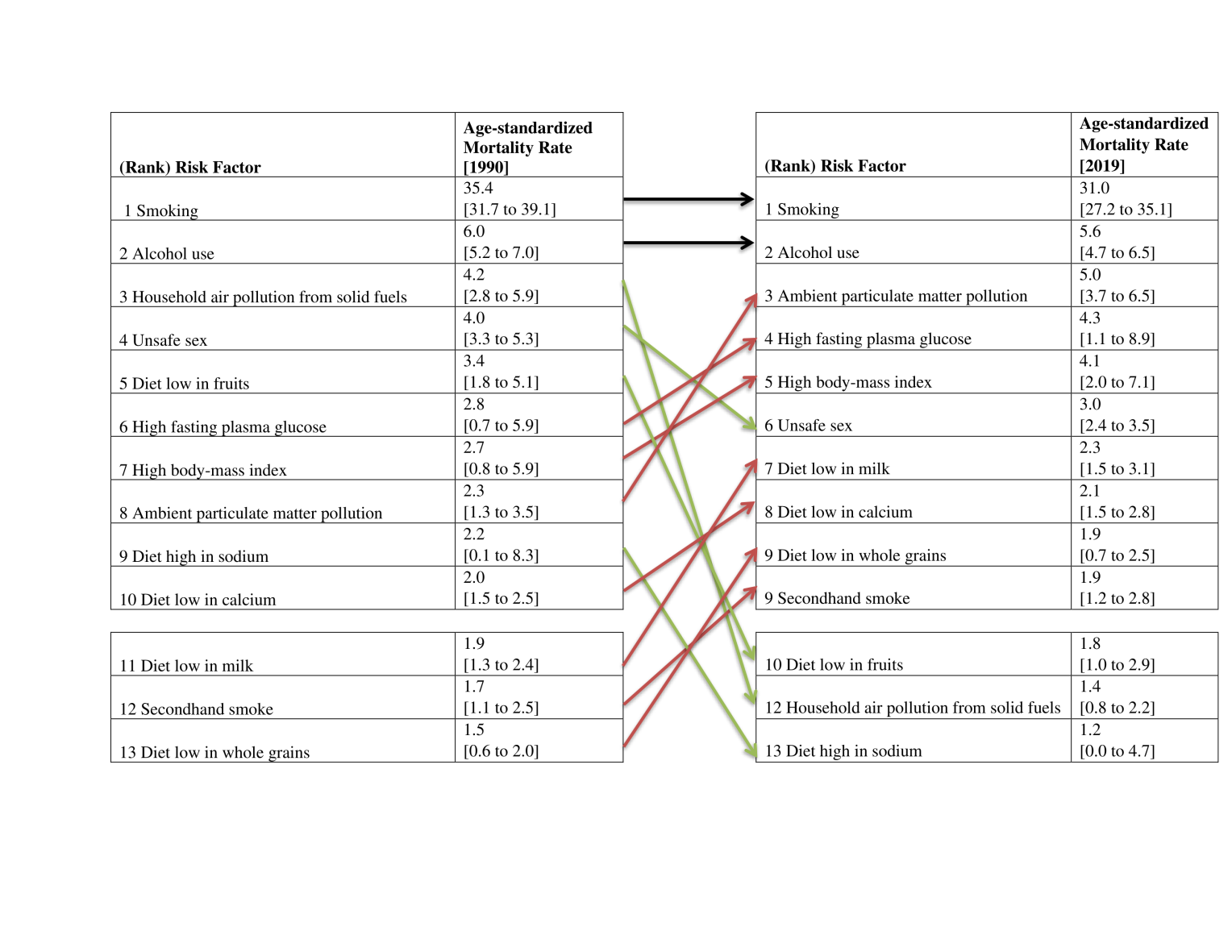


The numbers inside parenthesis represent 95% uncertainty interval. Data Source: Global Burden of Disease, Injuries and Risk Factors 2019 Study

**Supplementary Figure S19 Countrywise Rank of different Risk Factors as per Age-standardized DALYs Rate in 2019, Both Sexes**

**
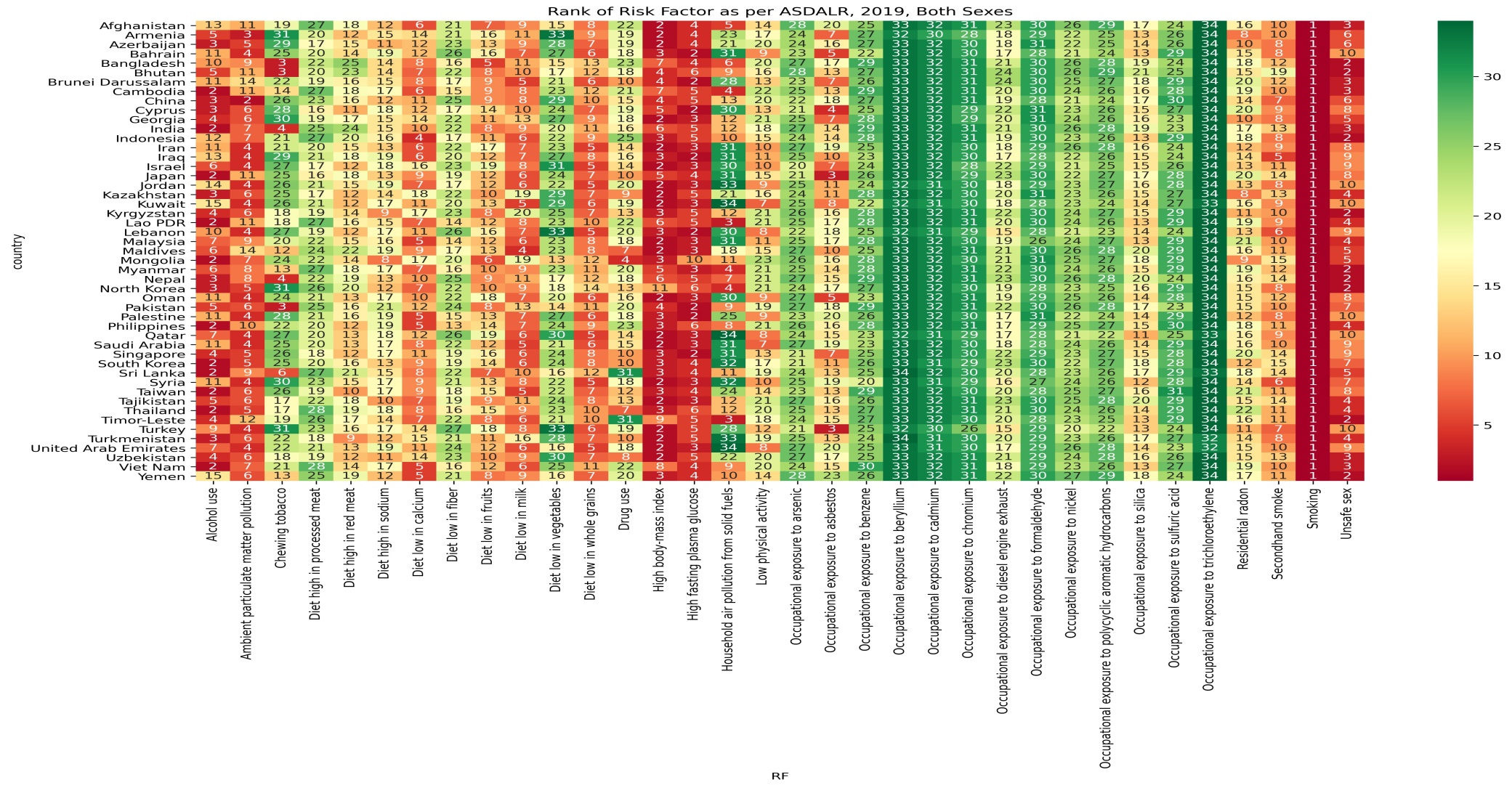
**

RF: Risk Factor; DALYs: Disability-adjusted life years. The numbers inside the cells represent the rank of risk factor in a country based on age-standardized DALYs rate in 2019.

**Supplementary Figure S20 Countrywise Rank of different Risk Factors as per Age-standardized DALYs Rate in 2019, Males**

**
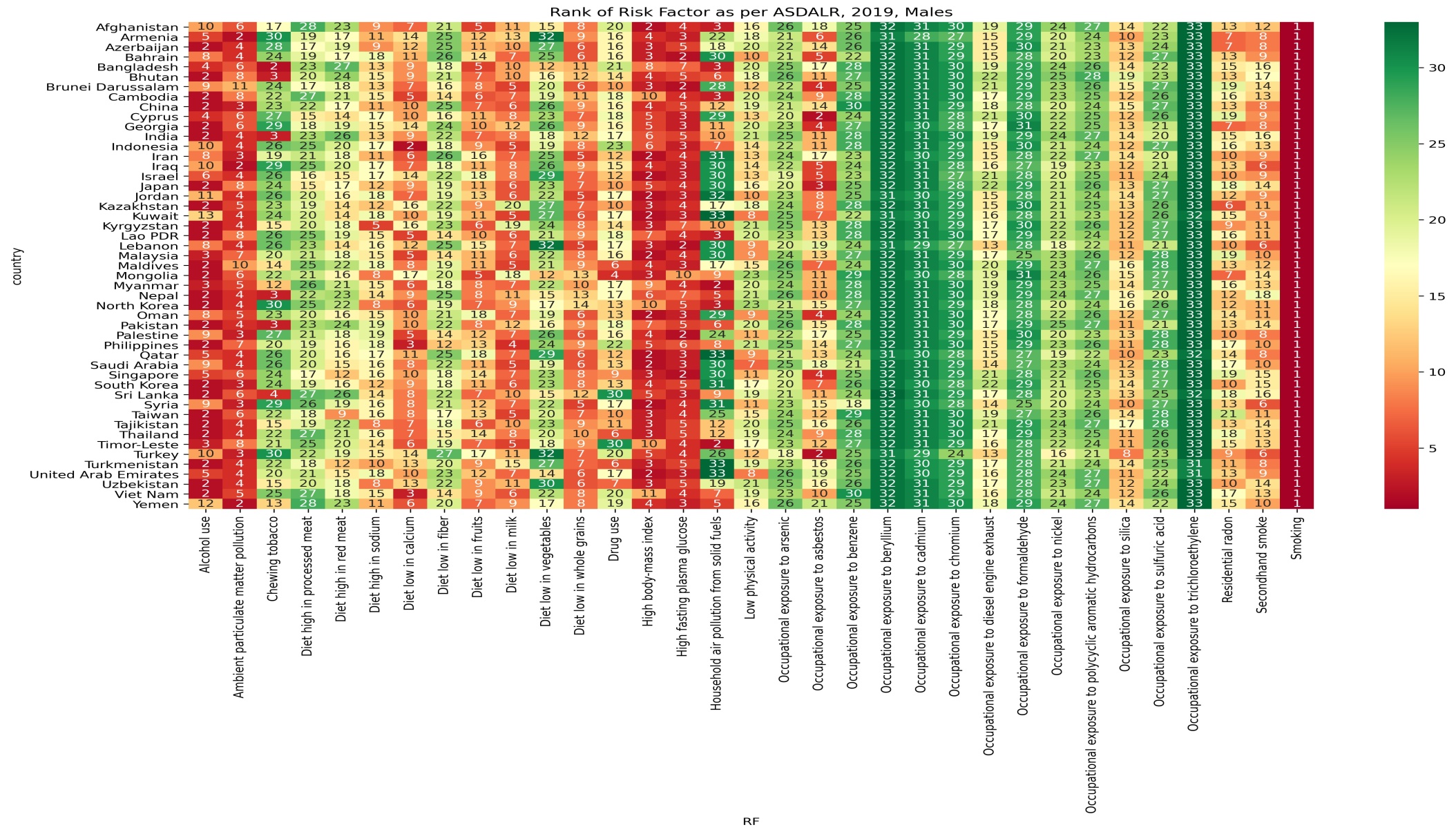
**

RF: Risk Factor; DALYs: Disability-adjusted life years. The numbers inside the cells represent the rank of risk factor in a country based on age-standardized DALYs rate in 2019.

**Supplementary Figure S21 Countrywise Rank of different Risk Factors as per Age-standardized DALYs Rate in 2019, Females**

**
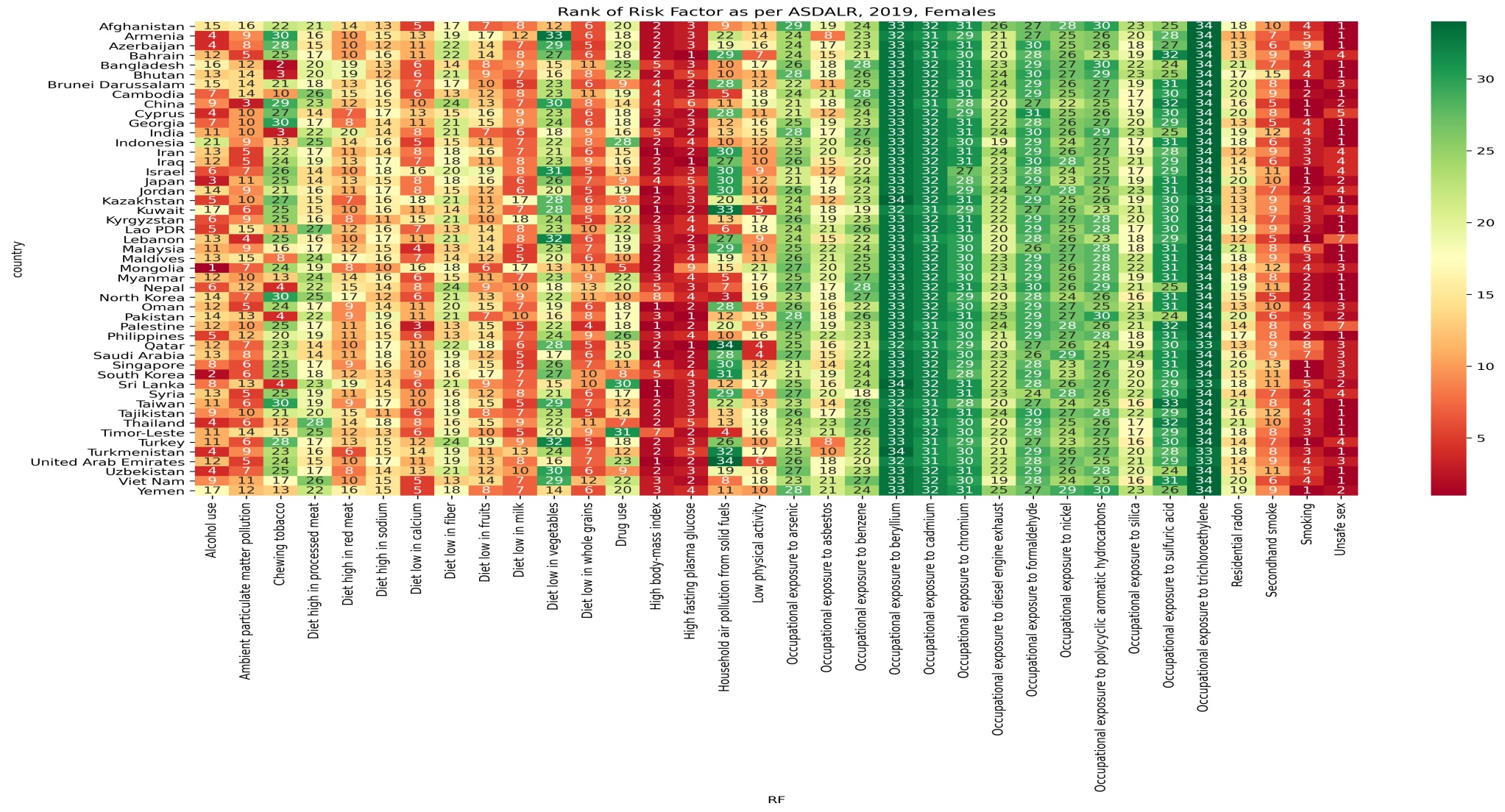
**

RF: Risk Factor; DALYs: Disability-adjusted life years. The numbers inside the cells represent the rank of risk factor in a country based on age-standardized DALYs rate in 2019.
